# 2-Fluoroenones via an Umpolung Morita–Baylis–Hillman Reaction of Enones

**DOI:** 10.1021/acs.orglett.3c00313

**Published:** 2023-02-13

**Authors:** Subrata Maity, Alex M. Szpilman

**Affiliations:** Department of Chemical Sciences, Ariel University, Ariel 4070000, Israel

## Abstract

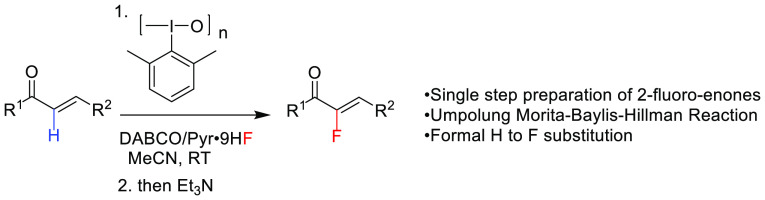

Several methods have been reported for the formation of 2-fluoroenones. However, all these methods involve laborious multiple-step sequences with resulting low overall yields. In this paper, we report the first formal enone-α-H to F substitution, leading to 2-fluoroenones in a single step from ubiquitous enones in 63–90% yield. The reaction is applicable to a wide range of aromatic and alkenyl enones and is carried out at room temperature using HF-pyridine complex as the fluoride source. Mechanistic investigations support that the reaction takes place through a rare umpolung Morita–Baylis–Hillman-type mechanism.

The proliferation of approved pharmaceuticals incorporating fluorine atoms^1−3^ has led to the development of numerous synthetic methods for the synthesis of fluorinated organic compounds. Of special relevance for this work is the ability of vinyl fluorides to act as enolate bioisosteres.^4^

2-Fluoroenones are interesting compounds with a large potential as building blocks for fluorinated medicines given their reactivity. 2-Fluoroenones undergo asymmetric hydrogenation to give chiral 2-fluoroketones,^5^ asymmetric Diels–Alder cycloaddition reactions,^6,7^ and participate readily in Meerwein arylations^8^ as well as conjugate addition reactions with a large variety of nucleophiles.^9^ Pannecoucke^10^ and Hoveyda^11^ demonstrated their conversion into biologically active 2-fluoro allylic amines.

Unfortunately, all reported syntheses of 2-fluoroenones require two- to five-step procedures from commercially available starting materials.

Even the shortest route of only two steps involves fluorination of enones with elementary fluorine followed by elimination and proceeds in a yield of 42–68%.^12^ Alternatively, Horner–Wadsworth–Emmons (HWE) reactions of aldehydes may be done with 2-fluoro-3-ketophosphonates.^13^ However, the preparation of the reagents for this HWE reaction require three to four steps.^13−15^ Another option is to carry out a HWE reaction between aldehydes and commercially available 2-fluorotriethylphosphonoacetate to give the corresponding esters followed by a two-step sequence to convert the ester to the ketone.^5,14,16^

2-Fluoroenones may also be prepared by selenium-catalyzed α-oxygenation of vinyl fluorides.^6a,9^ Addition of in situ-generated phenylselenyl fluoride (from PhSe-Br and AgF) to α-diazoketones and α-diazoesters was also reported.^17^ 1-Bromovinyl fluorides^18^ or 1-chlorovinyl fluorides^11^ may be coupled with alkoxyvinylzinc reagents using palladium catalysis. Again, these reactants are prepared in multistep sequences. Ketones may be fluorinated and then made to undergo an aldol condensation with aromatic aldehydes to give aromatic fluoroenones.^19^ Yet another approach to 2-fluoroenones involves the fluorination of 1,3-dicarbonyl compounds followed by a one-pot condensation–retro-Claisen reaction with an aldehyde.^20^

In comparison with all these multistep procedures, an operationally simple single-step preparation of 2-fluoroenones from readily available enones using commercially available reagents would be highly desirable.

Recently, umpolung via discrete iodine(III)–enolonium species^21^ has attracted much attention as a strategy to access new chemical reactions not possible through classical reactivity. In this context, we^22^ and others^23,24^ have reported extensively on the chemistry of iodine(III)–enolonium species, i.e., the electrophilic equivalent of classical lithium enolates. In 2020, we reported the first umpolung Morita–Baylis–Hillman reaction and showed that it could be used to prepare 1,2-diones and 2-tosylenones.^23d,25^ It occurred to us that this novel concept could be useful for accessing 2-fluoroenones from ubiquitous enones.

We therefore studied the reaction of enone **1** with various iodine(III) reagents (**2**),^26^ fluoride sources (**4**), and amine bases (**3**) (Table 1). The use of DIB (2 equiv) in conjunction with pyridine (1.5 equiv) with different inorganic fluoride salts failed to give any desired product **6** (Table 1, entries 1–3). Acetonitrile was used in order to ensure the solubility of DIB as well as partial solubility of these salts. Using more soluble organic salts such as TBAF or TBAT also failed (entries 4–6). However, using the inexpensive fluoride source triethylamine-HF complex (5 equiv) in combination with DIB (2 equiv) and using pyridine (1.5 equiv) as a MBH activator for the first time led to complete consumption of **1** and formation of a new product, later identified as **5**, as observed by TLC. Adding triethylamine to both neutralize excess HF and cause elimination of the amine leaving group of **5** led to the formation of the desired product **6** in 36% isolated yield (entry 7). Knowing the propensity of DIB to oxidize triethylamine as a side reaction,^25a^ we increased the amount of DIB in the reaction, but this decreased the yield to 30% (entry 8). Carrying out the reaction in dichloromethane led to a decrease in yield to 15% (entry 9). Replacing DIB with 2 equiv of PhI(OPiv)_2_ (entry 10) did not give any improvement. Exchanging DIB for PIFA, or Koser’s reagent, or TolIF_2_ led to no product formation at all (entries 11–13). However, using iodosyl benzene did lead to an improved yield of 55% (entry 14). A distinct issue is the low solubility of iodosyl benzene in most solvents. However, the protonated reagent generated by the action of the fluoride source Et_3_N·3HF does dissolve in acetonitrile. Using the more hindered and soluble 2-iodosyl-1,3-dimethylbenzene (2 equiv) improved the yield of **6** further to 67% (entry 15).

**Table 1:**
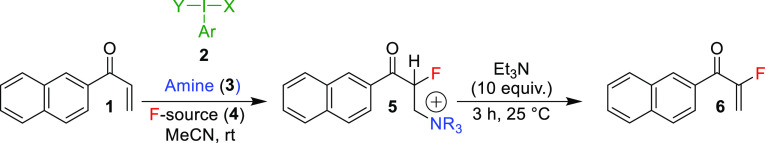
Optimization of the Reaction Conditions^a^

entry	amine **3** (1.5 equiv)	reagent **2** (equiv)	fluoride source (equiv)	yield (%)^b^
1	pyridine	PhI(OAc)_2_ (2)	KF (5)	0
2	pyridine	PhI(OAc)_2_ (2)	AgF_2_ (5)	0
3	pyridine	PhI(OAc)_2_ (2)	ZnF_2_ (5)	0
4	pyridine	PhI(OAc)_2_ (2)	TBAF (1 M in THF) (5)	0
5	pyridine	PhI(OAc)_2_ (2)	TBAF (solid) (5)	trace
6	pyridine	PhI(OAc)_2_ (2)	TBAT (5)	0
7	pyridine	PhI(OAc)_2_ (2)	Et_3_N·3HF (5)	36
8	pyridine	PhI(OAc)_2_ (4)	Et_3_N·3HF (5)	30
9^c^	pyridine	PhI(OAc)_2_ (2)	Et_3_N·3HF (3)	15
10	pyridine	PhI(OPiv)_2_ (2)	Et_3_N·3HF (5)	20
11	pyridine	PhI(O_2_CCF_3_)_2_ (2)	Et_3_N·3HF (5)	0
12	pyridine	PhI(OH)OTs (2)	Et_3_N·3HF (5)	0
13	pyridine	TolIF_2_ (2)	Et_3_N·3HF (5)	0
14	pyridine	PhIO (2)	Et_3_N·3HF (5)	55
15	pyridine	ArIO (2)^d^	Et_3_N·3HF (5)	67
16	pyridine	ArIO (2)^d^	Py·9HF (5)	76
17	pyridine	ArIO (2.5)^d^	Py·9HF (6)	82
18	pyridine	ArIO (2.5)^d^	Py·9HF (3)	46
19	DABCO	ArIO (1.6)^d^	Py·9HF (3)	60
20^c^	DABCO	ArIO (1.6)^d^	Py·9HF (3)	45
21^e^	DABCO	ArIO (1.6)^d^	Py·9HF (3)	40
22^c^	DABCO	ArIO (1.6)^d^	Py·9HF (5)	72
23	DABCO	ArIO (1.6)^d^	Et_3_N·3HF (5)	42
24	DABCO	ArIO (1.6)^d^	Py·9HF (5)	84

a All experiments were performed on a 0.549 mmol scale of **1** (1.0 equiv) with the specified iodine(III) reagent and fluoride source in 4.0 mL of MeCN at 25 °C.

b All yields are isolated yields.

c Using DCM as the solvent.

d 2-Iodosyl-1,3-dimethylbenzene.

e Using THF as the solvent.

The use of the pyridine-HF complex (Py·9HF) in lieu of the triethylamine-HF complex afforded **6** in 76% yield (entry 16). With slightly higher amounts of the pyridine-HF complex (6 equiv) and 2-iodosyl-1,3-dimethylbenzene (2.5 equiv), an 82% yield was achieved (entry 17). In contrast, lowering the amount of HF-pyridine to 3 equiv with 2.5 equiv of 2-iodosyl-1,3-dimethylbenzene reduced the yield of isolated **6** to only 46% (entry 18).

Finally, we tested DABCO, a known promoter of Morita–Baylis–Hillman reactions.^27^ Using 3 equiv of Py·9HF afforded **6** in 60% yield in acetonitrile (entry 19), 45% in DCM (entry 20), and 40% in THF (entry 21). With an increased amount of Py·9HF (5 equiv), a 72% yield of **6** could be obtained in DCM (entry 22). However, acetonitrile proved to be the optimal solvent, allowing preparation of **6** in 84% yield (entry 24). Using Et_3_N·3HF instead of Py·9HF was disadvantageous (entry 23).

The reaction mechanism remained ambiguous at this point in time. At least two likely mechanisms could be postulated. In one (Scheme 1, path b), formation of “an F^+^-type reagent”, such as ArI(OH)F, which could form in the reaction by the action of HF on ArI=O (see Scheme 1 for the structure), might lead to difluorination of enone **1** to give an intermediate difluoride product such as **9**. Based on our previous work,^25a^ a mechanism involving formation of a Morita–Baylis–Hillman enolonium species **7**, i.e., path a Scheme 1, was also suggested. Reaction with nucleophilic fluoride would then give intermediate **8**. Elimination with triethylamine would then afford the isolated product **6**.

**Scheme 1:**
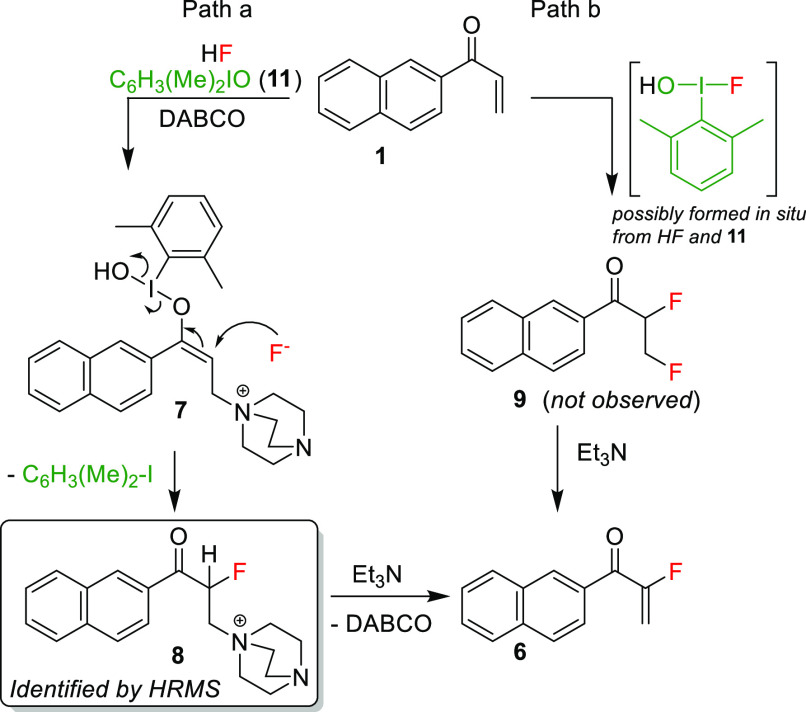
Simplified Possible Mechanisms

We therefore studied the reaction mixture by NMR and HRMS. ^1^H NMR results were similar to those we observed in our previous work (see the Supporting Information (SI)).^25a^ Conclusively, direct injection HRMS of the reaction mixture, before addition of triethylamine, clearly showed the presence of **8** but no trace of difluoro intermediate **9** (see the SI). Thus, path a is likely the mechanism of the reaction.

Next, we examined the scope of the reaction (Scheme 2). Enones with *E* geometry and methyl or ethyl substituents led to **13** and **14** in 70 and 79% yield, respectively, with the double bond geometry shown (Scheme 2). The latter reaction was carried out on a 1 mmol scale of **10** (R^1^ = 2-naphthyl, R^2^ = Et). In contrast, enone **10** (R^1^ = Ph, R^2^ = Ph) did not afford any product when subjected to the standard reaction conditions. Reaction of 1-phenylprop-2-en-1-one led to **15** in 78% yield.

**Scheme 2:**
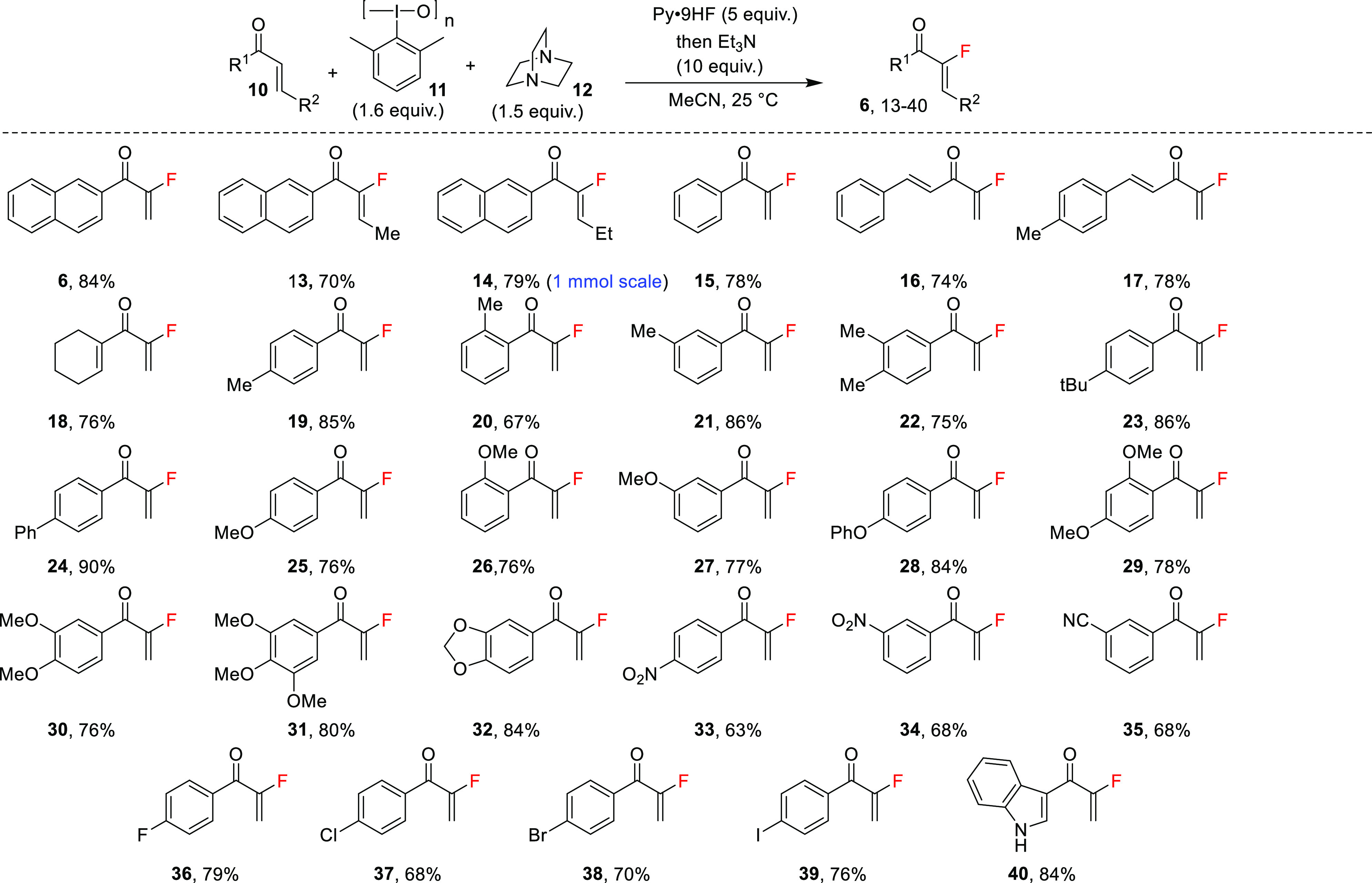
Scope of Enones in the Umpolung MBH Fluorination Reaction

Intriguingly, enones with an additional potentially reactive conjugated double bond underwent highly selective reactions in good yields at the less substituted double bond. Thus, enones with both a terminal double bond and an *E*-phenyl-substituted double bond reacted selectively to give **16** and **17** in 74 and 78% yield, respectively. Similarly, the cyclohexene-substituted 2-fluoroenone **18** could be prepared in 76% yield. In none of these three cases was the products of reaction at the more substituted double bond observed. This is further circumstantial support for a Morita–Baylis–Hillman mechanism.

Substituted 1-phenyl-2-enones with methyl in the *para-*, *ortho-*, or *meta*-positions of the phenyl group afforded vinyl fluorides **19**, **20**, and **21** in 85, 67, and 86% yield, respectively. This indicates that steric hindrance of an *ortho*-substituent could impair the successful reaction, while a *meta*-substituent would not affect the yield. Indeed, 1-mesitylprop-2-en-1-one was recovered unchanged when submitted to the reaction conditions. In contrast, two methyl groups in the *para*- and *meta-*positions are acceptable with **22** being produced in 75% yield. The *para-tert*-butyl-substituted 2-fluoroenone **23** was produced in 86% yield. Interestingly, phenyl-substituted **24** was formed in 90% yield.

Despite the oxidizing and acidic conditions of the reaction, enones with electron-donating groups are quite compatible with the reaction conditions. Thus, 2-fluoroenones **25**, **26**, and **27** with methoxy groups in the *para*-, *ortho*-, and *meta*-positions, respectively, were all isolated in similar yields of 76–77%, thus indicating that methyl ethers of phenol are quite compatible with the reaction’s conditions. Phenoxy-substituted 2-fluoroenone **28** was produced in 84% yield. Remarkably, two or even three methoxy-group-substituted benzenes were also tolerated despite being highly susceptible to both oxidation and acid hydrolysis. Thus, 2-fluoroenones **29**, **30**, and **31** could be prepared in 78, 76, and 80% yield, respectively. 2-Fluoroenone **32** with the adjacent oxygen atoms protected as a formaldehyde acetal could be synthesized in 84% yield.

Enones with a powerful electron-withdrawing nitro group might be less prone to oxidative conditions, and we therefore tested enones with nitro groups in the *para*- and *meta*-positions and were able to prepare **33** in 63% yield and **34** in 68% yield. Cyano-substituted fluoroenone **35** was successfully synthesized in 68% yield. Halogen atoms on the benzene ring were also compatible with **36**, **37**, **38**, and **39** being formed in 79, 68, 70, and 76% yield, respectively.

Interestingly, attempts to prepare **40** from the corresponding enone failed. However, when the *N*-tosyl-protected indole enone was used instead, the deprotected product **40** was isolated in 84% yield. Unprotected pyrrole-substituted enone also did not give the desired product. Additionally, alkyl-substituted enones such as (*E*)-2-hexen-3-one did not work in the reaction.

Adding to the known synthetic use of 2-fluoroenones,^5−11^ we demonstrated two synthetic transformations of 2-fluoroenones (Scheme 3). Thus, α-fluoroenone **6** was converted to corresponding alkyl alcohol **41** (full reduction) and allyl alcohol **42** (partial reduction) under Pd–C/H_2_ and NaBH_4_, respectively (Scheme 3). Esters of 2-fluoroallylic alcohols have been utilized in Claisen-type rearrangements^28^ and Tsuji-Trost allylic functionalization reactions.^29^

**Scheme 3:**
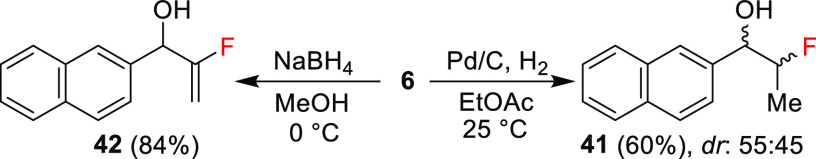
Selected Transformations

In conclusion, we have developed a single-step synthesis of 2-fluoroenones using HF-pyridine as a source of nucleophilic fluoride. The reaction likely takes place via an umpolung Morita–Baylis–Hillman mechanism.

## Data Availability

The data underlying this study are available in the published article and its online Supporting Information.

## Supporting Information Available

Experimental procedures, characterization data, and copies of NMR spectra (PDF)
