# Supplementary material for: 2-Fluoroenones via an Umpolung Morita–Baylis–Hillman Reaction of Enones
Source: Org Lett. 2023 Feb 13;25(7):1218–22. doi: 10.1021/acs.orglett.3c00313 (PMC9972470; doi:10.1021/acs.orglett.3c00313)
Supplement: Supplementary file 1 — ol3c00313_si_001.pdf [file ol3c00313_si_001.pdf]

Supporting Information

2-Fluoro-Enones via an Umpolung Morita-Baylis-Hilman Reaction of Enones

Subrata Maity[a] and Alex M. Szpilman*[a]

[a]Department of Chemical Sciences, Ariel University, Ariel 4070000, Israel.

Corresponding authors' Email: Szpilman@ariel.ac.il

List of Contents

General Information S1

General procedure **A** for the Umpolung MBH Fluorination Reaction S2

Yields and Characterization data of *2*-Fluoro-enones S3

Procedure for the synthesis of as well as Characterization data for compounds **41** & **42** S15

HRMS Analysis of Crude Reaction Mixture S16

General procedures B and C for the synthesis of *α*, *β*-unsaturated carbonyl compounds S17

Yields and Characterization data for α, β-unsaturated carbonyl compounds S19

Synthesis of 2-Iodosyl-1,3-dimethylbenzene (**11**) S27

References S28

Copies of NMR spectra S29

NMR Spectra of the Crude Reaction Mixture Before and After Addition of Triethylamine S91

General Information: All the reactions were carried out using oven dried (temp of oven kept at 120 °C) glassware under an atmosphere of Argon (Ar). Room temperature refers to 20–25 °C. All reagents were used as purchased from commercial supplier without further purification. Solvents were dried and distilled following usual protocols. Flash column chromatography was performed in all cases using the indicated solvent system on silica gel (230-400 mesh) purchased. Analytical thin layer chromatography was performed using 60 F254 precoated silica gel plate (TLC Silica gel 60 F254 from Merck) (0.2 mm thickness). Compounds were visualized by irradiation of UV light and developed with Iodine and $KMnO_4$ stains, followed by heating if necessary. The ^{1}H NMR, ^{13}C NMR (proton decoupling) and ^{19}F NMR (proton decoupling) spectra measurements were carried out on Bruker 400 MHz, 101 MHz and 376 MHz NMR spectrometers with $CDCl_3$ or DMSO-d_6 or CD_3CN. Data for ^{1}H, ^{13}C, ^{19}F were recorded as follows: chemical shift (δ, ppm), multiplicity (s = singlet, d = doublet, t = triplet, q = quartet, m = multiplet, dd = doublet of doublets, ddd = doublet of doublet of doublets, dt = doublet of triplets, dq = doublet of quartets, td = triplet of doublets, br. s = broad singlet). High resolution mass spectra (HRMS) were obtained using a Waters Xevo G2-XS-QTOF instrument. Fourier transform infrared (FTIR) spectra were recorded on a JASCO FT-IR spectrometer (ATR) in the 500-3600 Cm^{-1} region.

General procedure A for the Umpolung MBH Fluorination Reaction:

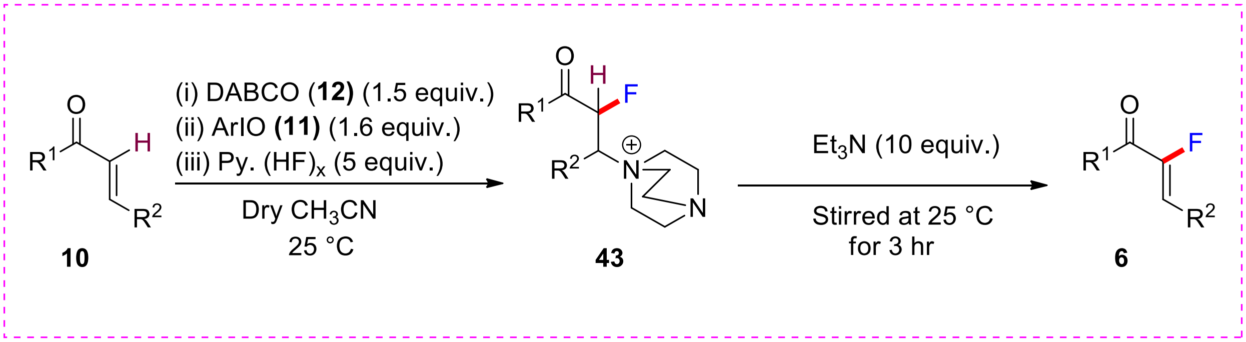

To a stirring solution of 1-(Naphthalen-2-yl) prop-2-en-1-one **1** (0.100 g, 0.548 mmol) in a Falcon tube (Teflon coated) in dry acetonitrile (4 mL) at 25 °C was added **DABCO** (0.092 g, 0.823 mmol, 1.5 eq) under a nitrogen atmosphere. The mixture was stirred at the same temperature for 5 minutes under a nitrogen atmosphere. Then **2-Iodosyl-1,3-dimethylbenzene**[1] (0.217 g, 0.877 mmol, 1.6 equiv.) and **Py•9HF** (0.247 mL, 2.74 mmol, 5 equiv.) were added to the reaction mixture sequentially. The resulting homogeneous mixture was stirred at the same temperature and monitored by TLC. On completion (14-18 hours), **triethyl amine** (0.765 mL, 5.485 mmol, 10 equiv.) was added to the reaction mixture and stirred for another 3 hr at the same temperature. Solvent was evaporated under reduced pressure and the crude residue was purified by silica gel (230-400) flash chromatography using (6% EtOAc in hexane as eluent) to afford the desire product **6** (0.092 g, 84%).

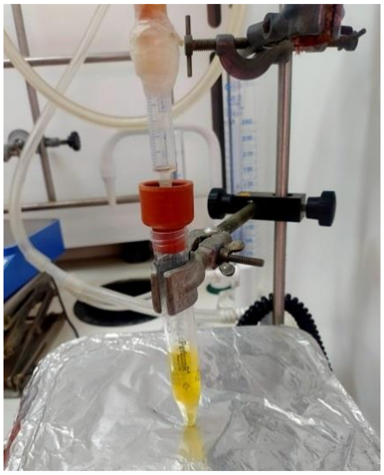

Figure 1: Reaction setup for the Umpolung MBH Fluorination Reaction.

<u>**Characterization data for 2-Fluoro-enones**</u>

2-Fluoro-1-(naphthalen-2-yl) prop-2-en-1-one (6): The product was prepared by following general procedure **A** and was obtained as white solid (0.092 g, 84%). The compound was purified

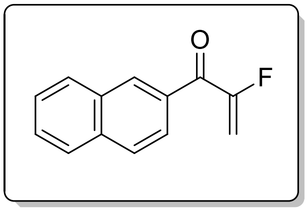

by silica gel (230-400) flash chromatography using 6% EtOAc in hexane as eluent. The spectral data was identical to the previous literature report.[4] **FT-IR** (ν cm^{-1}): 3050, 1662, 1627, 1466, 1377, 1250, 1165, 1118, 918, 775. **^{1}H NMR** (400 MHz, $CDCl_3$): δ 8.45 (s, 1H), 7.98 – 7.89 (m, 4H), 7.66 – 7.56 (m, 2H), 5.65 (dd, *J* = 45.2, 3.6 Hz, 1H), 5.56 (dd, *J* = 15.0, 3.4 Hz, 1H). **^{13}C NMR** (101 MHz, $CDCl_3$): δ 187.1 (d, *J* = 29.4 Hz, 1C), 160.1 (d, *J* = 269.9 Hz, 1C), 135.6, 132.7, 132.2, 131.4 (d, *J* = 5.3 Hz, 1C), 129.6, 128.8, 128.5, 127.8, 127.0, 124.8 (d, *J* = 2.7 Hz, 1C), 104.3 (d, *J* = 16.5 Hz, 1C). **^{19}F NMR** (376 MHz, $CDCl_3$): δ -110.1. **HRMS** (ESI/Q-TOF) m/z: [M+H]$^+$ Calcd for $C_{13}H_{10}FO^+$ 201.0710; found 201.0706.

(Z)-2-fluoro-1-(naphthalen-2-yl) but-2-en-1-one (13): The reaction was performed 0.7643 mmole scale. The product was prepared by following general procedure **A** and was obtained as

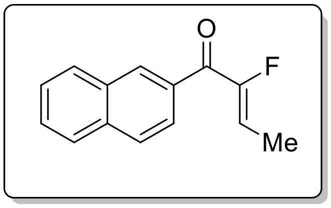

colorless gummy liquid (0.114 g, 70%). The reaction time is 48 hr. The compound was purified by silica gel (230-400) flash chromatography using 4% EtOAc in hexane as eluent. **FT-IR** (ν cm^{-1}): 3058, 2923, 2854, 2742, 2302, 1936, 1816, 1658, 1504, 1457, 1457, 1280, 998, 914, 829. **^{1}H NMR** (400 MHz, $CDCl_3$): δ 8.36 (s, 1H), 7.95 – 7.85 (m, 4H), 7.62 – 7.53 (m, 2H), 6.24 – 6.10 (m, 1H), 1.93 (dt, *J* = 7.6, 2.6 Hz, 3H). **^{13}C NMR** (101 MHz, $CDCl_3$): δ 187.2 (d, *J* = 28.1 Hz, 1C), 156.3 (d, *J* = 261.9 Hz, 1C), 135.3, 133.4, 132.2, 130.8 (d, *J* = 5.0 Hz, 1C), 129.4, 128.5, 128.3, 127.8, 126.9, 125.0 (d, *J* = 2.7 Hz, 1C), 119.0 (q, *J* = 3.4 Hz, 1C), 10.1 (d, *J* = 5.2 Hz, 1C). **^{19}F NMR** (376 MHz, $CDCl_3$): δ -124.5. **HRMS** (ESI/Q-TOF) m/z: [M+H]$^+$ Calcd for $C_{14}H_{12}FO^+$ 215.0867; found 215.0856.

(Z)-2-Fluoro-1-(naphthalen-2-yl) pent-2-en-1-one (14): The reaction was performed 1.046

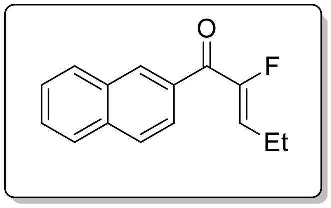

mmole scale. The product was prepared by following general procedure **A** and was obtained as colorless gummy liquid (0.188 g, 79%). The reaction time is 48 hr. The compound was purified by silica gel (230-400) flash chromatography using 3% ether in hexane as

eluent. **FT-IR** (ν cm^{-1}): 3058, 2973, 1662, 1465, 1357, 1288. **^{1}H NMR** (400 MHz, $CDCl_3$): δ 8.36 (s, 1H), 7.97 – 7.86 (m, 4H), 7.63 – 7.54 (m, 2H), 6.11 (dt, *J* = 34.4, 7.7 Hz, 1H), 2.45 – 2.37 (m, 2H), 1.14 (t, *J* = 7.6 Hz, 3H). **^{13}C NMR** (101 MHz, $CDCl_3$): δ 187.5 (d, *J* = 28.3 Hz, 1C), 155.0 (d, *J* = 261.8 Hz, 1C), 135.3, 133.5 , 132.2, 130.9 (d, *J* = 5.2 Hz, 1C), 129.4, 128.5, 128.3, 127.8, 126.9, 125.5 (d, *J* = 13.0 Hz, 1C), 125.1 (d, *J* = 2.7 Hz, 1C), 18.2 (d, *J* = 4.0 Hz, 1C), 13.0 (d, *J* = 1.9 Hz, 1C). **^{19}F NMR** (376 MHz, $CDCl_3$): δ -124.5. **HRMS** (ESI/Q-TOF) m/z: [M+H] $^+$ Calcd for $C_{15}H_{14}FO^+$ 229.1023; found 229.1024.

2-Fluoro-1-phenylprop-2-en-1-one (15): The product was prepared by following general procedure **A** and was obtained as colorless gummy liquid (0.088 g, 78%). The compound was

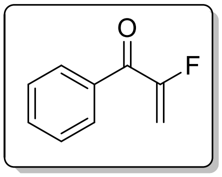

purified by silica gel (230-400) flash chromatography using 5% EtOAc in hexane as eluent. **FT-IR** (ν cm^{-1}): 3024, 1697, 1600, 1450, 1211, 1145, 1064, 933, 748. **^{1}H NMR** (400 MHz, $CDCl_3$): δ 7.88 (d, *J* = 4.0 Hz, 2H), 7.61 (t, *J* = 8.0 Hz, 1H), 7.49 (t, *J* = 8.0 Hz, 2H), 5.58 (dd, *J* = 40.0, 4.0 Hz, 1H), 5.52 – 5.49 (m, 1H). **^{13}C NMR** (101 MHz, $CDCl_3$): δ 187.3 (d, *J* = 30.3 Hz, 1C), 159.9 (d, *J* = 269.7 Hz, 1C), 135.5, 133.3, 129.4 (d, *J* = 3.0 Hz, 1C), 128.5, 104.3 (d, *J* = 16.2 Hz, 1C). **^{19}F NMR** (376 MHz, $CDCl_3$): δ -110.8. **HRMS** (ESI/Q-TOF) m/z: [M+H] $^+$ Calcd for $C_9H_8FO^+$ 151.0554; found 151.0552.

(*E*)-4-fluoro-1-phenylpenta-1,4-dien-3-one (16): The product was prepared by following general procedure **A** and was obtained as yellow solid (0.082 g, 74%). The compound was purified by silica gel (230-400) flash chromatography using 8% EtOAc in hexane as eluent. **FT-IR** (ν cm^{-1}):

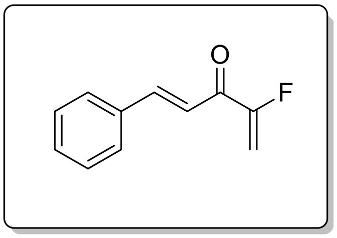

3127, 3027, 2927, 2854, 2672, 1643, 1600, 1504, 1450, 1353, 1257, 1211, 1072, 983, 933, 898. **^{1}H NMR** (400 MHz, $CDCl_3$): δ 7.67 (dd, *J* = 15.6, 0.4 Hz, 1H), 7.46 – 7.44 (m, 2H), 7.28 – 7.21 (m, 3H), 7.08 – 7.04 (m, 1H), 5.53 (dd, *J* = 45.6, 3.2 Hz, 1H), 5.13 (dd, *J* = 14.4, 3.2 Hz, 1H). **^{13}C NMR** (101 MHz, $CDCl_3$): δ 182.9 (d, *J* = 31.8 Hz, 1C), 160.6 (d, *J* = 269.7 Hz, 1C), 146.2 (d, *J* = 2.2 Hz, 1C), 134.3, 131.1, 129.0, 128.8, 119.2, 100.9 (d, *J* = 16.5 Hz, 1C). **^{19}F NMR** (376 MHz, $CDCl_3$): δ -117.3. **HRMS** (ESI/Q-TOF) m/z: [M+H] $^+$ Calcd for $C_{11}H_{10}FO^+$ 177.0710; found 177.0697.

(*E*)-4-fluoro-1-(p-tolyl) penta-1,4-dien-3-one (17): The product was prepared by following general procedure **A** and was obtained as brown solid (0.086 g, 78%). The compound was purified by silica gel (230-400) flash chromatography using 8% EtOAc in hexane as eluent. **FT-IR** (ν cm^{-1}): 3127, 3023, 2923, 2854, 1643, 1592, 1511, 1349, 1253, 1214, 1060. **^{1}H NMR** (400 MHz, $CDCl_3$): δ 7.84 (d, *J* = 15.6 Hz, 1H), 7.53 (d, *J* = 8.0 Hz, 2H), 7.24 – 7.17 (m, 3H), 5.70 (dd, *J* = 45.6, 3.2 Hz, 1H), 5.29 (dd, *J* = 14.8, 3.2 Hz, 1H), 2.40 (s, 3H). **^{13}C NMR** (101 MHz, $CDCl_3$): δ 182.9 (d, *J* = 31.9 Hz, 1C), 160.7 (d, *J* = 272.6 Hz, 1C), 146.3 (d, *J* = 2.2 Hz, 1C), 141.8, 131.6, 129.8, 128.8, 118.2, 100.7 (d, *J* = 16.8 Hz, 1C), 21.6. **^{19}F NMR** (376 MHz, $CDCl_3$): δ -117.2. **HRMS** (ESI/Q-TOF) m/z: [M+Na]$^+$ Calcd for $C_{12}H_{11}FNaO^+$ 213.0686; found 213.0706.

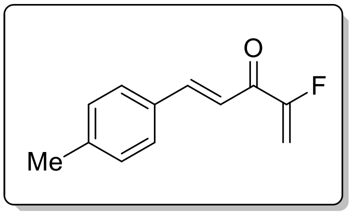

1-(Cyclohex-1-en-1-yl)-2-fluoroprop-2-en-1-one (18): The product was prepared by following general procedure **A** and was obtained as colorless gummy liquid (0.086 g, 76%). The compound was purified by silica gel (230-400) flash chromatography using 4% EtOAc in hexane as eluent. **FT-IR** (ν cm^{-1}): 3131, 3046, 2935, 2861, 1631, 1438, 1384, 1276, 1172, 987, 925, 894. **^{1}H NMR** (400 MHz, $CDCl_3$): δ 6.94 (td, *J* = 3.8, 1.9 Hz, 1H), 5.36 (dd, *J* = 46.0, 3.2 Hz, 1H), 5.26 (dd, *J* = 15.8, 3.0 Hz, 1H), 2.32 – 2.27 (m, 4H), 1.72 – 1.62 (m, 4H). **^{13}C NMR** (101 MHz, $CDCl_3$): δ 187.9 (d, *J* = 27.9 Hz, 1C), 160.0 (d, *J* = 270.9 Hz, 1C), 143.5 (d, *J* = 3.2 Hz, 1C), 137.3, 101.8 (d, *J* = 16.9 Hz), 26.1, 23.6 (d, *J* = 1.0 Hz, 1C), 21.7, 21.4. **^{19}F NMR** (376 MHz, $CDCl_3$): δ -109.2. **HRMS** (ESI/Q-TOF) m/z: [M+H]$^+$ Calcd for $C_9H_{12}FO^+$ 155.0867; found 155.0869.

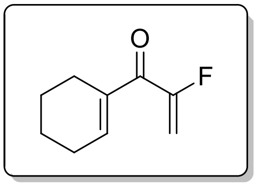

2-Fluoro-1-(*p*-tolyl) prop-2-en-1-one (19): The product was prepared by following general procedure **A** and was obtained as white liquid (0.95 g, 85%). The compound was purified by silica gel (230-400) flash chromatography using 7% EtOAc in hexane as eluent. **FT-IR** (ν cm^{-1}): 3024, 2927, 1670, 1604, 1277, 748. **^{1}H NMR** (400 MHz, $CDCl_3$): δ 7.81 – 7.79 (m, 2H), 7.29 – 7.28 (m, 2H), 5.55 (dd, *J* = 44.8, 2.8 Hz, 1H), 5.47 (dd, *J* = 14.6, 3.0 Hz, 1H), 2.44 (s, 3H). **^{13}C NMR** (101 MHz, $CDCl_3$): δ 186.9 (d, *J* = 29.3 Hz, 1C), 160.1 (d, *J* = 269.7 Hz, 1C), 144.4, 132.9, 129.6 (d, *J* = 4.0

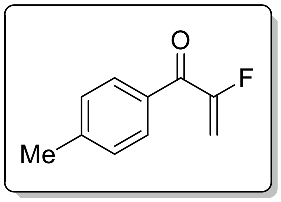

Hz, 1C), 129.2, 103.8 (d, J = 16.2 Hz, 1C), 21.7. **^{19}F NMR** (376 MHz, $CDCl_3$): δ -110.4 (s). **HRMS** (ESI/Q-TOF) m/z: $[M+H]^+$ Calcd for $C_{10}H_{10}FO^+$ 165.0710; found 165.0705.

2-Fluoro-1-(o-tolyl) prop-2-en-1-one (20): The product was prepared by following general procedure **A** and was obtained as colorless liquid (0.075 g, 67%). The reaction time is 48 hr. The

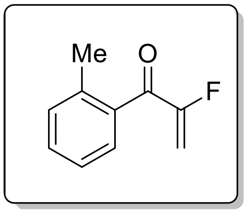

compound was purified by silica gel (230-400) flash chromatography using 6% EtOAc in hexane as eluent. **FT-IR** (ν cm^{-1}): 3029, 2865, 2572, 1681, 1604, 1454, 1214. **^{1}H NMR** (400 MHz, $CDCl_3$): δ 7.28 – 7.24 (m, 2H), 7.14 – 7.09 (m, 2H), 5.43 (dd, J = 12.0, 4.0 Hz, 1H), 5.25 (dd, J = 44.0, 4.0 Hz, 1H), 2.25 (s, 3H). **^{13}C NMR** (101 MHz, $CDCl_3$): δ 190.2 (d, J = 28.3 Hz, 1C), 160.0 (d, J = 267.7 Hz, 1C), 137.32, 137.31, 136.0 (d, J = 2.0 Hz, 1C), 131.2 (d, J = 3.0 Hz, 1C), 128.4 (d, J = 2.0 Hz, 1C), 125.3, 106.2 (d, J = 17.2 Hz, 1C), 19.6. **^{19}F NMR** (376 MHz, $CDCl_3$): δ -113.7. **HRMS** (ESI/Q-TOF) m/z: $[M+H]^+$ Calcd for $C_{10}H_{10}FO^+$ 165.0710; found 165.0708.

2-Fluoro-1-(m-tolyl) prop-2-en-1-one (21): The product was prepared by following general procedure **A** and was obtained as colorless liquid (0.096 g, 86%). The compound was purified by

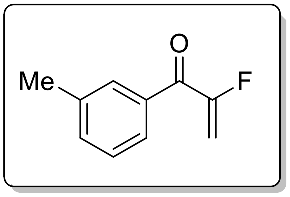

silica gel (230-400) flash chromatography using 6% EtOAc in hexane as eluent. **FT-IR** (ν cm^{-1}): 2927, 2873, 1674, 1600, 1512, 1431, 1288, 1238, 1157, 802, 752. **^{1}H NMR** (400 MHz, $CDCl_3$): δ 7.67 – 7.65 (m, 2H), 7.43 – 7.34 (m, 2H), 5.57 (dd, J = 35.4, 3.4 Hz, 1H), 5.47 (q, J = 3.6 Hz, 1H), 2.42 (s, 3H). **^{13}C NMR** (101 MHz, $CDCl_3$): δ 187.6 (d, J = 29.3 Hz, 1C), 159.9 (d, J = 269.7 Hz, 1C), 138.4, 135.6, 134.1, 129.8 (d, J = 4.0 Hz, 1C), 128.3, 126.6 (d, J = 4.0 Hz, 1C), 104.3 (d, J = 17.2 Hz, 1C), 21.3. **^{19}F NMR** (376 MHz, $CDCl_3$): δ -110.8. **HRMS** (ESI/Q-TOF) m/z: $[M+H]^+$ Calcd for $C_{10}H_{10}FO^+$ 165.0710; found 165.0709.

1-(3,4-Dimethylphenyl)-2-fluoroprop-2-en-1-one (22): The reaction was performed 0.7677 mmol scale. The product was prepared by following general procedure **A** and was obtained as

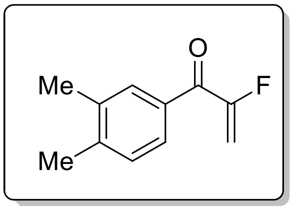

yellow liquid (0.103 g, 75%). The compound was purified by silica gel (230-400) flash chromatography using 6% EtOAc in hexane as eluent. **FT-IR** (ν cm^{-1}): 3023, 2973, 1909, 1670, 1608, 1404, 1446, 1411, 1365, 1276, 1218, 1126, 833, 760. **^{1}H NMR** (400 MHz, $CDCl_3$): δ 7.66 (s, 1H),

7.63 (d, J = 8.0 Hz, 1H), 7.23 (d, J = 8.0 Hz, 1H), 5.54 (dd, J = 43.2, 2.4 Hz, 1H), 5.46 (dd, J = 13.0, 2.2 Hz, 1H), 2.34 (s, 3H), 2.33 (s, 3H). **^{13}C NMR** (101 MHz, $CDCl_3$): δ 187.1 (d, J = 29.3 Hz, 1C), 160.1 (d, J = 269.7 Hz, 1C), 143.1, 137.0, 133.3, 130.5 (d, J = 4.0 Hz, 1C), 129.7, 127.2 (d, J = 4.0 Hz, 1C), 103.8 (d, J = 16.2 Hz, 1C), 20.0, 19.7. **^{19}F NMR** (376 MHz, $CDCl_3$): δ -110.3. **HRMS** (ESI/Q-TOF) m/z: [M+H]$^+$ Calcd for $C_{11}H_{12}FO^+$ 179.0867; found 179.0872.

1-(4-(Tert-butyl) phenyl)-2-fluoroprop-2-en-1-one (23): The product was prepared by following general procedure **A** and was obtained as colorless liquid (0.094 g, 86%). The compound

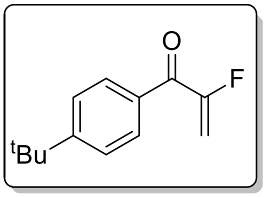

was purified by silica gel (230-400) flash chromatography using 8% EtOAc in hexane as eluent. The spectral data was identical to the previous literature report.[4] **FT-IR** (ν cm^{-1}): 2962, 1674, 1604, 1365, 1284, 1211, 752. **^{1}H NMR** (400 MHz, $CDCl_3$): δ 7.86 – 7.82 (m, 2H), 7.52 – 7.49 (m, 2H), 5.57 (dd, J = 45.2, 3.2 Hz, 1H), 5.48 (dd, J = 15.2, 3.2 Hz, 1H), 1.36 (s, 9H). **^{13}C NMR** (101 MHz, $CDCl_3$): δ 186.9 (d, J = 29.3 Hz, 1C), 160.1 (d, J = 269.7 Hz, 1C), 157.3, 132.8, 129.5 (d, J = 4.0 Hz, 1C), 125.5, 103.9 (d, J = 17.2 Hz, 1C), 35.2, 31.0. **^{19}F NMR** (376 MHz, $CDCl_3$): δ -110.5. **HRMS** (ESI/Q-TOF) m/z: [M+H]$^+$ Calcd for $C_{13}H_{16}FO^+$ 207.1180; found 207.1189.

1-([1, 1'-Biphenyl]-4-yl)-2-fluoroprop-2-en-1-one (24): The product was prepared by following general procedure **A** and was obtained as white solid (0.098 g, 90%). The compound was purified

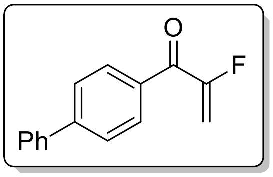

by silica gel (230-400) flash chromatography using 7% EtOAc in hexane as eluent. The spectral data was identical to the previous literature report.[4] **FT-IR** (ν cm^{-1}): 3047, 2927, 2858, 2310, 1948, 1809, 1666, 1600, 1400, 1284, 1180, 972, 926, 848, 748, 694. **^{1}H NMR** (400 MHz, $CDCl_3$): δ 8.00 – 7.97 (m, 2H), 7.73 – 7.70 (m, 2H), 7.66 – 7.64 (m, 2H), 7.52 – 7.47 (m, 2H), 7.45 – 7.41 (m, 1H), 5.63 (dd, J = 45.2, 3.2 Hz, 1H), 5.52 (dd, J = 15.0, 3.4 Hz, 1H). **^{13}C NMR** (101 MHz, $CDCl_3$): δ 186.8 (d, J = 30.3 Hz, 1C), 161.0 (d, J = 270.7.0 Hz, 1C), 146.2, 139.7, 134.1, 130.1 (d, J = 4.0 Hz), 129.0, 128.4, 127.3, 127.2, 104.1 (d, J = 16.2 Hz, 1C). **^{19}F NMR** (376 MHz, $CDCl_3$): δ -110.5. **HRMS** (ESI/Q-TOF) m/z: [M+H]$^+$ Calcd for $C_{15}H_{12}FO^+$ 227.0867; found 227.0869.

2-Fluoro-1-(4-methoxyphenyl) prop-2-en-1-one (25): The product was prepared by following general procedure **A** and was obtained as colorless gummy liquid (0.084 g, 76%). The compound was purified by silica gel (230-400) flash chromatography using 15% EtOAc in hexane as eluent. The spectral data was identical to the previous literature report.[4] **FT-IR** (*ν* cm^{-1}): 2966, 2843, 1724,

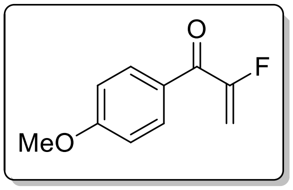

1666, 1597, 1512, 1423, 1365, 1311, 1257, 1168, 1026, 845, 775. **^{1}H NMR** (400 MHz, $CDCl_3$): δ 7.95 – 7.91 (m, 2H), 6.98 – 6.94 (m, 2H), 5.55 (dd, *J* = 48.0, 4.0 Hz, 1H), 5.42 (dd, *J* = 16.0, 4.0 Hz, 1H), 3.89 (s, 3H). **^{13}C NMR** (101 MHz, $CDCl_3$): δ 185.5 (d, *J* = 29.3 Hz, 1C), 163.9, 160.4 (d, *J* = 270.7 Hz, 1C), 132.0 (d, *J* = 5.0 Hz, 1C), 128.1, 113.8, 103.0 (d, *J* = 16.2 Hz), 55.5. **^{19}F NMR** (376 MHz, $CDCl_3$): δ -109.5. **HRMS** (ESI/Q-TOF) m/z: $[M+H]^+$ Calcd for $C_{10}H_{10}FO_2^+$ 181.0659; found 181.0644.

2-Fluoro-1-(2-methoxyphenyl) prop-2-en-1-one (26): The product was prepared by following general procedure **A** and was obtained as white liquid (0.084 g, 76%). The compound was purified by silica gel (230-400) flash chromatography using 12% EtOAc in hexane as eluent. **FT-IR** (*ν*

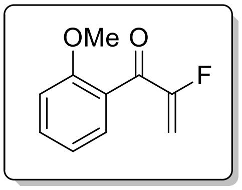

cm^{-1}): 2950, 2842, 1682, 1597, 1485, 1466, 1250, 1188, 930, 752. **^{1}H NMR** (400 MHz, $CDCl_3$): δ 7.50 – 7.46 (m, 1H), 7.39 – 7.37 (m, 1H), 7.04 – 6.97 (m, 2H), 5.46 (dd, *J* = 24.0, 4.0 Hz, 1H), 5.39 (dd, *J* = 8.0, 4.0 Hz, 1H), 3.85 (s, 3H). **^{13}C NMR** (101 MHz, $CDCl_3$): δ 188.6 (d, *J* =32.3 Hz, 1C), 160.4 (d, *J* = 268.7 Hz, 1C), 157.8, 132.9, 129.6, 126.6, 120.5, 111.5, 103.6 (d, *J* = 17.2 Hz, 1C), 55.7. **^{19}F NMR** (376 MHz, $CDCl_3$): δ -115.3. **HRMS** (ESI/Q-TOF) m/z: $[M+H]^+$ Calcd for $C_{10}H_{10}FO_2^+$ 181.0659; found 181.0670.

2-Fluoro-1-(3-methoxyphenyl) prop-2-en-1-one (27): The product was prepared by following general procedure **A** and was obtained as colorless liquid (0.085 g, 77%). The compound was

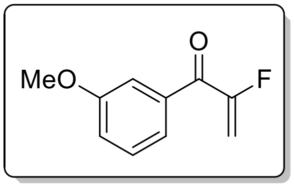

purified by silica gel (230-400) flash chromatography using 12% EtOAc in hexane as eluent. The spectral data was identical to those reported in the literature.[4] **FT-IR** (*ν* cm^{-1}): 2950, 2842, 1689, 1581, 1461, 1288, 1246, 1142, 1049. **^{1}H NMR** (400 MHz, $CDCl_3$): δ 7.48 – 7.45 (m, 1H), 7.41 – 7.37 (m, 2H), 7.15 (ddd, *J* = 8.4, 2.7, 1.1 Hz, 1H), 5.59 (dd, *J* = 39.0, 3.4 Hz, 1H), 5.51 (dd, *J* = 9.4, 3.4 Hz, 1H), 3.87 (s, 3H). **^{13}C NMR** (101 MHz, $CDCl_3$): δ 187.1 (d, *J* = 29.3 Hz, 1C),

159.8 (d, *J* =268.7 Hz, 1C), 159.6, 136.8, 129.5, 121.9 (d, *J* = 4.0 Hz), 119.8, 113.8 (d, *J* = 3.0 Hz), 104.5 (d, *J* = 16.2 Hz, 1C), 55.5. ^{19}**F NMR** (376 MHz, $CDCl_3$): δ -110.8. **HRMS** (ESI/Q-TOF) m/z: $[M+H]^+$ Calcd for $C_{10}H_{10}FO_2^+$ 181.0659; found 181.0662.

2-Fluoro-1-(4-phenoxyphenyl) prop-2-en-1-one (28): The product was prepared by following general procedure **A** and was obtained as colorless gummy liquid (0.091 g, 84%). The compound

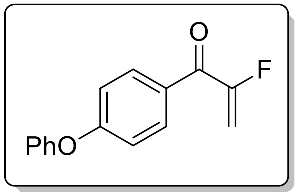

was purified by silica gel (230-400) flash chromatography using 6% EtOAc in hexane as eluent. The spectral data was identical to those reported in the literature.[4] **FT-IR** (*ν* cm^{-1}): 3062, 2040, 1666, 1578, 1493, 1242, 1161, 976, 760, 690. ^{1}**H NMR** (400 MHz, $CDCl_3$): δ 7.94 – 7.90 (m, 2H), 7.45 – 7.40 (m, 2H), 7.25 – 7.21 (m, 1H), 7.11 – 7.08 (m, 2H), 7.04 – 7.00 (m, 2H), 5.58 (dd, *J* = 44.0, 4.0 Hz, 1H), 5.45 (dd, *J* = 12.0, 4.0 Hz, 1H). ^{13}**C NMR** (101 MHz, $CDCl_3$): δ 185.6 (d, *J* = 30.3 Hz, 1C), 162.5, 160.2 (d, *J* = 270.7 Hz, 1C), 155.2, 131.9 (d, *J* = 5.0 Hz, 1C), 130.1, 129.7, 124.8, 120.3, 117.1, 103.4 (d, *J* = 17.2 Hz, 1C). ^{19}**F NMR** (376 MHz, $CDCl_3$): δ -109.8. **HRMS** (ESI/Q-TOF) m/z: $[M+H]^+$ Calcd for $C_{15}H_{12}FO_2^+$ 243.0816; found 243.0816.

1-(2,4-Dimethoxyphenyl)-2-fluoroprop-2-en-1-one (29): The product was prepared by following general procedure **A** and was obtained as colorless gummy liquid (0.085 g, 78%). The

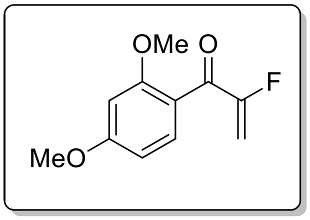

compound was purified by silica gel (230-400) flash chromatography using 15% EtOAc in hexane as eluent. **FT-IR** (*ν* cm^{-1}): 3024, 2958, 2931, 1682, 1454, 1215. ^{1}**H NMR** (400 MHz, $CDCl_3$): δ 7.43 (d, *J* = 8.4 Hz, 1H), 6.53 (dd, *J* = 8.6, 2.2 Hz, 1H), 6.48 (d, *J* = 2.4 Hz, 1H), 5.41 (dd, *J* = 44.4, 3.2 Hz, 1H), 5.31 (dd, *J* = 13.8, 3.0 Hz, 1H), 3.86 (s, 3H), 3.84 (s, 3H). ^{13}**C NMR** (101 MHz, $CDCl_3$): δ 187.1 (d, *J* = 32.3 Hz, 1C), 164.2, 161.0 (d, *J* = 268.7 Hz, 1C), 160.2, 132.0 (d, *J* = 1.0 Hz, 1C), 119.4, 104.9, 102.0 (d, *J* = 17.2 Hz, 1C), 98.7, 55.7, 55.5. ^{19}**F NMR** (376 MHz, $CDCl_3$): δ -113.4. **HRMS** (ESI/Q-TOF) m/z: $[M+Na]^+$ Calcd for $C_{11}H_{11}FNaO_3^+$ 233.0584; found 233.0593.

1-(3,4-Dimethoxyphenyl)-2-fluoroprop-2-en-1-one (30): The product was prepared by following general procedure **A** and was obtained as white solid (0.083 g, 76%). The compound was purified by silica gel (230-400) flash chromatography using 15% EtOAc in hexane as eluent. **FT-IR** (ν cm^{-1}): 3135, 3008, 2942, 2696, 2611, 1658, 1589, 1511, 1265, 1141, 1018, 929, 879, 767, 671, 617. **^{1}H NMR** (400 MHz, $CDCl_3$): δ 7.61 (dt, *J* = 8.4, 1.8 Hz, 1H), 7.47 (d, *J* = 1.2 Hz, 1H), 6.91 (d, *J* = 8.4 Hz, 1H), 5.55 (dd, *J* = 45.8, 3.4 Hz, 1H), 5.41 (dd, *J* = 15.6, 3.2 Hz, 1H), 3.96 (s, 3H), 3.94 (s, 3H). **^{13}C NMR** (101 MHz, $CDCl_3$): δ 185.4 (d, *J* = 29.3 Hz, 1C), 160.3 (d, *J* = 270.7 Hz, 1C), 153.8, 149.0, 128.1, 124.6 (d, *J* = 7.0 Hz, 1C), 111.6 (d, *J* = 3.0 Hz, 1C), 110.0 (d, *J* = 1.0 Hz), 103.1 (d, *J* = 17.2 Hz), 56.1, 56.0. **^{19}F NMR** (376 MHz, $CDCl_3$): δ -109.1 (d, *J* = 11.3 Hz). **HRMS** (ESI/Q-TOF) m/z: [M+H]$^+$ Calcd for $C_{11}H_{12}FO_3^+$ 211.0765; found 211.0766.

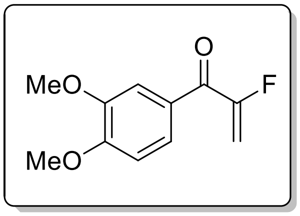

2-Fluoro-1-(3,4,5-trimethoxyphenyl) prop-2-en-1-one (31): The product was prepared by following general procedure **A** and was obtained as white solid (0.086 g, 80%). The compound was purified by silica gel (230-400) flash chromatography using 15% EtOAc in hexane as eluent. **FT-IR** (ν cm^{-1}): 2942, 2842, 2638, 1654, 1500, 1457, 1415, 1342, 1122, 1056, 995, 933, 840, 671. **^{1}H NMR** (400 MHz, $CDCl_3$): δ 7.17 (d, *J* = 1.2 Hz, 2H), 5.58 (dd, *J* = 45.4, 3.4 Hz, 1H), 5.47 (dd, *J* = 15.2, 3.2 Hz, 1H), 3.94 (s, 3H), 3.91 (s, 6H). **^{13}C NMR** (101 MHz, $CDCl_3$): δ 185.8 (d, *J* = 29.3 Hz, 1C), 161.4, 160.0 (d, *J* = 270.7 Hz, 1C), 153.0, 143.0, 130.3, 107.1 (d, *J* = 5.0 Hz), 103.8 (d, *J* = 16.2 Hz), 61.0, 56.3. **^{19}F NMR** (376 MHz, $CDCl_3$): δ -109.4. **HRMS** (ESI/Q-TOF) m/z: [M+H]$^+$ Calcd for $C_{12}H_{14}FO_4^+$ 241.0871; found 241.0886.

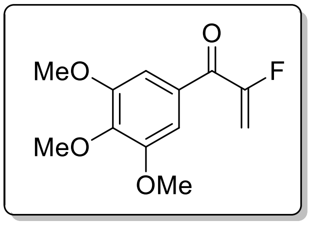

1-(Benzo[d][1,3] dioxol-5-yl)-2-fluoroprop-2-en-1-one (32): The product was prepared by following general procedure **A** and was obtained as colorless gummy liquid (0.092 g, 84%). The compound was purified by silica gel (230-400) flash chromatography using 12% EtOAc in hexane as eluent. **FT-IR** (ν cm^{-1}): 2912, 1658, 1608, 1492, 1442, 1358, 1246, 1099, 1034, 926, 879, 767. **^{1}H NMR** (400 MHz, $CDCl_3$): δ 7.55 (dt, *J* = 8.4, 1.6 Hz, 1H), 7.39 (t, *J* = 1.4 Hz,

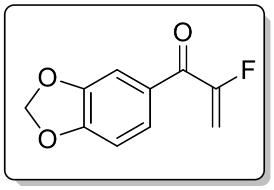

1H), 6.88 (d, *J* = 8.0 Hz, 1H), 6.07 (s, 2H), 5.54 (dd, *J* = 45.6, 3.2 Hz, 1H), 5.42 (dd, *J* = 15.2, 3.2 Hz, 1H). **^{13}C NMR** (101 MHz, $CDCl_3$): δ 185.1 (d, *J* = 29.3 Hz, 1C), 161.4 (d, *J* = 270.7 Hz, 1C), 152.3, 148.1, 129.7, 126.3 (d, *J* = 6.0 Hz), 109.3 (d, *J* = 4.0 Hz), 107.9, 103.3 (d, *J* = 16.2 Hz), 102.0. **^{19}F NMR** (376 MHz, $CDCl_3$): δ -109.3. **HRMS** (ESI/Q-TOF) m/z: $[M+H]^+$ Calcd for $C_{10}H_8FO_3^+$ 195.0452; found 195.0436.

2-Fluoro-1-(4-nitrophenyl) prop-2-en-1-one (33): The product was prepared by following general procedure **A** and was obtained as white solid (0.069 g, 63%). The compound was purified by silica gel (230-400) flash chromatography using 15% EtOAc in hexane as eluent. Pyridine was used as tertiary amine instead of DABCO. **FT-IR** (*ν* cm^{-1}): 3393, 3116, 3004, 2923, 2854, 2707, 1943, 1808, 1689, 1639, 1600, 1515, 1342, 1288, 1195, 1149, 1083, 852, 786, 713. **^{1}H NMR** (400 MHz, CD_3CN): δ 8.34 – 8.30 (m, 2H), 8.01 – 7.98 (m, 2H), 5.77 (dd, *J* = 15.6, 4.4 Hz, 1H), 5.62 (dd, *J* = 46.2, 4.2 Hz, 1H). **^{13}C NMR** (101 MHz, CD_3CN): δ 187.8 (d, *J* = 29.7 Hz, 1C), 160.4 (d, *J* = 264.3 Hz, 1C), 151.8, 142.4, 131.8 (d, *J* = 2.8 Hz, 1C), 125.1, 108.3 (d, *J* = 16.3 Hz, 1C). **^{19}F NMR** (376 MHz, CD_3CN): δ -114.9. **HRMS** (ESI/Q-TOF) m/z: $[M+H]^+$ Calcd for $C_9H_7FNO_3^+$ 196.0404; found 196.0417.

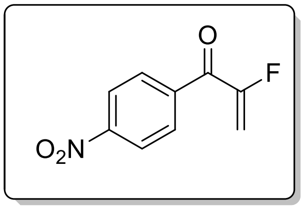

2-Fluoro-1-(3-nitrophenyl) prop-2-en-1-one (34): The product was prepared by following general procedure **A** and was obtained as white solid (0.075 g, 68%). The compound was purified by silica gel (230-400) flash chromatography using 15% EtOAc in hexane as eluent. Pyridine was used as tertiary amine instead of DABCO. The spectral data was identical to those reported in the literature.[4] **FT-IR** (*ν* cm^{-1}): 3023, 2873, 2402, 1712, 1681, 1619, 1535, 1349, 1214, 1099, 933. **^{1}H NMR** (400 MHz, CD_3CN): δ 8.36 (s, 1H), 8.24 (ddd, *J* = 8.0, 2.2, 1.0 Hz, 1H), 7.98 – 7.96 (m, 1H), 7.59 – 7.55 (m, 1H), 5.54 (dd, *J* = 15.6, 4.4 Hz, 1H), 5.44 (dd, *J* = 46.2, 4.2 Hz, 1H). **^{13}C NMR** (101 MHz, CD_3CN): δ 187.1 (d, *J* = 29.8 Hz, 1C), 160.4 (d, *J* = 264.7 Hz, 1C), 149.7, 138.4, 136.5 (d, *J* = 3.0 Hz, 1C), 131.7, 128.9, 125.4 (d, *J* = 3.3 Hz, 1C), 107.9 (d, *J* = 16.3 Hz, 1C). **^{19}F NMR** (376 MHz, CD_3CN): δ -114.5. **HRMS** (ESI/Q-TOF) m/z: $[M+H]^+$ Calcd for $C_9H_7FNO_3^+$ 196.0404; found 196.0415.

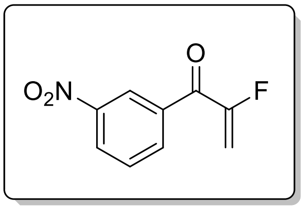

3-(2-Fluoroacryloyl) benzonitrile (35): The product was prepared by following general procedure **A** and was obtained as yellow liquid (0.075 g, 68%). The compound was purified by

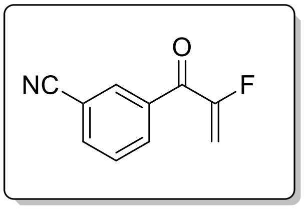

silica gel (230-400) flash chromatography using 15% EtOAc in hexane as eluent. Pyridine was used as tertiary amine instead of DABCO. The spectral data was identical to those reported in the literature.[4] **FT-IR** (v cm^{-1}): 3417, 3023, 2237, 1894, 1724, 1604, 1511, 1045, 925, 752. **^{1}H NMR** (400 MHz, $CDCl_3$): δ 8.17 – 8.16 (m, 1H), 8.11 (ddd, J = 7.8, 3.1, 1.3 Hz, 1H), 7.90 – 7.87 (m, 1H), 7.64 (td, J = 8.0, 0.5 Hz, 1H), 5.67 (dd, J = 44.8, 3.6 Hz, 1H), 5.55 (dd, J = 14.8, 3.6 Hz, 1H). **^{13}C NMR** (101 MHz, $CDCl_3$): δ 184.9 (d, J = 31.7 Hz, 1C), 159.4 (d, J = 270.1 Hz, 1C), 136.2, 133.3 (d, J = 4.6 Hz, 1C), 133.0 (d, J = 5.2 Hz), 129.6, 117.6, 113.2, 105.0 (d, J = 15.8 Hz). **^{19}F NMR** (376 MHz, $CDCl_3$): δ -111.5. **HRMS** (ESI/Q-TOF) m/z: $[M+H]^+$ Calcd for $C_{10}H_7FNO^+$ 176.0506; found 176.0512.

2-Fluoro-1-(4-fluorophenyl) prop-2-en-1-one (36): The product was prepared by following general procedure **A** and was obtained as colorless gummy liquid (0.088 g, 79%). The compound

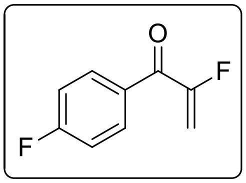

was purified by silica gel (230-400) flash chromatography using 7% EtOAc in hexane as eluent. The spectral data was identical to those reported in the literature.[4] **FT-IR** (v cm^{-1}): 3074, 1674, 1597, 1508, 1284, 1230, 1153, 929, 845, 771. **^{1}H NMR** (400 MHz, $CDCl_3$): δ 7.97 – 7.92 (m, 2H), 7.19 – 7.14 (m, 2H), 5.59 (dd, J = 44.0, 4.0 Hz, 1H), 5.48 (dd, J = 16.0, 4.0 Hz, 1H). **^{13}C NMR** (101 MHz, $CDCl_3$): δ 185.5 (d, J = 30.3 Hz, 1C), 165.9 (d, J = 256.5 Hz, 1C), 159.9 (d, J = 269.7 Hz, 1C), 132.2 (dd, J = 9.6, 4.5 Hz), 131.7 (d, J = 2.0 Hz), 115.8 (d, J = 22.2 Hz, 1C), 104.0 (d, J = 17.2 Hz). **^{19}F NMR** (376 MHz, $CDCl_3$) δ -103.9, -110.3. **HRMS** (ESI/Q-TOF) m/z: $[M+H]^+$ Calcd for $C_9H_7F_2O^+$ 169.0459; found 169.0452.

1-(4-Chlorophenyl)-2-fluoroprop-2-en-1-one (37): The reaction was performed 0.9 mmol scale. The product was prepared by following general procedure **A** and was obtained as colorless liquid

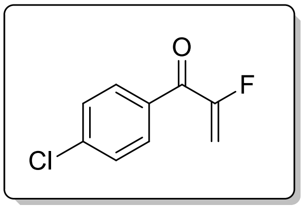

(0.113 g, 68%). The compound was purified by silica gel (230-400) flash chromatography using 6% EtOAc in hexane as eluent. **FT-IR** (v cm^{-1}): 3336, 3135, 3027, 2869, 1924, 1674, 1639, 1589, 1504, 1400, 1365, 1284, 1199, 1091, 979, 929, 840, 756, 624. **^{1}H NMR** (400 MHz,

$CDCl_3$): δ 7.85 – 7.83 (m, 2H), 7.48 – 7.45 (m, 2H), 5.60 (dd, *J* = 45.2, 3.6 Hz, 1H), 5.49 (dd, *J* = 15.0, 3.4 Hz, 1H). **^{13}C NMR** (101 MHz, $CDCl_3$): δ 185.9 (d, *J* = 30.5 Hz, 1C), 159.8 (d, *J* = 270.2 Hz, 1C), 140.0, 133.7, 130.9 (d, *J* = 4.5 Hz), 128.9, 104.2 (d, *J* = 16.5 Hz). **^{19}F NMR** (376 MHz, $CDCl_3$): δ -110.7. **HRMS** (ESI/Q-TOF) m/z: [M+Na] $^+$ Calcd for $C_9H_6ClFNaO^+$ 206.9983; found 206.9967.

1-(4-Bromophenyl)-2-fluoroprop-2-en-1-one (38): The reaction was performed 0.714 mmol scale. The product was prepared by following general procedure **A** and was obtained as colorless liquid (0.105 g, 70%). The compound was purified by silica gel (230-400) flash chromatography using 6% EtOAc in hexane as eluent. The spectral data was identical to those reported in the literature.[4] **FT-IR** (ν cm^{-1}): 3340, 3135, 3027, 2869, 2564, 1924, 1674, 1639, 1581, 1515, 1072, 979, 840, 756. **^{1}H NMR** (400 MHz, $CDCl_3$) δ 7.78 – 7.74 (m, 2H), 7.64 – 7.61 (m, 2H), 5.59 (dd, *J* = 44.0, 4.0 Hz, 1H), 5.49 (dd, *J* = 12.0, 4.0 Hz, 1H). **^{13}C NMR** (101 MHz, $CDCl_3$): δ 186.0 (d, *J* = 30.3 Hz, 1C), 159.7 (d, *J* = 269.7 Hz, 1C), 134.1, 131.9, 130.9 (d, *J* = 5.0 Hz), 128.6, 104.3 (d, *J* = 16.2 Hz). **^{19}F NMR** (376 MHz, $CDCl_3$): δ -110.8. **HRMS** (ESI/Q-TOF) m/z: [M+H] $^+$ Calcd for $C_9H_7BrFO^+$ 228.9659; found 228.9664.

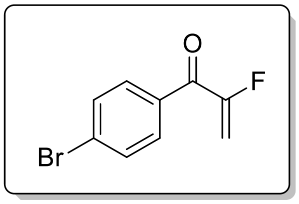

2-Fluoro-1-(4-iodophenyl) prop-2-en-1-one (39): The product was prepared by following general procedure **A** and was obtained as colorless liquid (0.081 g, 76%). The compound was purified by silica gel (230-400) flash chromatography using 8% EtOAc in hexane as eluent. **FT-IR** (ν cm^{-1}): 3020, 1697, 1612, 1215, 1149. **^{1}H NMR** (400 MHz, $CDCl_3$): δ 7.88 – 7.84 (m, 2H), 7.62 – 7.58 (m, 2H), 5.59 (dd, *J* = 44.0, 4.0 Hz, 1H), 5.50 (dd, *J* = 16.0, 4.0 Hz, 1H). **^{13}C NMR** (101 MHz, $CDCl_3$) δ 186.4 (d, *J* = 30.3 Hz, 1C), 159.7 (d, *J* = 269.7 Hz, 1C), 137.9, 134.7, 130.7 (d, *J* = 5.0 Hz, 1C), 104.4 (d, *J* = 16.2 Hz, 1C), 101.4. **^{19}F NMR** (376 MHz, $CDCl_3$) δ -110.9. **HRMS** (ESI/Q-TOF) m/z: [M+H] $^+$ Calcd for $C_9H_7FIO^+$ 276.9520; found 276.9521.

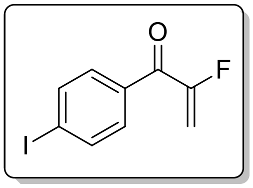

Procedure for the synthesis of 2-Fluoro-1-(1H-indol-3-yl) prop-2-en-1-one (40): To a stirring solution of 1-(1-tosyl-1H-indol-3-yl) prop-2-en-1-one **44** (0.100 g, 0.307 mmol) in a Falcon tube (Teflon coated) in dry acetonitrile (4 mL) at 25 °C was added **DABCO** (0.052 g, 0.461 mmol, 1.5

equiv.) under a nitrogen atmosphere. The mixture was stirred at the same temperature for 5 minutes under a nitrogen atmosphere. Then **2-Iodosyl-1,3-dimethylbenzene** (0.122 g, 0.492 mmol, 1.6 equiv.) and **Py•9HF** (0.138 mL, 1.54 mmol, 5 equiv.) were added to the reaction mixture sequentially. The resulting homogeneous mixture was stirred at the same temperature and monitored by TLC. On completion (14 hours), **triethyl amine** (0.428 mL, 3.073 mmol, 10 equiv.) was added to the reaction mixture and stirred for another 3 hr at the same temperature. Solvent was evaporated under reduced pressure and the crude residue was purified by silica gel (230-400) flash chromatography using (25% EtOAc in hexane as eluent) to afford the product **40** (0.049 g, 84%) as white solid. The spectral data was identical to those reported in the literature.[4]

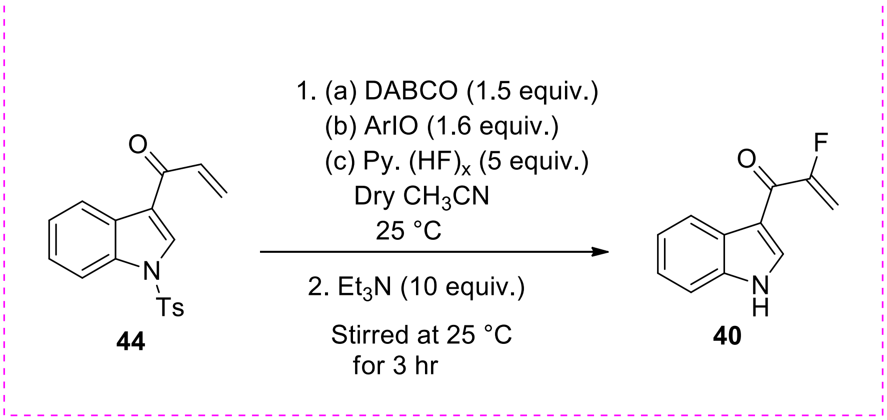

FT-IR (ν cm^{-1}): 3208, 2969, 2927, 1650, 1581, 1511, 1438, 1384, 1234, 1149, 948, 898.

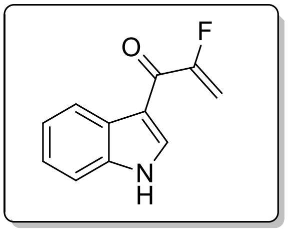

^{1}H NMR (400 MHz, DMSO-d_6): δ 12.24 (s, 1H), 8.33 (t, *J* = 3.4 Hz, 1H), 8.26 – 8.24 (m, 1H), 7.55 – 7.52 (m, 1H), 7.30 – 7.22 (m, 2H), 5.67 (dd, *J* = 48.8, 3.2 Hz, 1H), 5.43 (dd, *J* = 16.8, 3.6 Hz, 1H). **^{13}C NMR** (101 MHz, DMSO-d_6): δ 178.7 (d, *J* = 31.1 Hz, 1C), 160.8 (d, *J* = 269.9 Hz, 1C), 136.3, 135.8 (d, *J* = 17.1 Hz, 1C), 126.3, 123.5, 122.4, 121.5, 112.9 (d, *J* = 3.1 Hz, 1C), 112.4, 100.3 (d, *J* = 16.1 Hz, 1C). **^{19}F NMR** (376 MHz, DMSO-d_6): δ -111.9. **HRMS** (ESI/Q-TOF) m/z: [M+H]$^{+}$ Calcd for $C_{11}H_9FNO^{+}$ 190.0663; found 190.0650.

Synthesis of 2-fluoro-1-(naphthalen-2-yl) propan-1-ol (41): Compound **6** (0.135 g, 0.674 mmol) was dissolved in EtOAc (4 mL) and treated with 10% Pd/C. The reaction mixture was charged with hydrogen balloon and stirred at 25°C. After completion of reaction (3 hr), the mixture was filtered through a celite bed. The filtrate was evaporated to dryness. The crude product was

purified by column chromatography using 20% EtOAc in hexane mixture as an eluent to afford the product **41** (0.082 g, 60%) as colorless liquid (*dr* = 55:45).

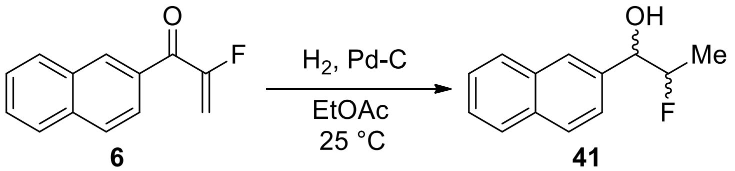

6 41

FT-IR (ν cm^{-1}): 3594, 3421, 3016, 2935, 2881, 2402, 2321, 1716, 1511, 1380, 1281, 1130, 1049.

^{1}H NMR (400 MHz, $CDCl_3$): δ 7.87 – 7.85 (m, 7.36H), 7.54 – 7.48 (m, 5.5H), 5.09 – 5.06 (m, 1H), 5.03 – 4.97 (m, 0.53H), 4.93 – 4.86 (m, 0.97H), 4.83 – 4.74 (m, 1.29H), 2.83 (br. s, 0.86H), 2.51 (br. s, 1H), 1.31 (dd, *J* = 19.4, 5.1 Hz, 3H), 1.27 – 1.21 (m, 2.65H). **^{13}C NMR** (101 MHz, $CDCl_3$): δ 136.5 (d, *J* = 4.5 Hz, 1C), 136.4 (d, *J* = 4.8 Hz, 1C), 133.3, 133.2, 133.1, 128.4, 128.2, 128.0, 127.9, 127.7, 127.6, 126.4, 126.3, 126.2, 126.1, 125.6, 125.5, 124.6, 124.21, 124.20, 94.3 (d, *J* = 83.3 Hz, 1C), 92.6 (d, *J* = 83.3 Hz, 1C), 77.8 (d, *J* = 20.8 Hz, 1C), 75.7 (d, *J* = 22.4 Hz, 1C), 17.1 (d, *J* = 22.3 Hz, 1C), 14.6 (d, *J* = 22.5 Hz, 1C. **^{19}F NMR** (376 MHz, $CDCl_3$): δ -179.9, -180.3. **HRMS** (ESI/Q-TOF) m/z: [M+H]$^+$ Calcd for $C_{13}H_{14}FO^+$ 205.1023; found 205.1025.

Synthesis of 2-fluoro-1-(naphthalen-2-yl) prop-2-en-1-ol (42):

To a stirring solution of **6** (0.137 g, 0.6842 mmol) in methanol (2 mL) at 0 °C, sodium borohydride (0.052 g, 1.368 mmol) was added and the mixture was stirred at the same temperature for 30 minutes. After 30 minutes the reaction mixture was quenched by aq. NH_4Cl solution and methanol was evaporated under reduced pressure. The residue was extracted with ethyl acetate, washed with brine and organic phase was dried over Na_2SO_4 and concentrated under reduced pressure. The crude product was purified by Flash column chromatography (silica gel, 20% ethyl acetate in hexane as eluent) to give **42** (0.116 g, 84 %) as colorless liquid.

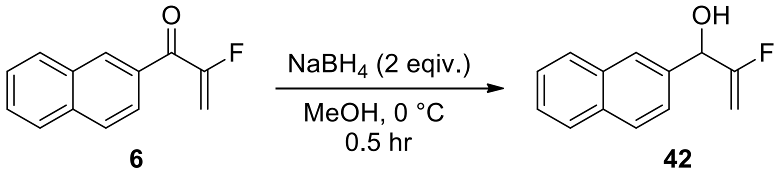

6 42

FT-IR (ν cm^{-1}): 3382, 3019, 2198, 1677, 1511, 1369, 1214, 1041. **^{1}H NMR** (400 MHz, $CDCl_3$): δ 7.89 – 7.84 (m, 4H), 7.55 – 7.51 (m, 3H), 5.35 (d, J = 9.2 Hz, 1H), 4.83 (dd, J = 17.2, 3.2 Hz, 1H), 4.72 (ddd, J = 48.8, 3.2, 1.0 Hz, 1H), 2.78 (br.s, 1H). **^{13}C NMR** (101 MHz, $CDCl_3$): δ 165.5 (d, J = 261.1 Hz, 1C), 136.4, 133.3, 133.1, 128.4, 128.1, 127.7, 126.3, 126.2, 126.0, 124.2, 91.4 (d, J = 17.1 Hz, 1C), 72.4. **^{19}F NMR** (376 MHz, $CDCl_3$): δ -107.9. **HRMS** (ESI/Q-TOF) m/z: $[M+H]^+$ Calcd for $C_{13}H_{12}FO^+$ 203.0867; found 203.0868.

HRMS Analysis of Crude Reaction Mixture Before Addition of Et_3N

The crude reaction mixture was monitored by HRMS (ESI/Q-TOF) (positive)

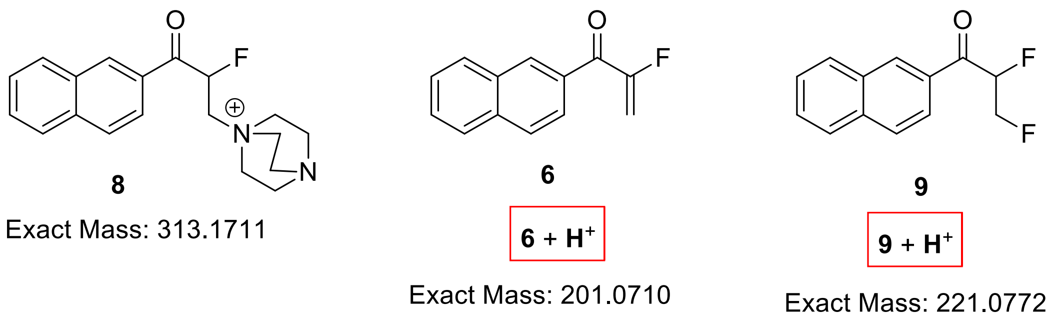

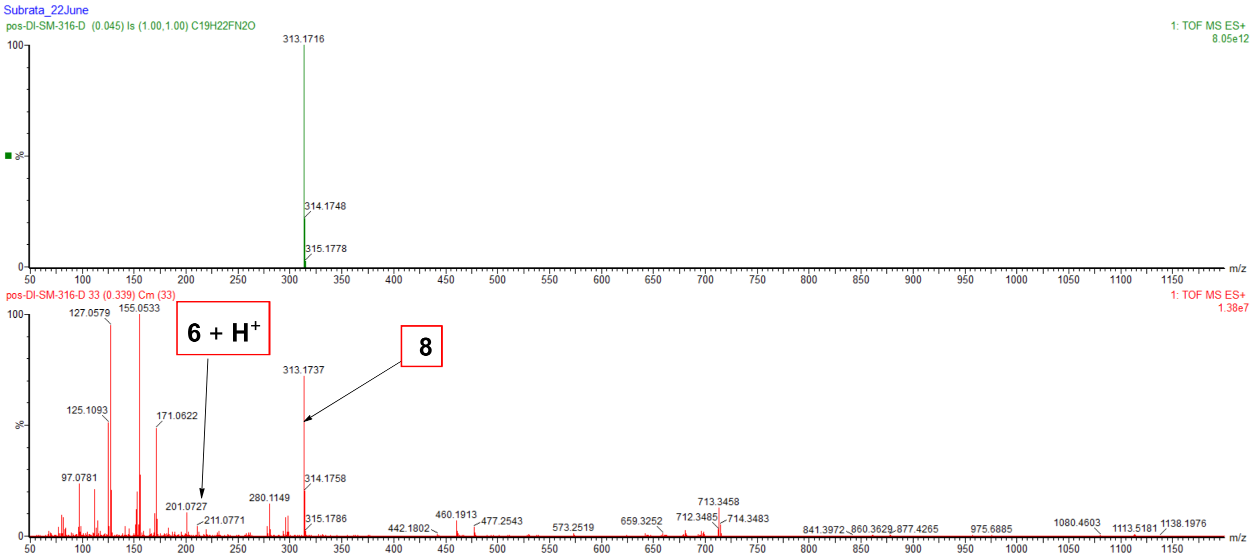

Figure 2: HRMS Chromatogram of crude reaction mixture.

These observations led us to propose the intermediate of the umpolung MBH fluorination reaction is **8** instead of **9**.

General procedure B for the synthesis of *α*, *β*-unsaturated carbonyl compounds: All *α*, *β*-unsaturated carbonyl compounds were prepared according to literature procedure[2] with little modification. To a mixture of a carbonyl compound **45** (1.0 mmol) and paraformaldehyde **46** (2.0 mmol) in dry THF (1.0 mL), diisopropylammonium trifluoroacetate salt **47** (1 mmol, 100 mol %) and trifluoroacetic acid (0.1 mmol, 10 mol %) were added under inert atmosphere. The reaction mixture was stirred at reflux (on an oil bath) for 2 h. The mixture became clear, then the reaction mixture was cooled down to room temperature and a second addition of

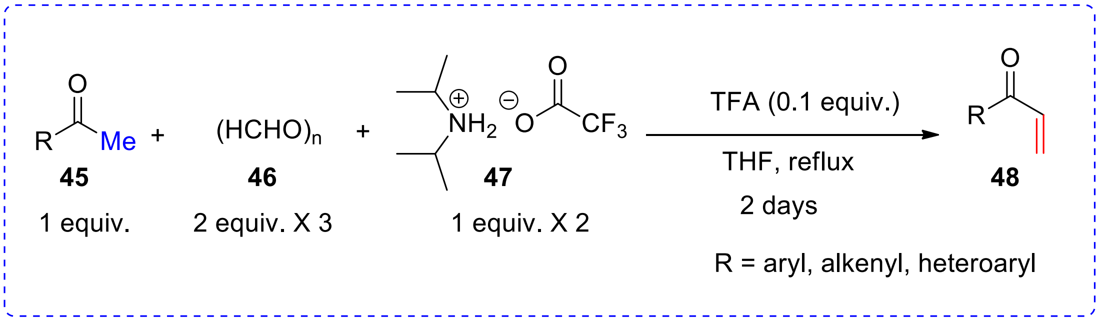

paraformaldehyde (2.0 mmol) was performed. Next, the reaction mixture was stirred at reflux (on an oil bath) for an additional 24 h under inert atmosphere. After 24 h the mixture was cooled down to room temperature. The third addition of paraformaldehyde (2.0 mmol) and second addition of diisopropylammonium trifluoroacetate salt (1 mmol, 100 mol %) were performed. The reaction mixture was then stirred at reflux (on an oil bath) for an additional 24 h. After that the mixture was cooled, solvent was removed under reduced pressure. The residue was dissolved in Et_2O, and the etheric solution was washed with 1 N HCl (100 mL), 1 N NaOH (100 mL), and brine, respectively. The organic phase was dried over Na_2SO_4 and concentrated under reduced pressure. The crude product was purified by Flash column chromatography (silica gel, 5-15% ethyl acetate in hexane as eluent) to give corresponding vinyl ketone **48**.

General procedure C for the synthesis of substituted *α, β*-unsaturated carbonyl compounds:

Substituted enones were prepared according to literature procedure.[3]

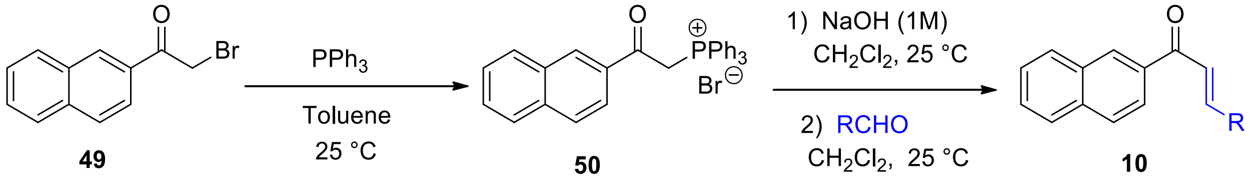

A mixture of 2-bromo-1-(naphthalen-2-yl) ethanone **49** (20.07 mmol) and triphenylphosphine (20.07 mmol) in toluene (40 mL) was stirred at 25 °C for 12 h. The formed precipitate was filtered and then dissolved in CH_2Cl_2 (70 mL) followed by NaOH (1.0 M, 100 mL) was added. After stirred for 2 h, the organic layer was separated, and water was extracted with CH_2Cl_2. The solvent was removed under vacuum and the solid was washed with PE and then filtrated to give stabilized ylide. To a solution of the appropriate stabilized ylide (9.29 mmol) in CH_2Cl_2 (10 mL) was added aldehyde (3 x 3 equiv. in 30 min interval) and the mixture was stirred at 25 °C for 3 h. The solvent was evaporated under reduced pressure and the crude mixture was purified by Flash chromatography to give **10**.

Yields and Characterization data for *α, β*-unsaturated carbonyl compounds

1-(Naphthalen-2-yl) prop-2-en-1-one (1): The reaction was performed 11.75 mmol scale. The product was prepared by following general procedure B and was obtained **1** as white solid (1.49 g, 70%). The compound was purified by silica gel (230-400) flash chromatography using 6% EtOAc in hexane as eluent. **^{1}H NMR** (400 MHz, $CDCl_3$): δ 8.47 (s, 1H), 8.04 (dd, *J* = 8.4, 1.6 Hz, 1H), 7.97 (dd, *J* = 8.0, 0.4 Hz, 1H), 7.93 – 7.88 (m, 2H), 7.63 – 7.54 (m, 2H), 7.33 (dd, *J* = 17.2, 10.8 Hz, 1H), 6.51 (dd, *J* = 17.0, 1.8 Hz, 1H), 5.98 (dd, *J* = 10.8, 1.6 Hz, 1H). **HRMS** (ESI/Q-TOF) m/z: $[M+H]^+$ Calcd for $C_{13}H_{11}O^+$ 183.0804; found 183.0804.

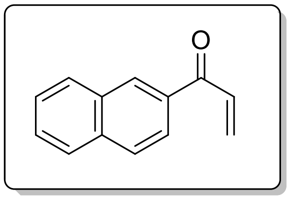

(*E*)-1-(Naphthalen-2-yl) but-2-en-1-one (10a): The reaction was performed 9.29 mmol scale. The product was prepared by following general procedure C and was obtained as white liquid (1.09 g, 60%). The compound was purified by silica gel (230-400) flash chromatography using 4% EtOAc in hexane as eluent. **^{1}H NMR** (400 MHz, $CDCl_3$): δ 8.45 (s, 1H), 8.04 (dd, *J* = 8.8, 1.6 Hz, 1H), 7.97 (d, *J* = 8.0 Hz, 1H), 7.89 (dd, *J* = 10.8, 8.4 Hz, 2H), 7.62 – 7.53 (m, 2H), 7.20 – 7.06 (m, 2H), 2.05 (dd, *J* = 6.4, 1.2 Hz, 3H). **HRMS** (ESI/Q-TOF) m/z: $[M+H]^+$ Calcd for $C_{14}H_{13}O^+$ 197.0961; found 197.0963.

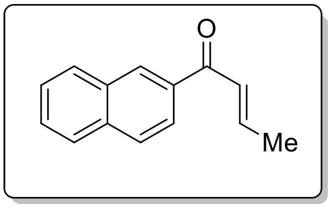

(*E*)-1-(Naphthalen-2-yl) pent-2-en-1-one (10b): The reaction was performed 9.29 mmol scale. The product was prepared by following general procedure C and was obtained as white liquid (1.25 g, 64%). The compound was purified by silica gel (230-400) flash chromatography using 3% EtOAc in hexane as eluent. **^{1}H NMR** (400 MHz, $CDCl_3$): δ 8.45 (s, 1H), 8.04 (dd, *J* = 8.6, 1.8 Hz, 1H), 7.98 (d, *J* = 8.0 Hz, 1H), 7.93 – 7.89 (m, 2H), 7.63 – 7.54 (m, 2H), 7.20 (dt, *J* = 15.2, 6.3 Hz, 1H), 7.05 (dt, *J* = 15.6, 1.5 Hz, 1H), 2.44 – 2.37 (m, 2H), 1.19 (t, *J* = 7.4 Hz, 3H). **HRMS** (ESI/Q-TOF) m/z: $[M+H]^+$ Calcd for $C_{15}H_{15}O$ 211.1117; found 211.1115.

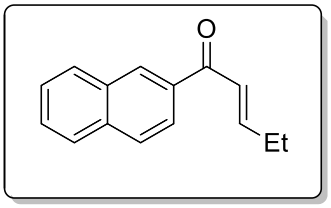

1-Phenylprop-2-en-1-one (48a): The reaction was performed 16.646 mmol scale. The product was prepared by following general procedure B and was obtained as colorless liquid (1.605 g, 73%). The compound was purified by silica gel (230-400) flash chromatography using 5% EtOAc in hexane as eluent. **^{1}H NMR** (400 MHz, $CDCl_3$): δ 7.97 – 7.94 (m, 2H), 7.60 – 7.55 (m, 1H), 7.50 – 7.46 (m, 2H), 7.16 (dd, *J* = 17.2, 10.4 Hz, 1H), 6.44 (dd, *J* = 17.0, 1.8 Hz, 1H), 5.93 (dd, *J* = 10.6, 1.8 Hz, 1H). **HRMS** (ESI/Q-TOF) m/z: $[M+H]^+$ Calcd for $C_9H_9O^+$ 133.0648; found 133.0642.

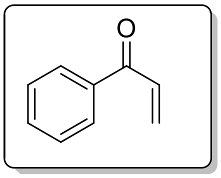

(*E*)-1-phenylpenta-1,4-dien-3-one (48b): The reaction was performed 13.681 mmol scale. The product was prepared by following general procedure B and was obtained as colorless liquid (1.60 g, 74%). The compound was purified by silica gel (230-400) flash chromatography using 8% EtOAc in hexane as eluent. **^{1}H NMR** (400 MHz, $CDCl_3$): δ 7.69 (d, *J* = 16.0 Hz, 1H), 7.61 – 7.59 (m, 2H), 7.43 – 7.40 (m, 3H), 7.02 (d, *J* = 16.0 Hz, 1H), 6.73 (dd, *J* = 17.4, 10.6 Hz, 1H), 6.39 (dd, *J* = 17.4, 1.4 Hz, 1H), 5.90 (dd, *J* = 10.8, 1.2 Hz, 1H). **HRMS** (ESI/Q-TOF) m/z: $[M+H]^+$ Calcd for $C_{11}H_{11}O^+$ 159.0804; found 159.0806.

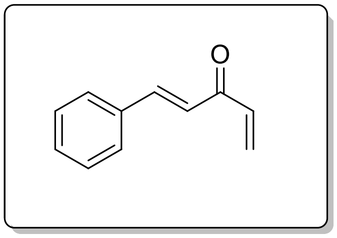

(*E*)-1-(*p*-tolyl) penta-1,4-dien-3-one (48c): The reaction was performed 12.483 mmol scale. The product was prepared by following general procedure B and was obtained as colorless liquid (1.55 g, 72%). The compound was purified by silica gel (230-400) flash chromatography using 8% EtOAc in hexane as eluent. **^{1}H NMR** (400 MHz, $CDCl_3$): δ 7.67 (d, *J* = 16.0 Hz, 1H), 7.49 (d, *J* = 8.0 Hz, 2H), 7.22 (d, *J* = 8.0 Hz, 2H), 6.98 (d, *J* = 16.0 Hz, 1H), 6.72 (dd, *J* = 17.4, 10.6 Hz, 1H), 6.38 (dd, *J* = 17.4, 1.4 Hz, 1H), 5.87 (dd, *J* = 10.6, 1.0 Hz, 1H), 2.39 (s, 3H). **HRMS** (ESI/Q-TOF) m/z: $[M+H]^+$ Calcd for $C_{12}H_{13}O^+$ 173.0961; found 173.0964.

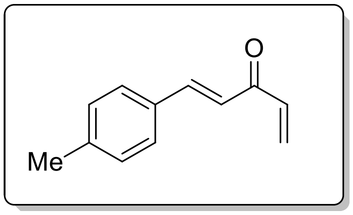

1-(Cyclohex-1-en-1-yl) prop-2-en-1-one (48d): The reaction was performed 16.1056 mmol scale. The product was prepared by following general procedure B and was obtained as colorless liquid (1.43 g, 65%). The compound was purified by silica gel (230-400) flash chromatography using 4% EtOAc in hexane as eluent. **^{1}H NMR** (400 MHz, $CDCl_3$): δ 6.96 – 6.89 (m, 2H), 6.24 (dd, *J* = 17.2, 2.0 Hz, 1H), 5.69 (dd, *J* = 10.8, 2.0 Hz, 1H), 2.32 – 2.26 (m, 4H), 1.70 – 1.60 (m, 4H). **HRMS** (ESI/Q-TOF) m/z: $[M+H]^+$ Calcd for $C_9H_{13}O^+$ 137.0961; found 137.0962.

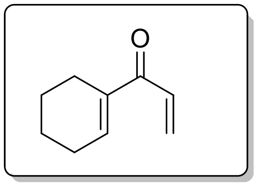

1-(*p*-tolyl) prop-2-en-1-one (48e): The reaction was performed 14.905 mmol scale. The product was prepared by following general procedure B and was obtained as colorless liquid (1.44 g, 66%).

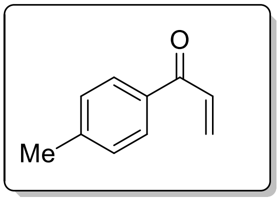

The compound was purified by silica gel (230-400) flash chromatography using 7% EtOAc in hexane as eluent. **^{1}H NMR** (400 MHz, $CDCl_3$): δ 7.89 – 7.86 (m, 2H), 7.29 (dd, *J* = 8.6, 0.6 Hz, 2H), 7.17 (dd, *J* = 17.0, 10.6 Hz, 1H), 6.44 (dd, *J* = 17.0, 1.8 Hz, 1H), 5.90 (dd, *J* = 10.6, 1.8 Hz, 1H), 2.43 (s, 3H). **HRMS** (ESI/Q-TOF) m/z: [M+H] $^+$ Calcd for $C_{10}H_{11}O^+$ 147.0804; found 147.0805.

1-(*o*-tolyl) prop-2-en-1-one (48f): The reaction was performed 14.905 mmol scale. The product

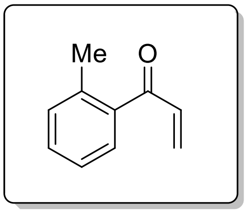

was prepared by following general procedure B and was obtained as colorless liquid (1.26 g, 58%). The compound was purified by silica gel (230-400) flash chromatography using 6% EtOAc in hexane as eluent. **^{1}H NMR** (400 MHz, $CDCl_3$): δ 7.87 (d, *J* = 8.0 Hz, 2H), 7.28 (d, *J* = 8.0 Hz, 2H), 7.16 (dd, *J* = 17.2, 10.4 Hz, 1H), 6.43 (dd, *J* = 17.2, 1.6 Hz, 1H), 5.90 (dd, *J* = 10.4, 1.6 Hz, 1H). **HRMS** (ESI/Q-TOF) m/z: [M+H] $^+$ Calcd for $C_{10}H_{11}O^+$ 147.0804; found 147.0806.

1-(*m*-Tolyl)prop-2-en-1-one (48g): The reaction was performed 14.905 mmol scale. The product was prepared by following general procedure B and was obtained as colorless liquid (1.48 g, 68%). The compound was purified by silica gel (230-400) flash chromatography using 6% EtOAc in

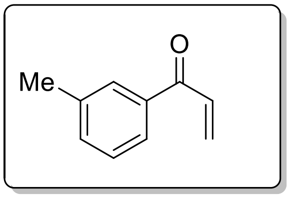

hexane as eluent. **^{1}H NMR** (400 MHz, $CDCl_3$): δ 7.78 – 7.74 (m, 2H), 7.41 – 7.36 (m, 2H), 7.16 (dd, *J* = 17.2, 10.4 Hz, 1H), 6.44 (dd, *J* = 17.2, 1.6 Hz, 1H), 5.93 (dd, *J* = 10.6, 1.8 Hz, 1H), 2.43 (s, 3H). **HRMS** (ESI/Q-TOF) m/z: [M+H] $^+$ Calcd for $C_{10}H_{11}O^+$ 147.0804; found 147.0808.

1-(3, 4-Dimethylphenyl) prop-2-en-1-one (48h): The reaction was performed 13.495 mmol

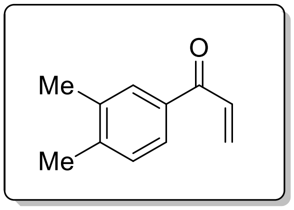

scale. The product was prepared by following general procedure B and was obtained as colorless liquid (1.51 g, 70%). The compound was purified by silica gel (230-400) flash chromatography using 6% EtOAc in hexane as eluent. **^{1}H NMR** (400 MHz, $CDCl_3$): δ 7.75 –

7.69 (m, 2H), 7.25 – 7.14 (m, 2H), 6.43 (dd, *J* = 17.0, 1.8 Hz, 1H), 5.89 (dd, *J* = 10.6, 1.8 Hz, 1H), 2.34 (s, 6H). **HRMS** (ESI/Q-TOF) m/z: [M+H] $^+$ Calcd for $C_{11}H_{13}O^+$ 161.0961, found 161.0948.

1-(4-(Tert-butyl) phenyl) prop-2-en-1-one (48i): The reaction was performed 5.673 mmol scale. The product was prepared by following general procedure B and was obtained as white gummy liquid (0.769 g, 72%). The compound was purified by silica gel (230-400) flash chromatography

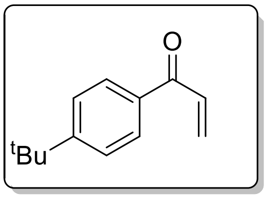

using 8% EtOAc in hexane as eluent. **^{1}H NMR** (400 MHz, $CDCl_3$): δ 7.92 – 7.89 (m, 2H), 7.52 – 7.48 (m, 2H), 7.21 – 7.14 (m, 1H), 6.44 (dd, *J* = 17.2, 2.0 Hz, 1H), 5.90 (dd, *J* = 10.6, 1.8 Hz, 1H), 1.35 (s, 9H). **HRMS** (ESI/Q-TOF) m/z: [M+H] $^+$ Calcd for $C_{13}H_{17}O^+$ 189.1274, found 189.1269.

1-([1,1'-Biphenyl]-4-yl) prop-2-en-1-one (48j): The reaction was performed 10.1916 mmol scale. The product was prepared by following general procedure B and was obtained as white solid

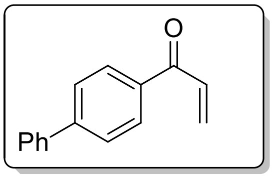

(1.46 g, 69%). The compound was purified by silica gel (230-400) flash chromatography using 7% EtOAc in hexane as eluent. **^{1}H NMR** (400 MHz, $CDCl_3$): δ 8.06 – 8.03 (m, 2H), 7.73 – 7.70 (m, 2H), 7.65 – 7.63 (m, 2H), 7.50 – 7.46 (m, 2H), 7.43 – 7.39 (m, 1H), 7.22 (dd, *J* = 17.0, 10.6 Hz, 1H), 6.48 (dd, *J* = 17.0, 1.8 Hz, 1H), 5.95 (dd, *J* = 10.6, 1.8 Hz, 1H). **HRMS** (ESI/Q-TOF) m/z: [M+H] $^+$ Calcd for $C_{15}H_{13}O^+$ 209.0961; found 209.0959.

1-(4-Methoxyphenyl) prop-2-en-1-one (48k): The reaction was performed 13.318 mmol scale. The product was prepared by following general procedure B and was obtained as colorless liquid

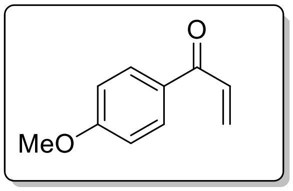

(1.511 g, 70%). The compound was purified by silica gel (230-400) flash chromatography using 15% EtOAc in hexane as eluent. **^{1}H NMR** (400 MHz, $CDCl_3$): δ 7.99 – 7.95 (m, 2H), 7.17 (dd, *J* = 17.0, 10.6 Hz, 1H), 6.98 – 6.94 (m, 2H), 6.42 (dd, *J* = 17.2, 1.6 Hz, 1H), 5.87 (dd, *J* = 10.6, 1.8 Hz, 1H), 3.88 (s, 3H). **HRMS** (ESI/Q-TOF) m/z: [M+H] $^+$ Calcd for $C_{10}H_{11}O_2^+$ 163.0754; found 163.0753.

1-(2-Methoxyphenyl) prop-2-en-1-one (48l): The reaction was performed 13.318 mmol scale. The product was prepared by following general procedure B and was obtained as colorless liquid

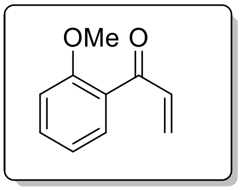

(1.403 g, 65%). The compound was purified by silica gel (230-400) flash chromatography using 12% EtOAc in hexane as eluent. **^{1}H NMR** (400 MHz, $CDCl_3$): δ 7.57 (dd, *J* = 7.6, 1.6 Hz, 1H), 7.46 (ddd, *J* = 8.4, 7.4, 1.8 Hz, 1H), 7.03 – 6.96 (m, 3H), 6.27 (dd, *J* = 17.2, 1.6 Hz, 1H), 5.81 (dd, *J* = 10.4, 1.6 Hz, 1H), 3.88 (s, 3H). **HRMS** (ESI/Q-TOF) m/z: [M+H] $^+$ Calcd for $C_{10}H_{11}O_2^+$ 163.0754; found 163.0752.

1-(3-Methoxyphenyl)prop-2-en-1-one (48m): The reaction was performed 13.318 mmol scale. The product was prepared by following general procedure B and was obtained as colorless liquid

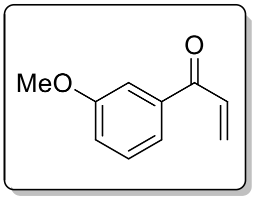

(1.49 g, 69%). The compound was purified by silica gel (230-400) flash chromatography using 12% EtOAc in hexane as eluent. **^{1}H NMR** (400 MHz, $CDCl_3$): δ 7.53 – 7.47 (m, 2H), 7.38 (t, *J* = 7.8 Hz, 1H), 7.17 – 7.11 (m, 2H), 6.44 (dd, *J* = 17.2, 1.6 Hz, 1H), 5.92 (dd, *J* = 10.6, 1.8 Hz, 1H), 3.86 (s, 3H). **HRMS** (ESI/Q-TOF) m/z: [M+H] $^+$ Calcd for $C_{10}H_{11}O_2^+$ 163.0754; found 163.0756.

1-(4-Phenoxyphenyl) prop-2-en-1-one (48n): The reaction was performed 9.423 mmol scale. The product was prepared by following general procedure B and was obtained as colorless liquid (1.27 g, 60%). The compound was purified by silica gel (230-400) flash chromatography using

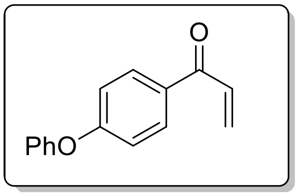

6% EtOAc in hexane as eluent. **^{1}H NMR** (400 MHz, $CDCl_3$): δ 7.98 – 7.94 (m, 2H), 7.43 – 7.38 (m, 2H), 7.23 – 7.12 (m, 2H), 7.09 – 7.06 (m, 2H), 7.04 – 7.00 (m, 2H), 6.44 (dd, *J* = 17.0, 1.8 Hz, 1H), 5.90 (dd, *J* = 10.6, 1.8 Hz, 1H). **HRMS** (ESI/Q-TOF) m/z: [M+H] $^+$ Calcd for $C_{15}H_{13}O_2^+$ 225.0910; found 225.0910.

1-(2,4-Dimethoxyphenyl) prop-2-en-1-one (48o): The reaction was performed 11.098 mmol

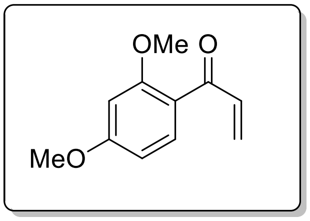

scale. The product was prepared by following general procedure B and was obtained as colorless liquid (1.45 g, 68%). The compound was purified by silica gel (230-400) flash chromatography using 15% EtOAc in hexane as eluent. **^{1}H NMR** (400 MHz, $CDCl_3$): δ 7.72 (d, *J* = 8.8 Hz,

1H), 7.14 (dd, J = 17.2, 10.4 Hz, 1H), 6.55 (dd, J = 8.6, 2.2 Hz, 1H), 6.47 (d, J = 2.4 Hz, 1H), 6.32 (dd, J = 17.2, 2.0 Hz, 1H), 5.71 (dd, J = 10.4, 2.0 Hz, 1H), 3.88 (s, 3H), 3.86 (s, 3H). **HRMS** (ESI/Q-TOF) m/z: [M+H] $^+$ Calcd for $C_{11}H_{13}O_3^+$ 193.0859; found 193.0862.

1-(3,4-Dimethoxyphenyl) prop-2-en-1-one (48p): The reaction was performed 11.098 mmol scale. The product was prepared by following general procedure B and was obtained as colorless

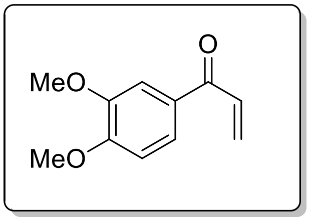

liquid (1.30 g, 61%). The compound was purified by silica gel (230-400) flash chromatography using 18% EtOAc in hexane as eluent. **^{1}H NMR** (400 MHz, $CDCl_3$): δ 7.61 – 7.56 (m, 2H), 7.19 (dd, J = 17.0, 10.6 Hz, 1H), 6.90 (d, J = 8.4 Hz, 1H), 6.43 (dd, J = 17.2, 1.6 Hz, 1H), 5.87 (dd, J = 10.4, 2.0 Hz, 1H), 3.95 (s, 3H), 3.94 (s, 3H). **HRMS** (ESI/Q-TOF) m/z: [M+H] $^+$ Calcd for $C_{11}H_{13}O_3^+$ 193.0859; found 193.0861.

1-(3,4,5-Trimethoxyphenyl) prop-2-en-1-one (48q): The reaction was performed 9.513 mmol scale. The product was prepared by following general procedure B and was obtained as white solid

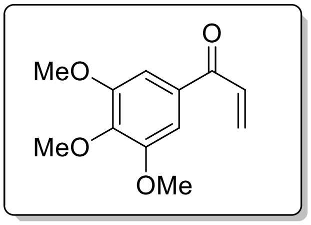

(1.48 g, 70%). The compound was purified by silica gel (230-400) flash chromatography using 15% EtOAc in hexane as eluent. **^{1}H NMR** (400 MHz, $CDCl_3$): δ 7.23 (s, 2H), 7.16 (dd, J = 17.0, 10.6 Hz, 1H), 6.45 (dd, J = 17.0, 1.8 Hz, 1H), 5.93 (dd, J = 10.6, 1.8 Hz, 1H), 3.94 (s, 9H). **HRMS** (ESI/Q-TOF) m/z: [M+H] $^+$ Calcd for $C_{12}H_{15}O_4^+$ 223.0965; found 223.0970.

1-(Benzo[d][1, 3] dioxol-5-yl)prop-2-en-1-one (48r): The reaction was performed 12.183 mmol scale. The product was prepared by following general procedure B and was obtained as white solid (1.65 g, 77%). The compound was purified by silica gel (230-400) flash chromatography using

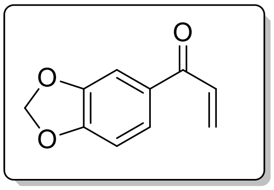

12% EtOAc in hexane as eluent. **^{1}H NMR** (400 MHz, $CDCl_3$): δ 7.56 (dd, J = 8.2, 1.7 Hz, 1H), 7.46 (d, J = 1.7 Hz, 1H), 7.12 (dd, J = 17.0, 10.5 Hz, 1H), 6.87 (d, J = 8.1 Hz, 1H), 6.41 (dd, J = 17.0, 1.8 Hz, 1H), 6.05 (s, 2H), 5.87 (dd, J = 10.5, 1.8 Hz, 1H). **HRMS** (ESI/Q-TOF) m/z: [M+H] $^+$ Calcd for $C_{10}H_9O_3^+$ 177.0546, found 177.0545.

1-(4-Nitrophenyl) prop-2-en-1-one (48s): The reaction was performed 18.165 mmol scale. The product was prepared by following general procedure B and was obtained as white solid (0.837 g, 26%). The compound was purified by silica gel (230-400) flash chromatography using 15% EtOAc in hexane as eluent. **^{1}H NMR** (400 MHz, $CDCl_3$): δ 8.34 (d, *J* = 8.8 Hz, 2H), 8.08 (d, *J* = 8.8 Hz, 2H), 7.13 (dd, *J* = 17.2, 10.4 Hz, 1H), 6.49 (dd, *J* = 17.2, 1.2 Hz, 1H), 6.09 – 6.06 (m, 1H). **HRMS** (ESI/Q-TOF) m/z: [M+H] $^+$ Calcd for $C_9H_8NO_3^+$ 178.0499; found 178.0498.

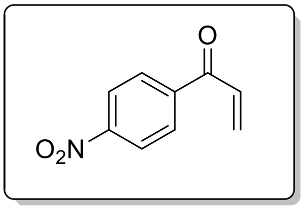

1-(3-Nitrophenyl) prop-2-en-1-one (48t): The reaction was performed 18.165 mmol scale. The product was prepared by following general procedure B and was obtained as white solid (0.901 g, 28%). The compound was purified by silica gel (230-400) flash chromatography using 15% EtOAc in hexane as eluent. **^{1}H NMR** (400 MHz, $CDCl_3$): δ 8.76 (t, *J* = 1.8 Hz, 1H), 8.44 (ddd, *J* = 8.4, 2.2, 1.2 Hz, 1H), 8.29 – 8.26 (m, 1H), 7.71 (t, *J* = 8.0 Hz, 1H), 7.18 (dd, *J* = 17.0, 10.6 Hz, 1H), 6.53 (dd, *J* = 17.0, 1.4 Hz, 1H), 6.07 (dd, *J* = 10.6, 1.4 Hz, 1H). **HRMS** (ESI/Q-TOF) m/z: [M+H] $^+$ Calcd for $C_9H_8NO_3^+$ 178.0499; found 178.0497.

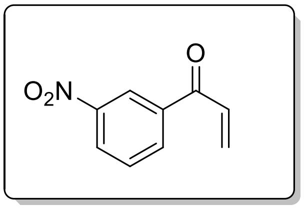

3-Acryloylbenzonitrile (48u): The reaction was performed 13.778 mmol scale. The product was prepared by following general procedure B and was obtained as colorless liquid (0.779 g, 36%). The compound was purified by silica gel (230-400) flash chromatography using 15% EtOAc in hexane as eluent. **^{1}H NMR** (400 MHz, $CDCl_3$): δ 8.21 (d, *J* = 1.6 Hz, 1H), 8.17 – 8.15 (m, 1H), 7.87 – 7.84 (m, 1H), 7.63 (t, *J* = 7.8 Hz, 1H), 7.10 (dd, *J* = 17.2, 10.4 Hz, 1H), 6.49 (dd, *J* = 17.0, 1.4 Hz, 1H), 6.05 (dd, *J* = 10.6, 1.4 Hz, 1H). **HRMS** (ESI/Q-TOF) m/z: [M+H] $^+$ Calcd for $C_{10}H_8NO^+$ 158.0600; found 158.0598.

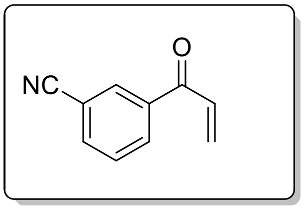

1-(4-Fluorophenyl) prop-2-en-1-one (48v): The reaction was performed 14.478 mmol scale. The product was prepared by following general procedure B and was obtained as colorless liquid (1.565 g, 72%). The compound was purified by silica gel (230-400) flash chromatography using 7% EtOAc in hexane as eluent. **^{1}H NMR** (400 MHz, $CDCl_3$): δ 8.01 – 7.96 (m, 2H), 7.18 – 7.10 (m, 3H), 6.44 (dd, *J* =

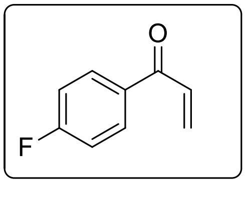

17.2, 1.6 Hz, 1H), 5.94 (dd, J = 10.8, 1.6 Hz, 1H). **HRMS** (ESI/Q-TOF) m/z: $[M+H]^+$ Calcd for $C_9H_8FO^+$ 151.0554, found 151.0556.

1-(4-Chlorophenyl) prop-2-en-1-one (48w): The reaction was performed 12.937 mmol scale. The product was prepared by following general procedure B and was obtained as colorless liquid

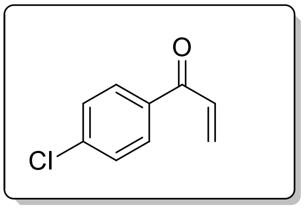

(1.38 g, 64%). The compound was purified by silica gel (230-400) flash chromatography using 6% EtOAc in hexane as eluent. **^{1}H NMR** (400 MHz, $CDCl_3$): δ 7.92 – 7.88 (m, 2H), 7.48– 7.45 (m, 2H), 7.12 (dd, J = 17.2, 10.4 Hz, 1H), 6.45 (dd, J = 17.2, 1.6 Hz, 1H), 5.96 (dd, J = 10.8, 1.6 Hz, 1H). **HRMS** (ESI/Q-TOF) m/z: $[M+H]^+$ Calcd for $C_9H_8ClO^+$ 167.0258; found 167.0257.

1-(4-Bromophenyl) prop-2-en-1-one (48x): The reaction was performed 10.048 mmol scale. The product was prepared by following general procedure B and was obtained as colorless liquid (1.31

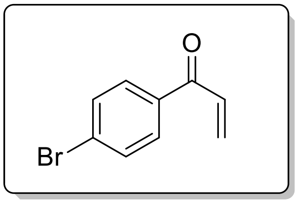

g, 62%). The compound was purified by silica gel (230-400) flash chromatography using 6% EtOAc in hexane as eluent. **^{1}H NMR** (400 MHz, $CDCl_3$): δ 7.83 – 7.80 (m, 2H), 7.65 – 7.61(m, 2H), 7.11 (dd, J = 17.2, 10.4 Hz, 1H), 6.45 (dd, J = 17.2, 1.6 Hz, 1H), 5.96 (dd, J = 10.4, 1.6 Hz, 1H). **HRMS** (ESI/Q-TOF) m/z: $[M-H]^-$ Calcd for C_9H_6BrO 208.9602; found 208.9601. (Note: HRMS was recorded in negative mode)

1-(4-Iodophenyl) prop-2-en-1-one (48y): The reaction was performed 8.128 mmol scale. The product was prepared by following general procedure B and was obtained as white solid (1.363 g,

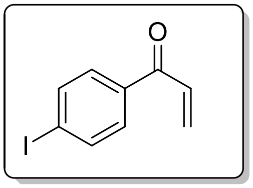

65%). The compound was purified by silica gel (230-400) flash chromatography using 8% EtOAc in hexane as eluent. **^{1}H NMR** (400 MHz, $CDCl_3$): δ 7.85 – 7.82 (m, 2H), 7.66 – 7.63 (m, 2H), 7.09 (dd, J = 17.2, 10.4 Hz, 1H), 6.43 (dd, J = 17.2, 1.6 Hz, 1H), 5.94 (dd, J = 10.8, 1.6 Hz, 1H). **HRMS** (ESI/Q-TOF) m/z: $[M+H]^+$ Calcd for $C_9H_8IO^+$ 258.9614; found 258.9608.

1-(1-Tosyl-1H-indol-3-yl) prop-2-en-1-one (44): The reaction was performed 6.38 mmol scale. The product was prepared by following general procedure B and was obtained as white solid (1.04 g, 50%). The compound was purified by silica gel (230-400) flash chromatography using 25% EtOAc in hexane as eluent. **^{1}H NMR** (400 MHz, $CDCl_3$): δ 8.40 – 8.38 (m, 1H), 8.27 (s, 1H), 7.96 – 7.93 (m, 1H), 7.85 – 7.83 (m, 2H), 7.41 – 7.34 (m, 2H), 7.28 (dd, *J* = 8.4, 0.4 Hz, 2H), 7.05 (dd, *J* = 17.2, 10.4 Hz, 1H), 6.49 (dd, *J* = 17.2, 1.6 Hz, 1H), 5.88 (dd, *J* = 10.4, 1.6 Hz, 1H), 2.36 (s, 3H). **HRMS** (ESI/Q-TOF) m/z: $[M+H]^+$ Calcd for $C_{18}H_{16}NO_3S^+$ 326.0845; found 326.0849.

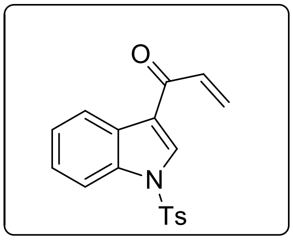

Synthesis of 2-Iodosyl-1,3-dimethylbenzene (11)

Compound **11** is commercially available but may also be prepared following the first two steps in Murphy's procedure for $TolIF_2$.[1] In our laboratory the reaction was performed at a 64.599 mmol scale and the product was obtained as white solid (10 g, 62%). The ^{1}H NMR of prepared **11** was identical to a commercial sample.

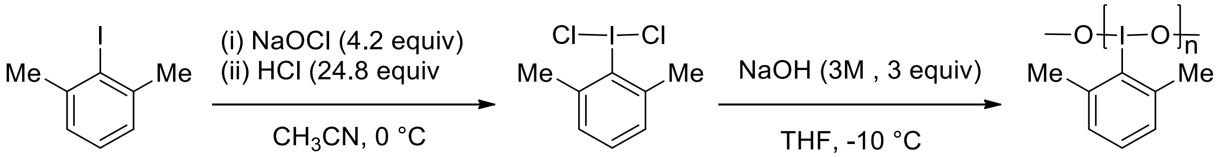

51 **52** **11**

^{1}H NMR (400 MHz, DMSO-*d6*): δ 7.19 – 7.12 (m, 3H), 2.40 (s, 6H). **^{13}C NMR** (101 MHz, DMSO-*d6*): δ 141.4, 127.8, 127.2, 108.3, 29.2.

References:

1. Tao, J.; Murphy, G. K.; A Practical, Large-Scale Synthesis of *p*-(Difluoroiodo)toluene (*p*-$TolIF_2$). *Synthesis* **2019**, *51*, 3055–3059.

2. (a) Li, Y. –Q.; Wang, H. –J.; Huang, Z. –Z.; Morita–Baylis–Hillman Reaction of *α*, *β*-Unsaturated Ketones with Allylic Acetates by the Combination of Transition-Metal Catalysis and Organomediation. *J. Org. Chem.* **2016**, *81*, 4429-4433. (b) Kong, L.; Hain, X.; Jiao, P.; Catalytic asymmetric Diels–Alder reactions involving aryl vinyl ketones. *Chem. Commun.* **2014**, *50*, 14113-14116.

(c) Guo, S. –H.; Xing, S. –Z.; Mao, S.; Gao, Y. –R.; Chen, W. –L.; Wang, Y. –Q.; Oxa-Michael addition promoted by the aqueous sodium carbonate. *Tetrahedron Lett.* **2014**, *55*, 6718-6720.

3. Chen, Z.; Hu, F.; Huang, S.; Zhao, Z.; Mao, H.; Qin, W.; Organocatalytic Enantioselective Selenosulfonylation of a C−C Double Bond to Form Two Stereogenic Centers in an Aqueous Medium. *J.Org. Chem.* **2019**, *84*, 8100−8111.

4. Song, X.; Chang, J.; Zhu, D.; Li, J.; Xu, C.; Liu, Q.; Wang, M.; Catalytic Domino Reaction of Ketones/Aldehydes with Me_3SiCF_2Br for the Synthesis of α-Fluoroenones/α-Fluoroenals. *Org. Lett*. **2015**, *17*, 1712−1715.

Copies of NMR spectra

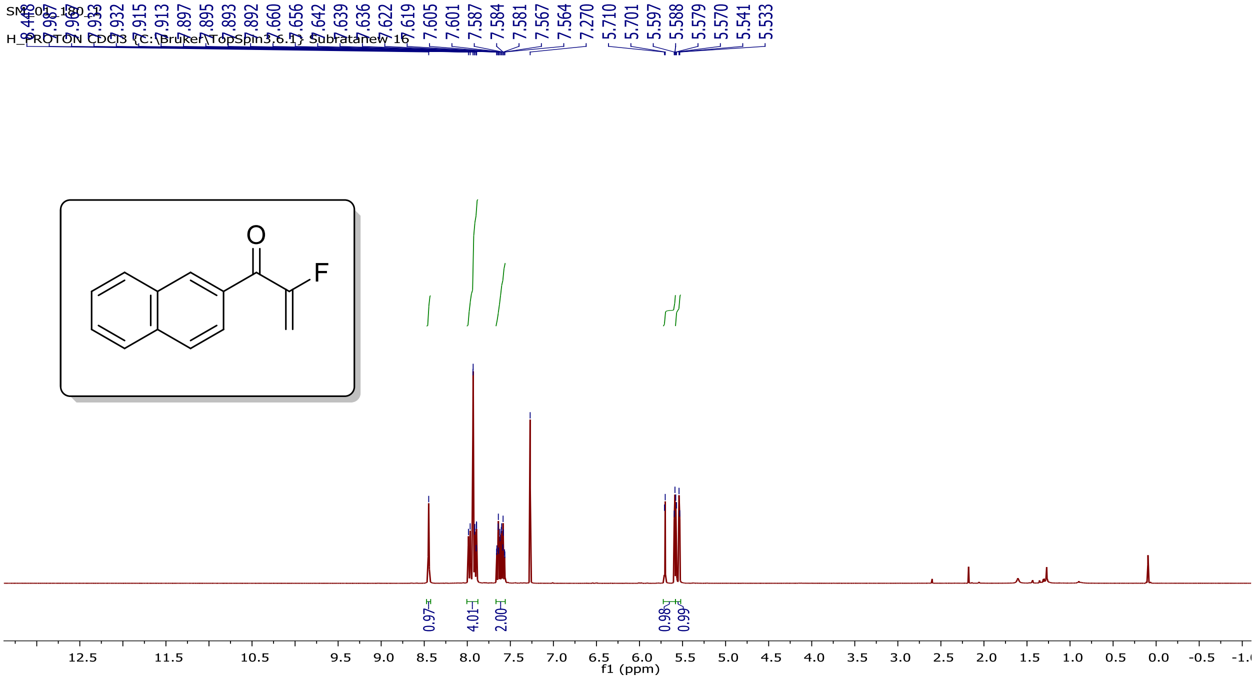

^{1}H NMR Spectrum of Compound **6** (400MHz, $CDCl_3$)

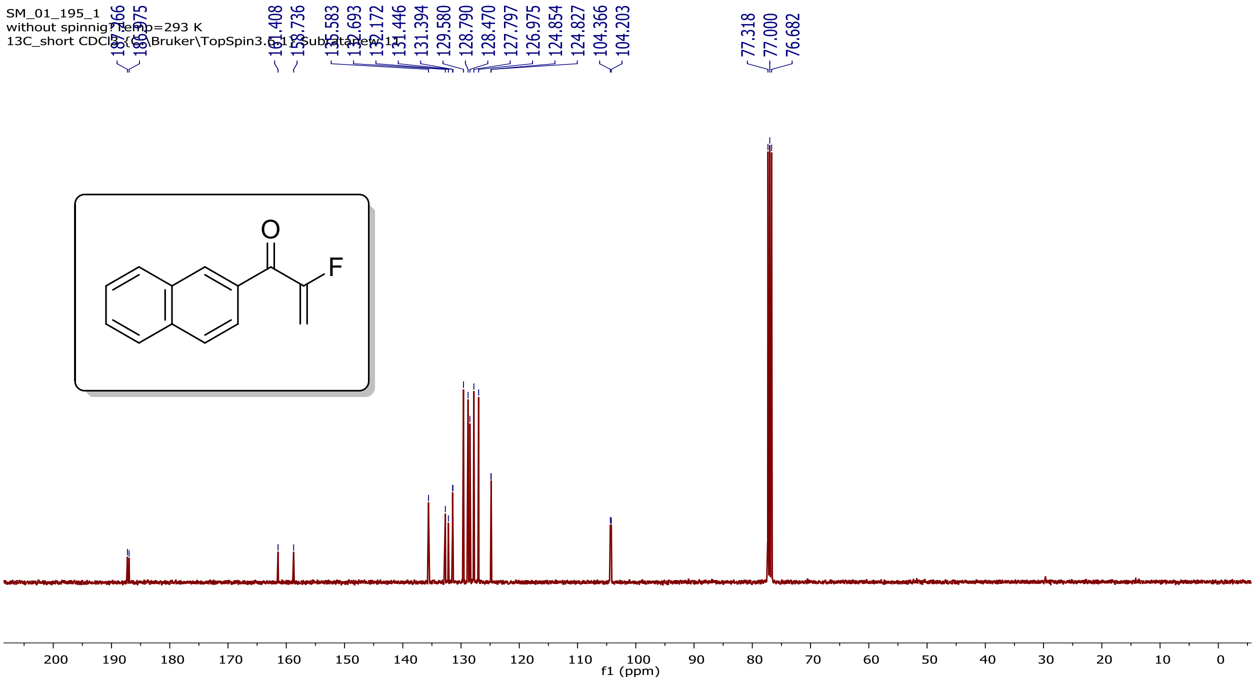

^{13}C NMR Spectrum of Compound **6** (101 MHz, $CDCl_3$)

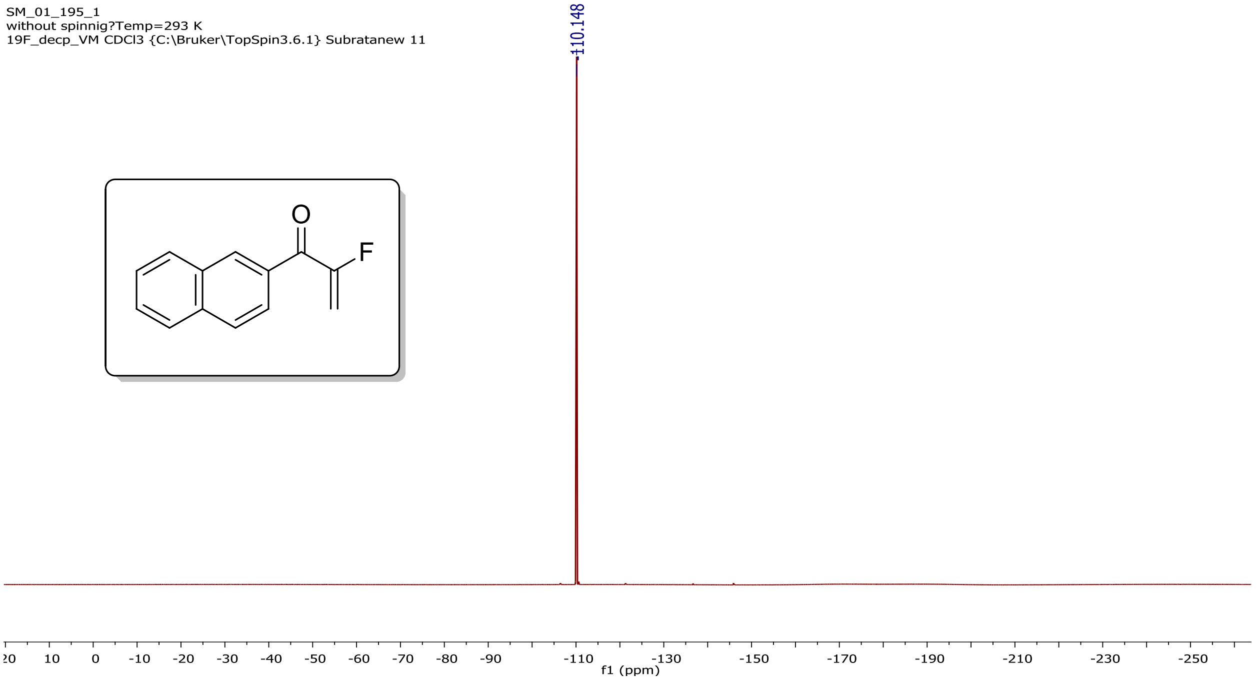

^{19}F NMR Spectrum of Compound **6** (376 MHz, $CDCl_3$)

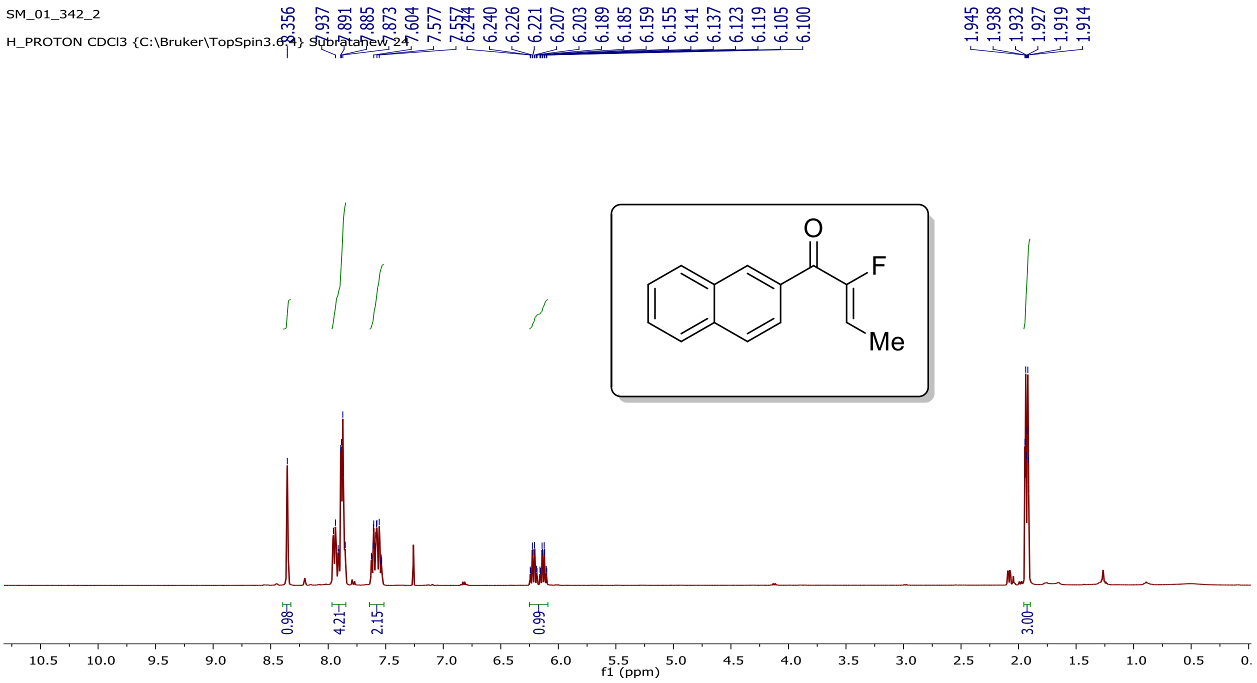

^{1}H NMR Spectrum of Compound **13** (400MHz, $CDCl_3$)

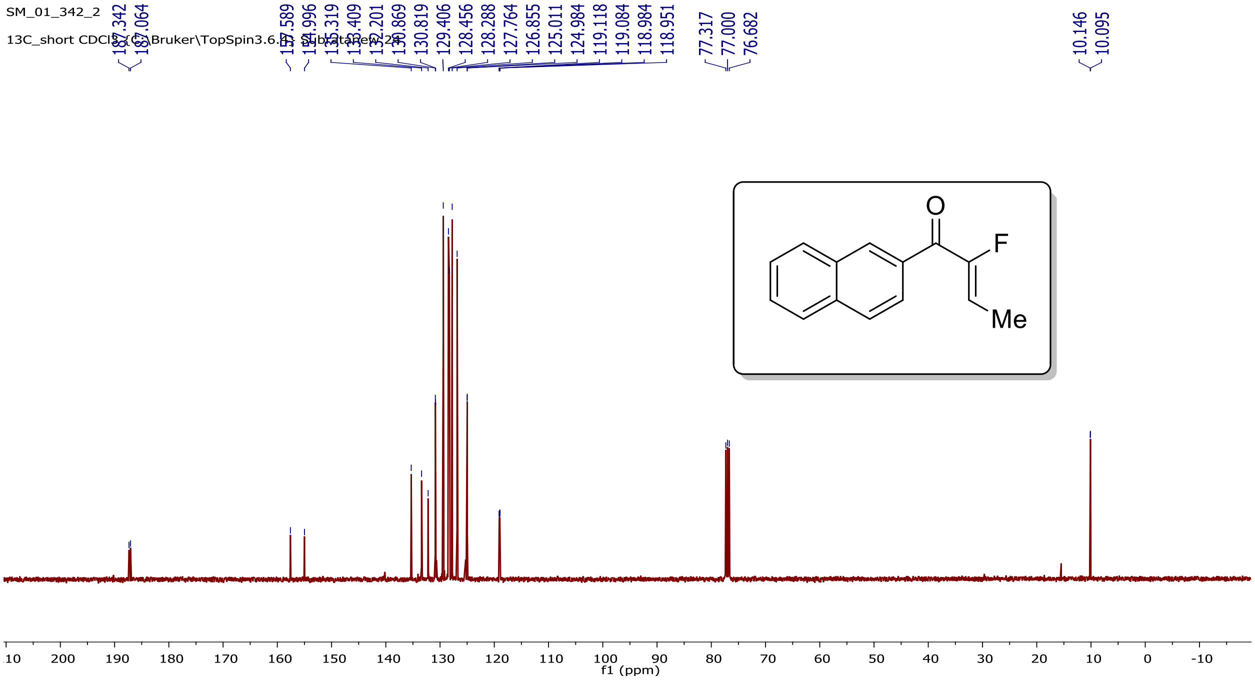

^{13}C NMR Spectrum of Compound **13** (101 MHz, $CDCl_3$)

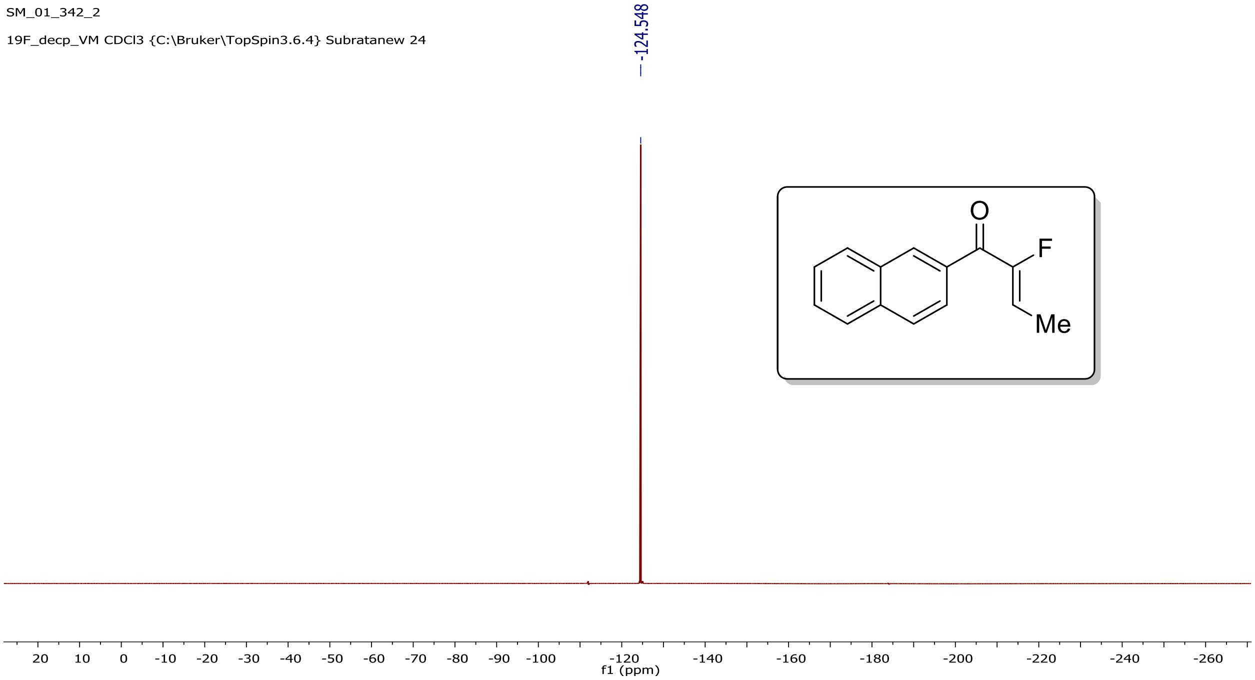

^{19}F NMR Spectrum of Compound **13** (376 MHz, $CDCl_3$)

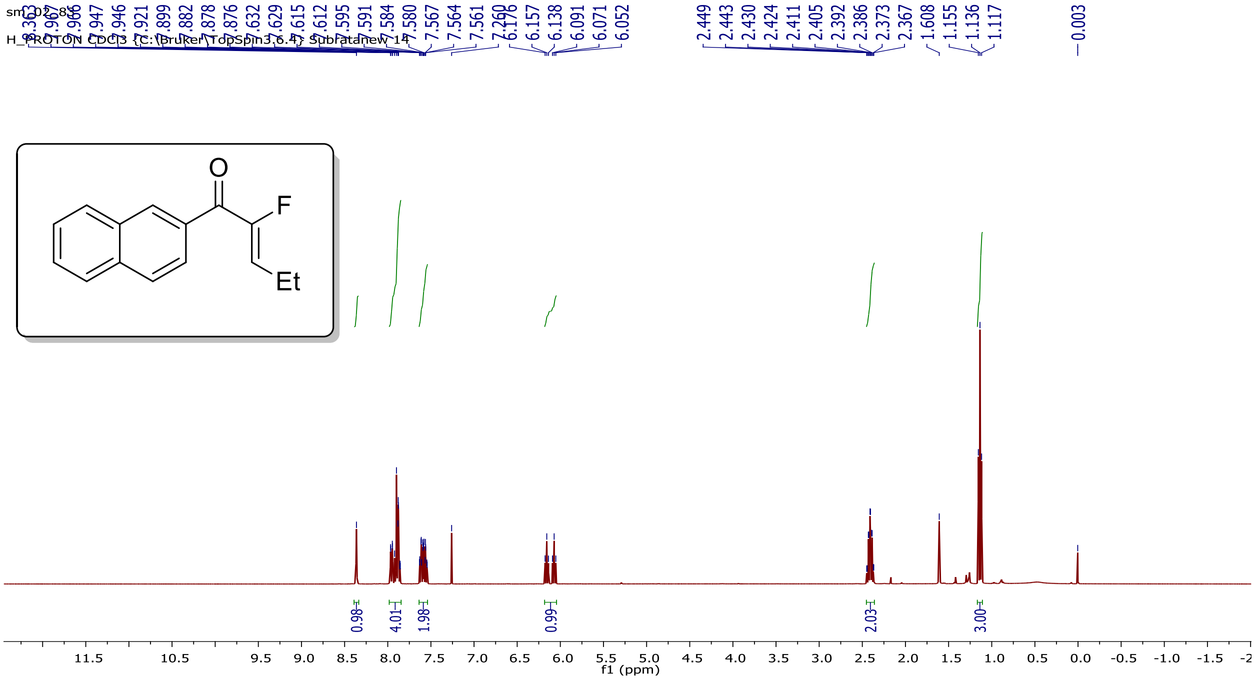

^{1}H NMR Spectrum of Compound **14** (400MHz, $CDCl_3$)

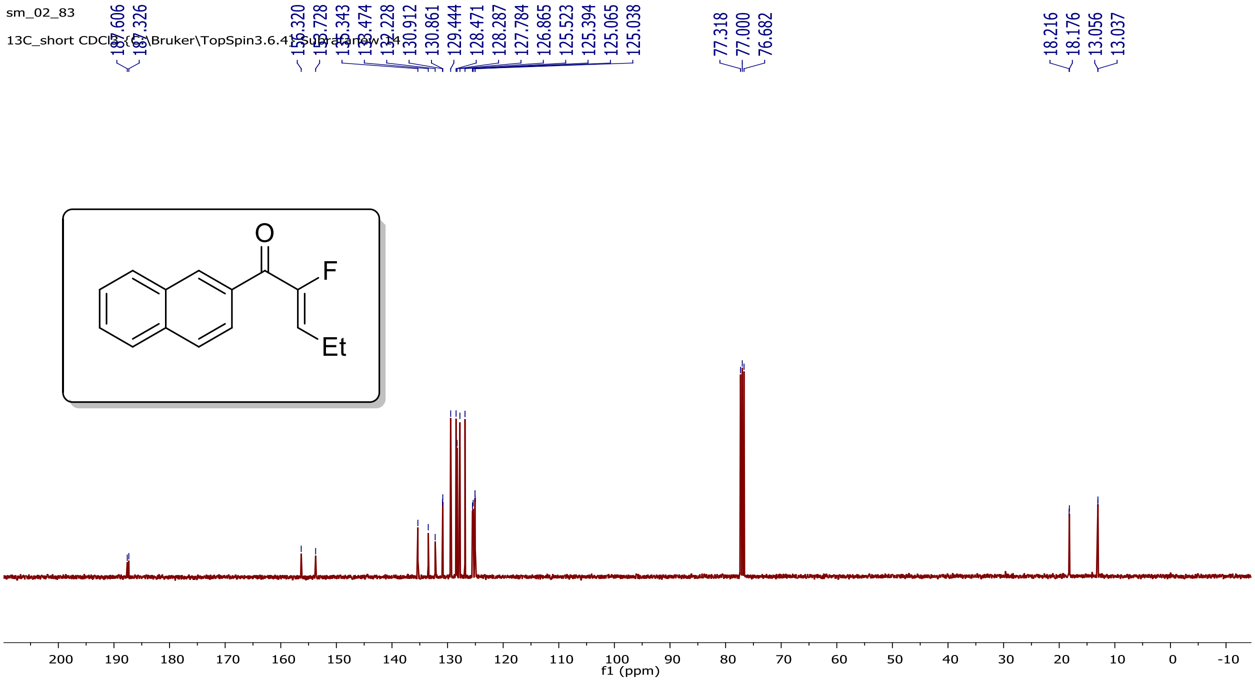

^{13}C NMR Spectrum of Compound **14** (101 MHz, $CDCl_3$)

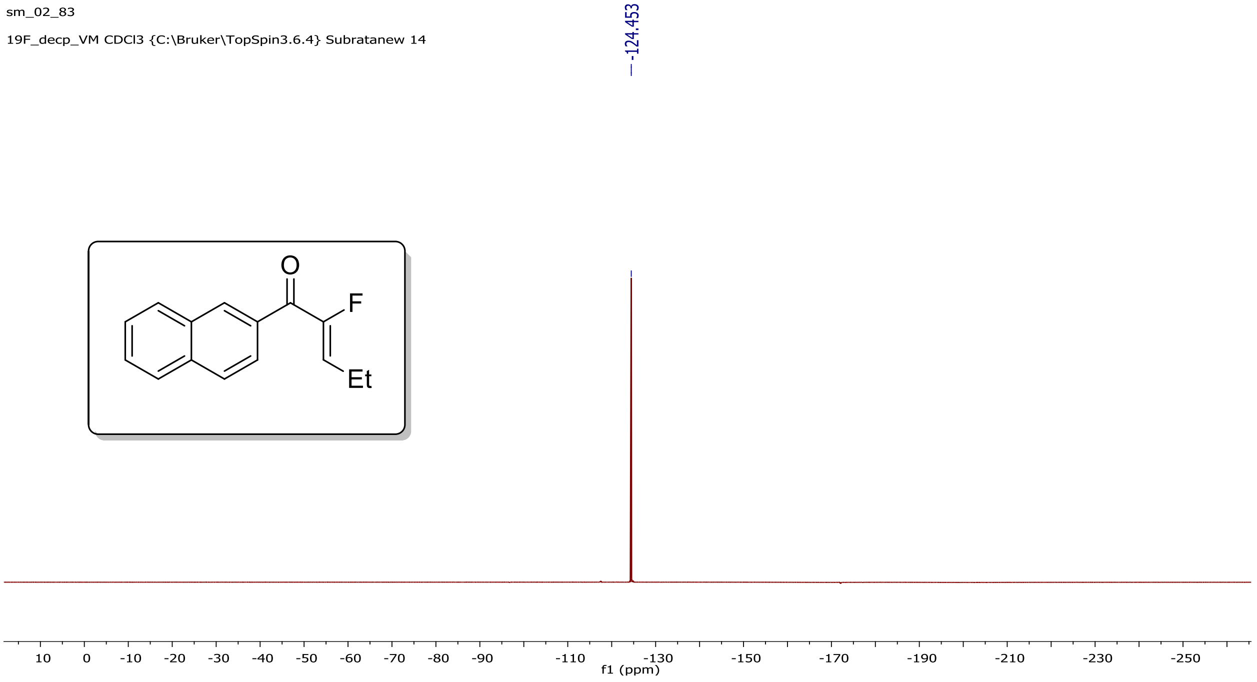

^{19}F NMR Spectrum of Compound **14** (376 MHz, $CDCl_3$)

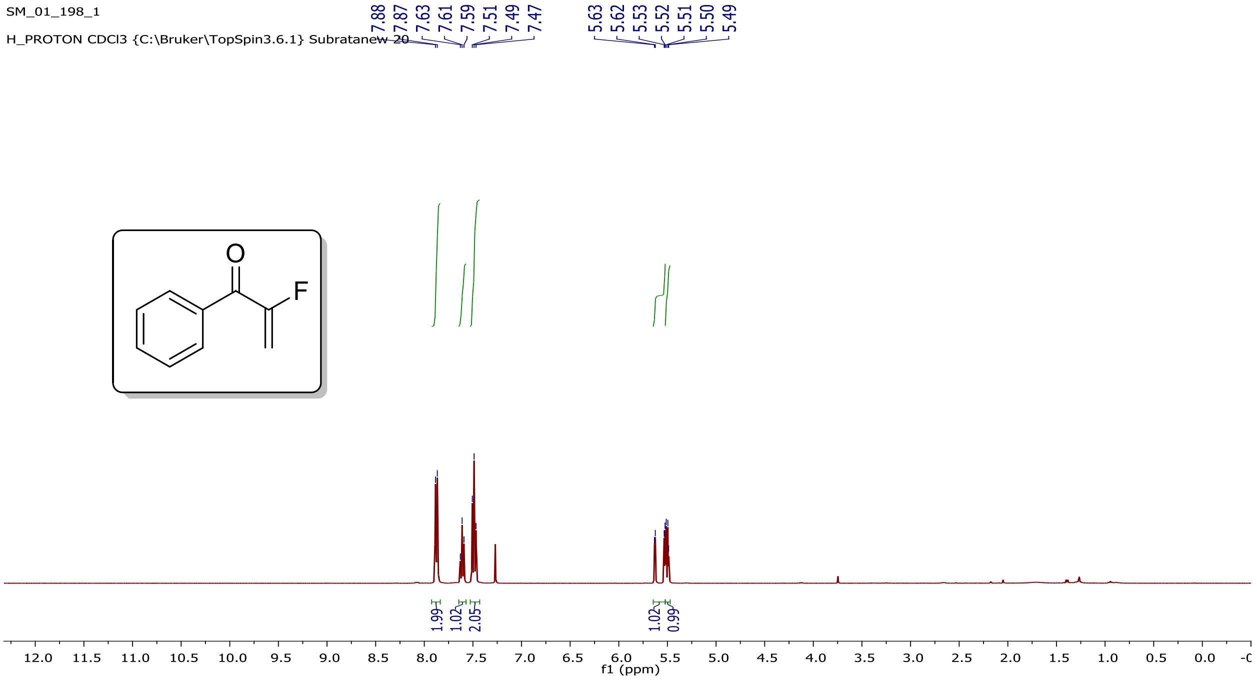

^{1}H NMR Spectrum of Compound **15** (400 MHz, $CDCl_3$)

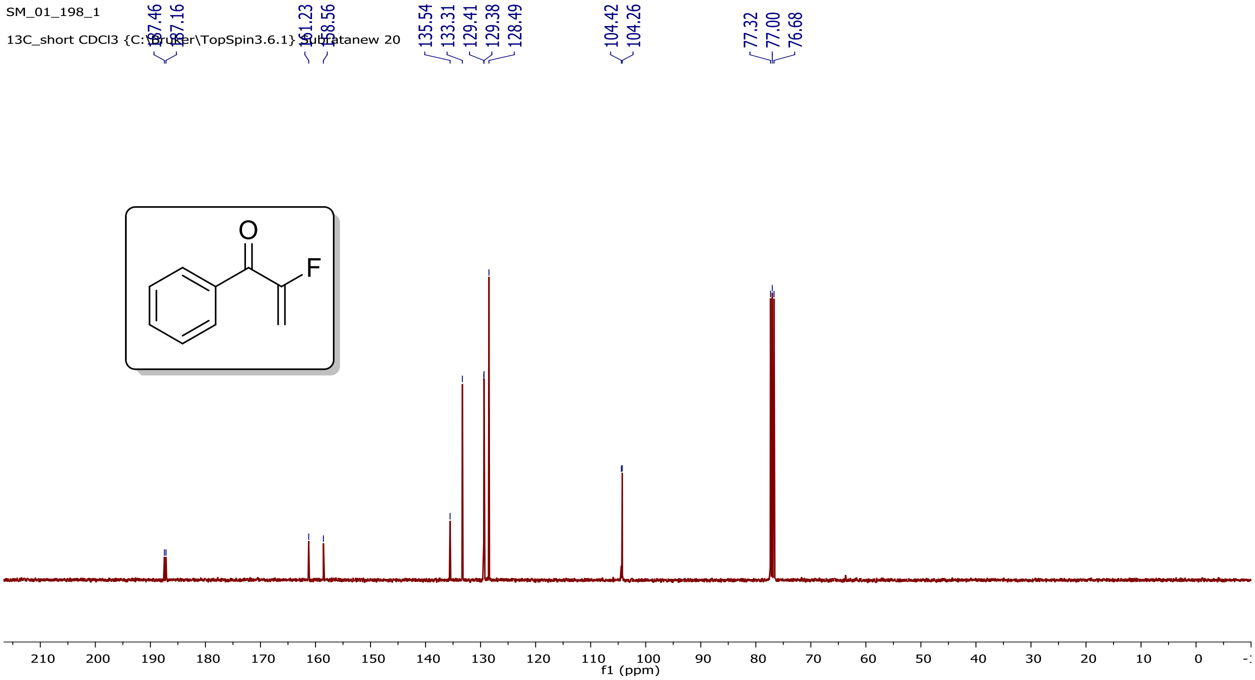

^{13}C NMR Spectrum of Compound **15** (101 MHz, $CDCl_3$)

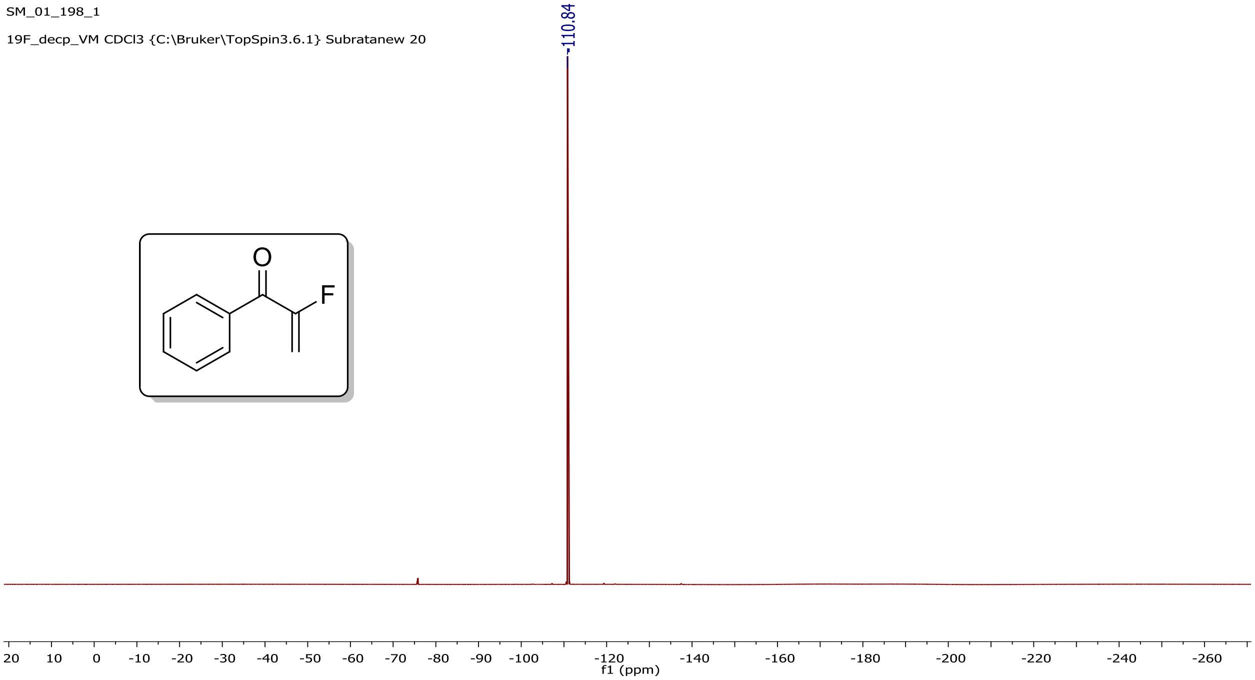

^{19}F NMR Spectrum of Compound **15** (376 MHz, $CDCl_3$)

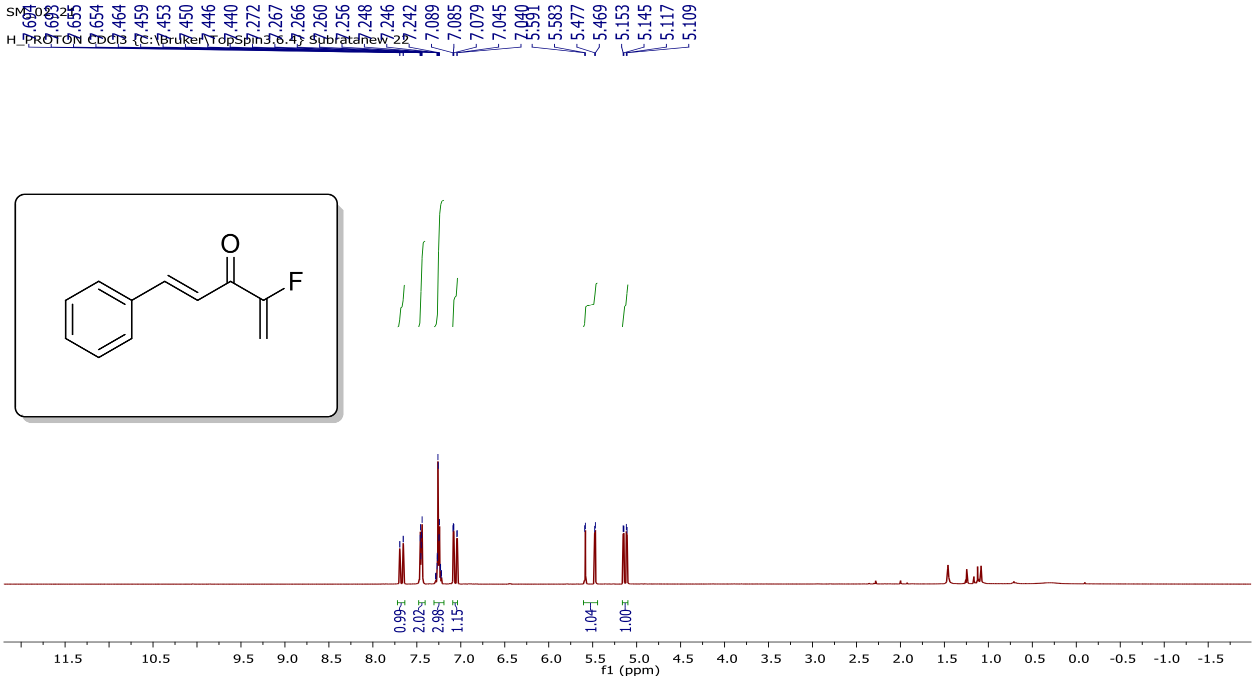

^{1}H NMR Spectrum of Compound **16** (400 MHz, $CDCl_3$)

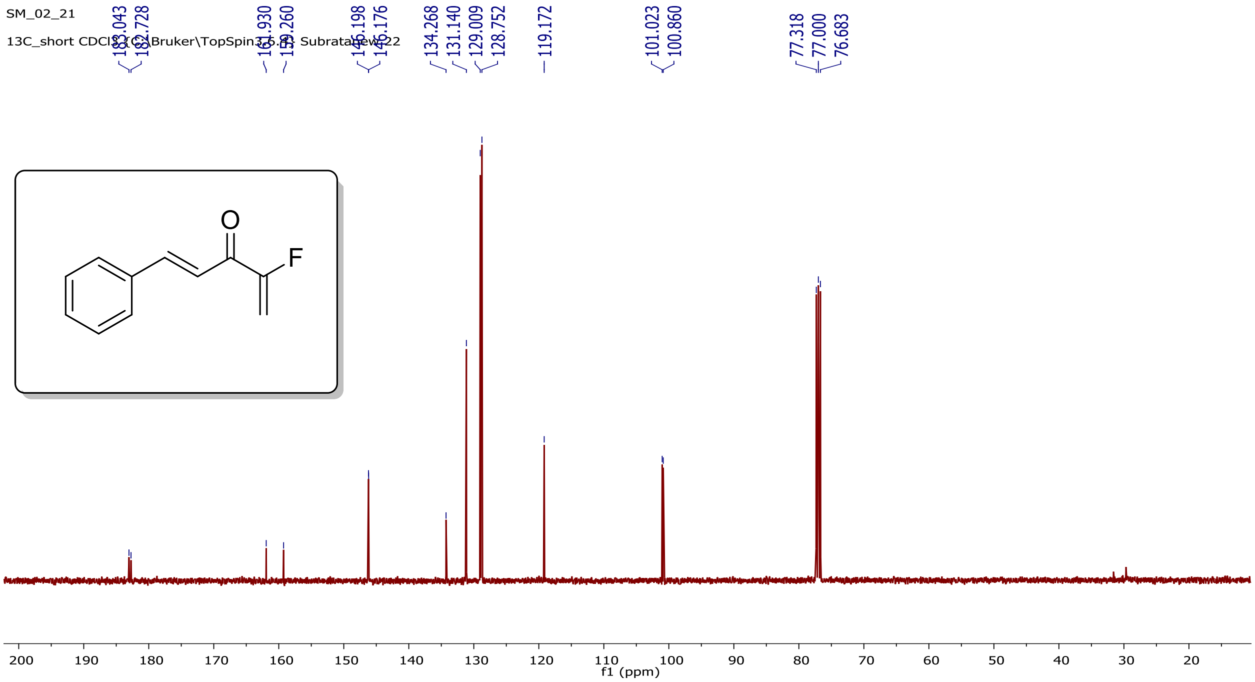

^{13}C NMR Spectrum of Compound **16** (101 MHz, $CDCl_3$)

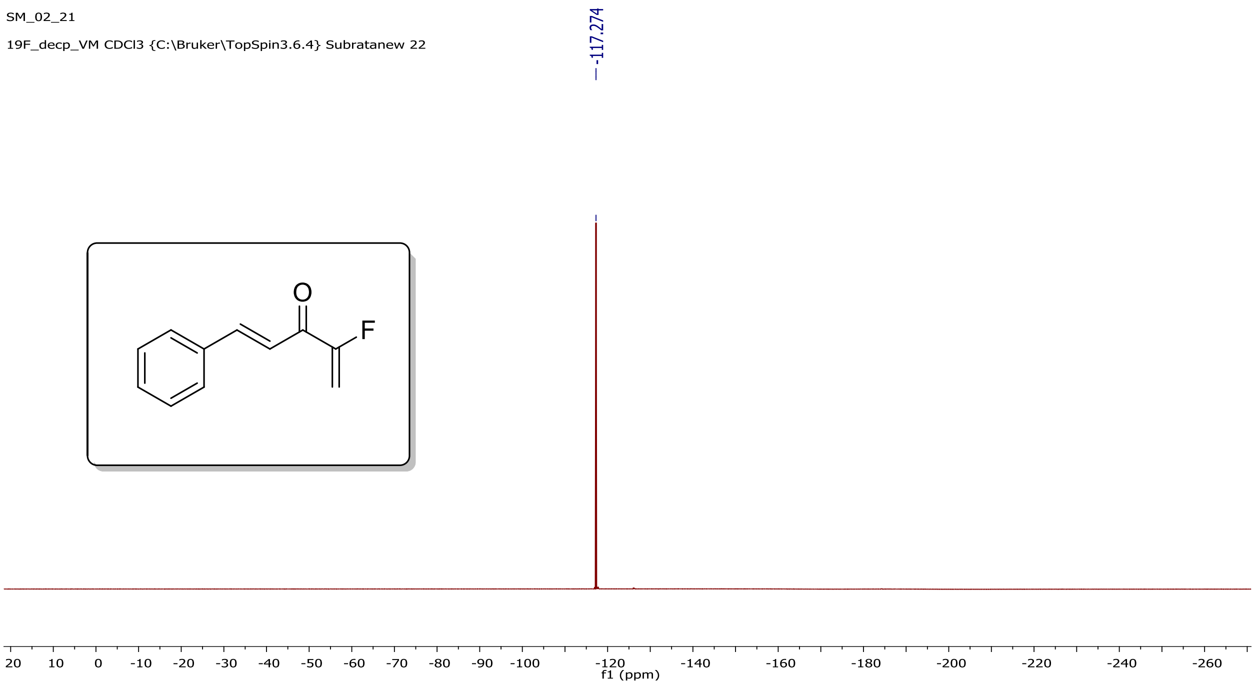

^{19}F NMR Spectrum of Compound **16** (376 MHz, $CDCl_3$)

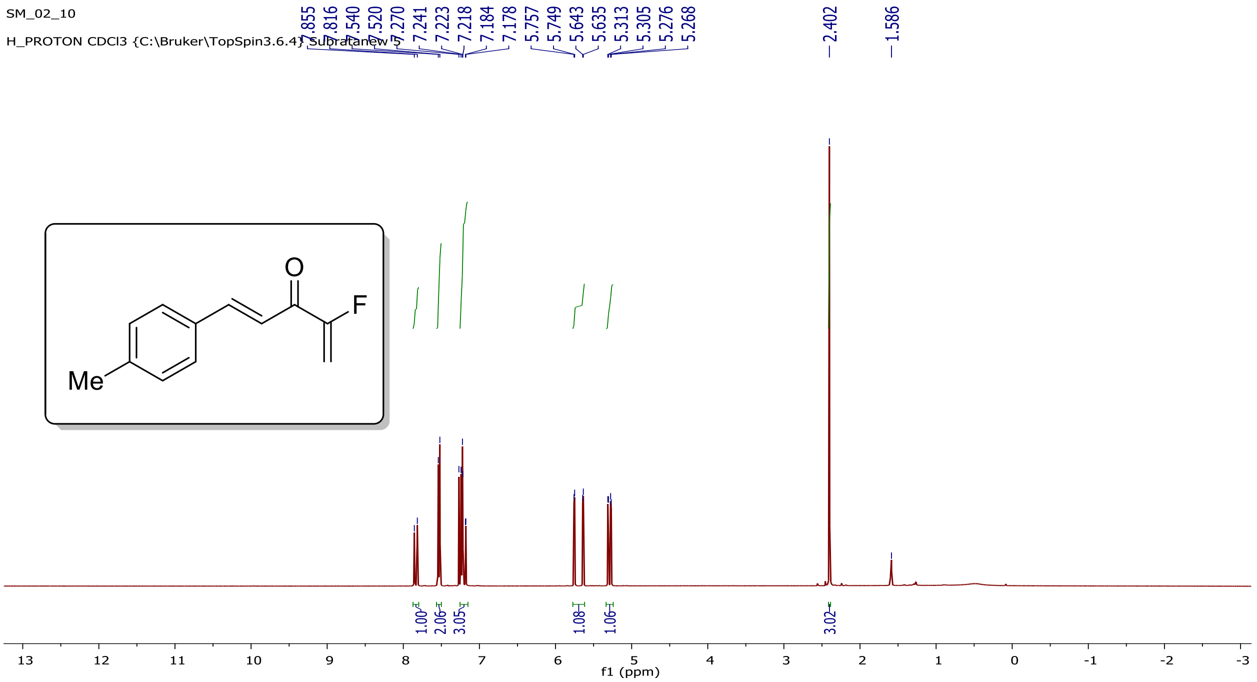

^{1}H NMR Spectrum of Compound **17** (400 MHz, $CDCl_3$)

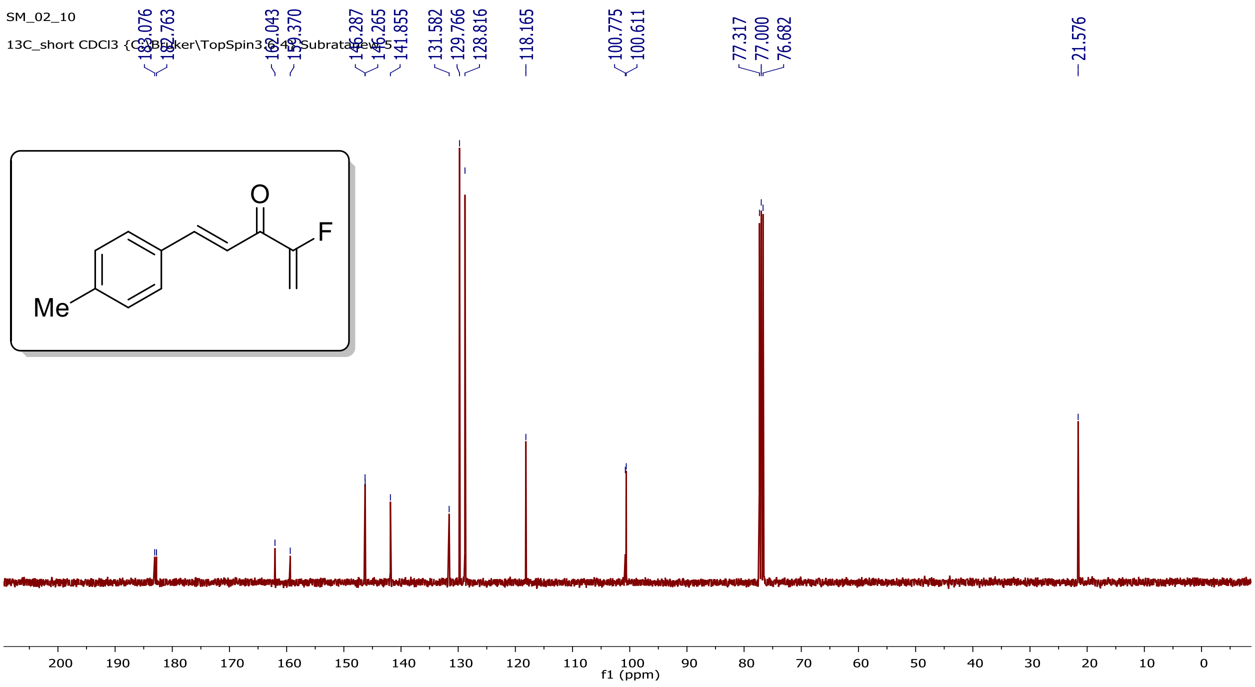

^{13}C NMR Spectrum of Compound **17** (101 MHz, $CDCl_3$)

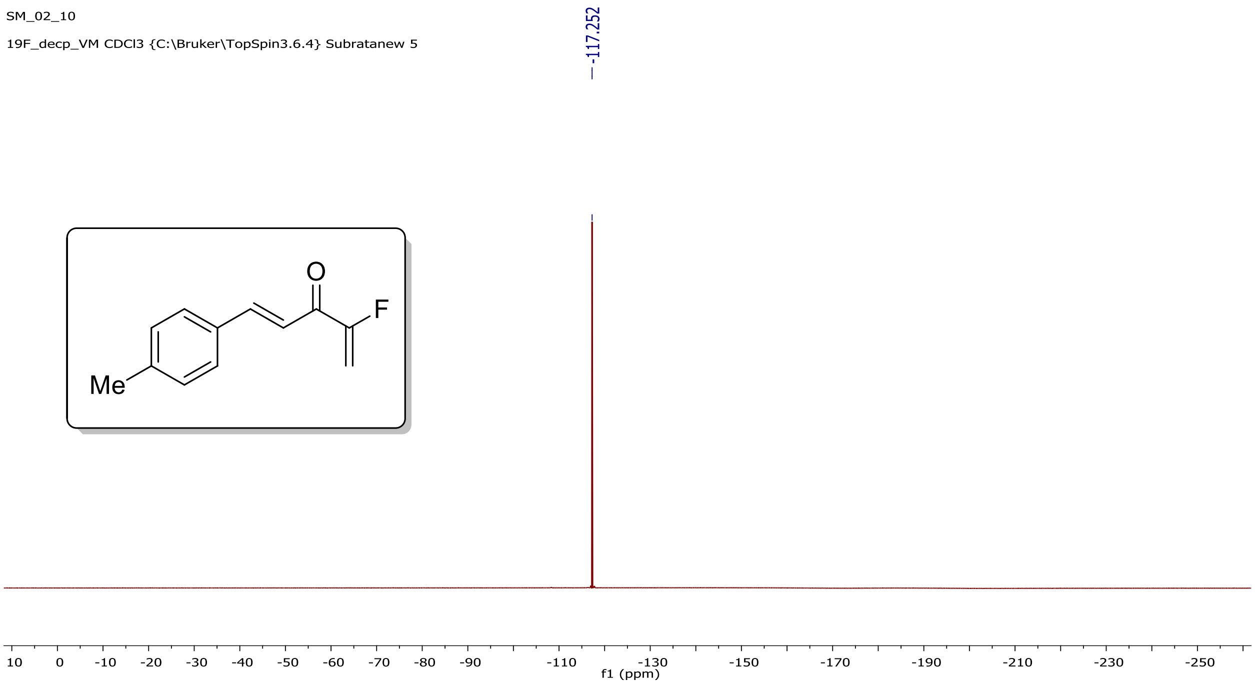

^{19}F NMR Spectrum of Compound **17** (376 MHz, $CDCl_3$)

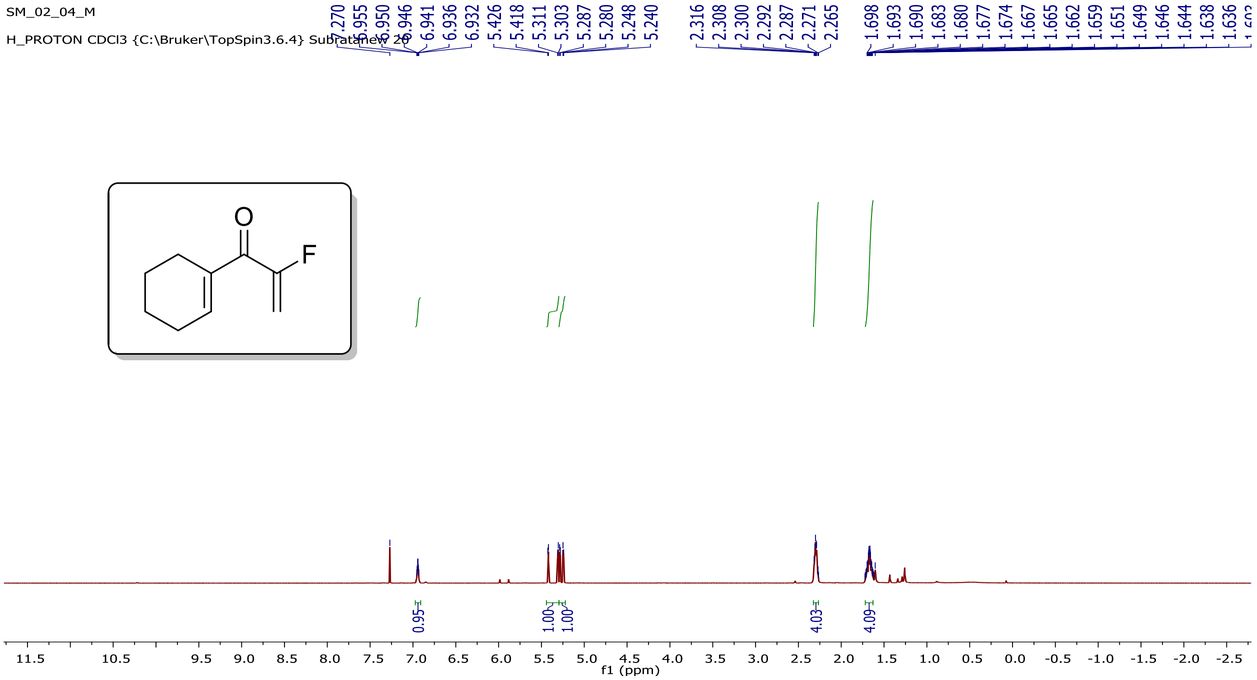

^{1}H NMR Spectrum of Compound **18** (400 MHz, $CDCl_3$)

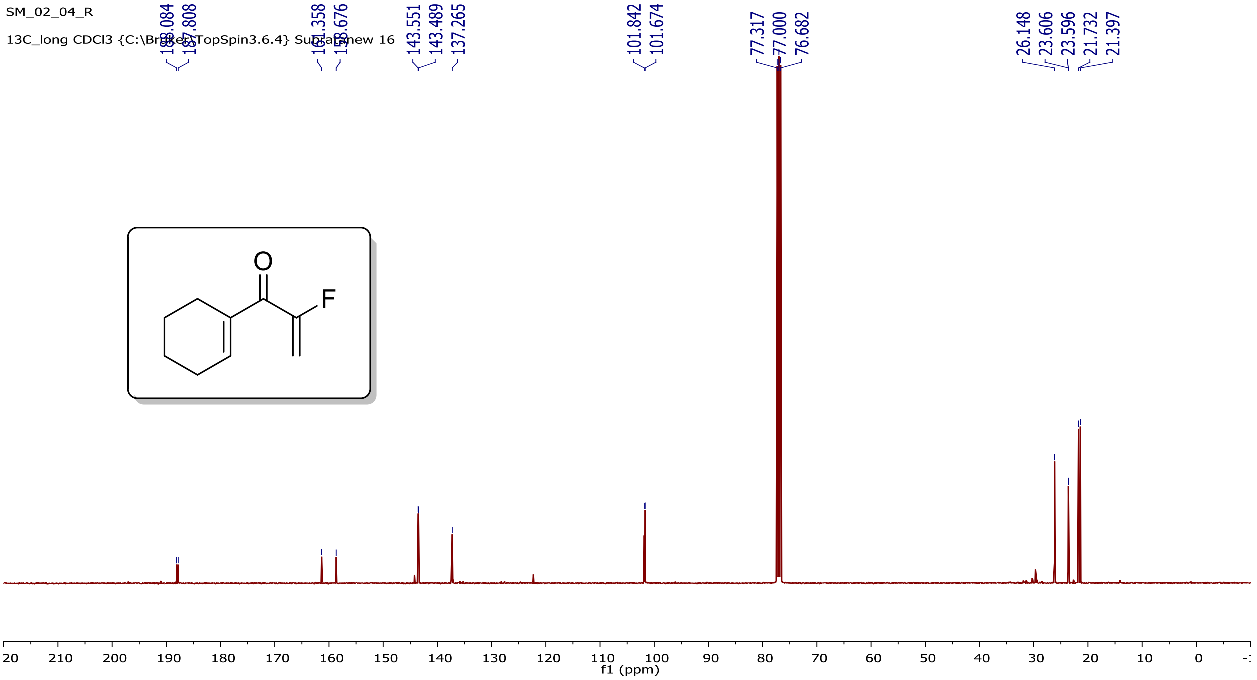

^{13}C NMR Spectrum of Compound **18** (101 MHz, $CDCl_3$)

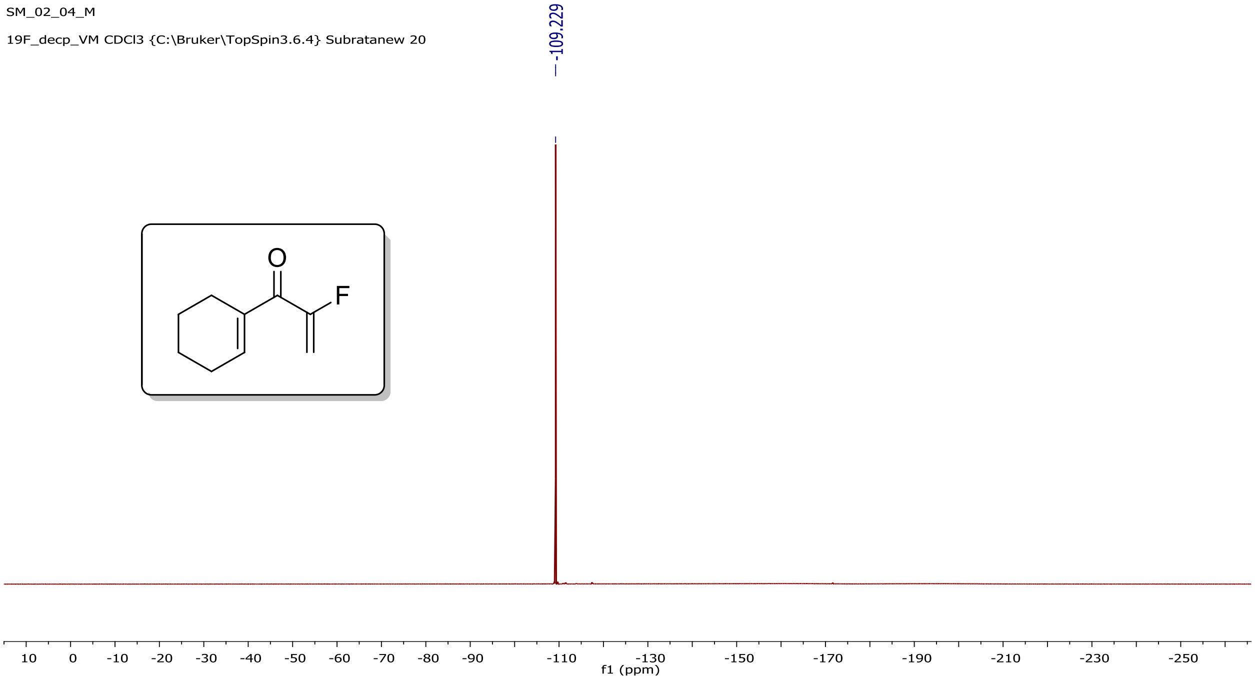

^{19}F NMR Spectrum of Compound **18** (376 MHz, $CDCl_3$)

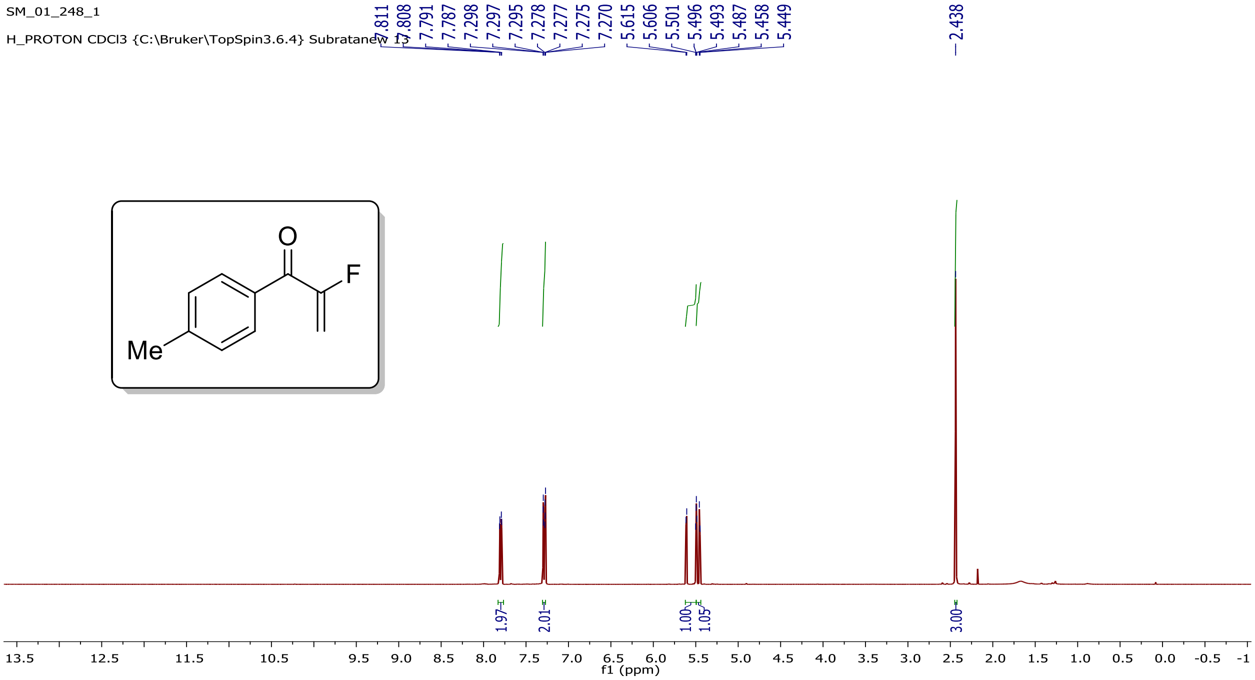

^{1}H NMR Spectrum of Compound **19** (400 MHz, $CDCl_3$)

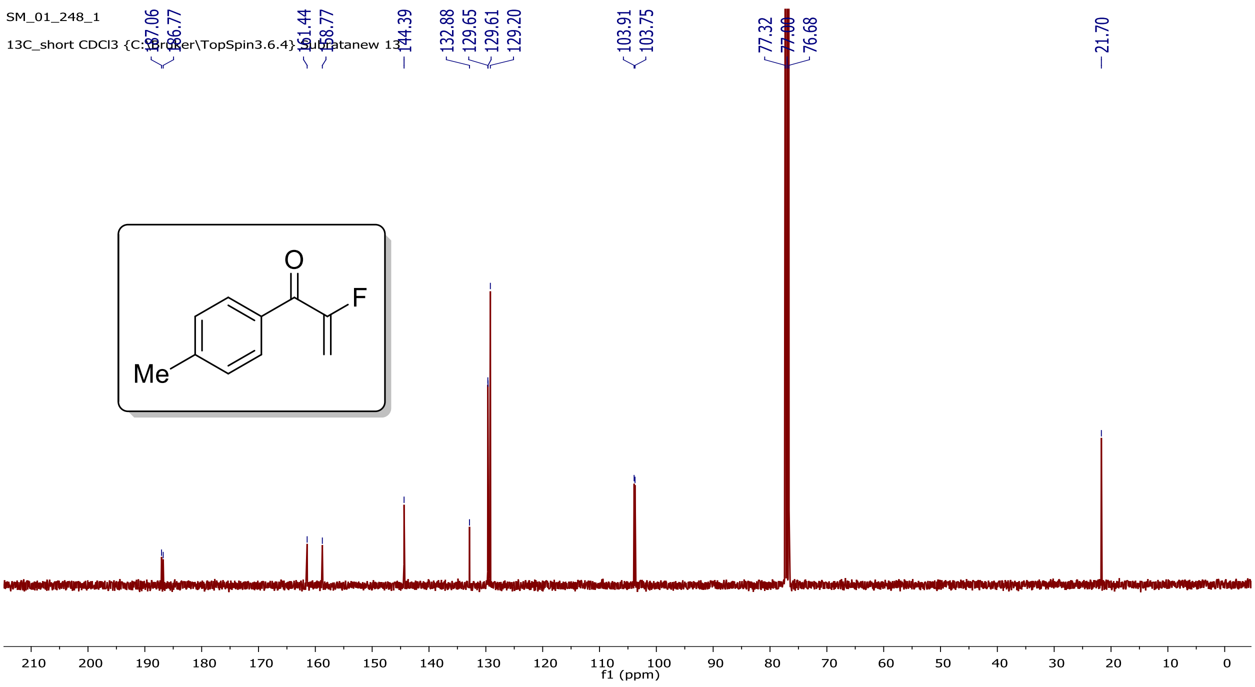

^{13}C NMR Spectrum of Compound **19** (101 MHz, $CDCl_3$)

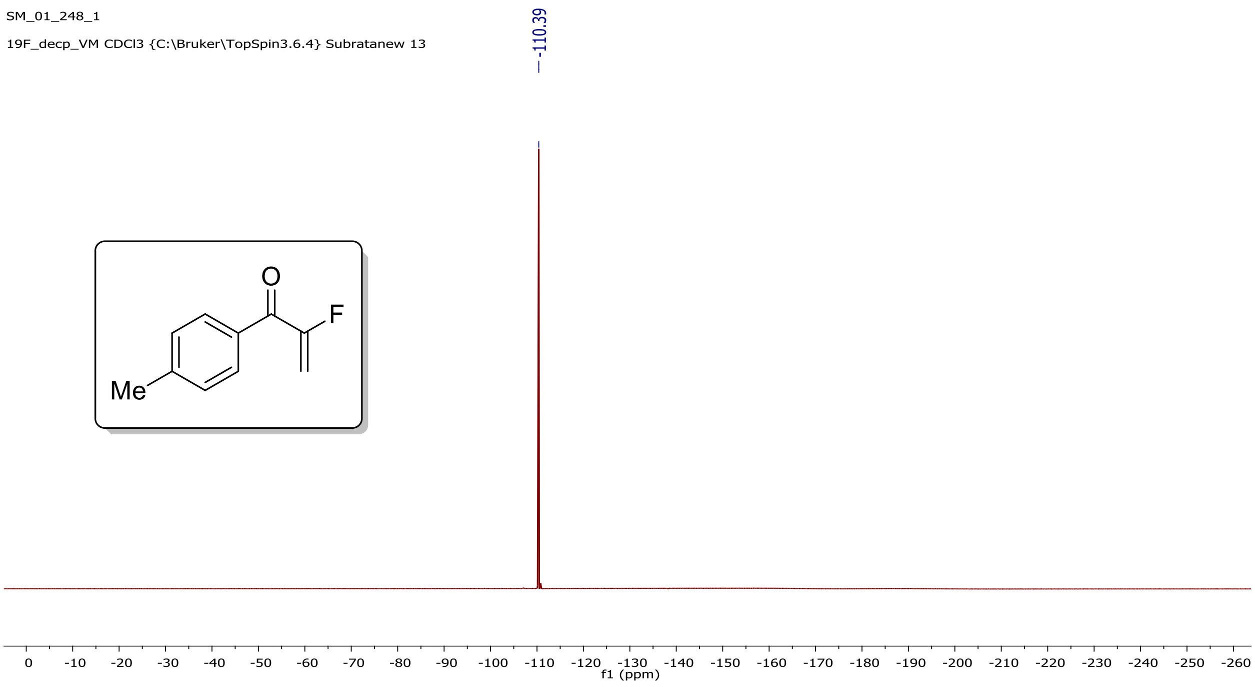

^{19}F NMR Spectrum of Compound **19** (376 MHz, $CDCl_3$)

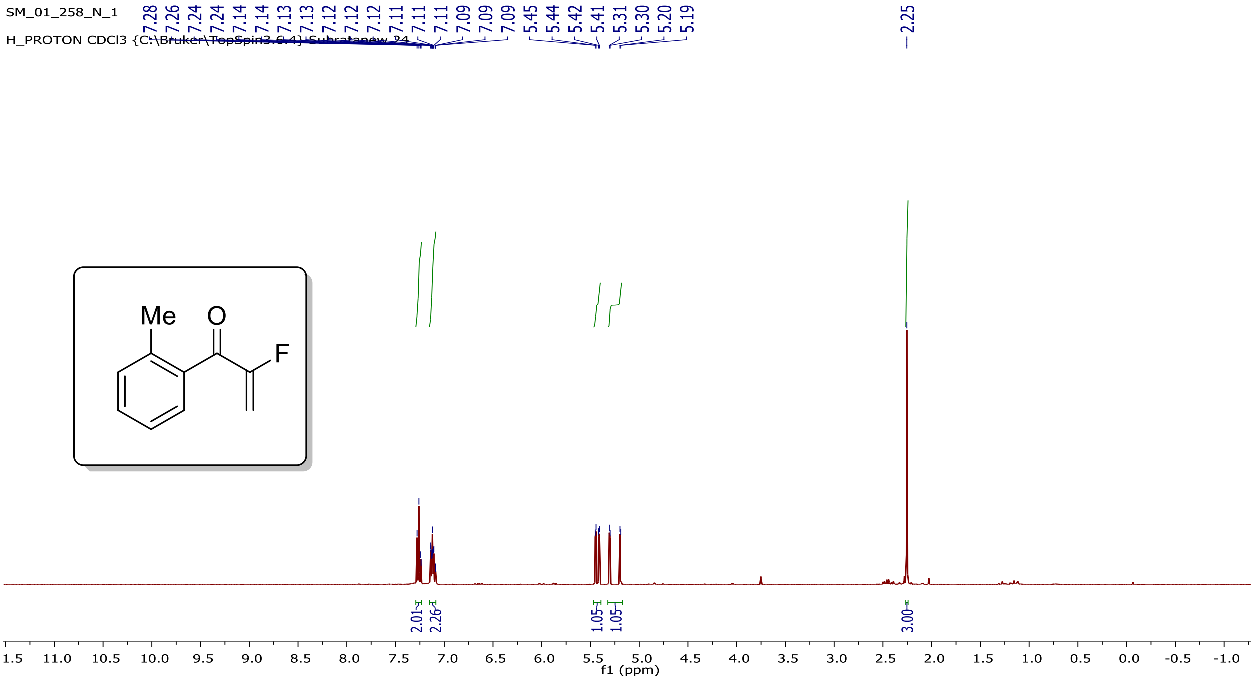

^{1}H NMR Spectrum of Compound **20** (400 MHz, $CDCl_3$)

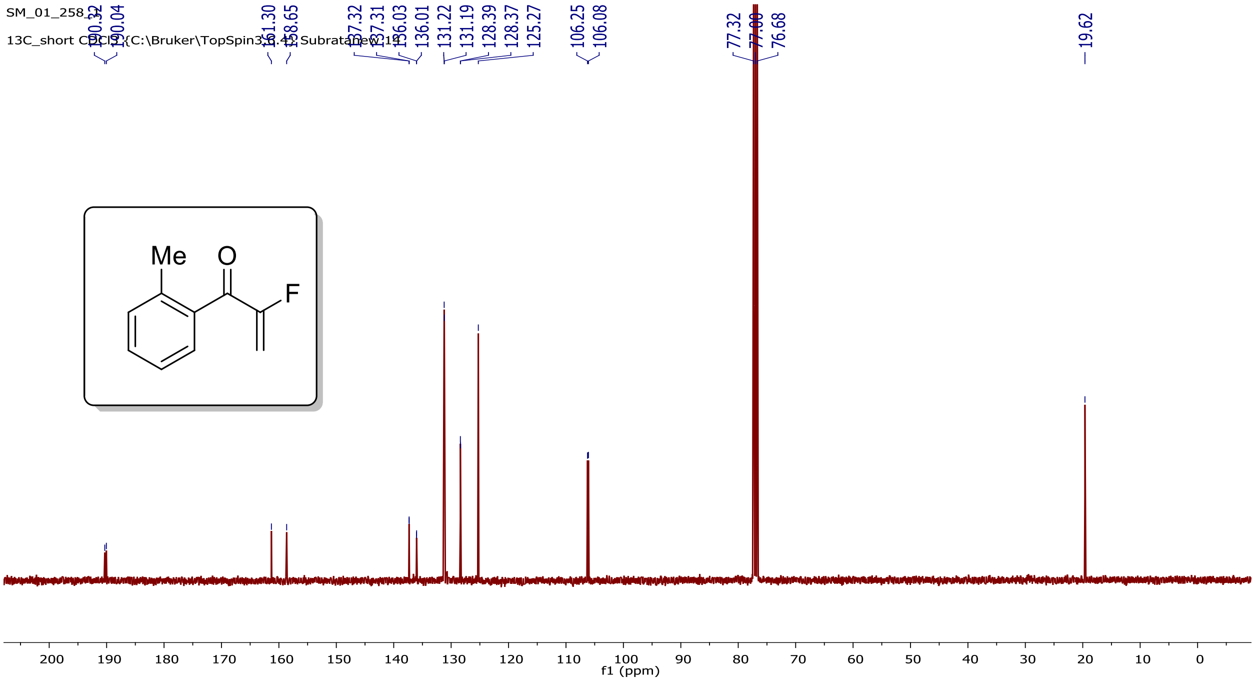

^{13}C NMR Spectrum of Compound **20** (101 MHz, $CDCl_3$)

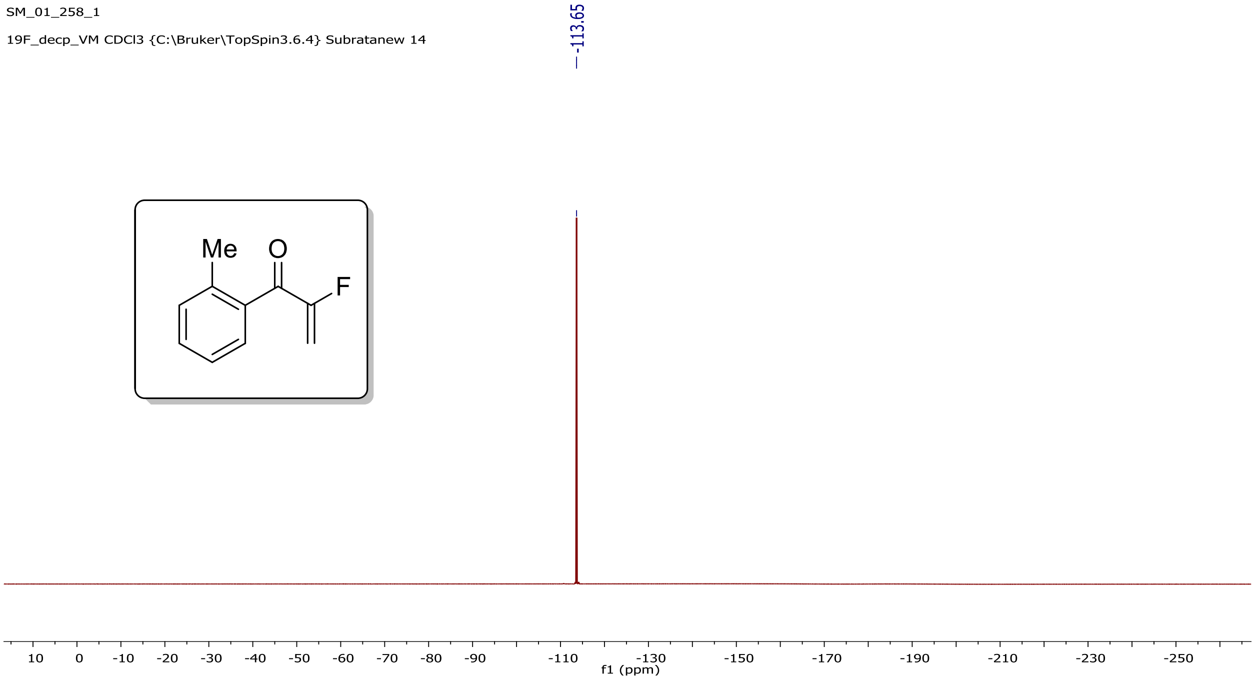

^{19}F NMR Spectrum of Compound **20** (376 MHz, $CDCl_3$)

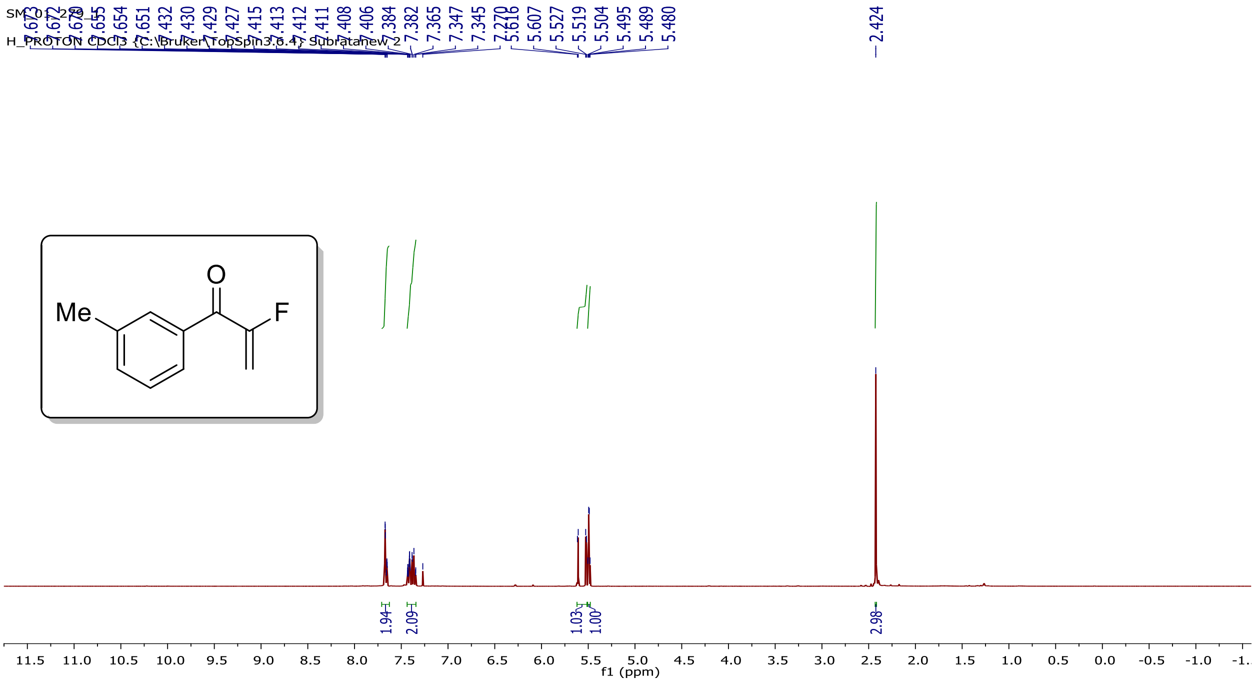

^{1}H NMR Spectrum of Compound **21** (400 MHz, $CDCl_3$)

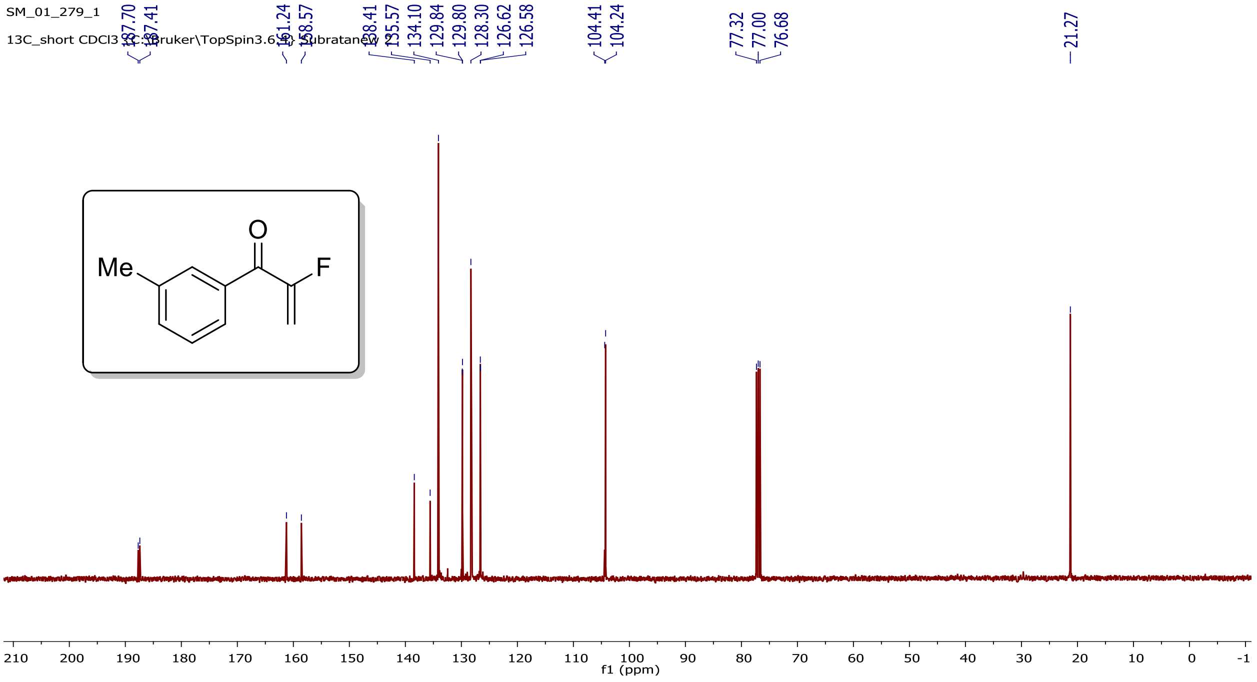

^{13}C NMR Spectrum of Compound **21** (101 MHz, $CDCl_3$)

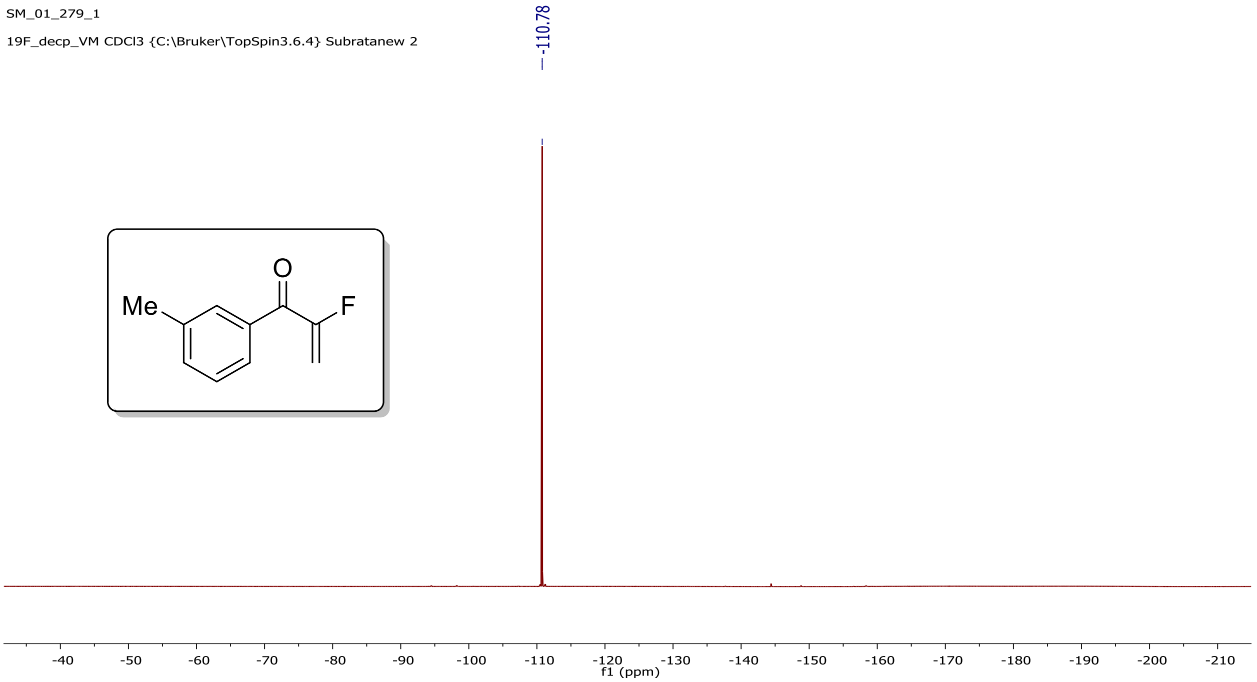

^{19}F NMR Spectrum of Compound **21** (376 MHz, $CDCl_3$)

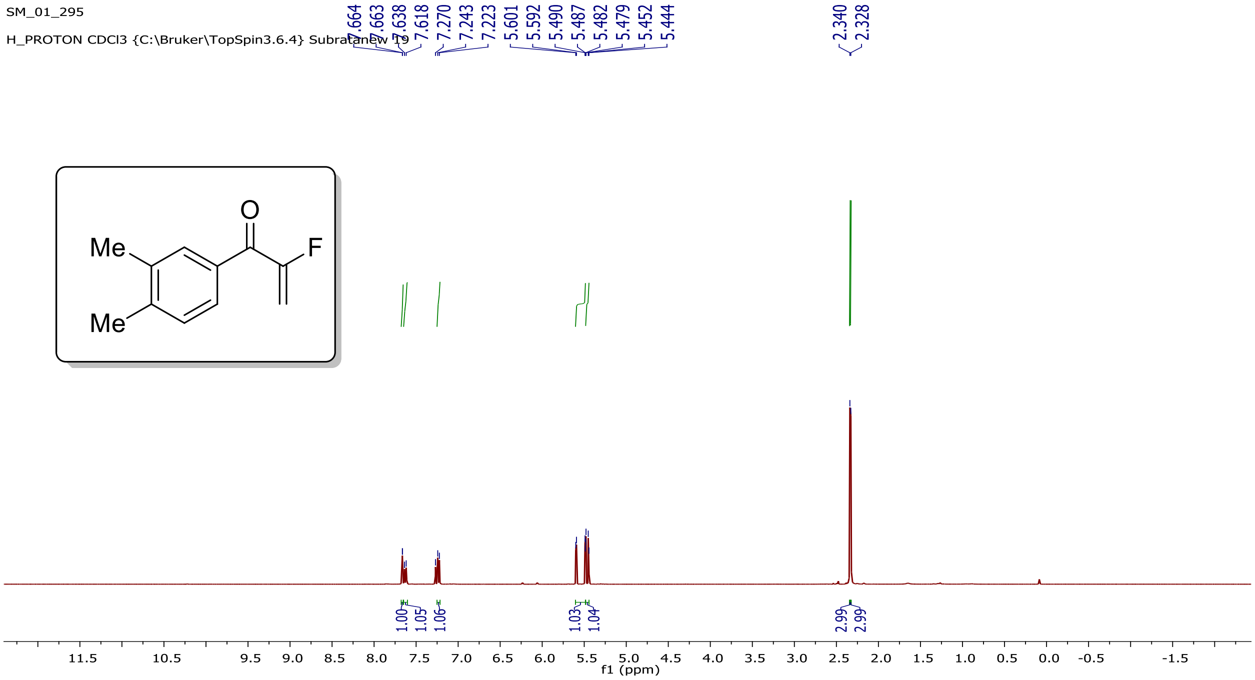

^{1}H NMR Spectrum of Compound **22** (400 MHz, $CDCl_3$)

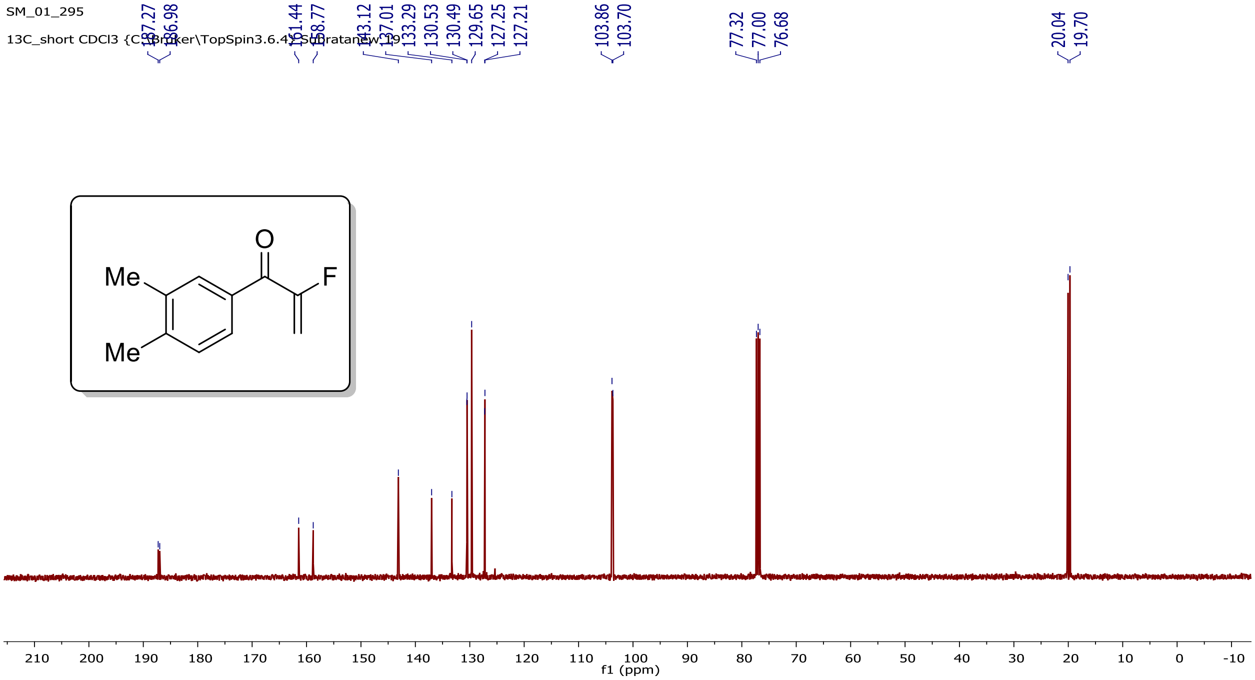

^{13}C NMR Spectrum of Compound **22** (101 MHz, $CDCl_3$)

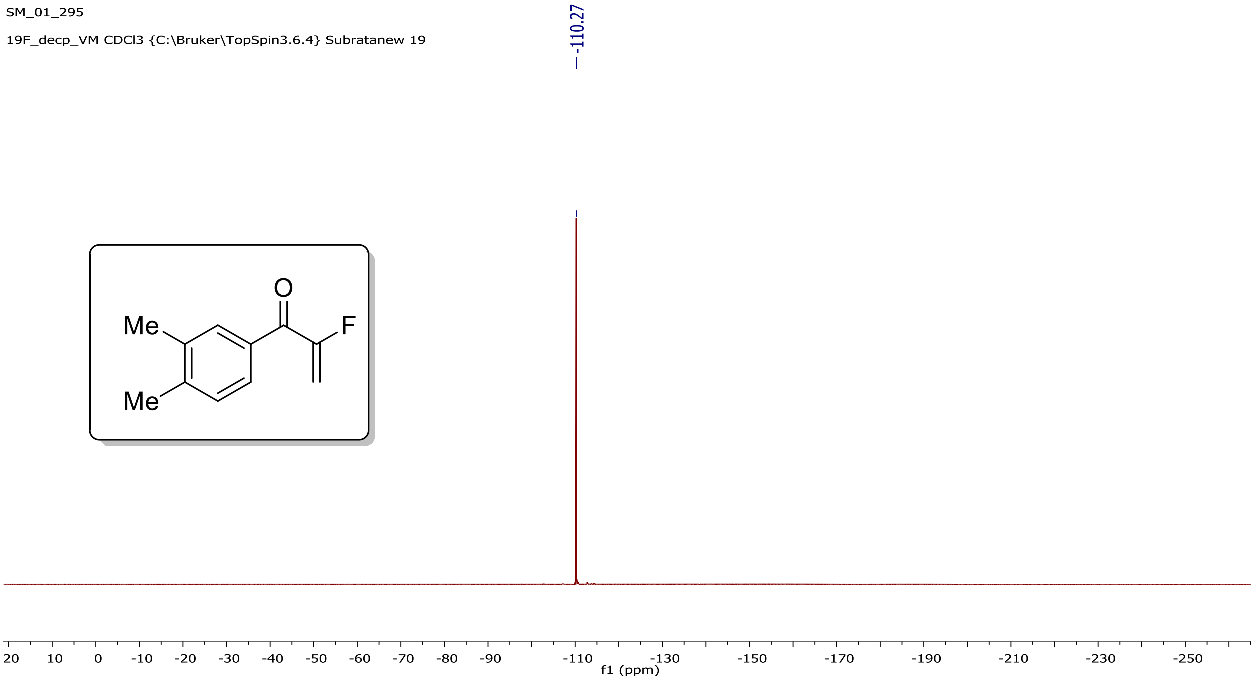

^{19}F NMR Spectrum of Compound **22** (376 MHz, $CDCl_3$)

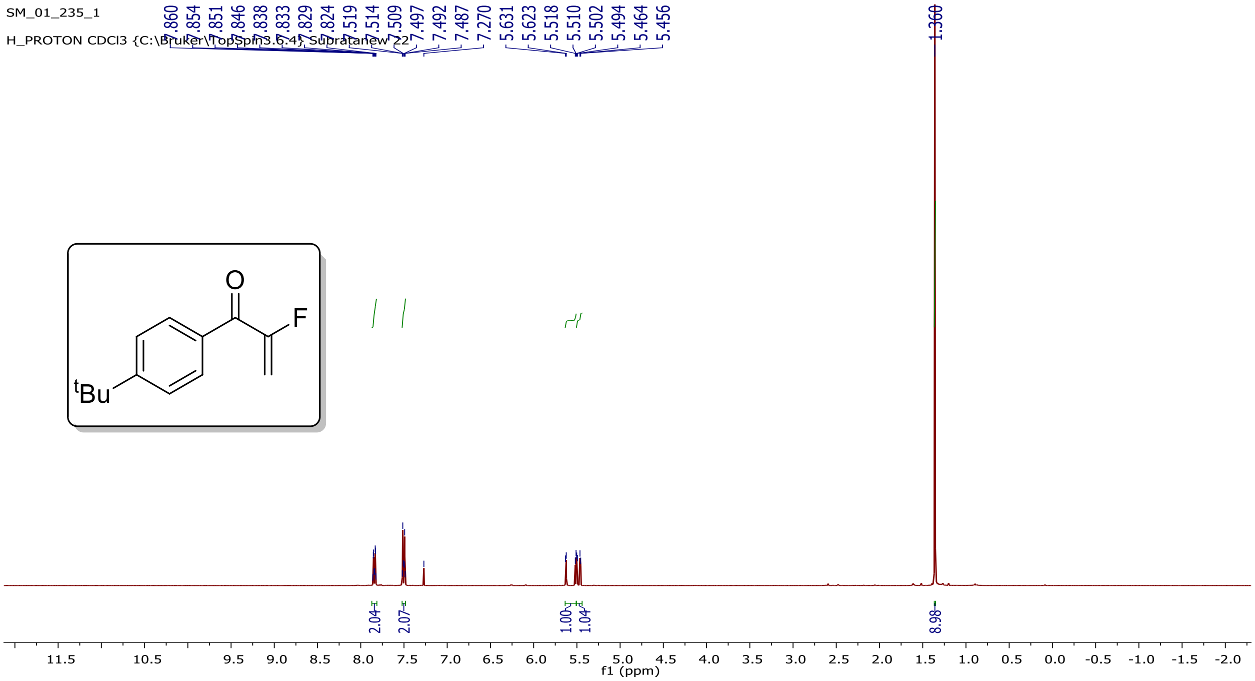

^{1}H NMR Spectrum of Compound **23** (400 MHz, $CDCl_3$)

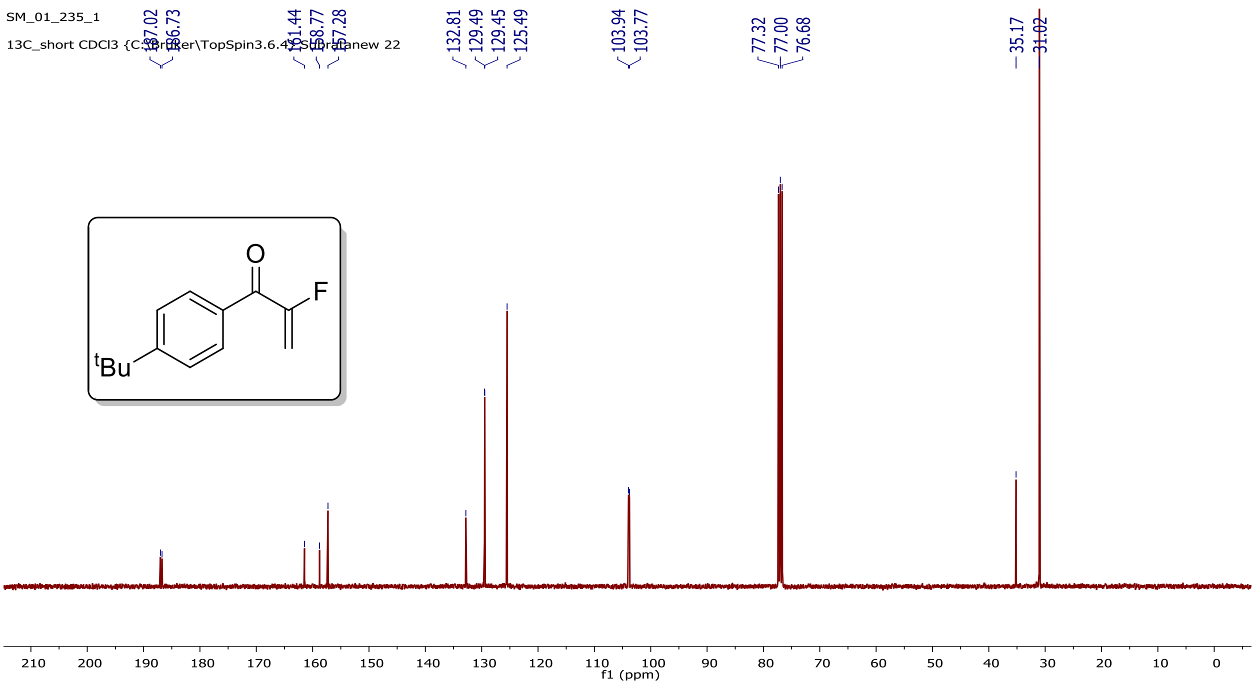

^{13}C NMR Spectrum of Compound **23** (101 MHz, $CDCl_3$)

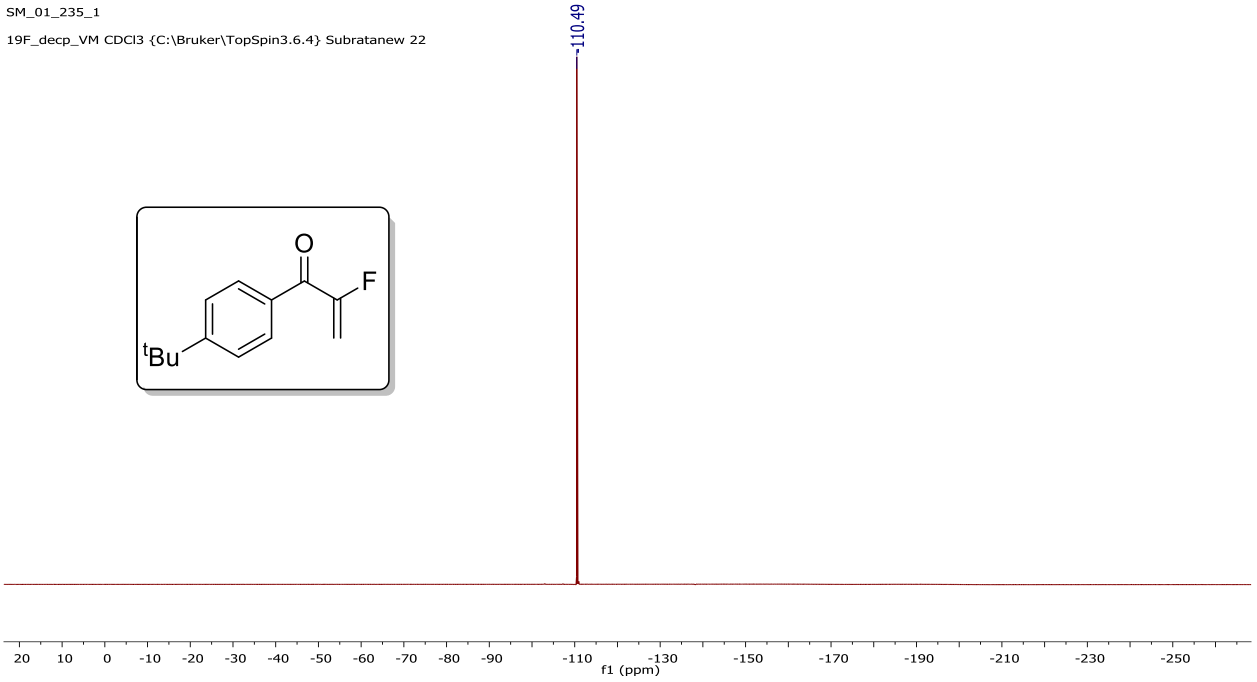

^{19}F NMR Spectrum of Compound **23** (376 MHz, $CDCl_3$)

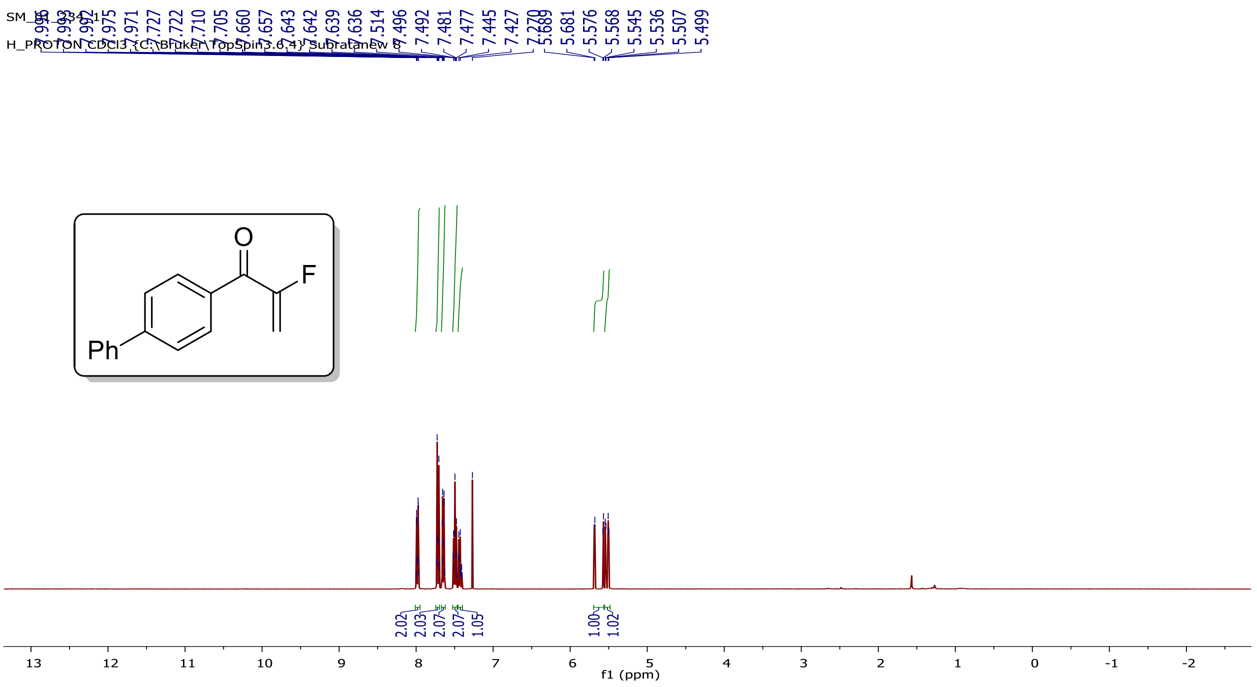

^{1}H NMR Spectrum of Compound **24** (400 MHz, $CDCl_3$)

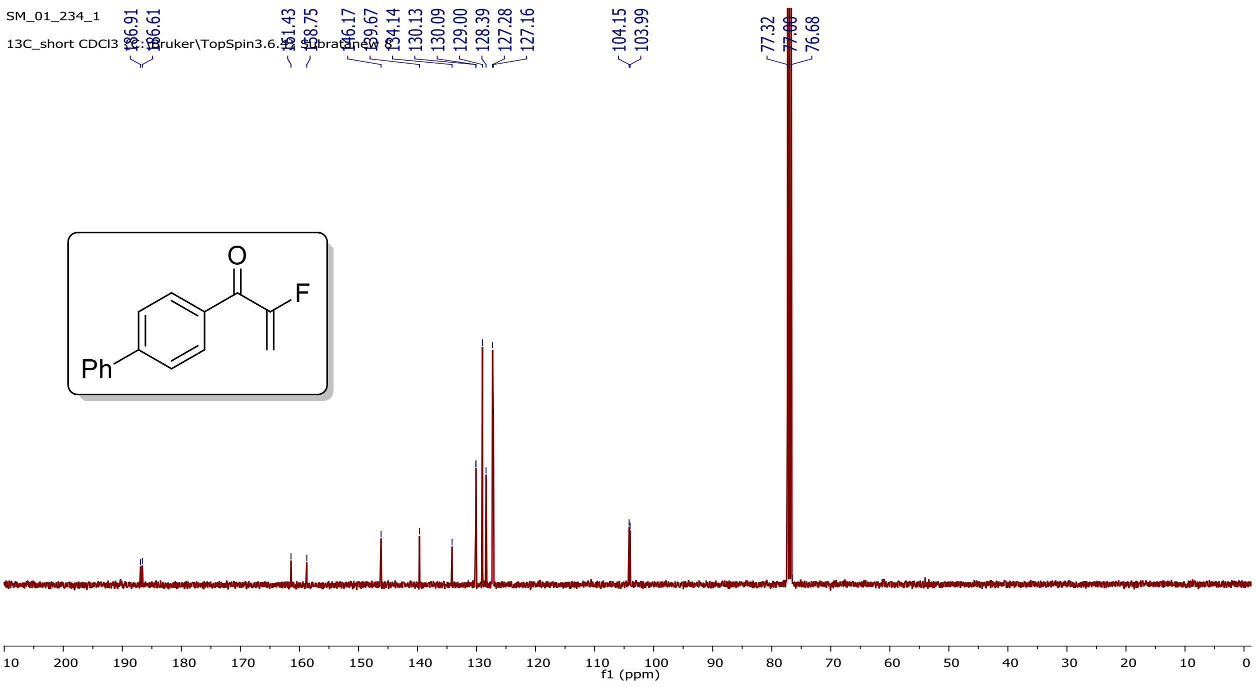

^{13}C NMR Spectrum of Compound **24** (101 MHz, $CDCl_3$)

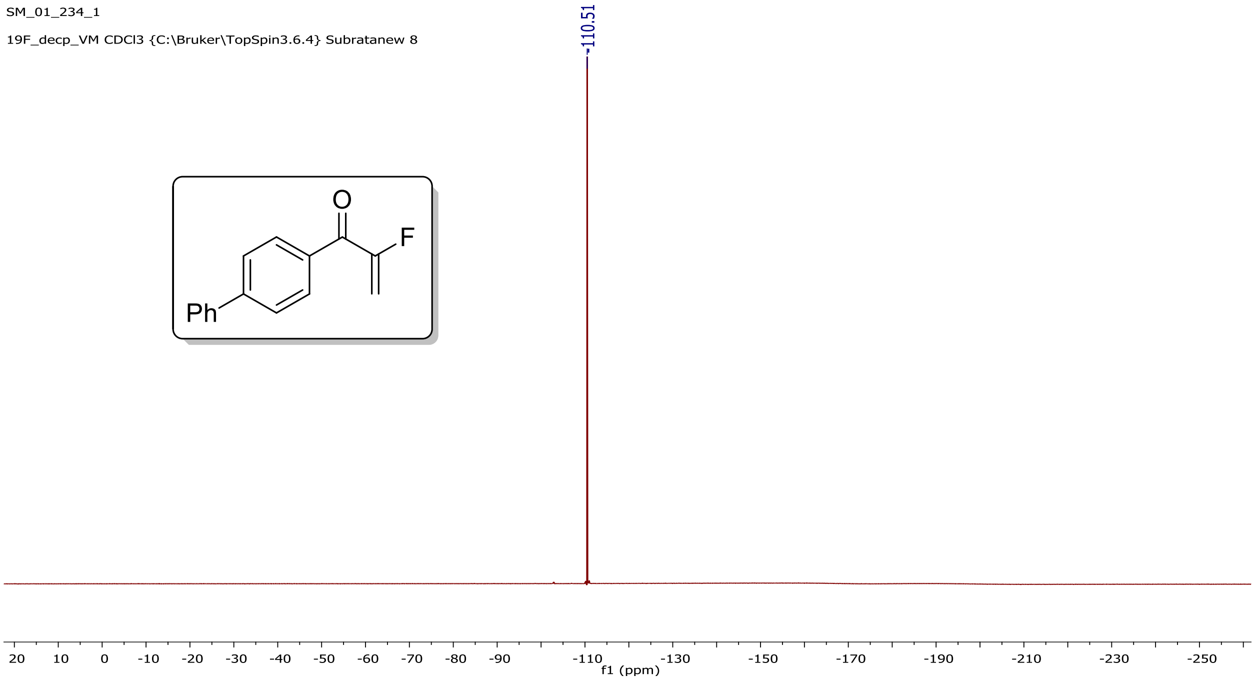

^{19}F NMR Spectrum of Compound **24** (376 MHz, $CDCl_3$)

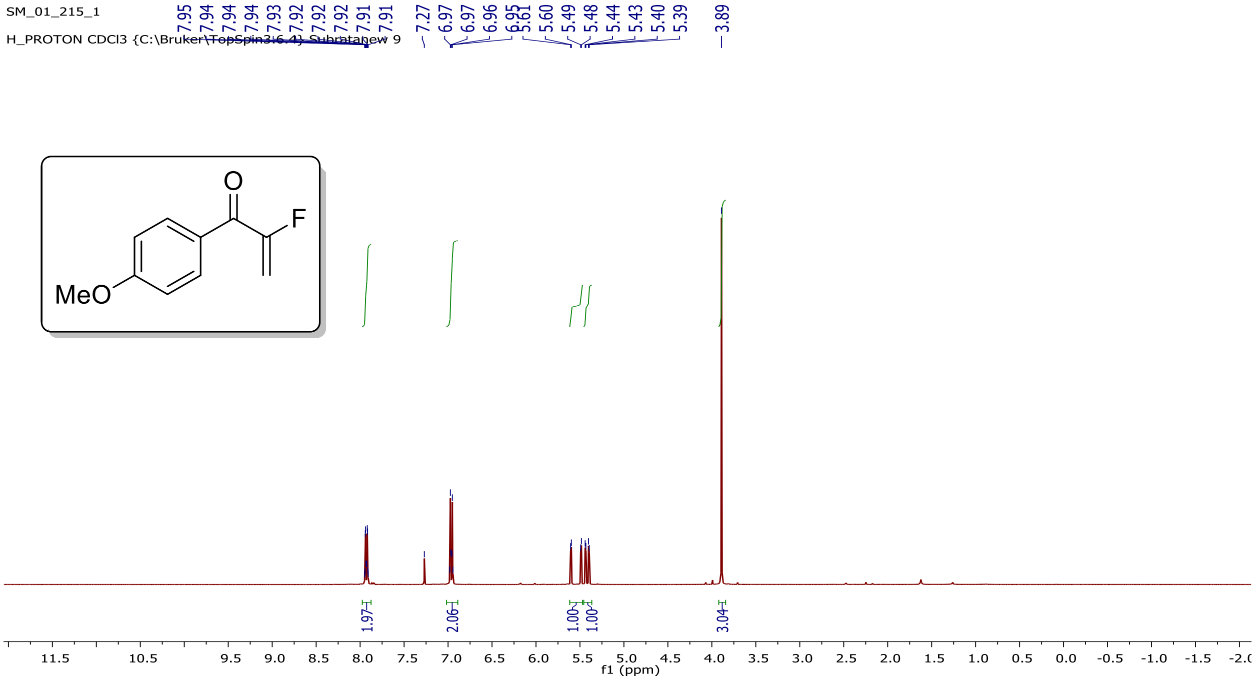

^{1}H NMR Spectrum of Compound **25** (400 MHz, $CDCl_3$)

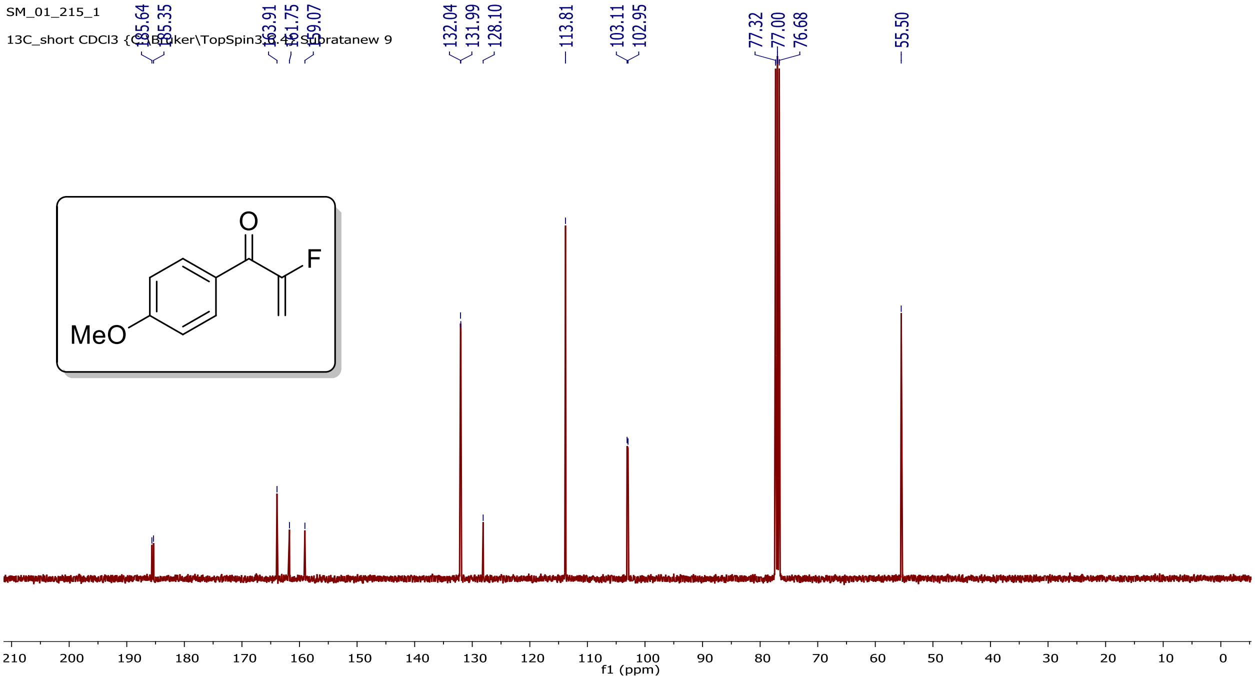

^{13}C NMR Spectrum of Compound **25** (101 MHz, $CDCl_3$)

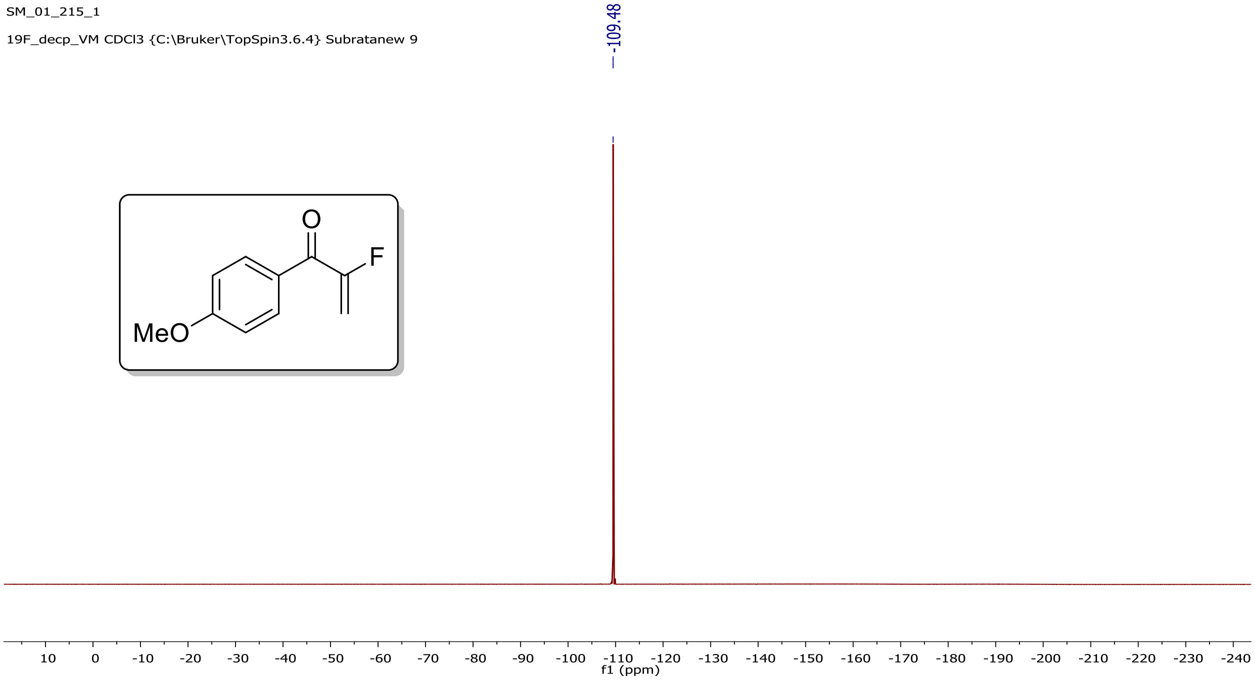

^{19}F NMR Spectrum of Compound **25** (376 MHz, $CDCl_3$)

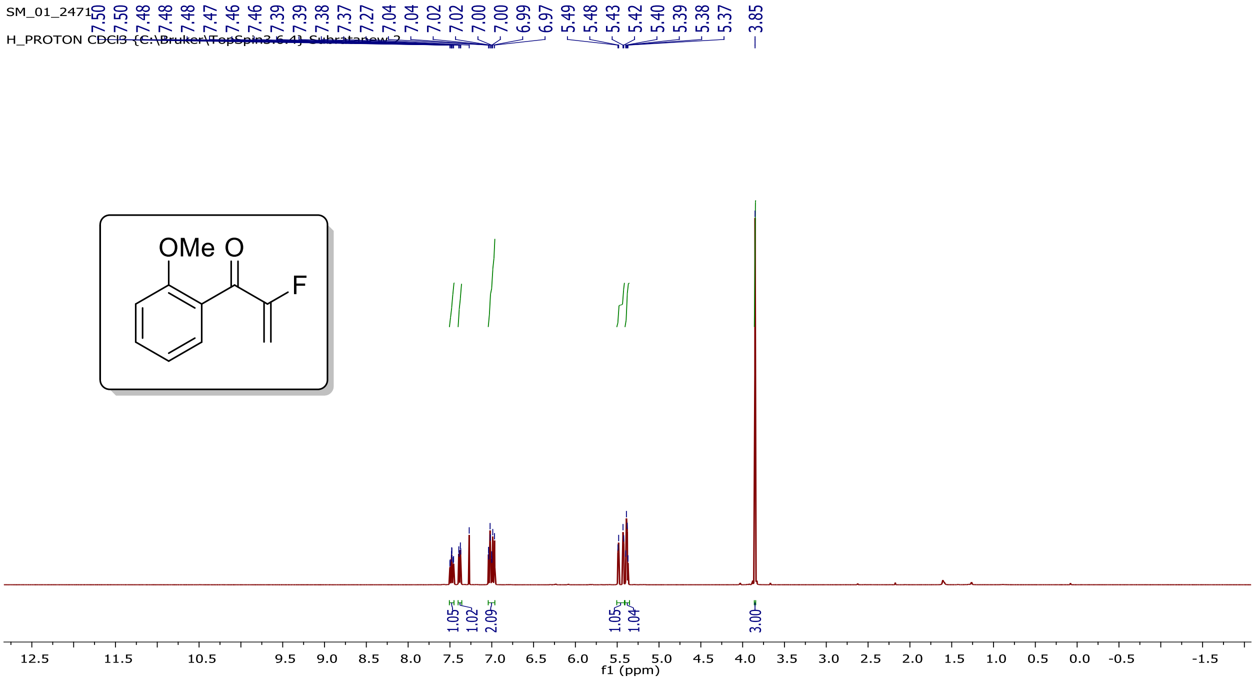

^{1}H NMR Spectrum of Compound **26** (400 MHz, $CDCl_3$)

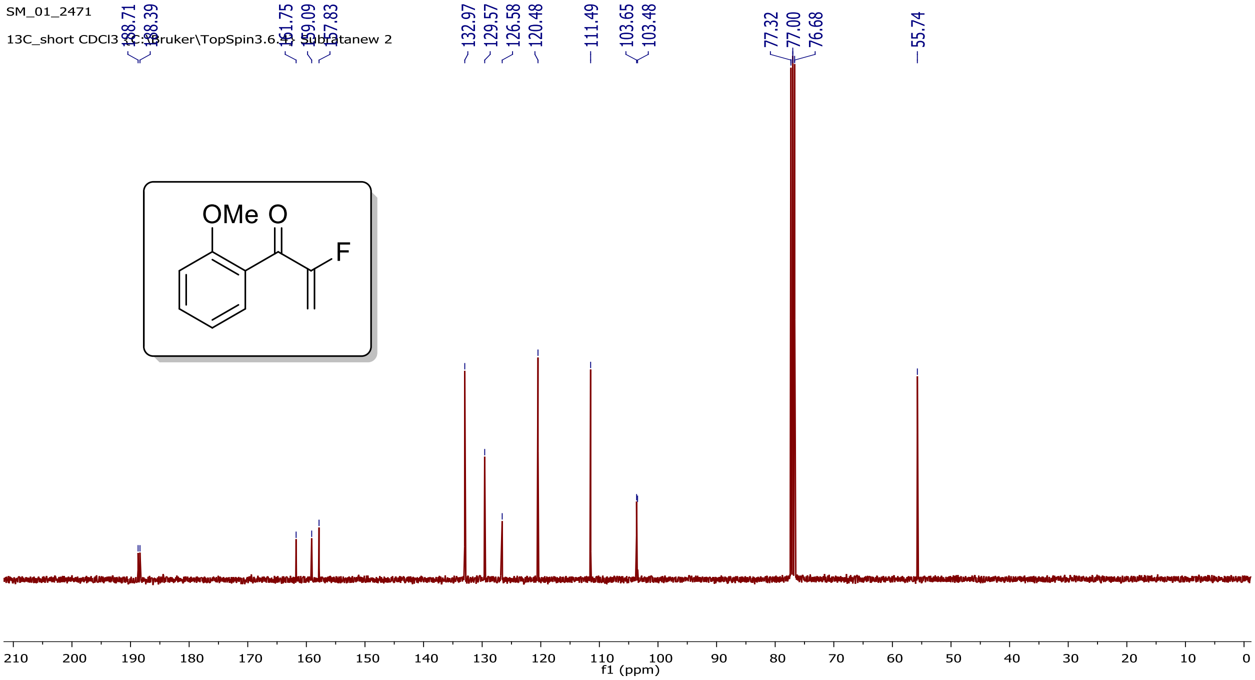

^{13}C NMR Spectrum of Compound **26** (101 MHz, $CDCl_3$)

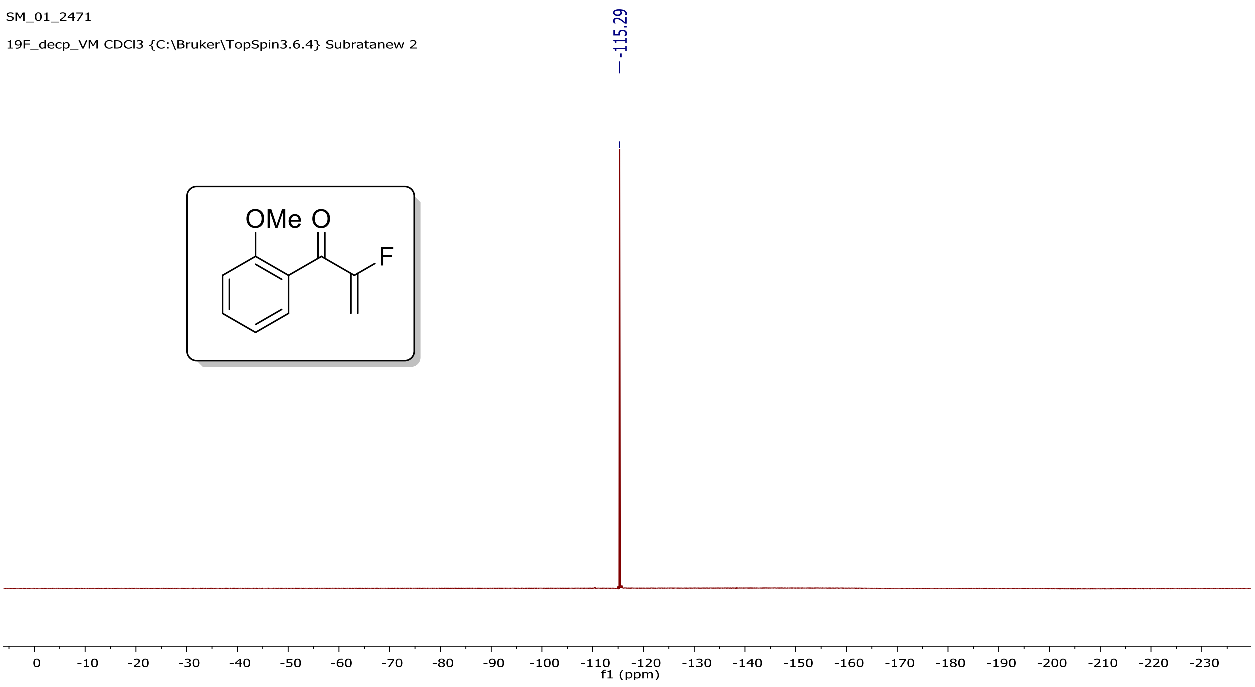

^{19}F NMR Spectrum of Compound **26** (376 MHz, $CDCl_3$)

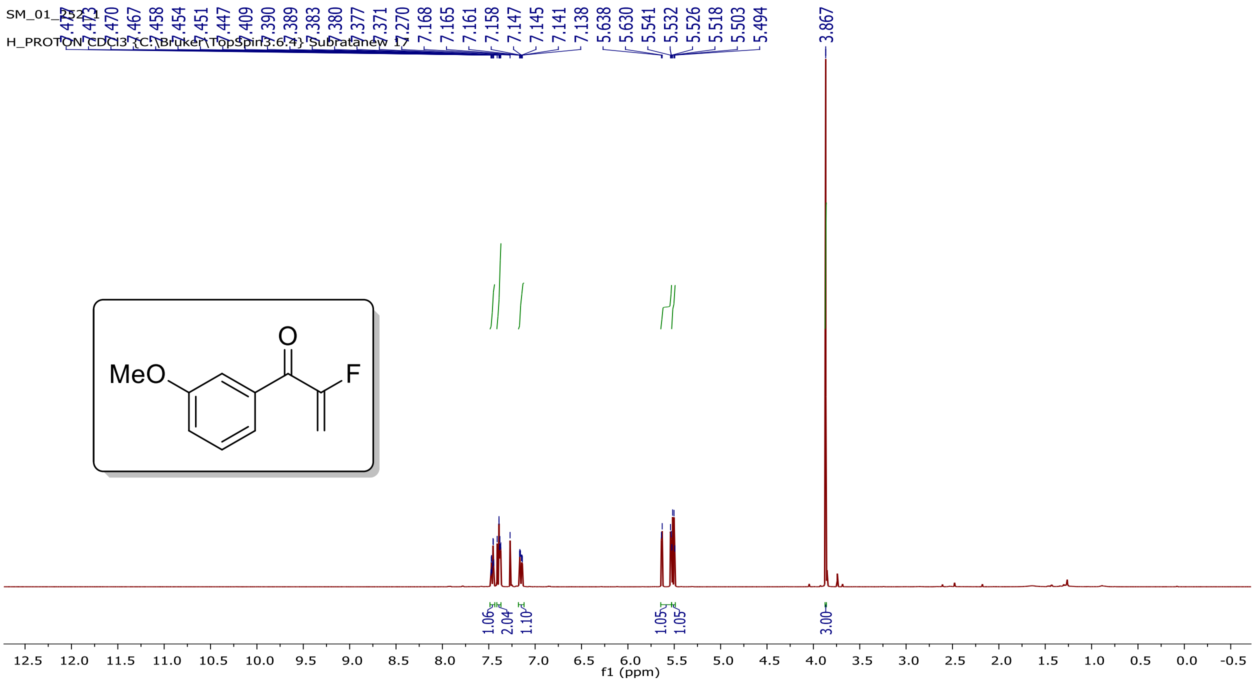

^{1}H NMR Spectrum of Compound **27** (400 MHz, $CDCl_3$)

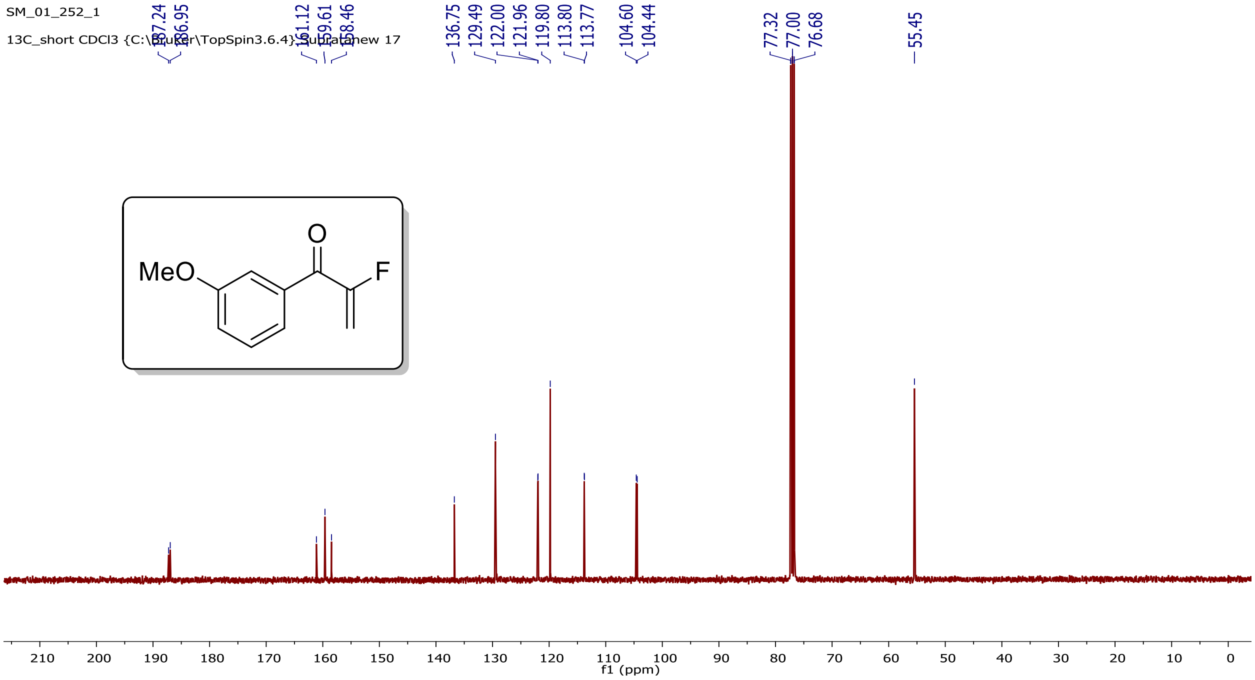

^{13}C NMR Spectrum of Compound **27** (101 MHz, $CDCl_3$)

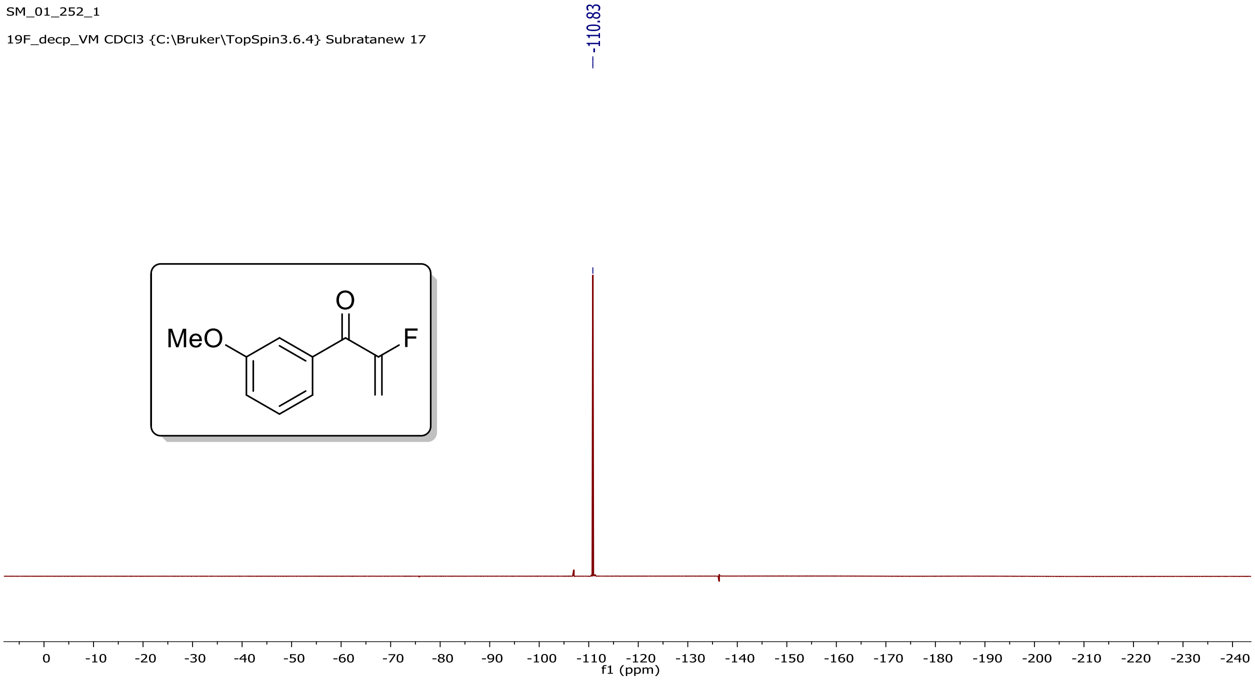

^{19}F NMR Spectrum of Compound **27** (376 MHz, $CDCl_3$)

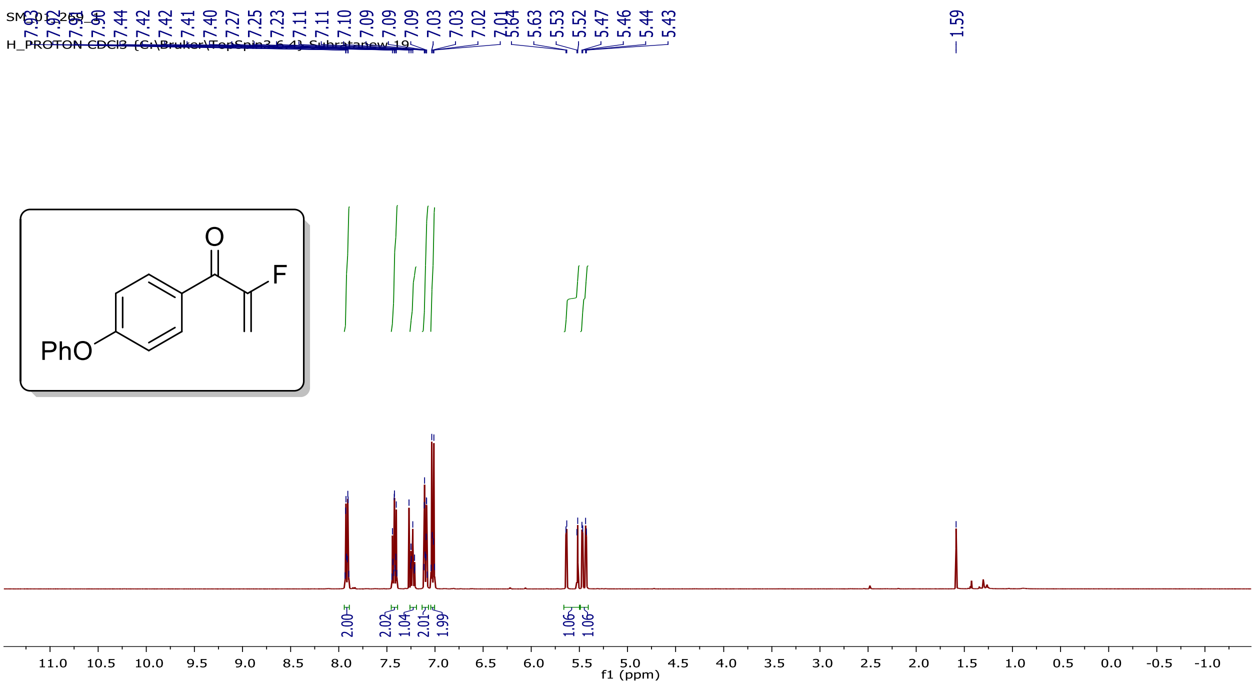

^{1}H NMR Spectrum of Compound **28** (400 MHz, $CDCl_3$)

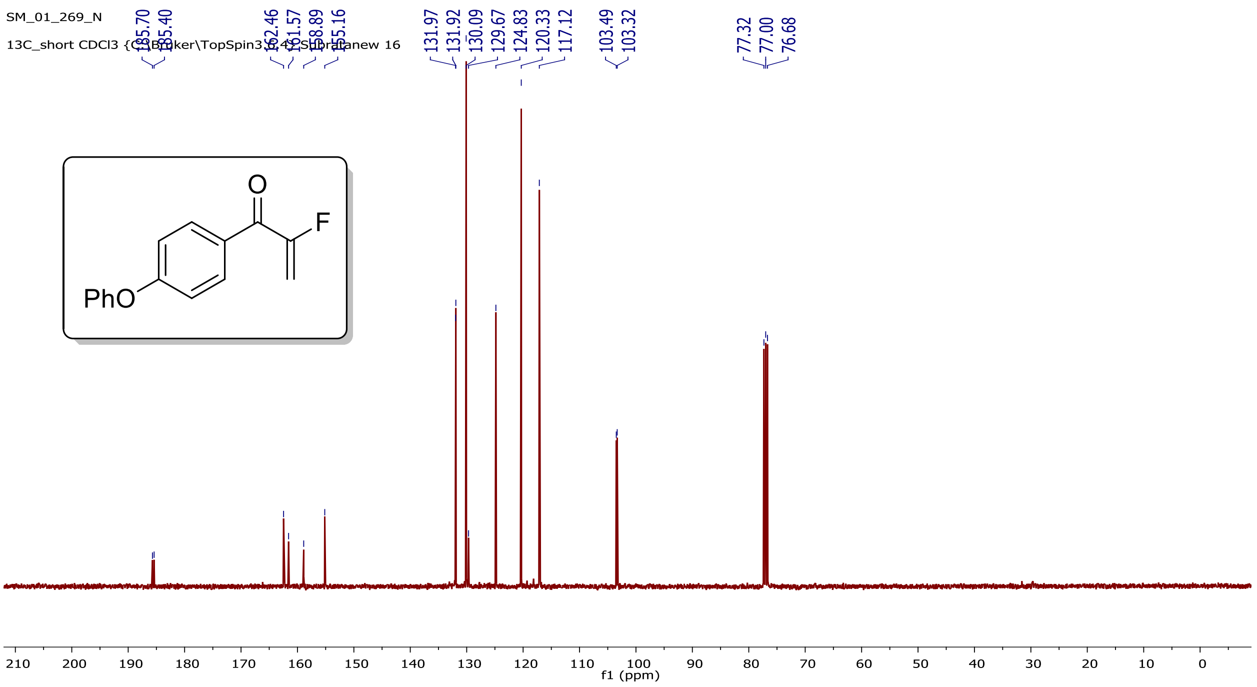

^{13}C NMR Spectrum of Compound **28** (101 MHz, $CDCl_3$)

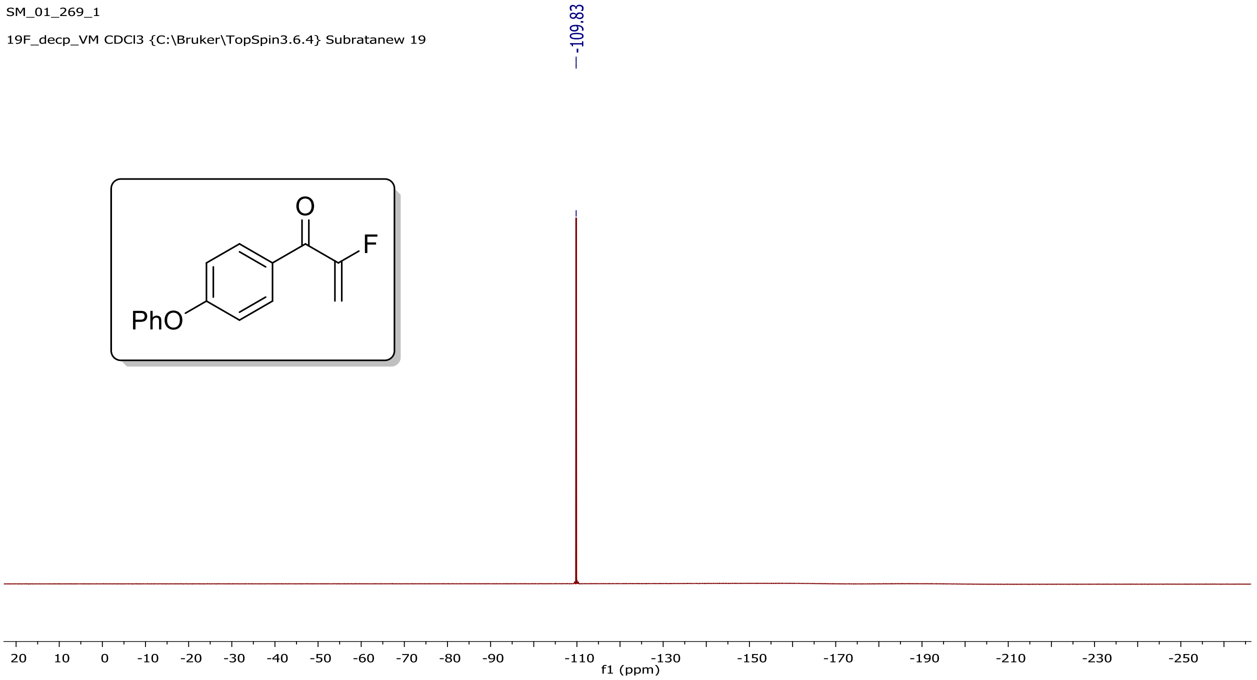

^{19}F NMR Spectrum of Compound **28** (376 MHz, $CDCl_3$)

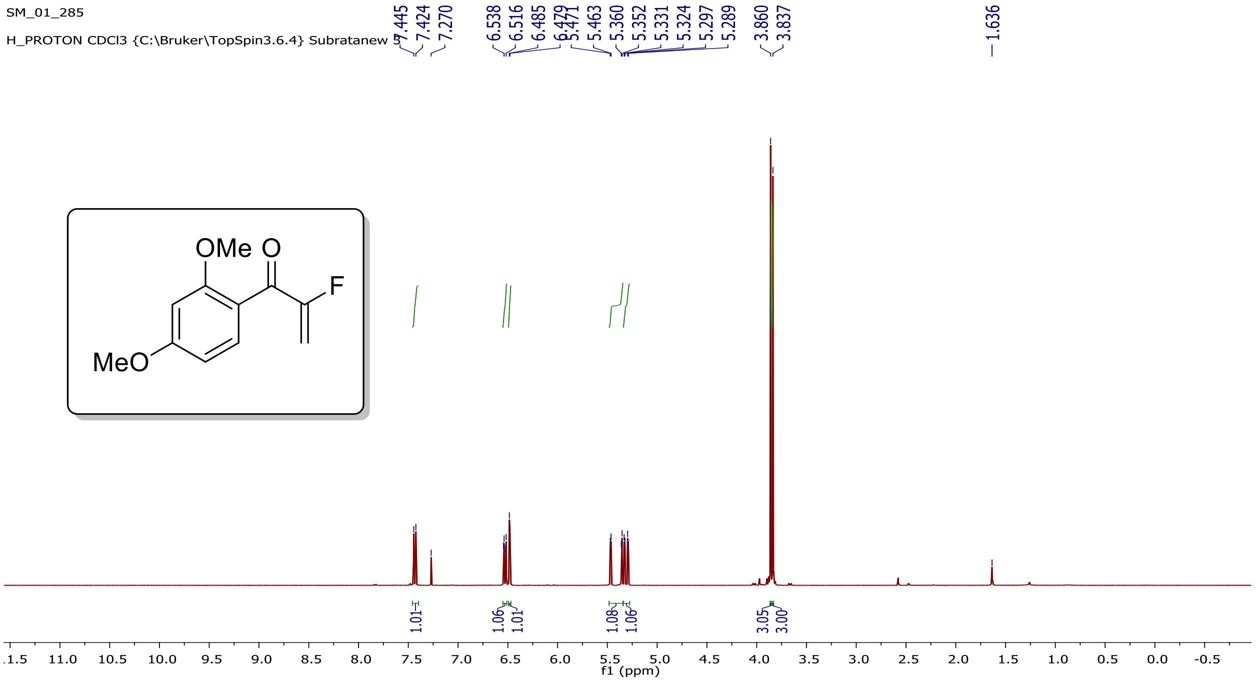

^{1}H NMR Spectrum of Compound **29** (400 MHz, $CDCl_3$)

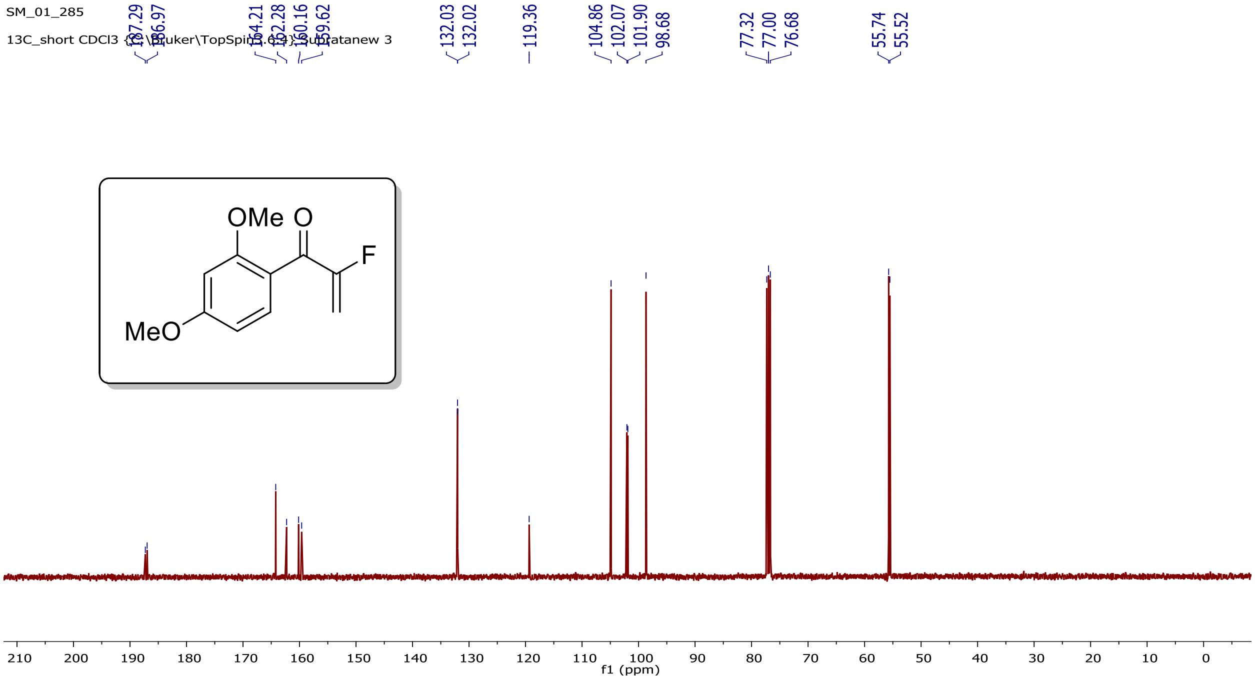

^{13}C NMR Spectrum of Compound **29** (101 MHz, $CDCl_3$)

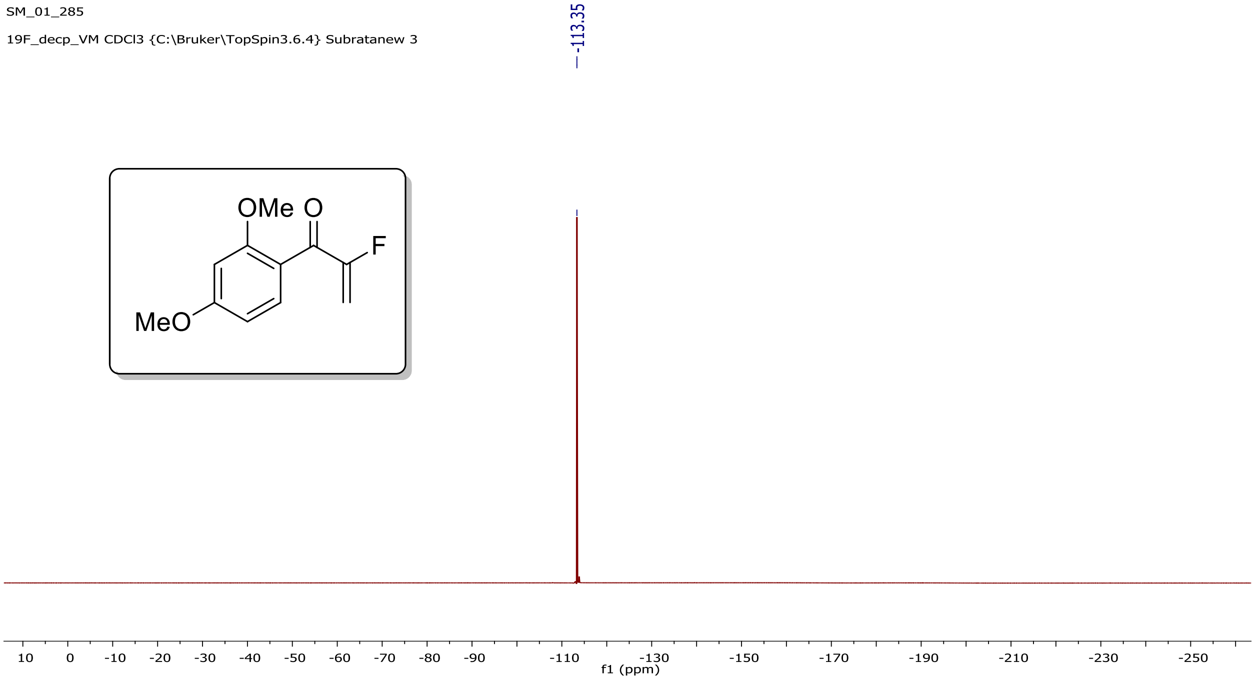

^{19}F NMR Spectrum of Compound **29** (376 MHz, $CDCl_3$)

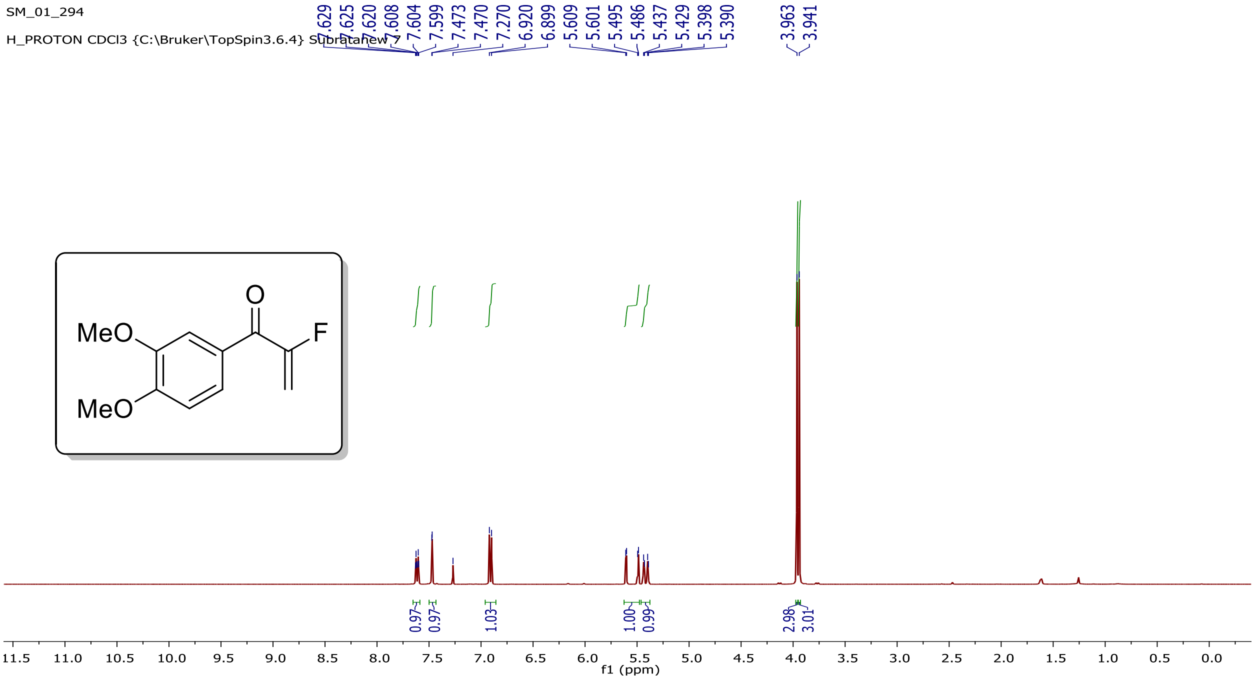

^{1}H NMR Spectrum of Compound **30** (400 MHz, $CDCl_3$)

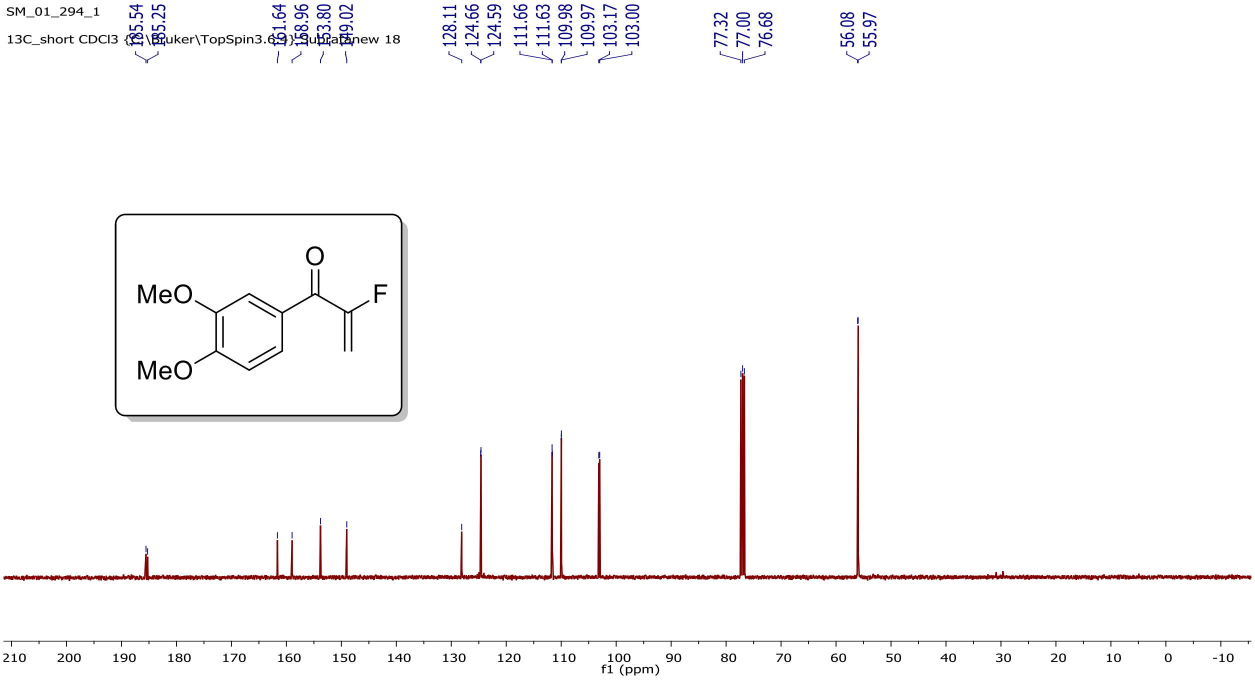

^{13}C NMR Spectrum of Compound **30** (101 MHz, $CDCl_3$)

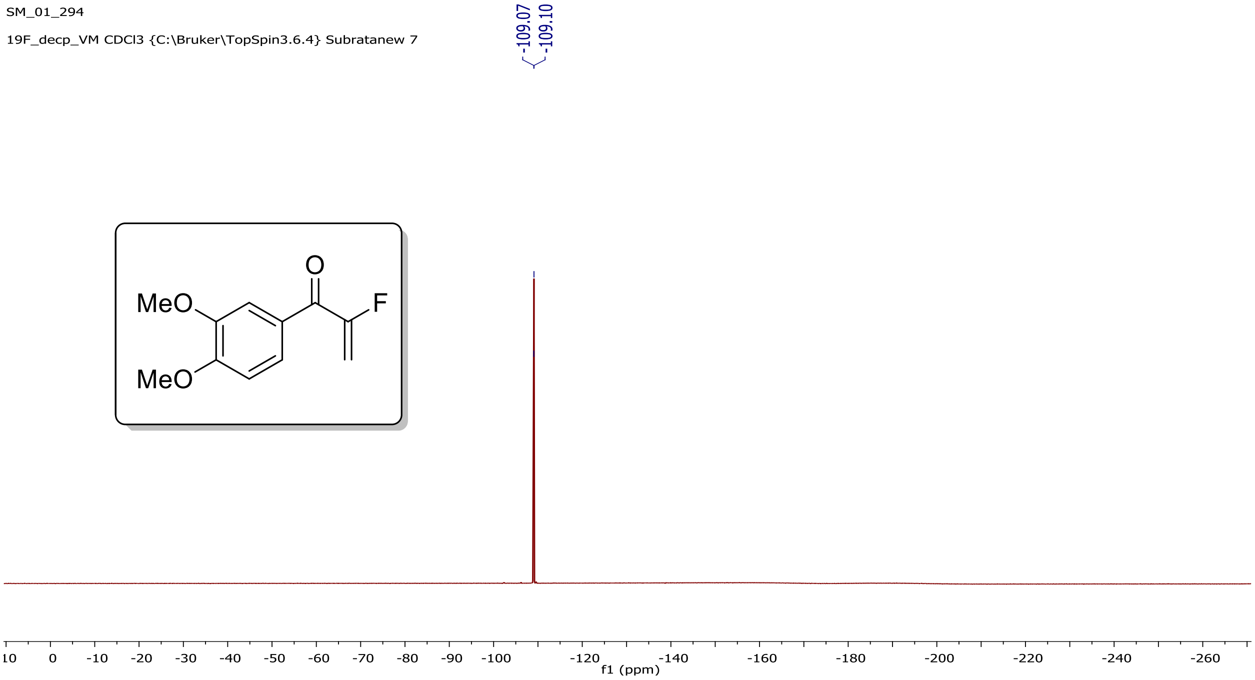

^{19}F NMR Spectrum of Compound **30** (376 MHz, $CDCl_3$)

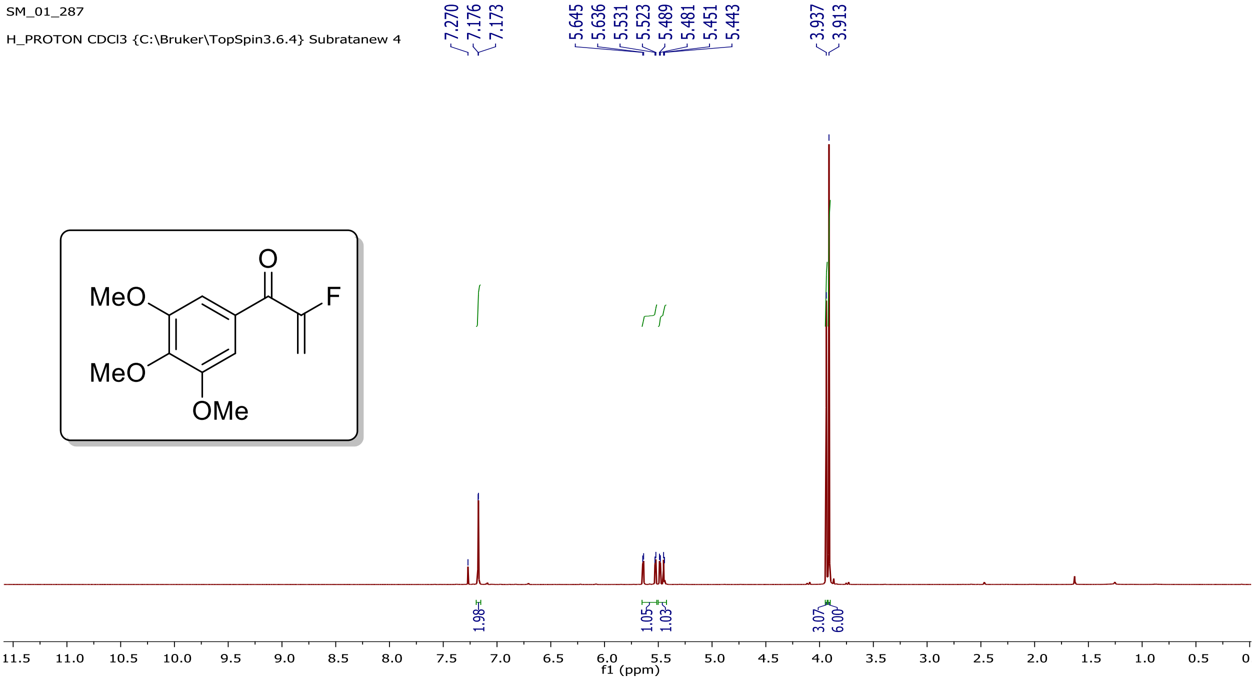

^{1}H NMR Spectrum of Compound **31** (400 MHz, $CDCl_3$)

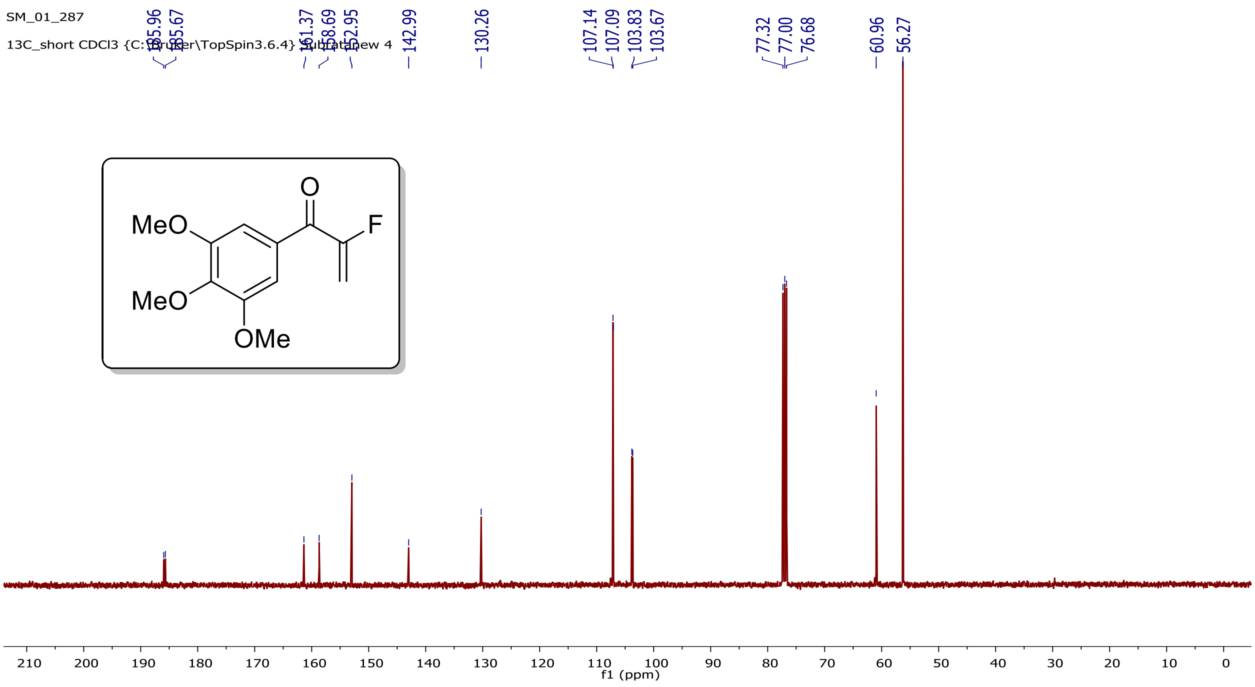

^{13}C NMR Spectrum of Compound **31** (101 MHz, $CDCl_3$)

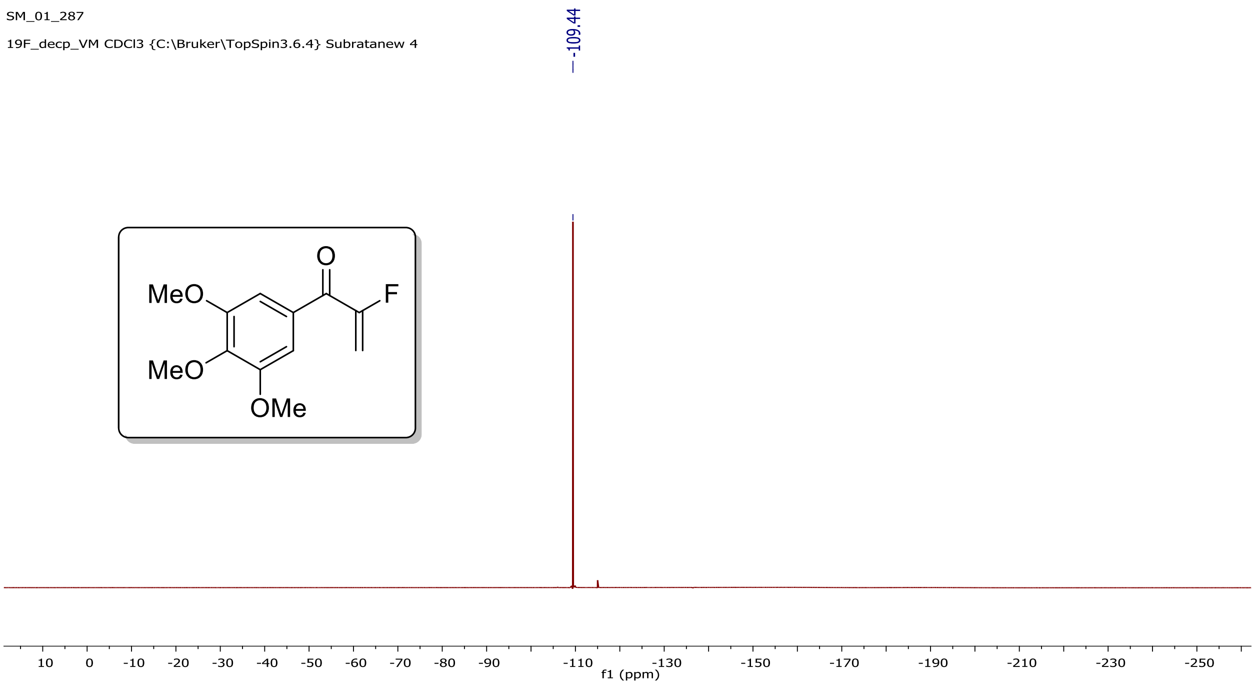

^{19}F NMR Spectrum of Compound **31** (376 MHz, $CDCl_3$)

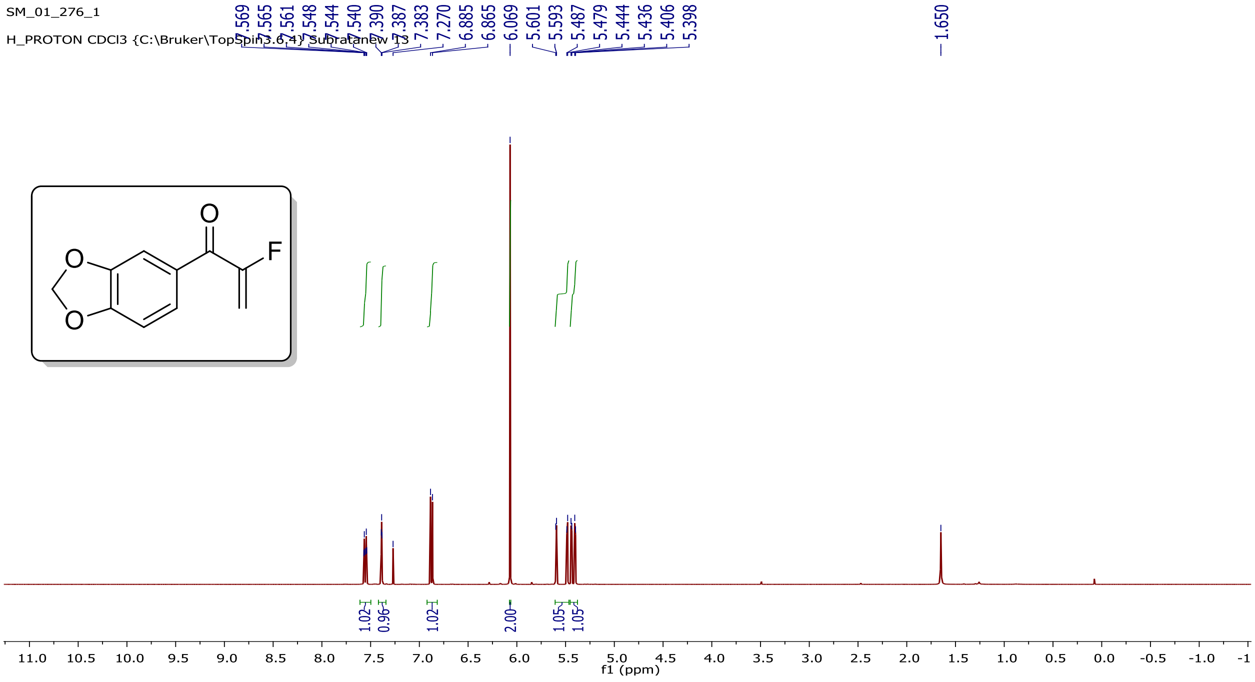

^{1}H NMR Spectrum of Compound **32** (400 MHz, $CDCl_3$)

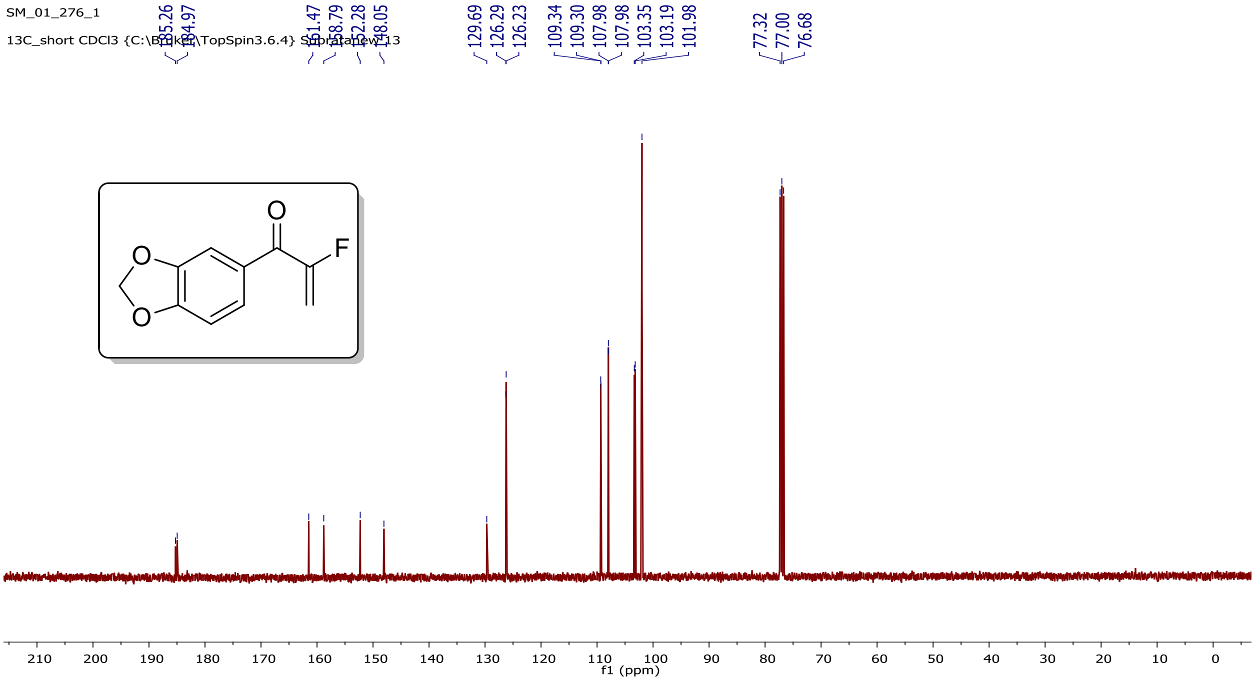

^{13}C NMR Spectrum of Compound **32** (101 MHz, $CDCl_3$)

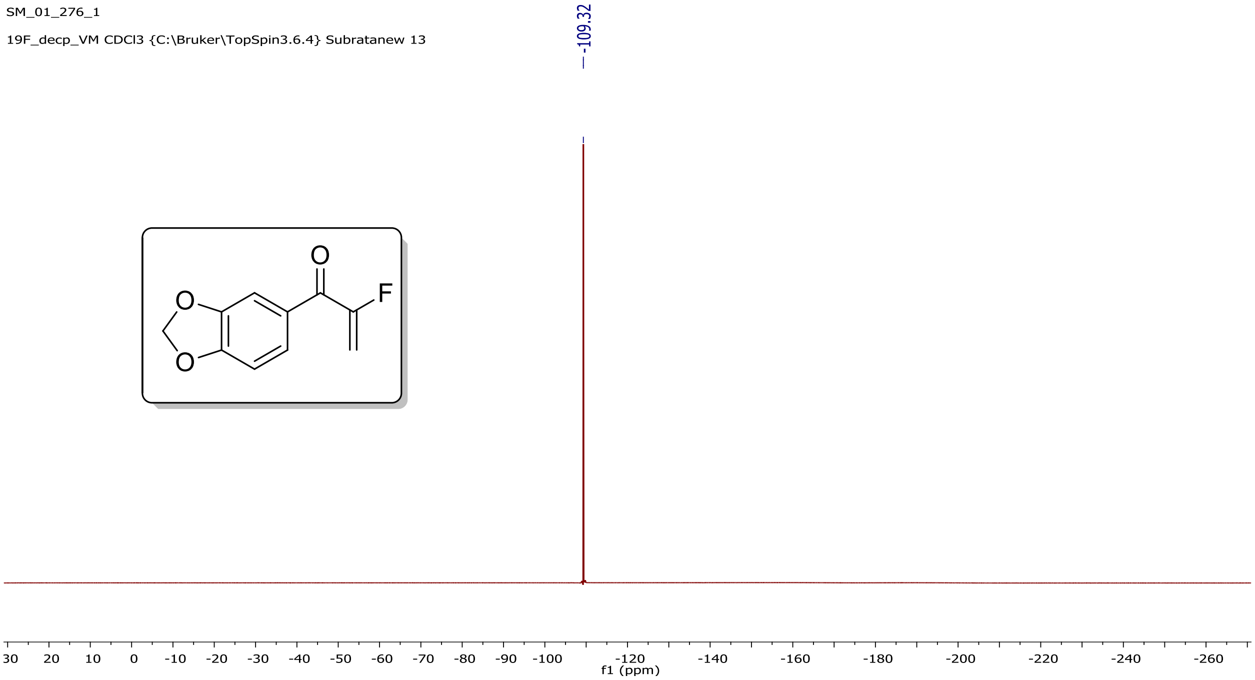

^{19}F NMR Spectrum of Compound **32** (376 MHz, $CDCl_3$)

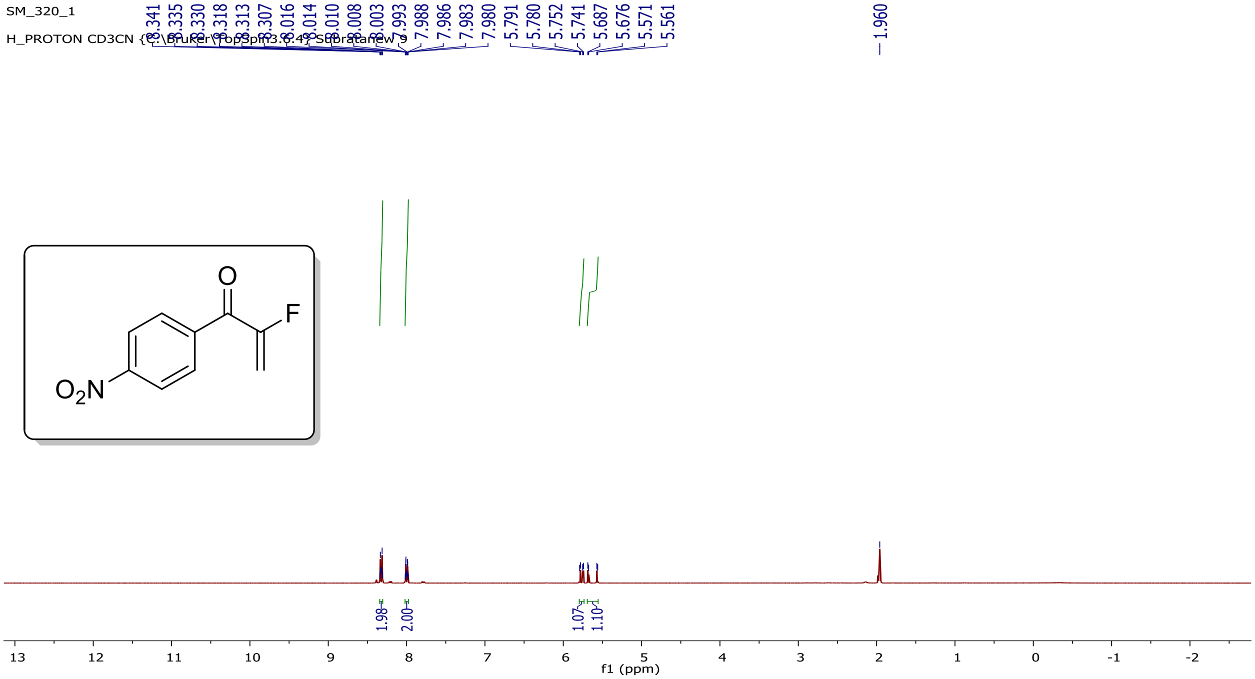

^{1}H NMR Spectrum of Compound **33** (400 MHz, CD_3CN)

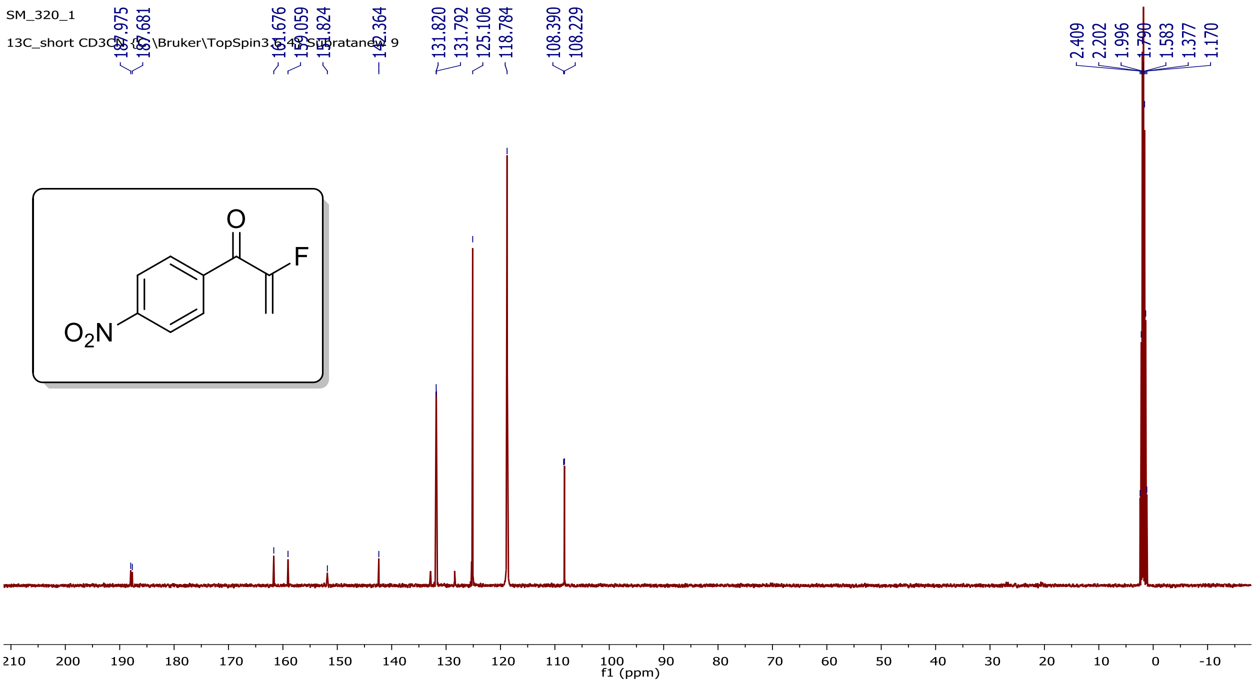

^{13}C NMR Spectrum of Compound **33** (101 MHz, CD_3CN)

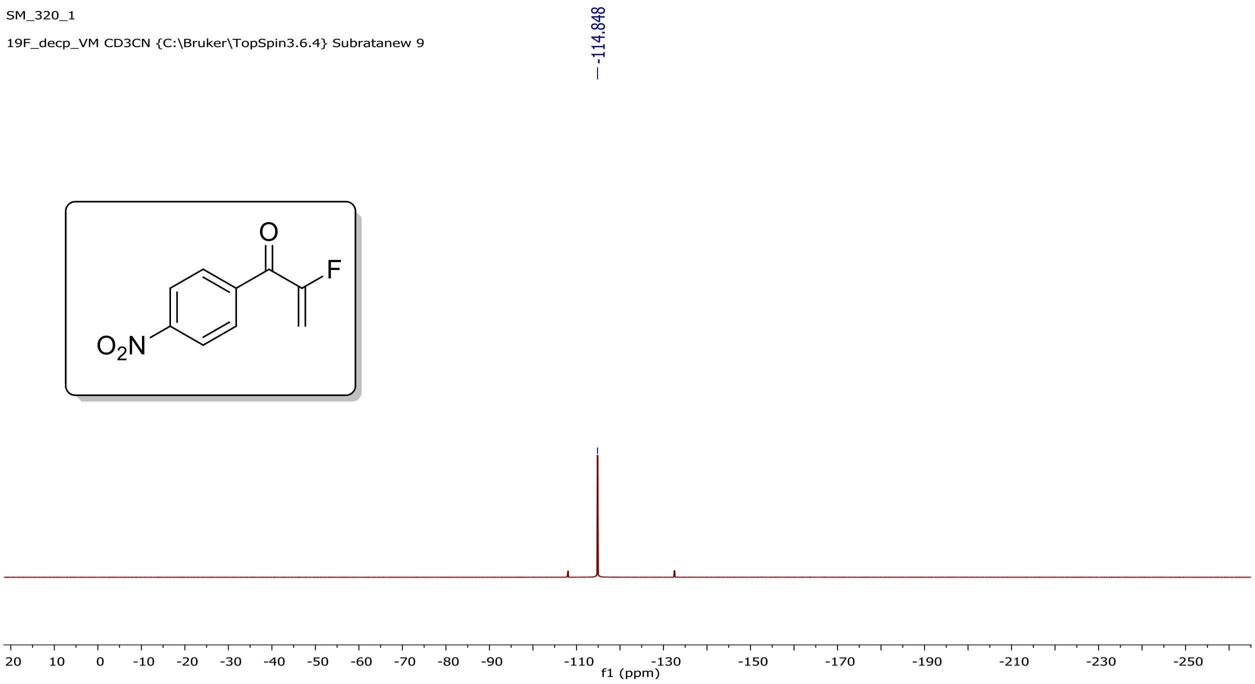

^{19}F NMR Spectrum of Compound **33** (376 MHz, CD_3CN)

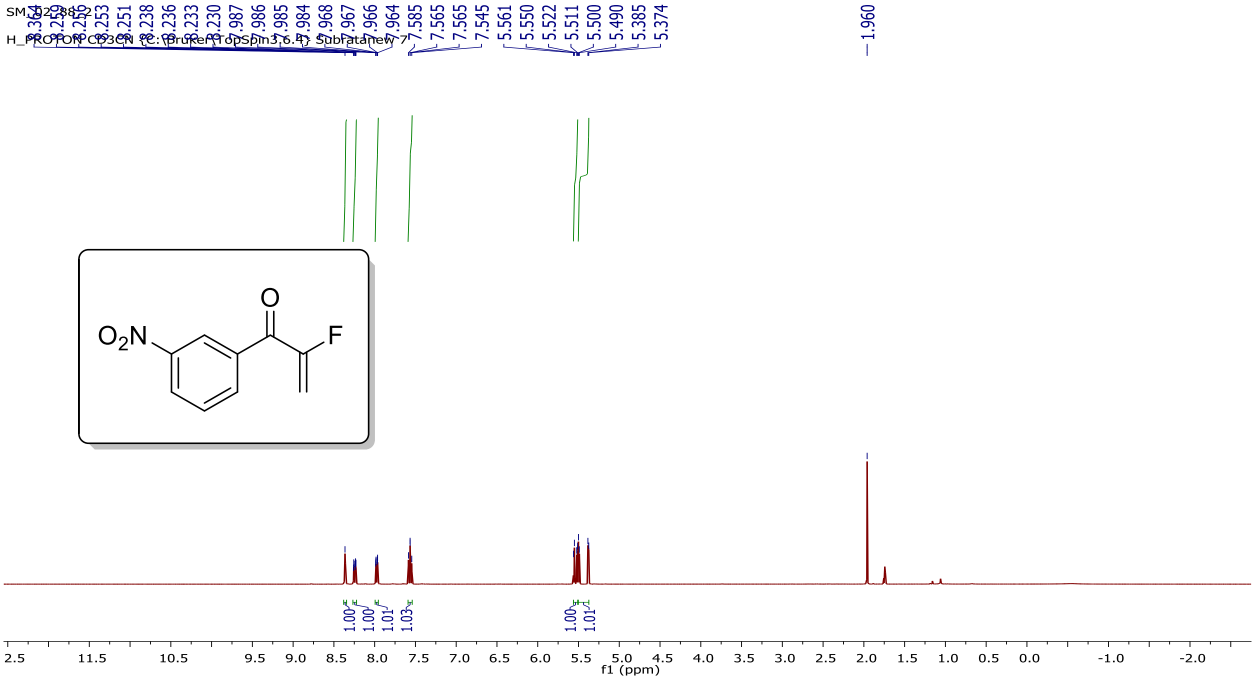

^{1}H NMR Spectrum of Compound **34** (400 MHz, CD_3CN)

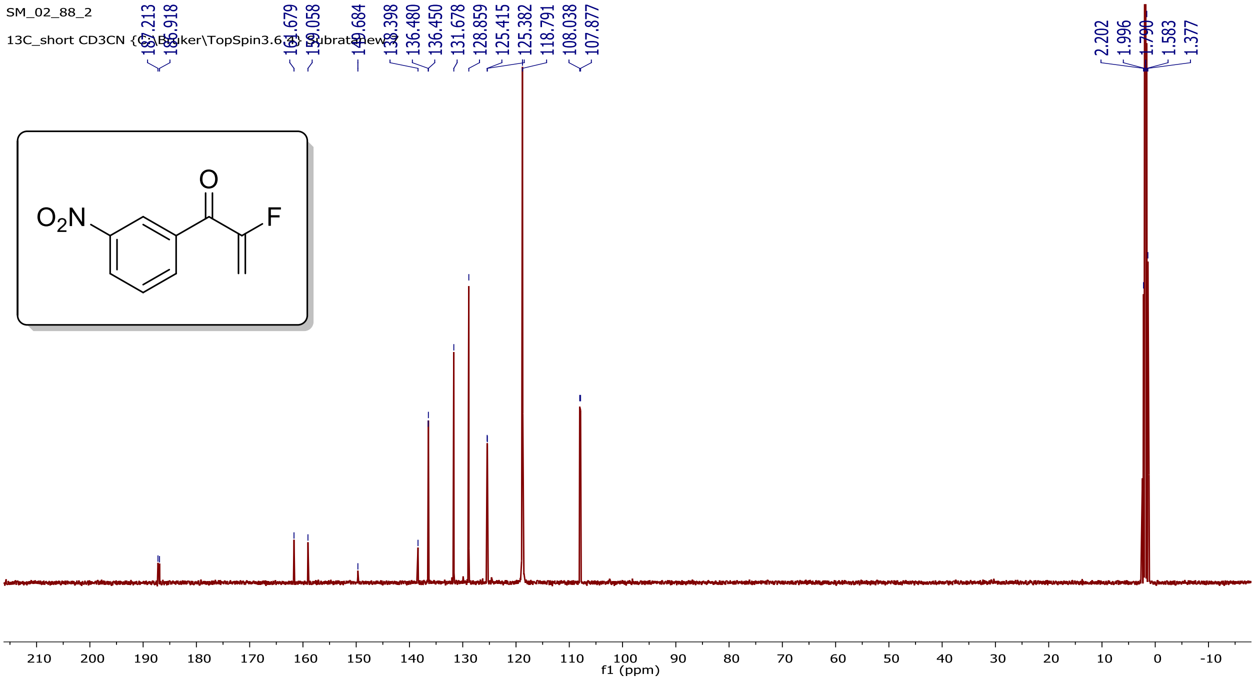

^{13}C NMR Spectrum of Compound **34** (101 MHz, CD_3CN)

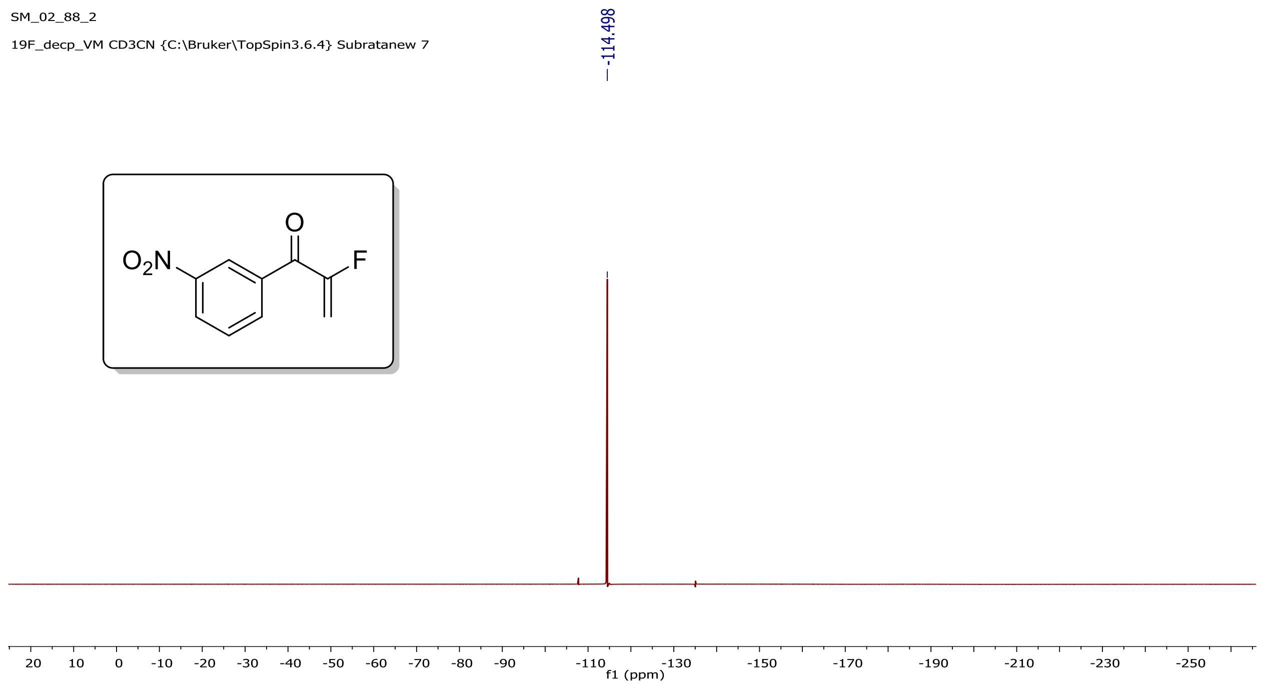

^{19}F NMR Spectrum of Compound **34** (376 MHz, CD_3CN)

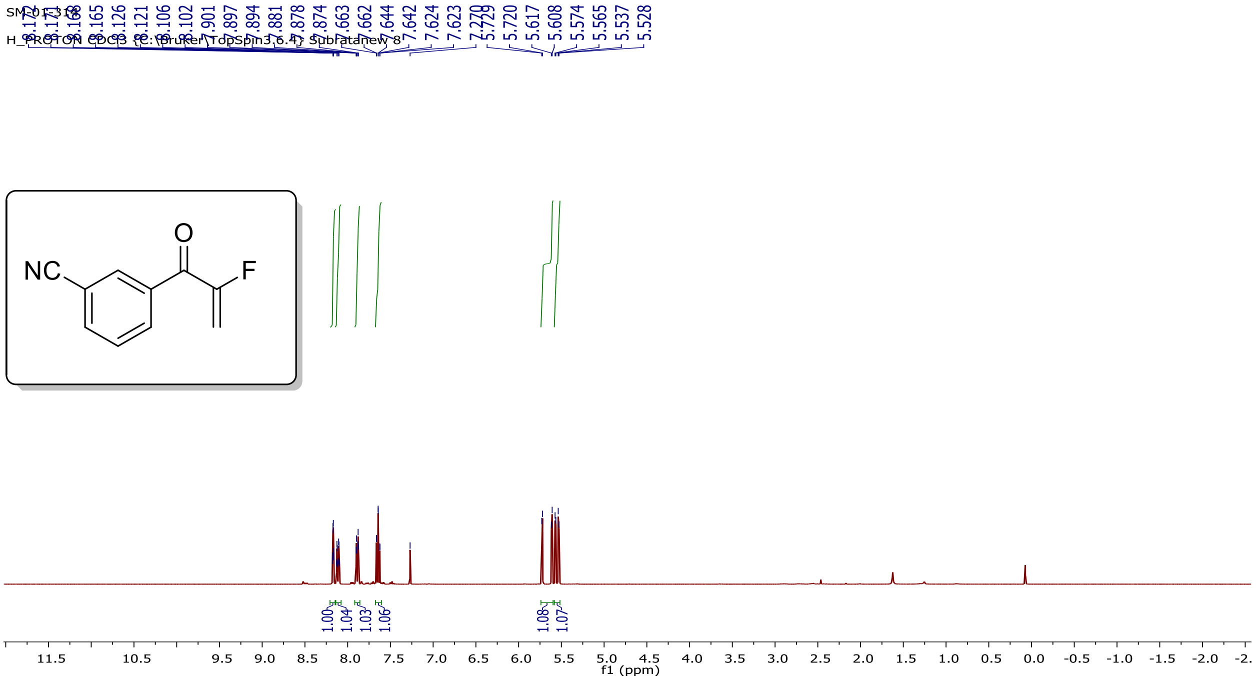

^{1}H NMR Spectrum of Compound **35** (400 MHz, $CDCl_3$)

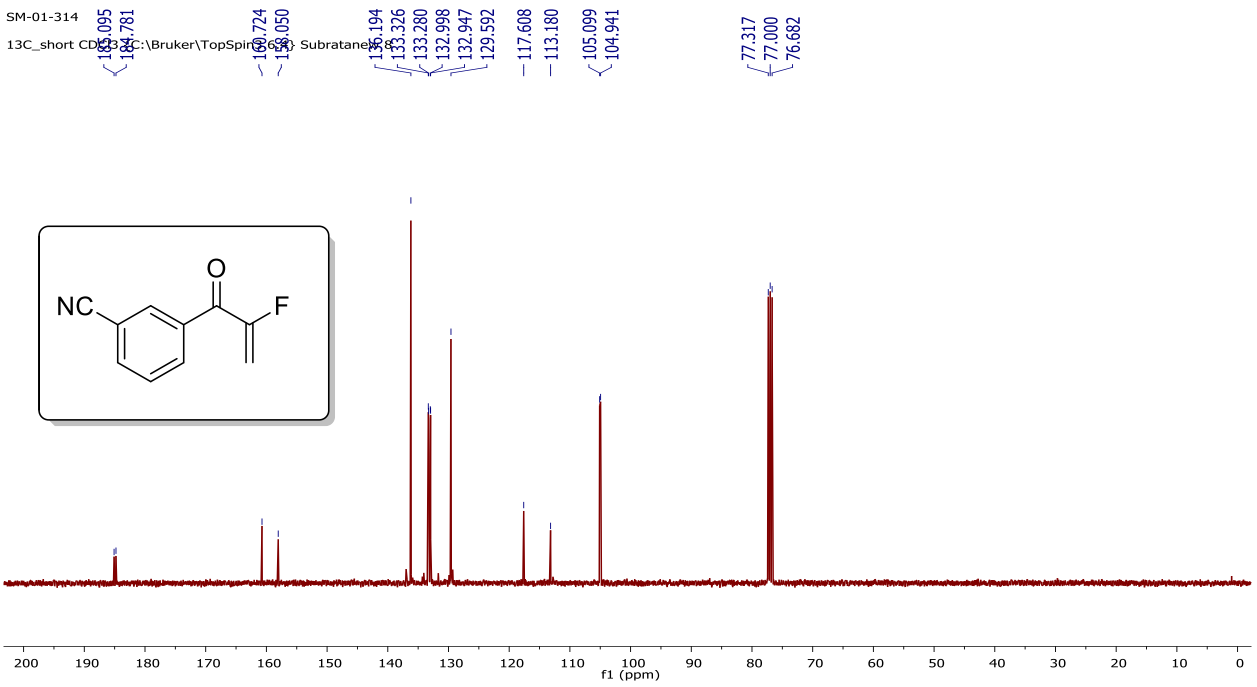

^{13}C NMR Spectrum of Compound **35** (101 MHz, $CDCl_3$)

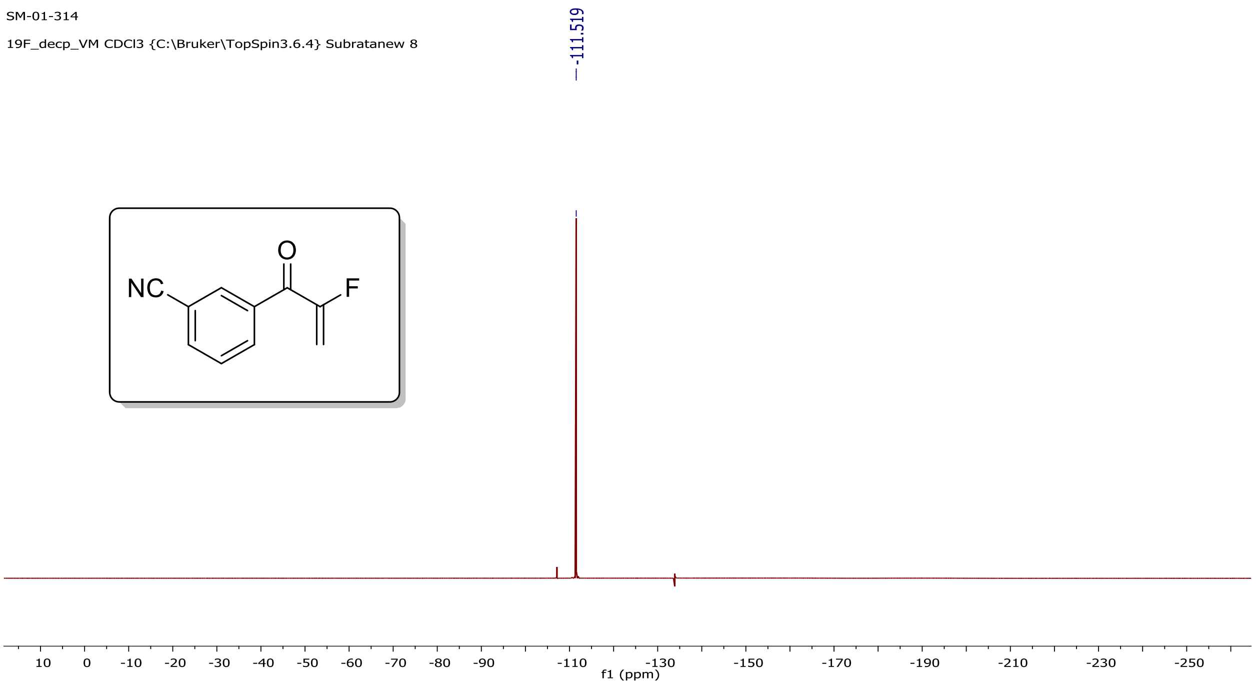

^{19}F NMR Spectrum of Compound **35** (376 MHz, $CDCl_3$)

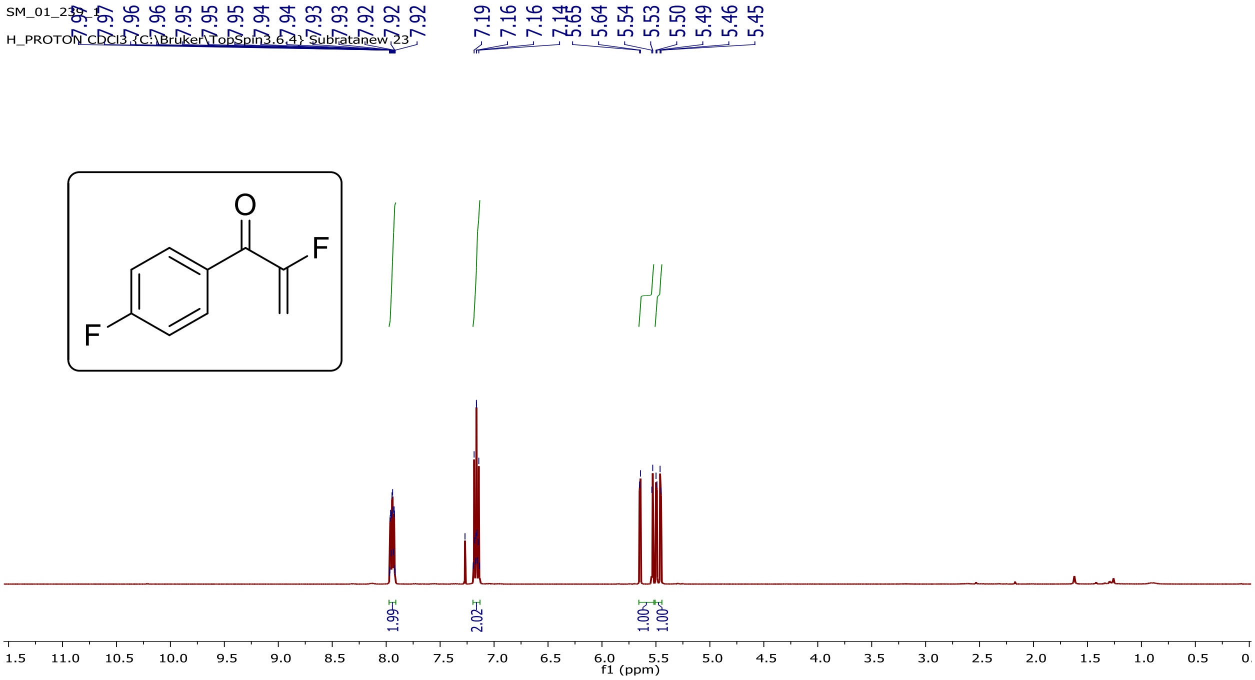

^{1}H NMR Spectrum of Compound **36** (400 MHz, $CDCl_3$)

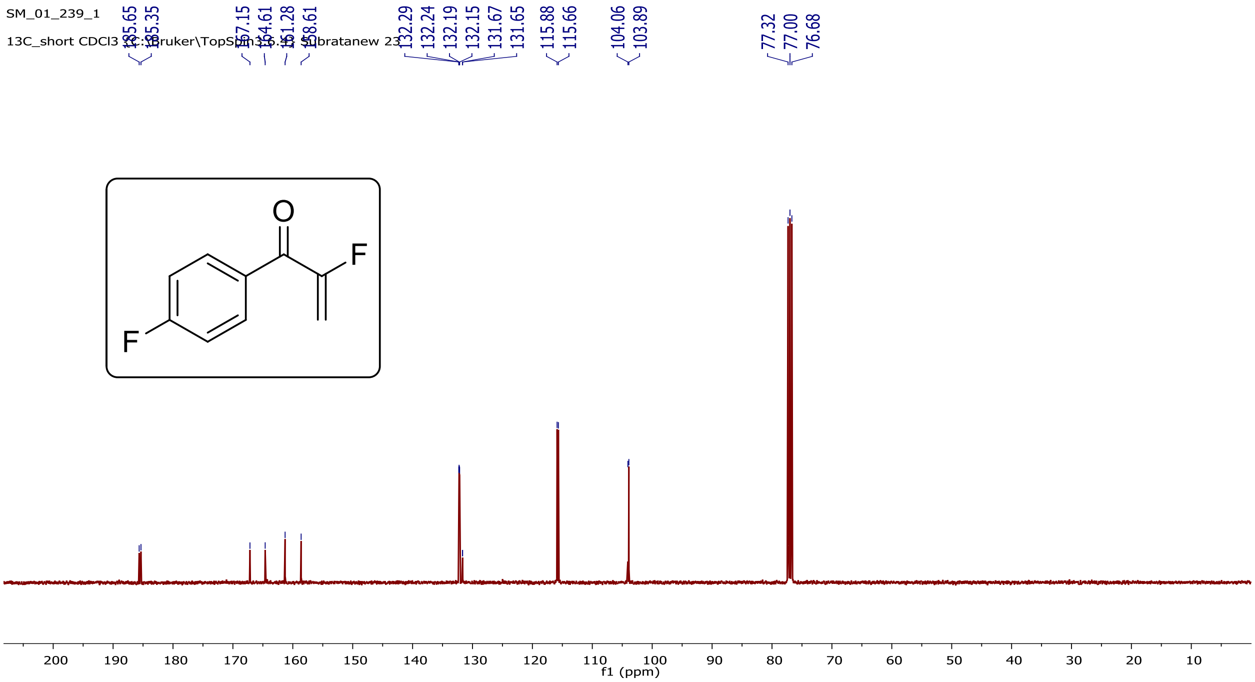

^{13}C NMR Spectrum of Compound **36** (101 MHz, $CDCl_3$)

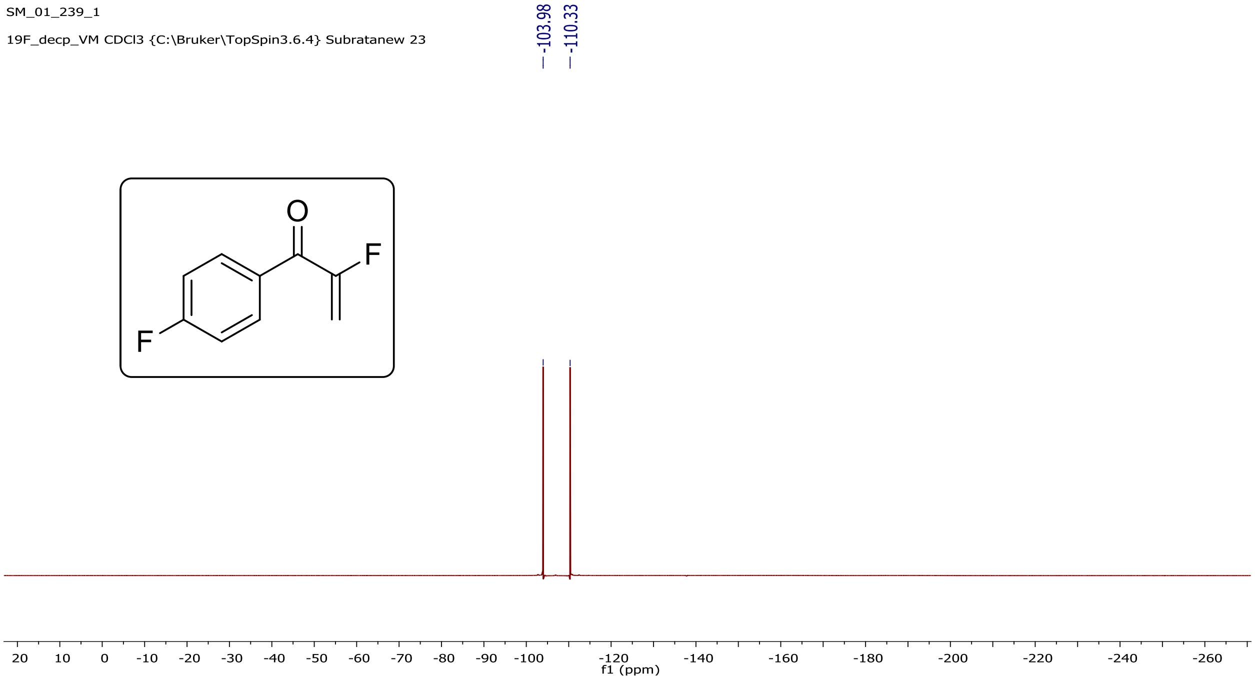

^{19}F NMR Spectrum of Compound **36** (376 MHz, $CDCl_3$)

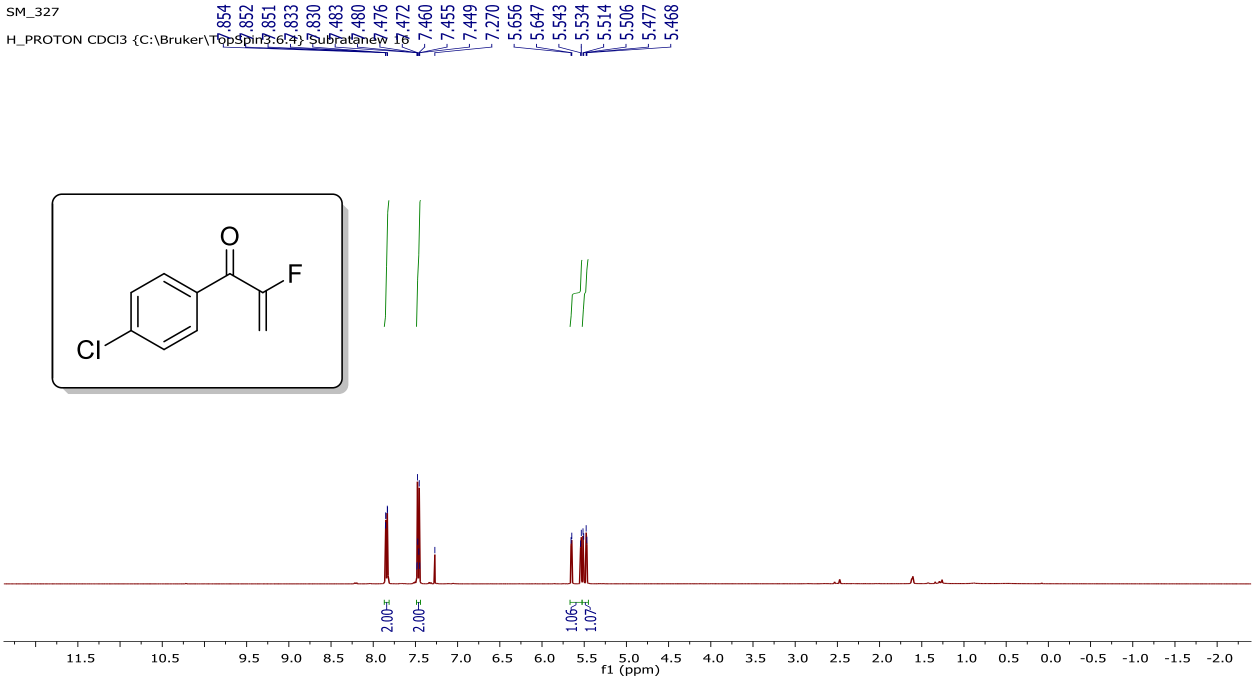

^{1}H NMR Spectrum of Compound **37** (400 MHz, $CDCl_3$)

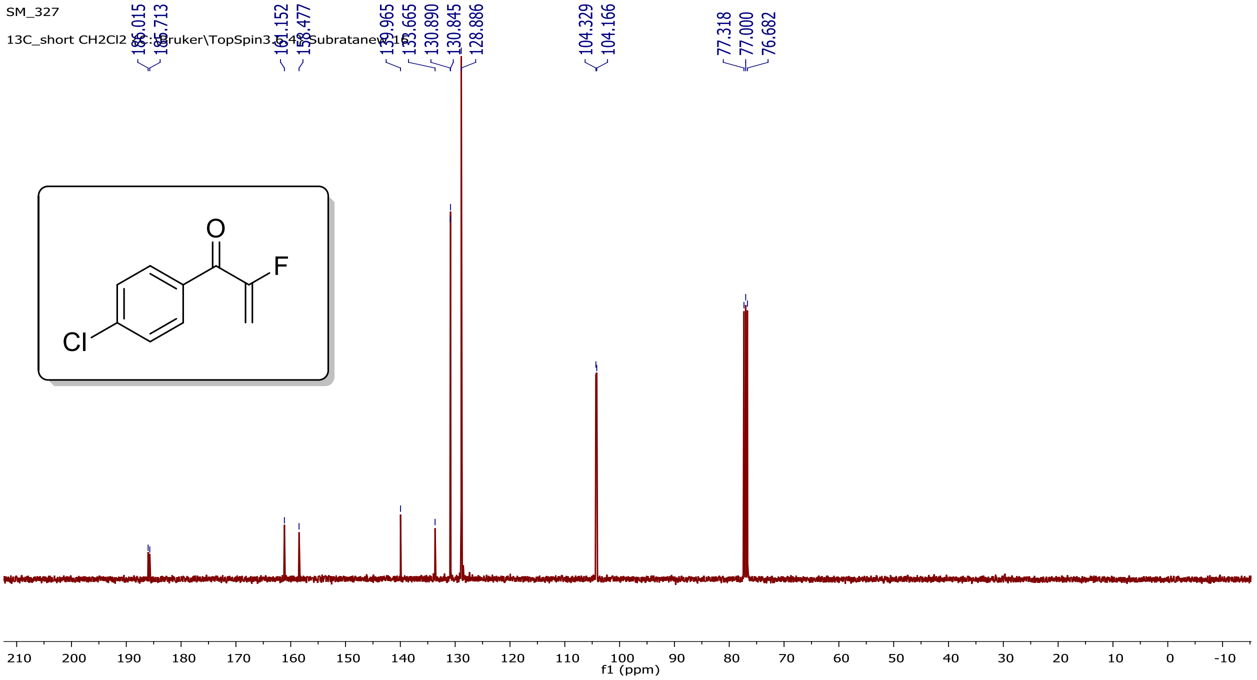

^{13}C NMR Spectrum of Compound **37** (101 MHz, $CDCl_3$)

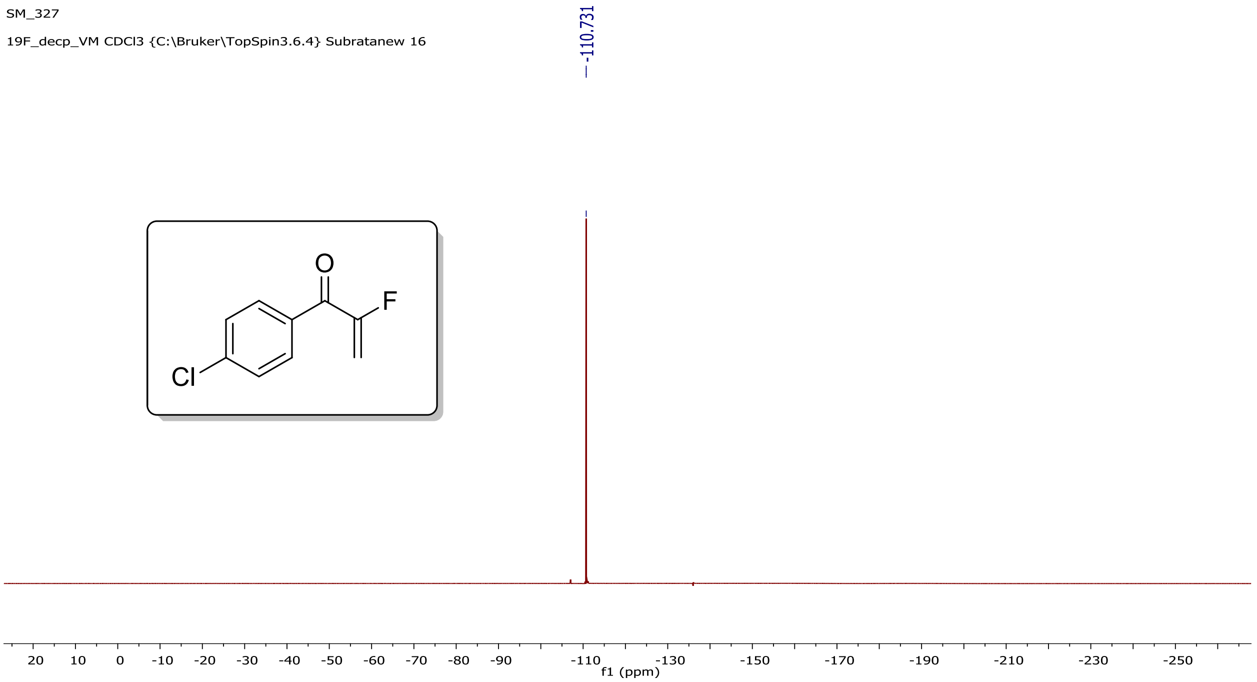

^{19}F NMR Spectrum of Compound **37** (376 MHz, $CDCl_3$)

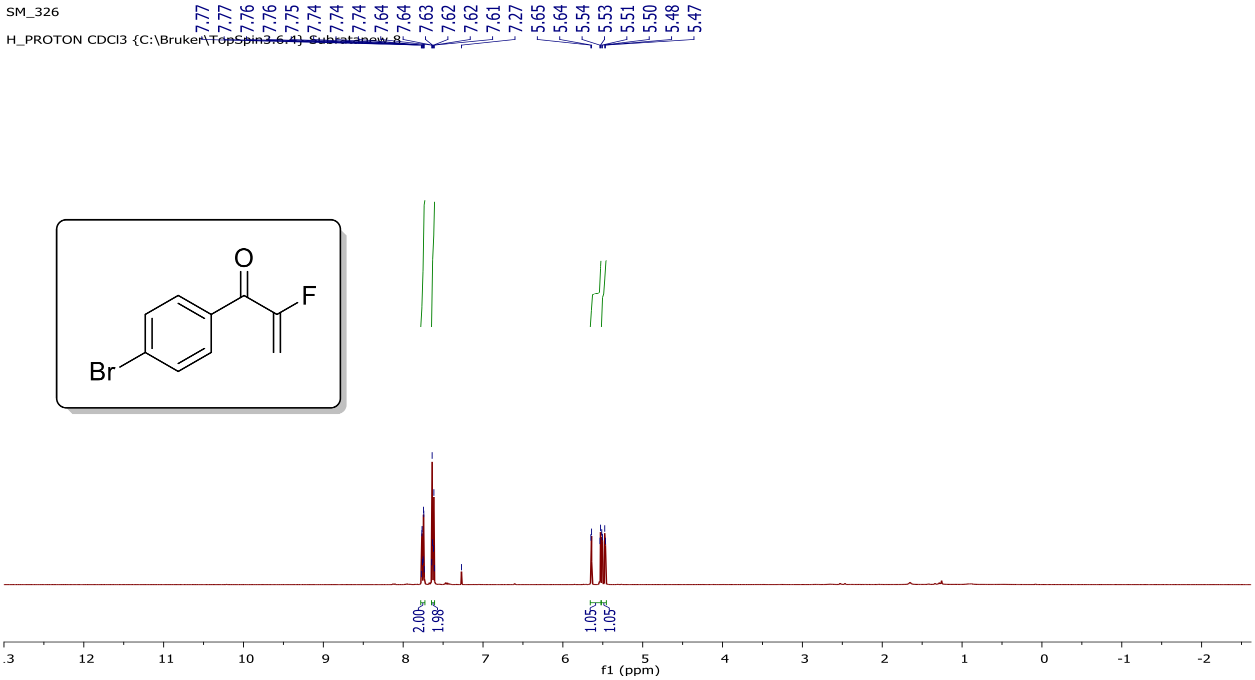

^{1}H NMR Spectrum of Compound **38** (400 MHz, $CDCl_3$)

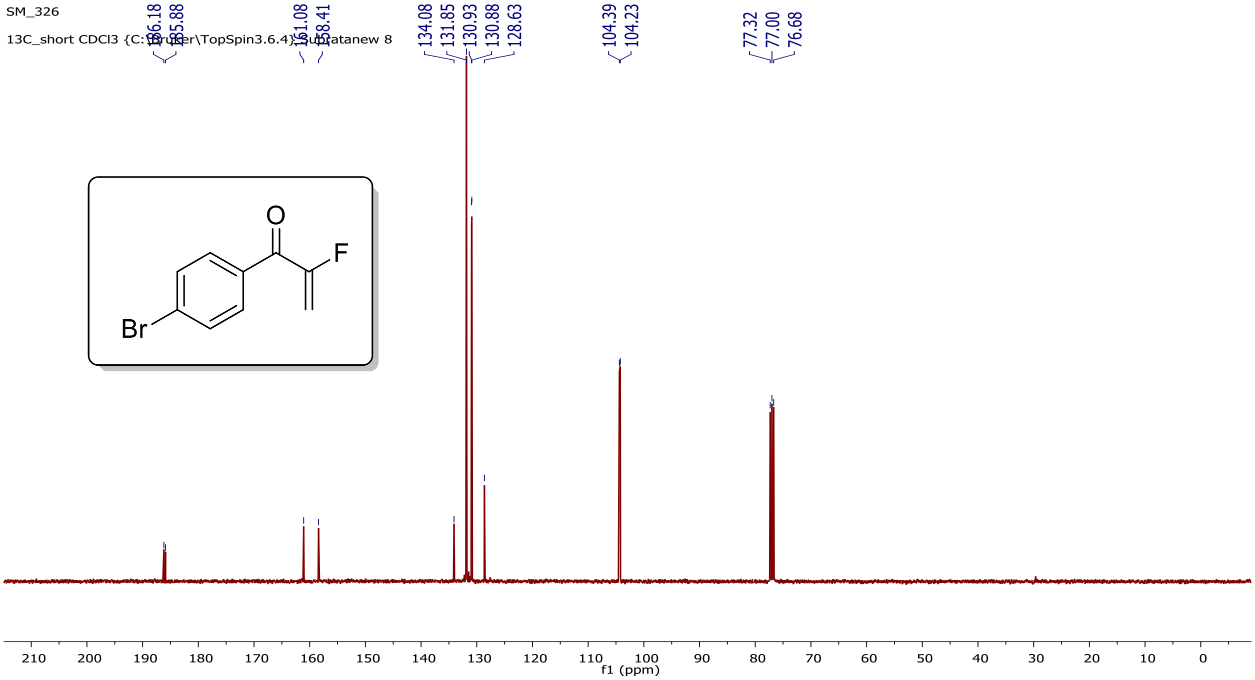

^{13}C NMR Spectrum of Compound **38** (101 MHz, $CDCl_3$)

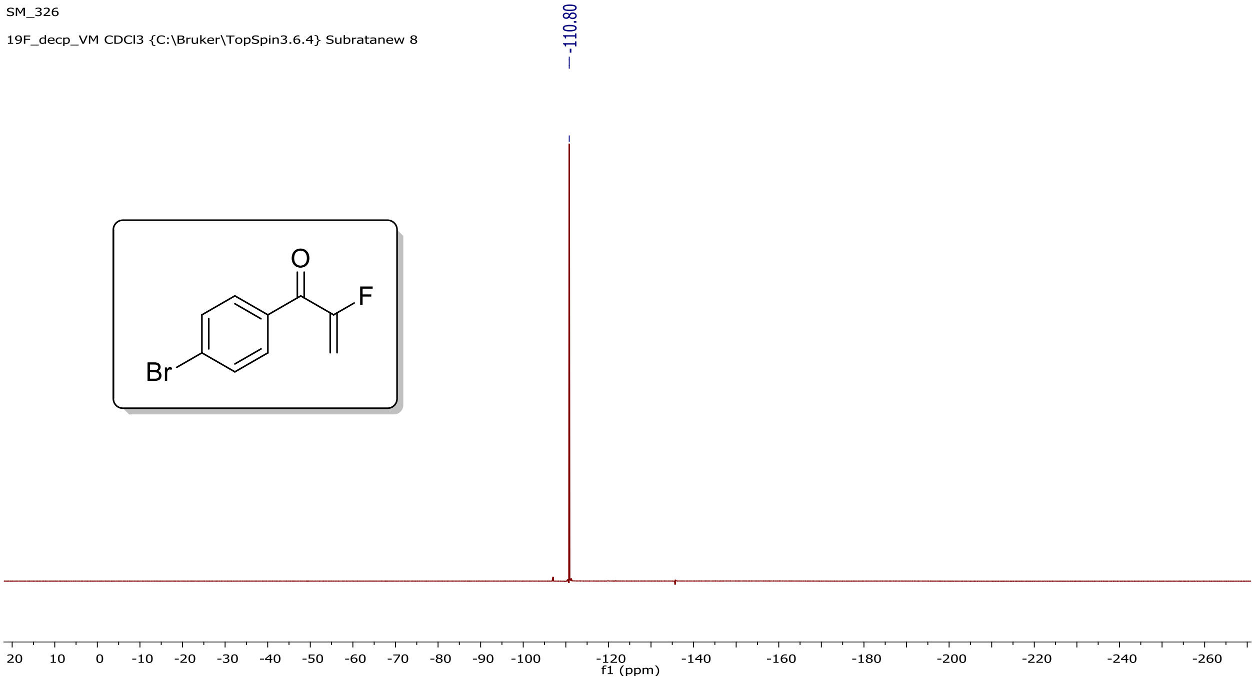

^{19}F NMR Spectrum of Compound **38** (376 MHz, $CDCl_3$)

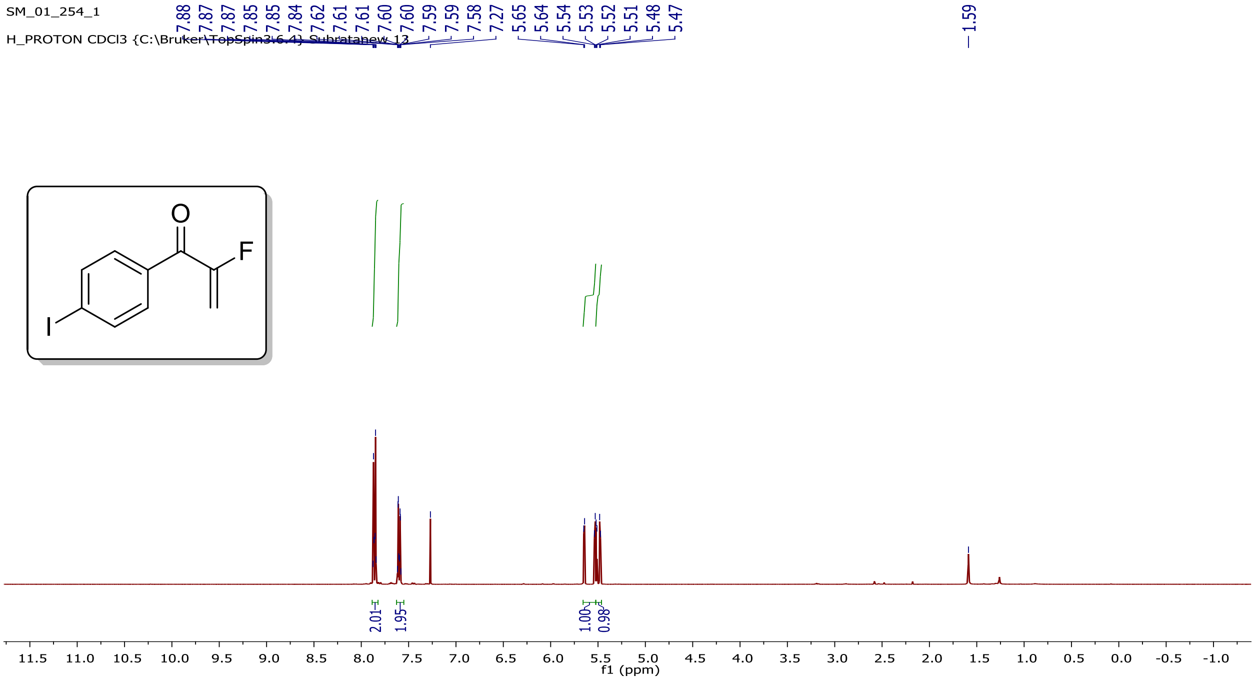

^{1}H NMR Spectrum of Compound **39** (400 MHz, $CDCl_3$)

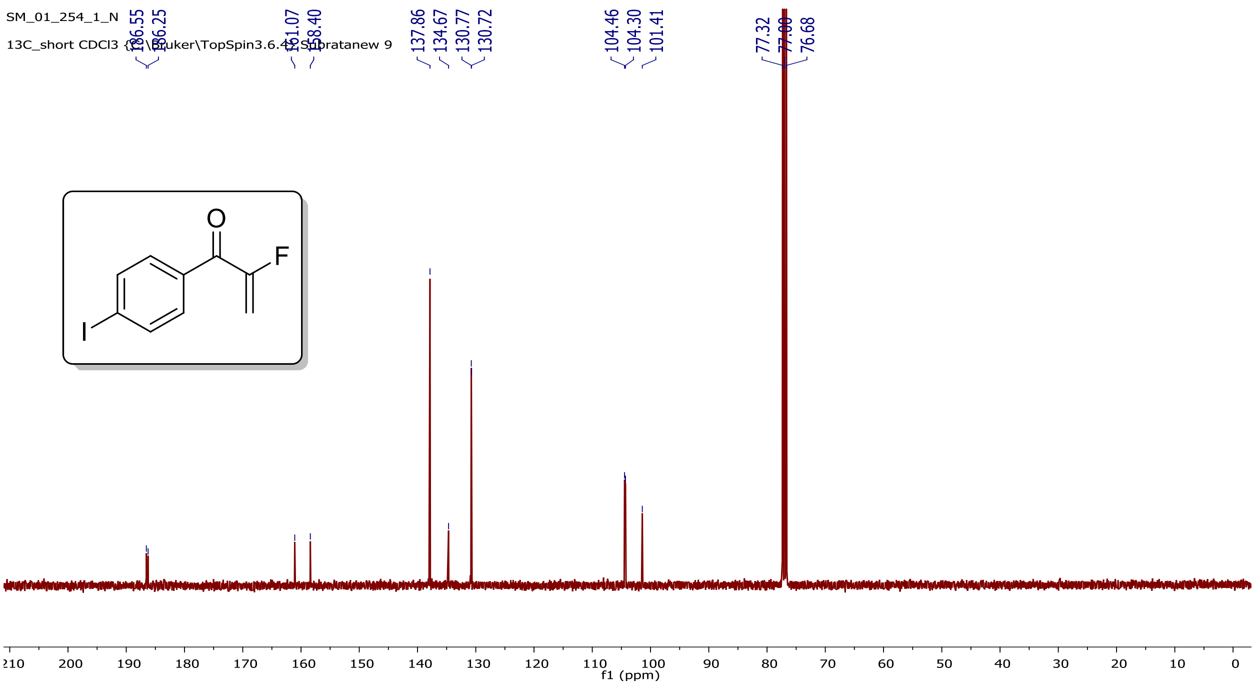

^{13}C NMR Spectrum of Compound **39** (101 MHz, $CDCl_3$)

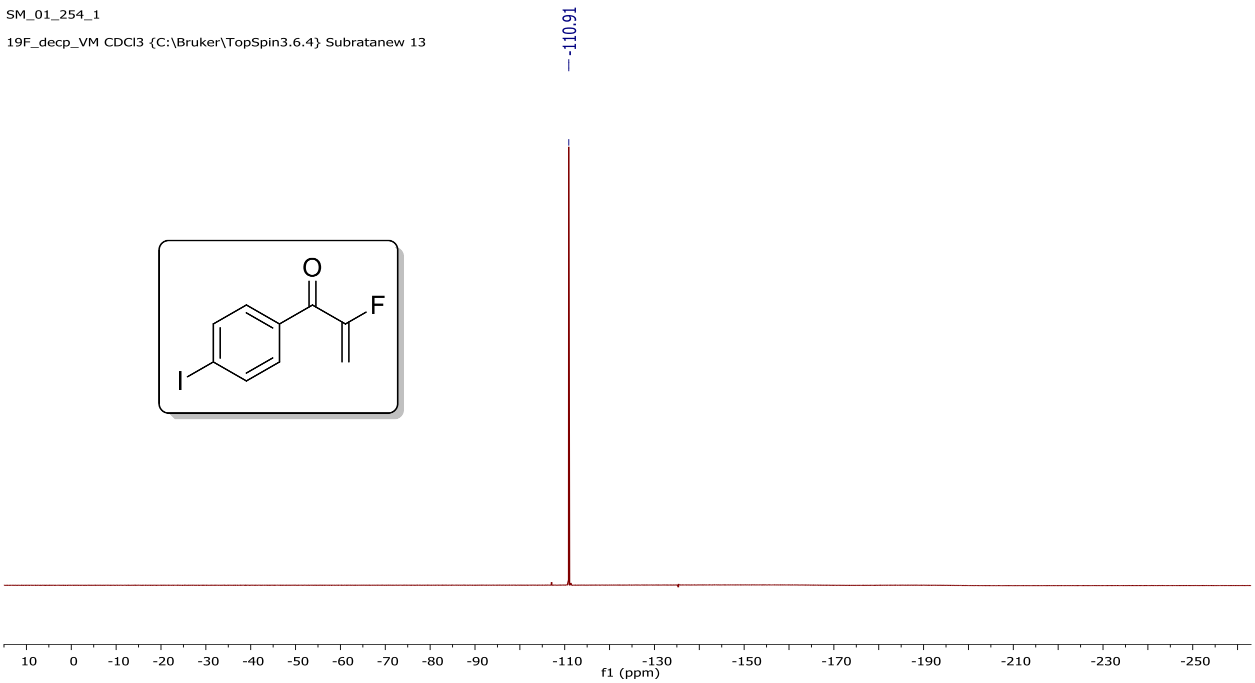

^{19}F NMR Spectrum of Compound **39** (376 MHz, $CDCl_3$)

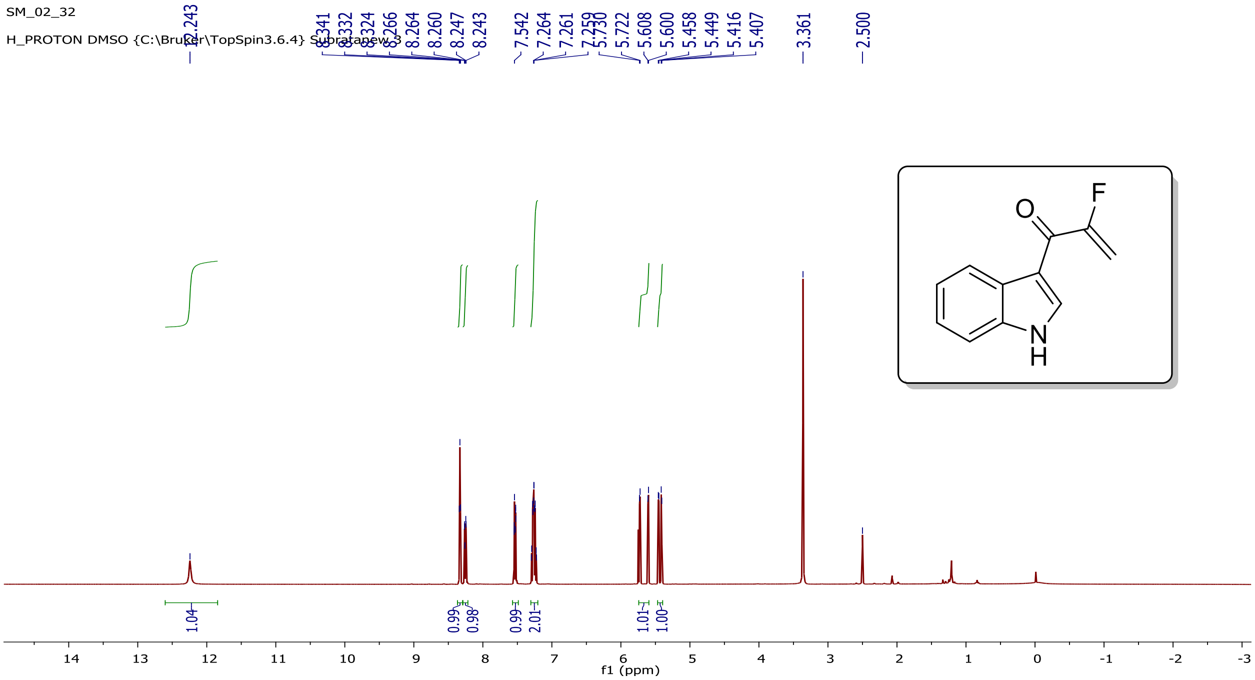

^{1}H NMR Spectrum of Compound **40** (400 MHz, DMSO-d_6)

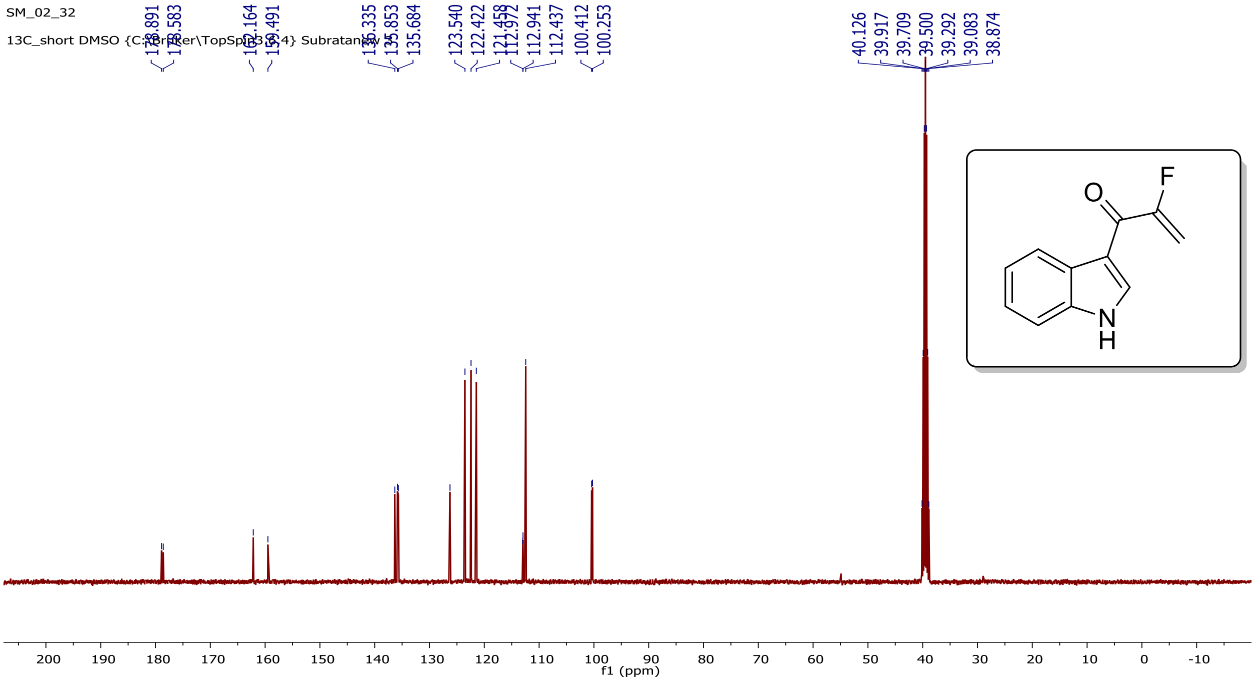

^{13}C NMR Spectrum of Compound **40** (101 MHz, DMSO-d_6)

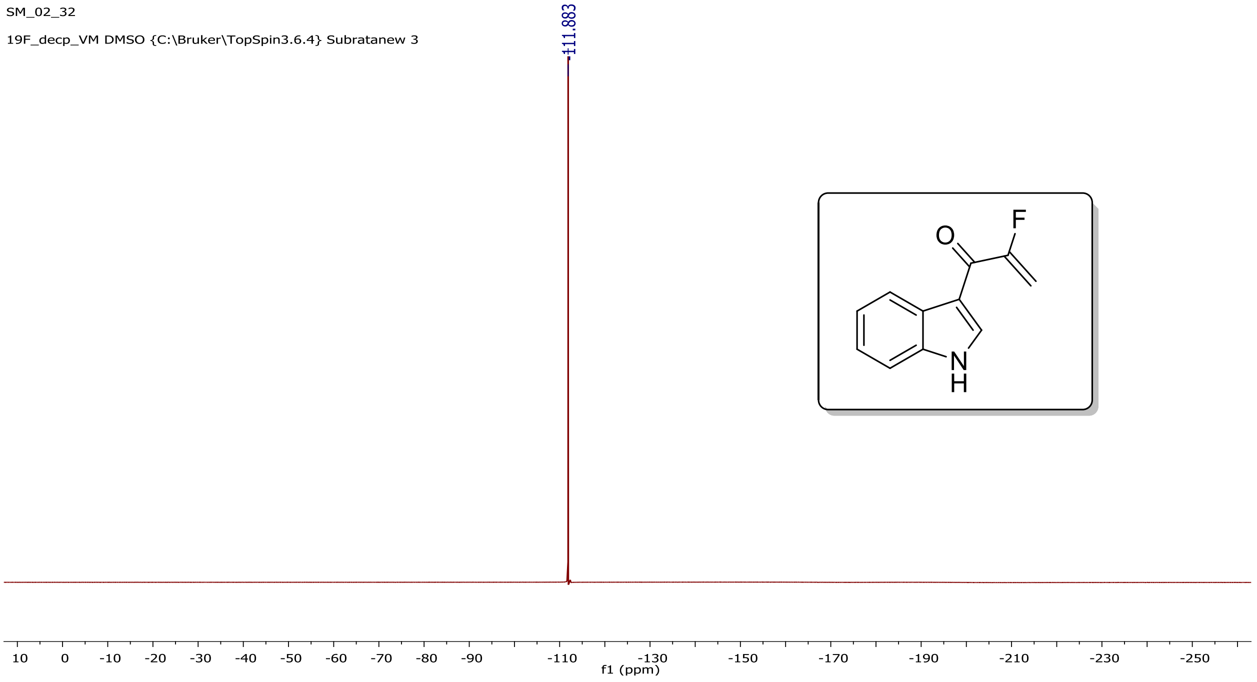

^{19}F NMR Spectrum of Compound **40** (376 MHz, DMSO-d_6)

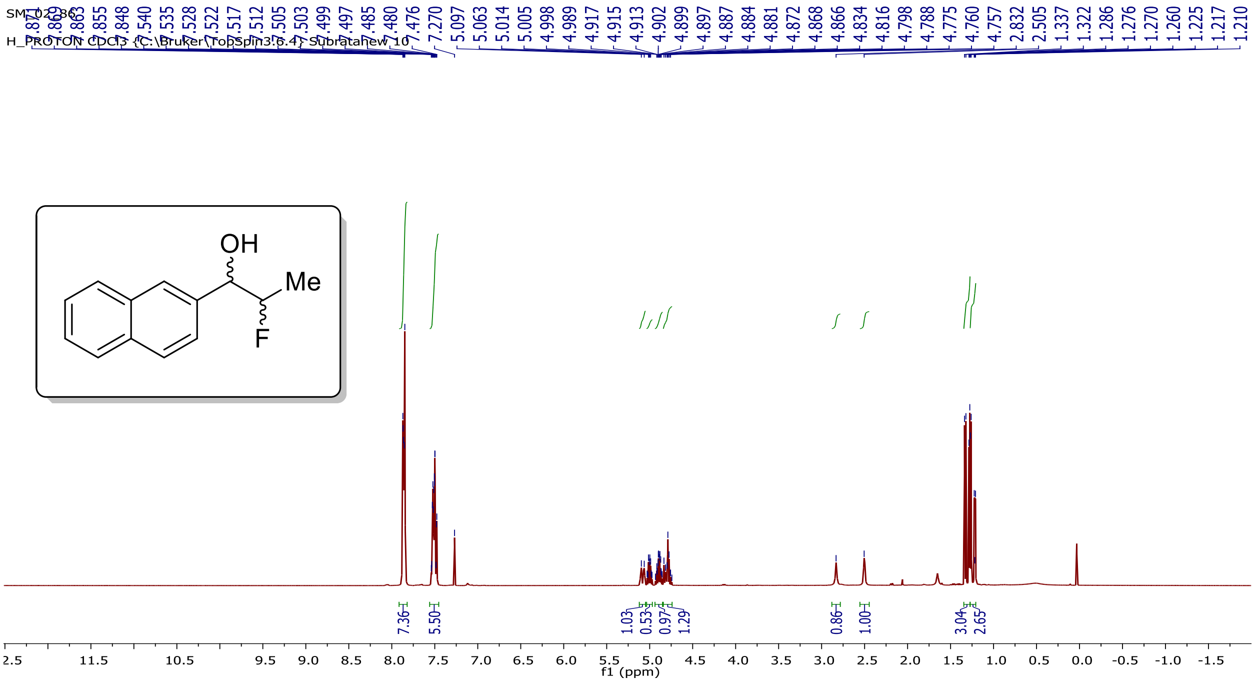

^{1}H NMR Spectrum of Compound **41**(400 MHz, $CDCl_3$)

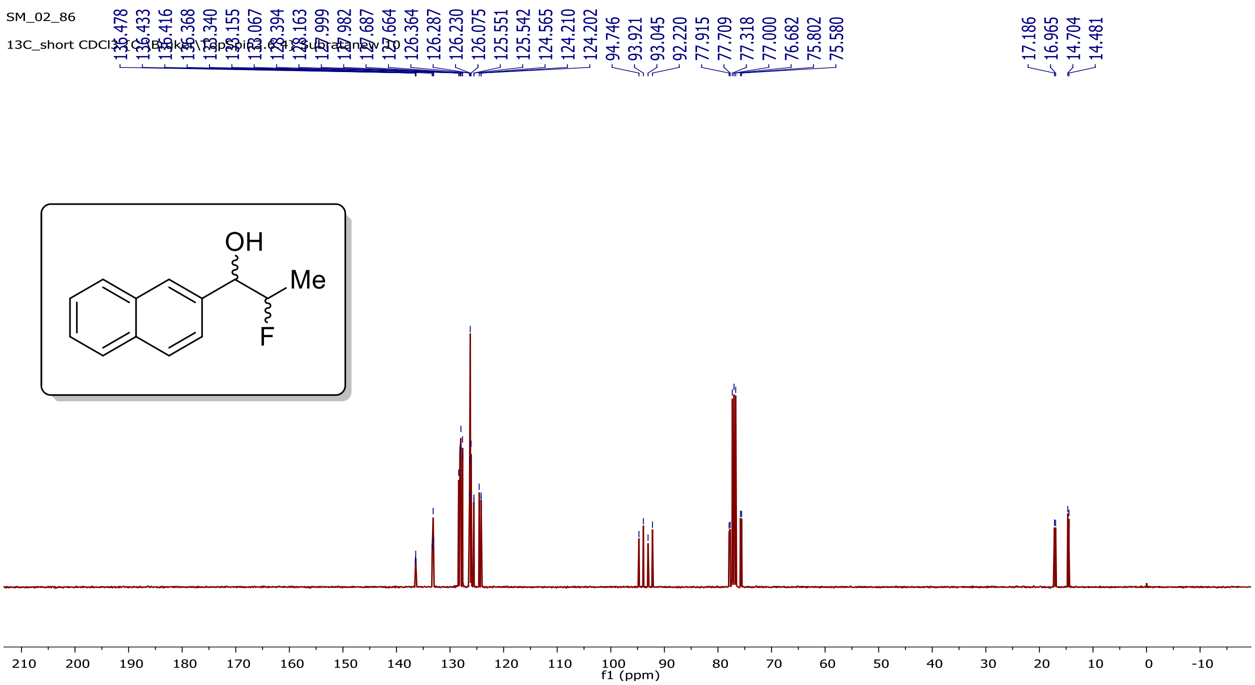

^{13}C NMR Spectrum of Compound **41** (101 MHz, $CDCl_3$)

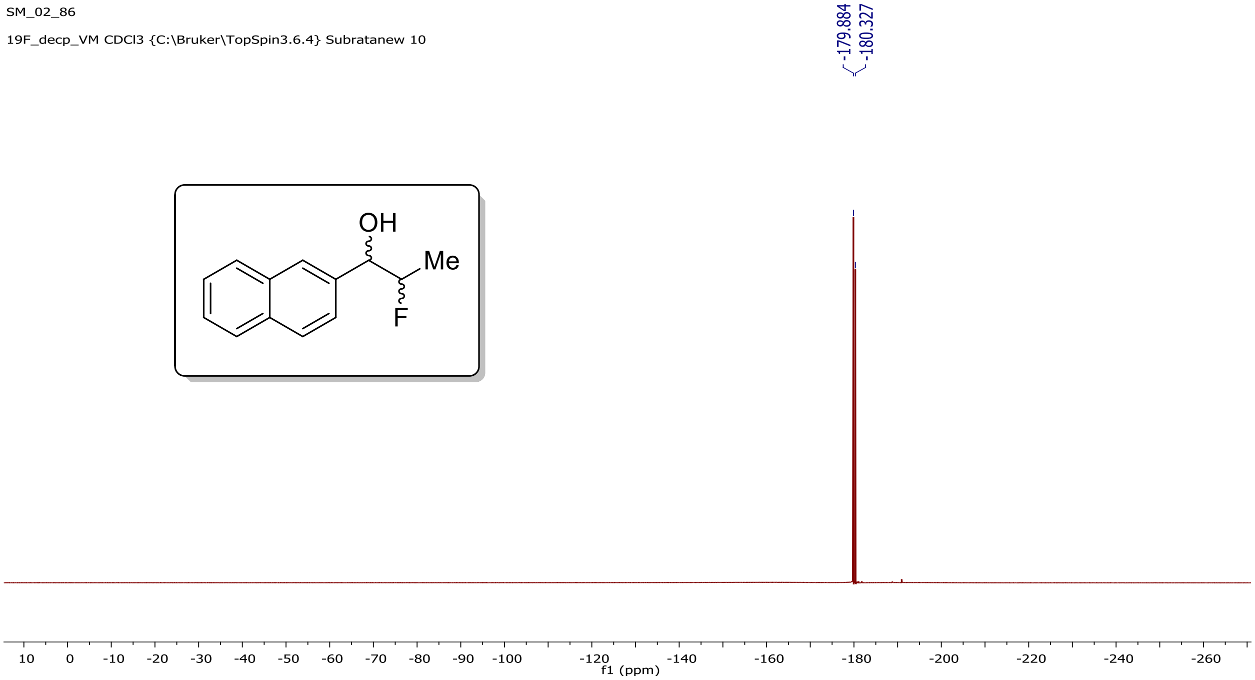

^{19}F NMR Spectrum of Compound **41** (376 MHz, $CDCl_3$)

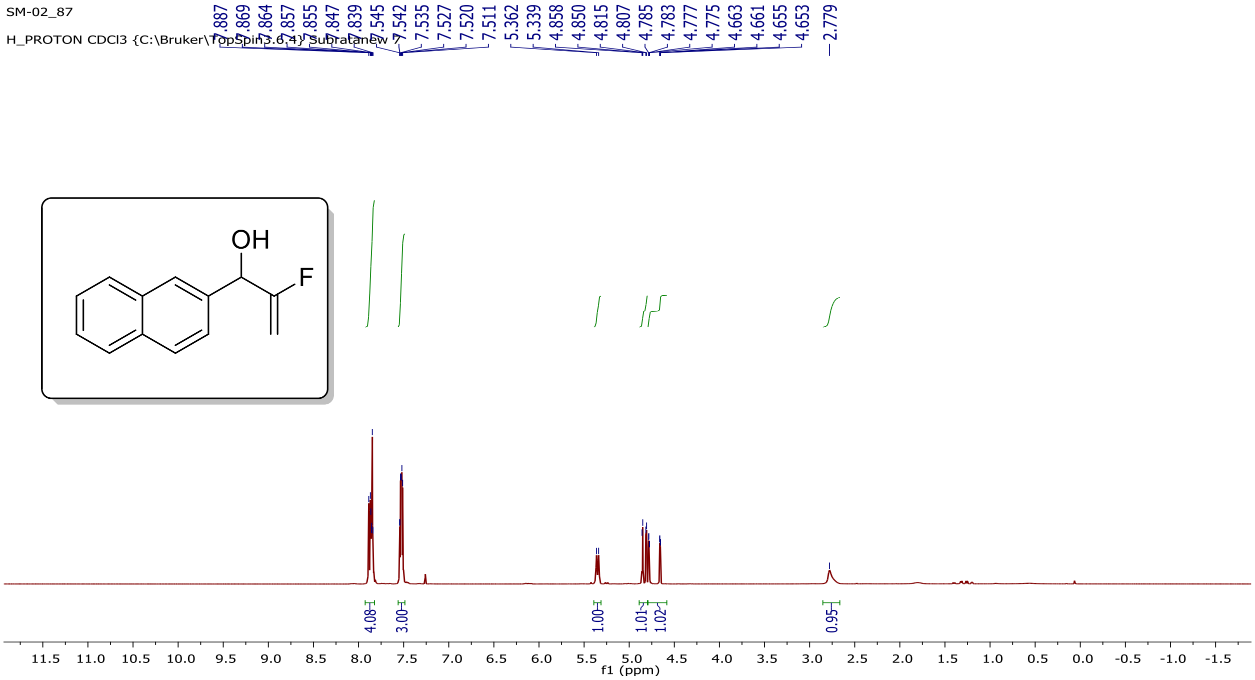

^{1}H NMR Spectrum of Compound **42** (400 MHz, $CDCl_3$)

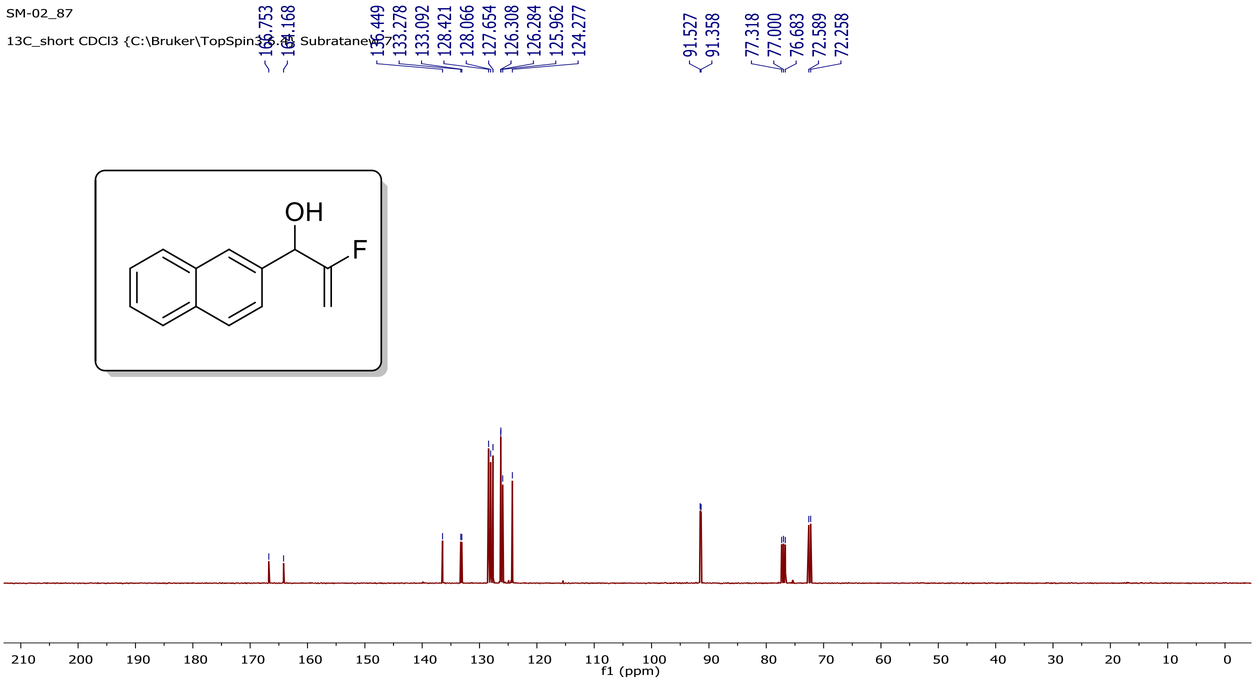

^{13}C NMR Spectrum of Compound **42** (101 MHz, $CDCl_3$)

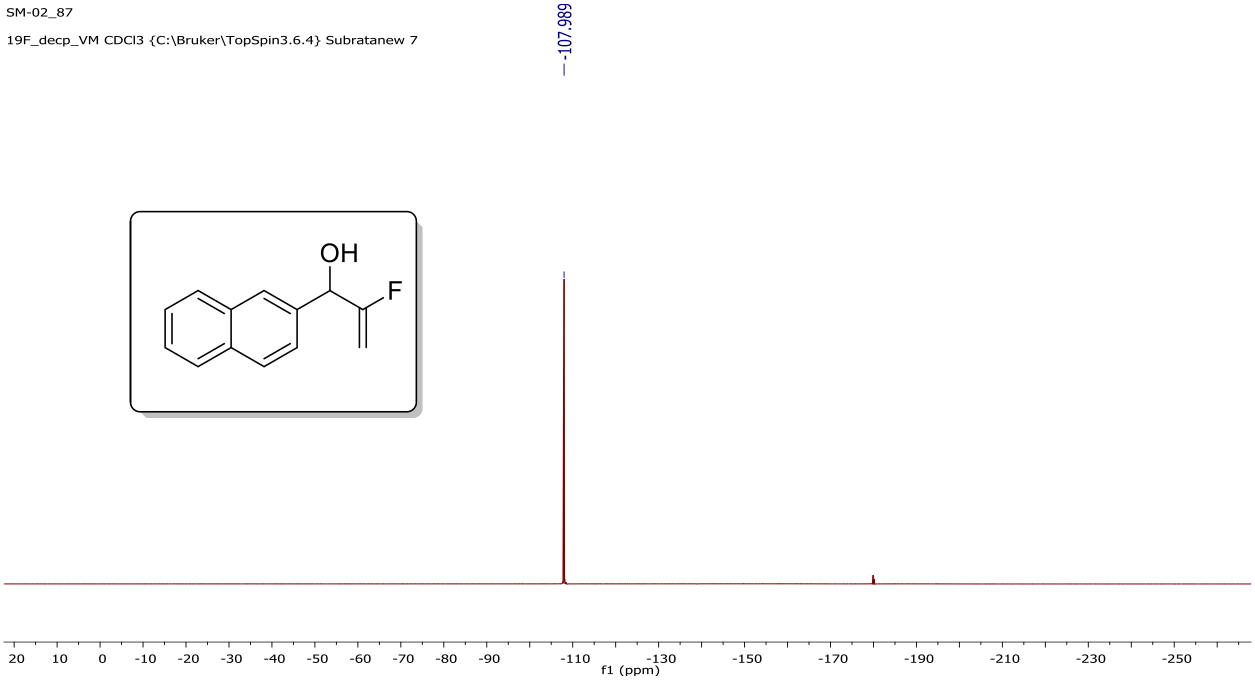

^{19}F NMR Spectrum of Compound **42** (376 MHz, $CDCl_3$)

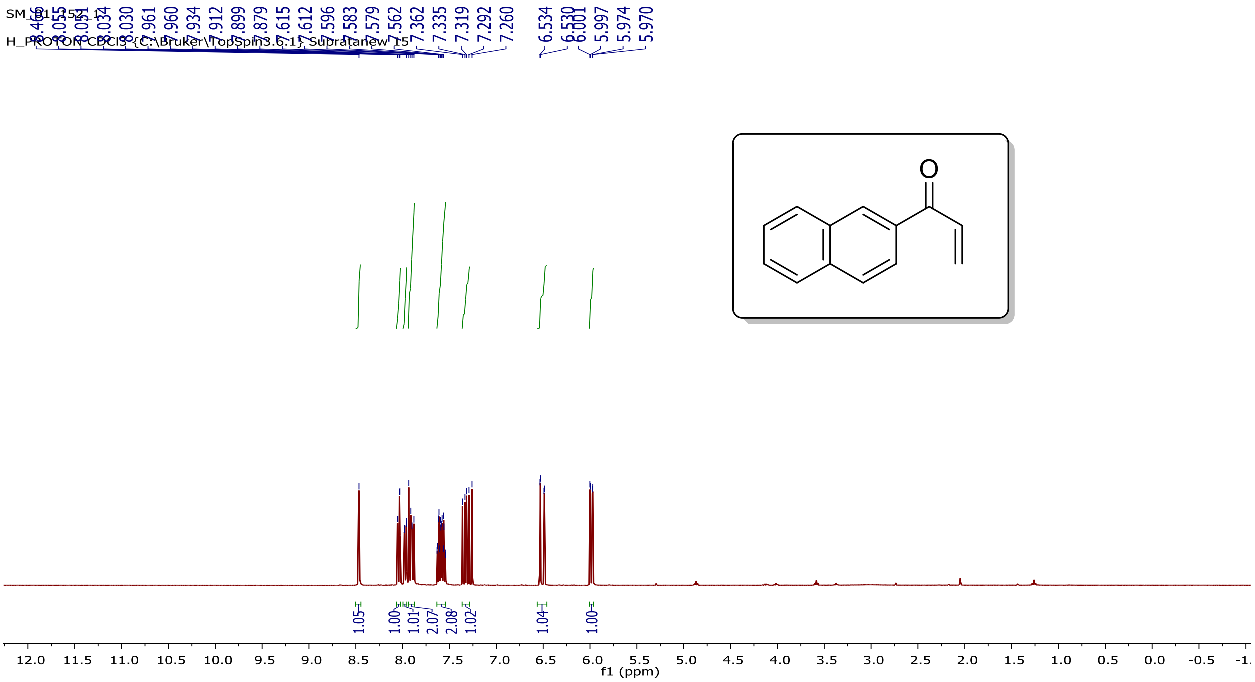

^{1}H NMR Spectrum of Compound **1** (400 MHz, $CDCl_3$)

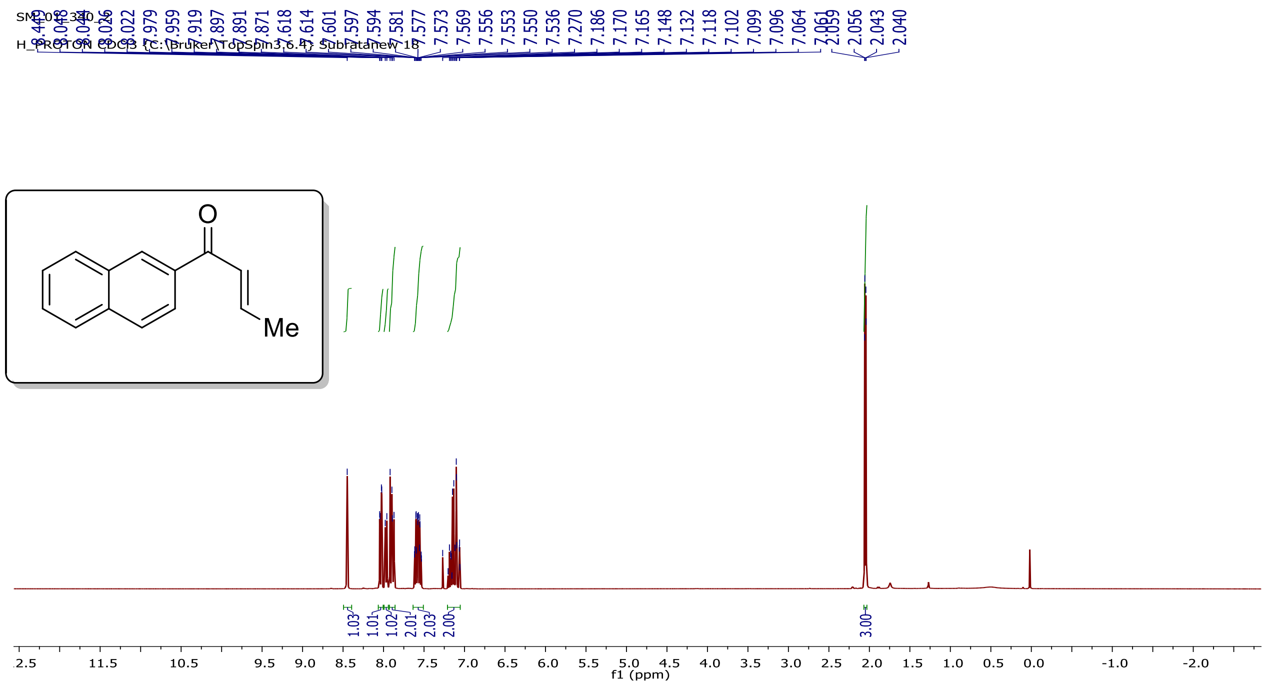

^{1}H NMR Spectrum of Compound **10a** (400 MHz, $CDCl_3$)

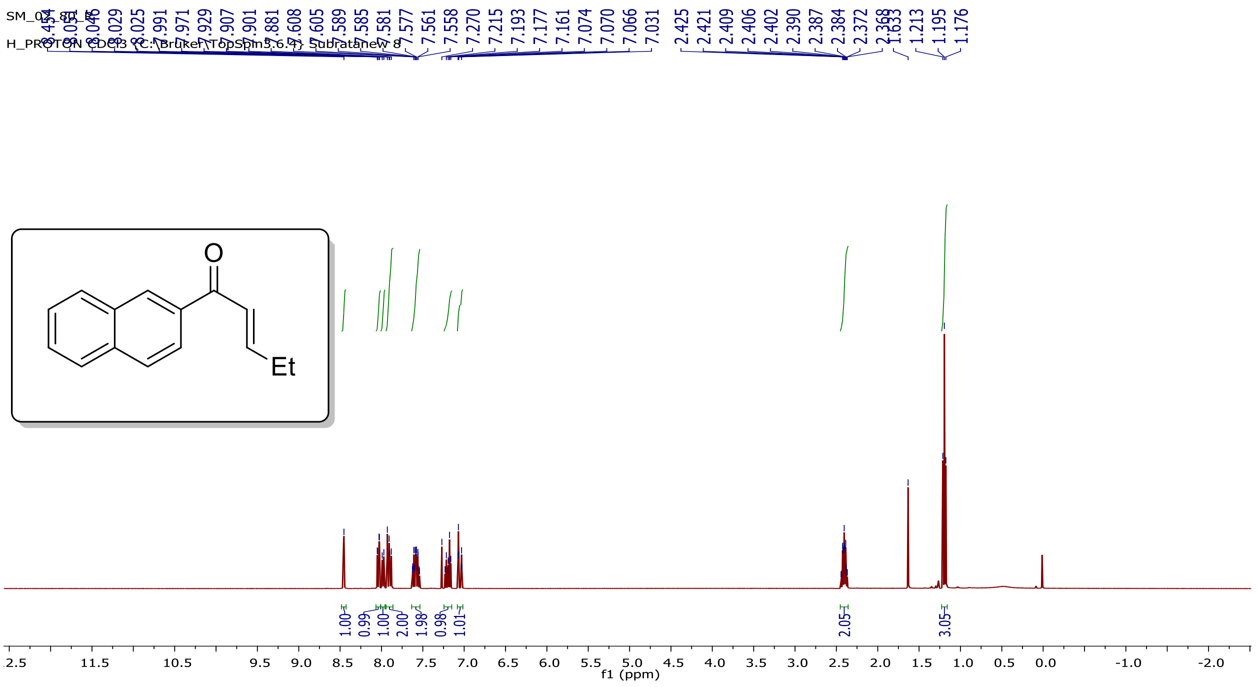

^{1}H NMR Spectrum of Compound **10b** (400 MHz, $CDCl_3$)

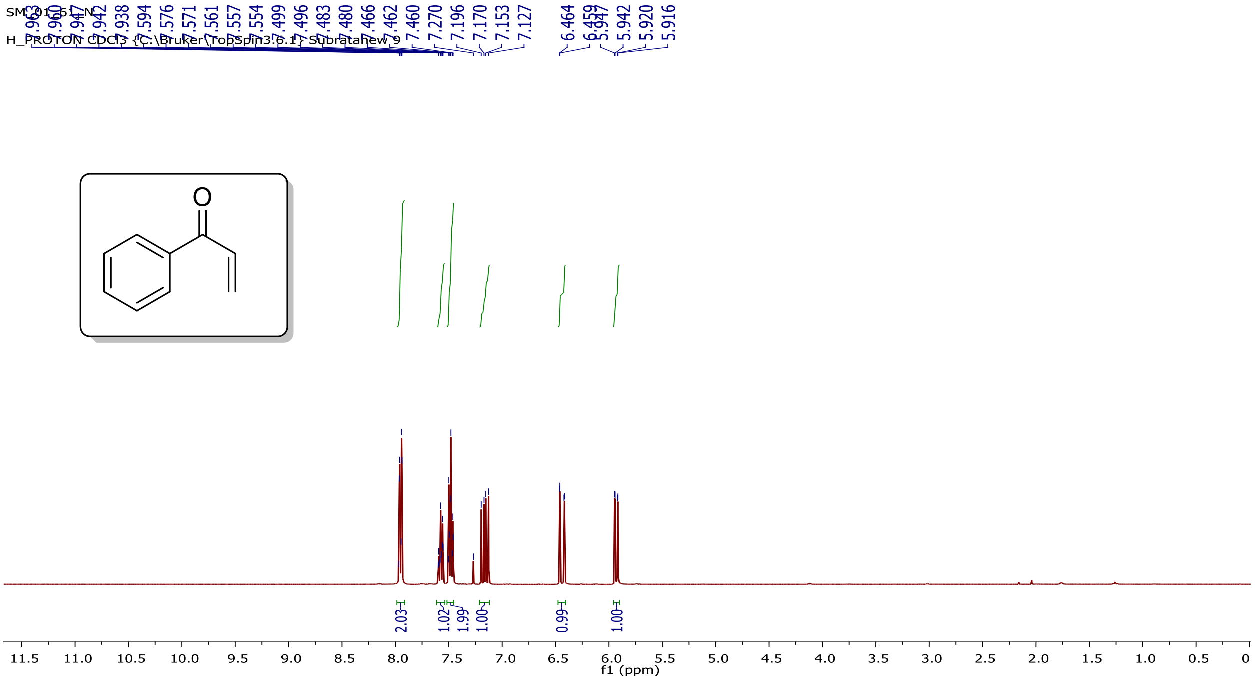

^{1}H NMR Spectrum of Compound **48a** (400 MHz, $CDCl_3$)

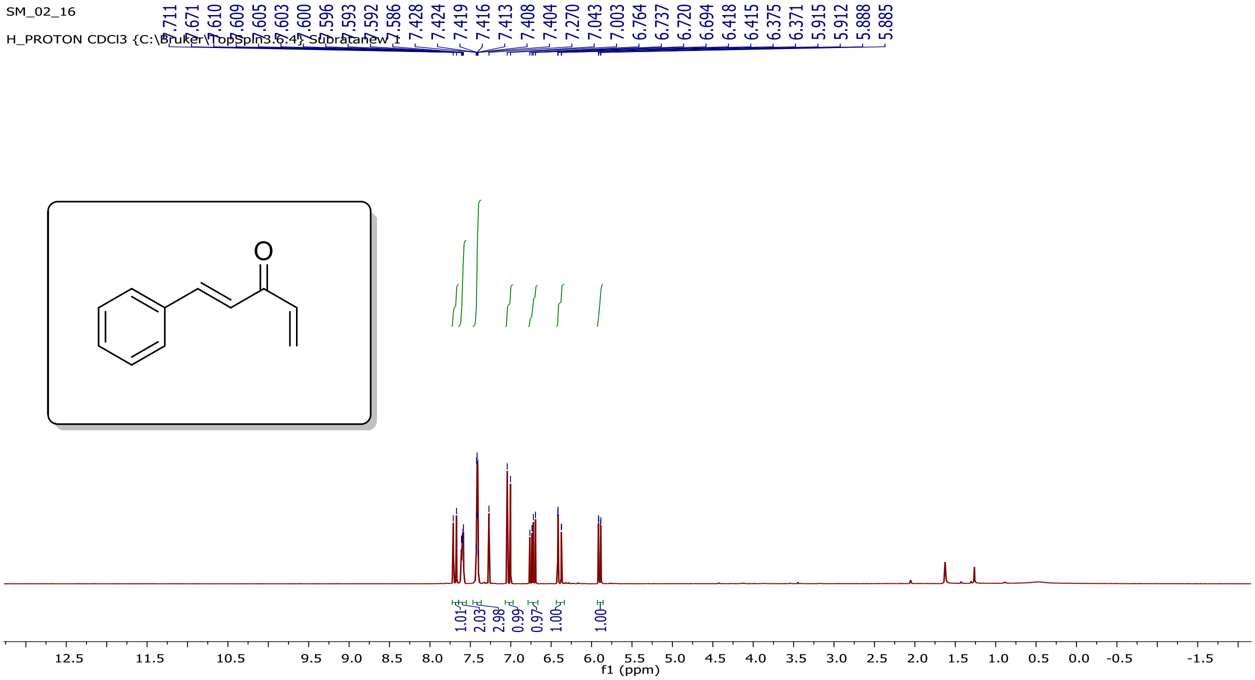

^{1}H NMR Spectrum of Compound **48b** (400 MHz, $CDCl_3$)

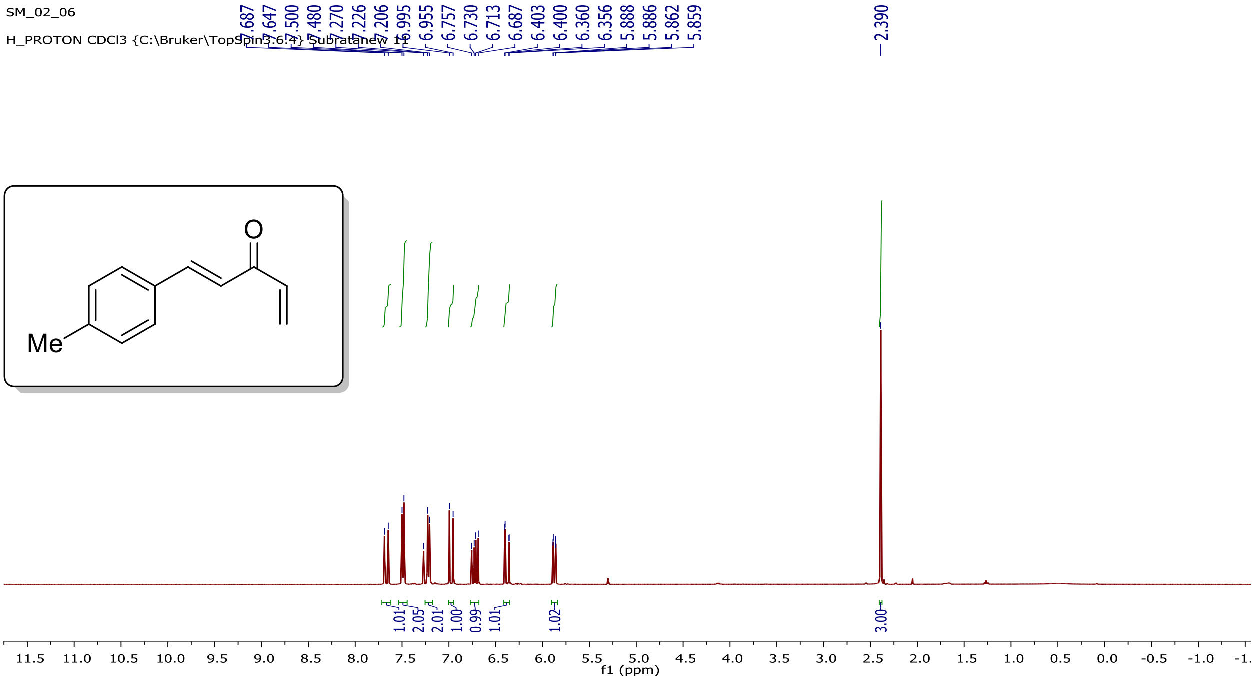

^{1}H NMR Spectrum of Compound **48c** (400 MHz, $CDCl_3$)

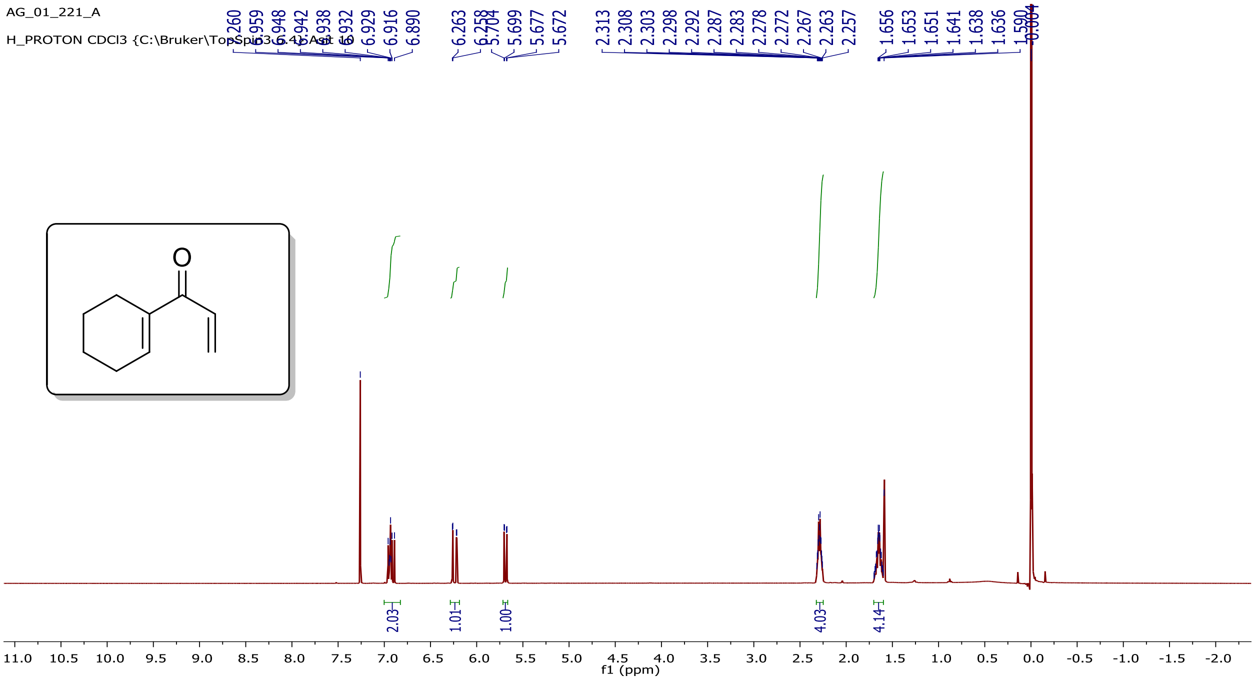

^{1}H NMR Spectrum of Compound **48d** (400 MHz, $CDCl_3$)

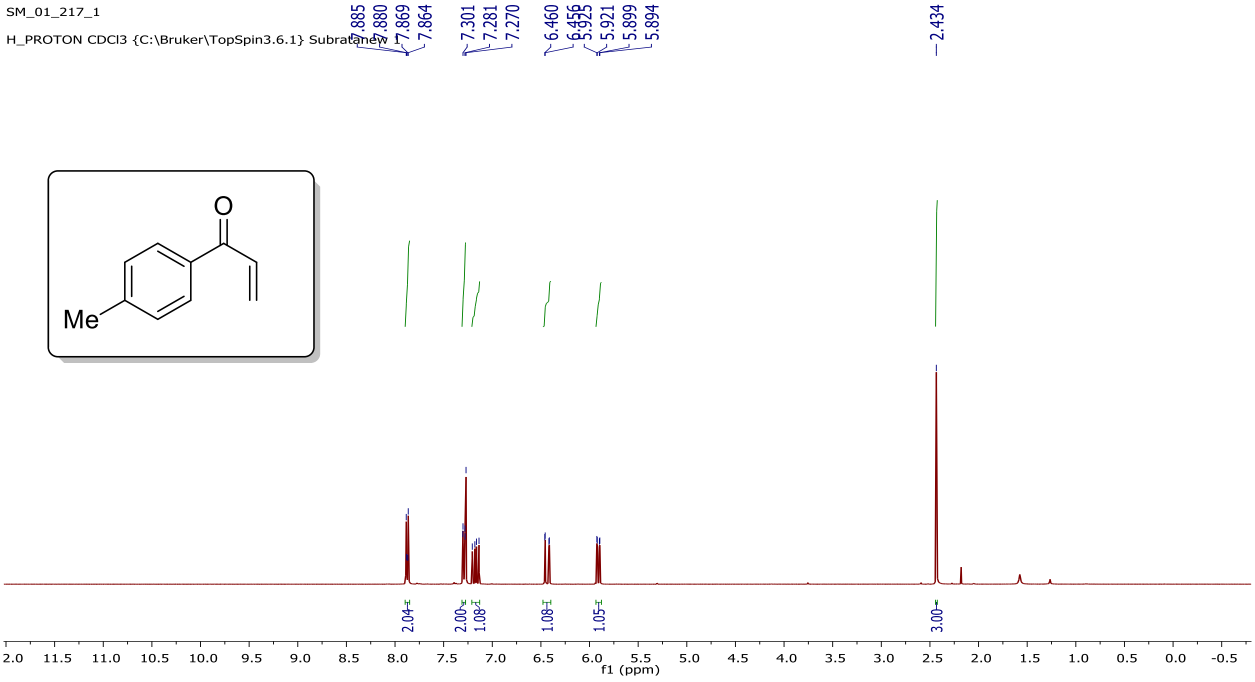

^{1}H NMR Spectrum of Compound **48e** (400 MHz, $CDCl_3$)

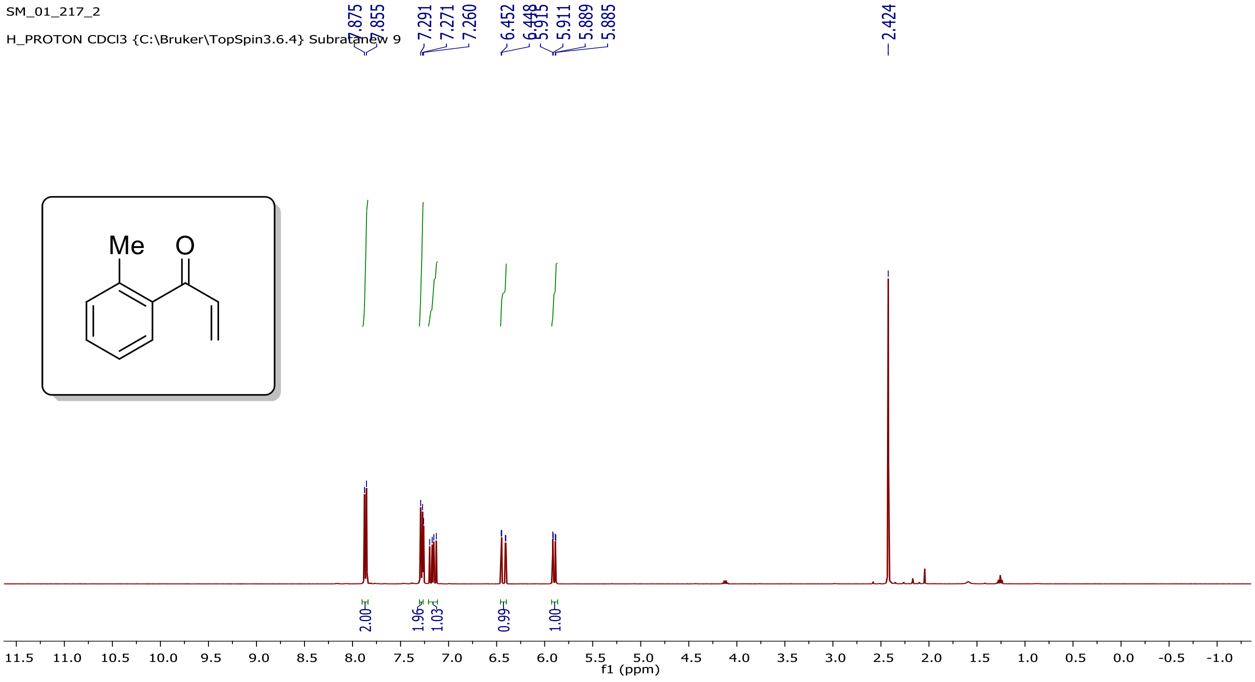

^{1}H NMR Spectrum of Compound **48f** (400 MHz, $CDCl_3$)

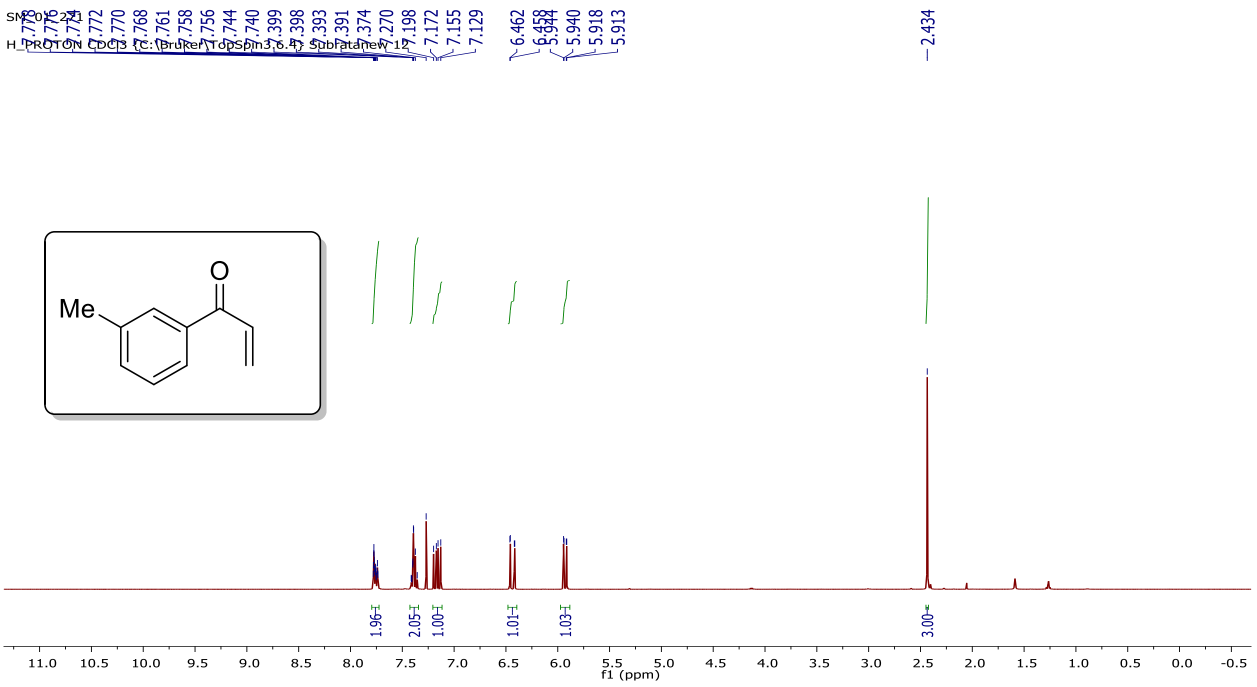

^{1}H NMR Spectrum of Compound **48g** (400 MHz, $CDCl_3$)

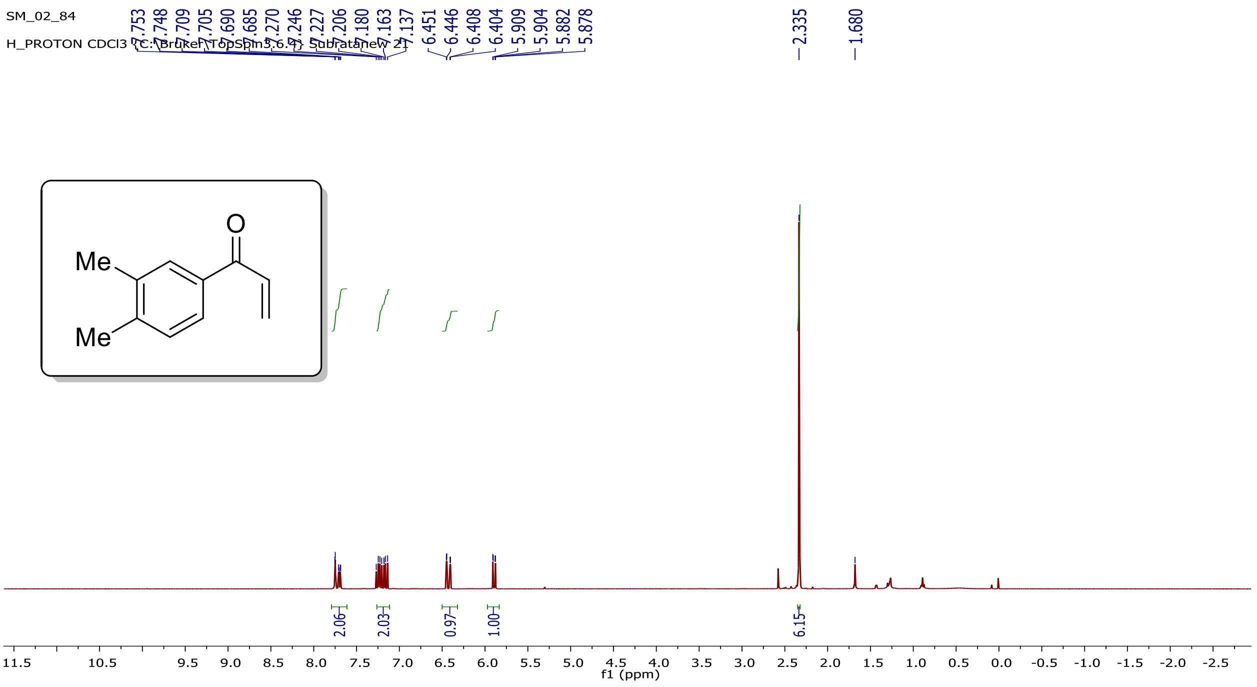

^{1}H NMR Spectrum of Compound **48h** (400 MHz, $CDCl_3$)

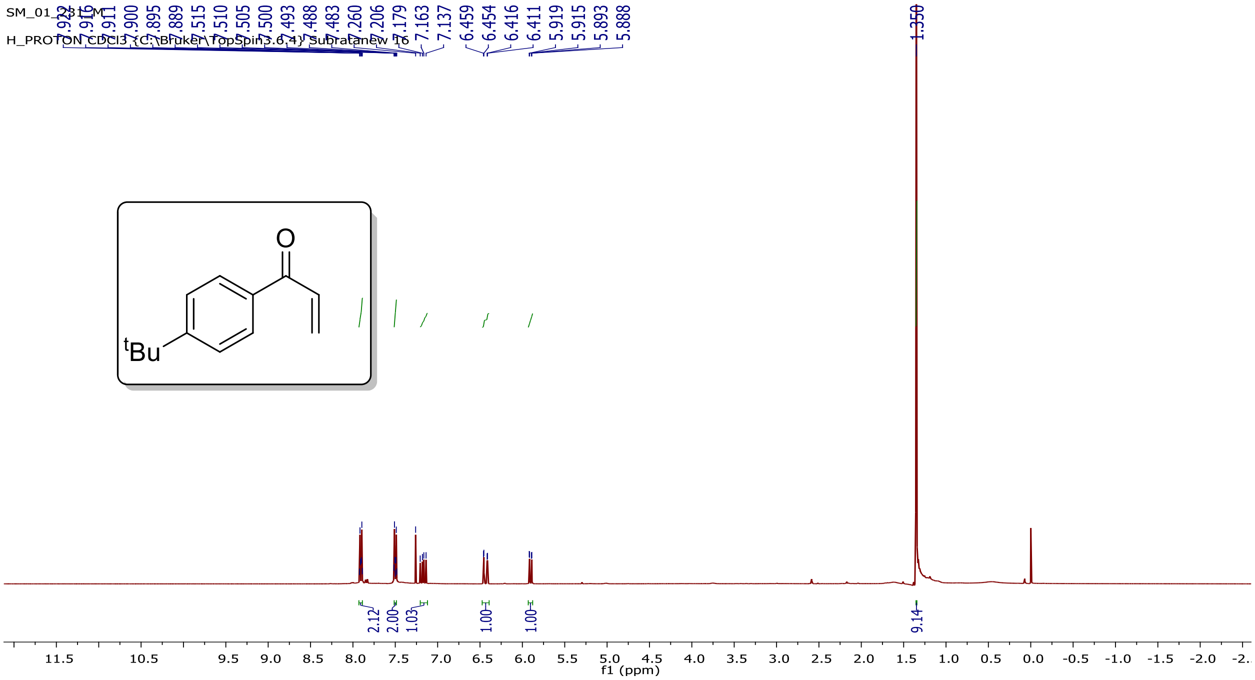

^{1}H NMR Spectrum of Compound **48i** (400 MHz, $CDCl_3$)

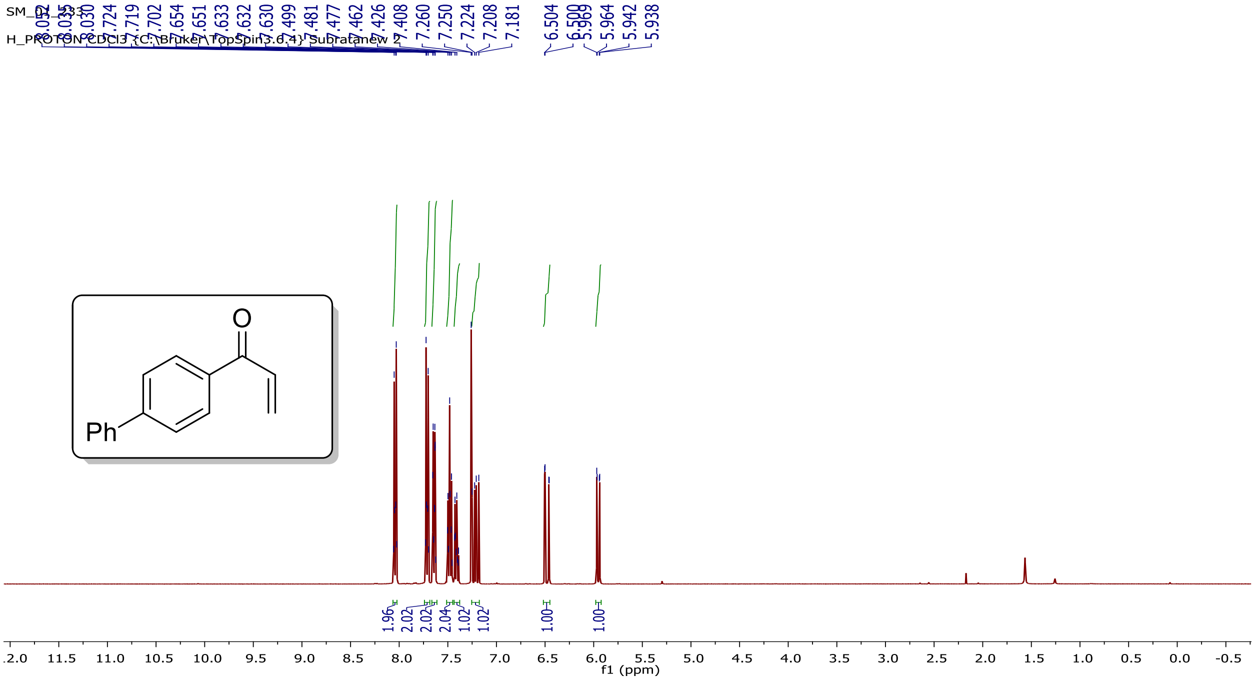

^{1}H NMR Spectrum of Compound **48j** (400 MHz, $CDCl_3$)

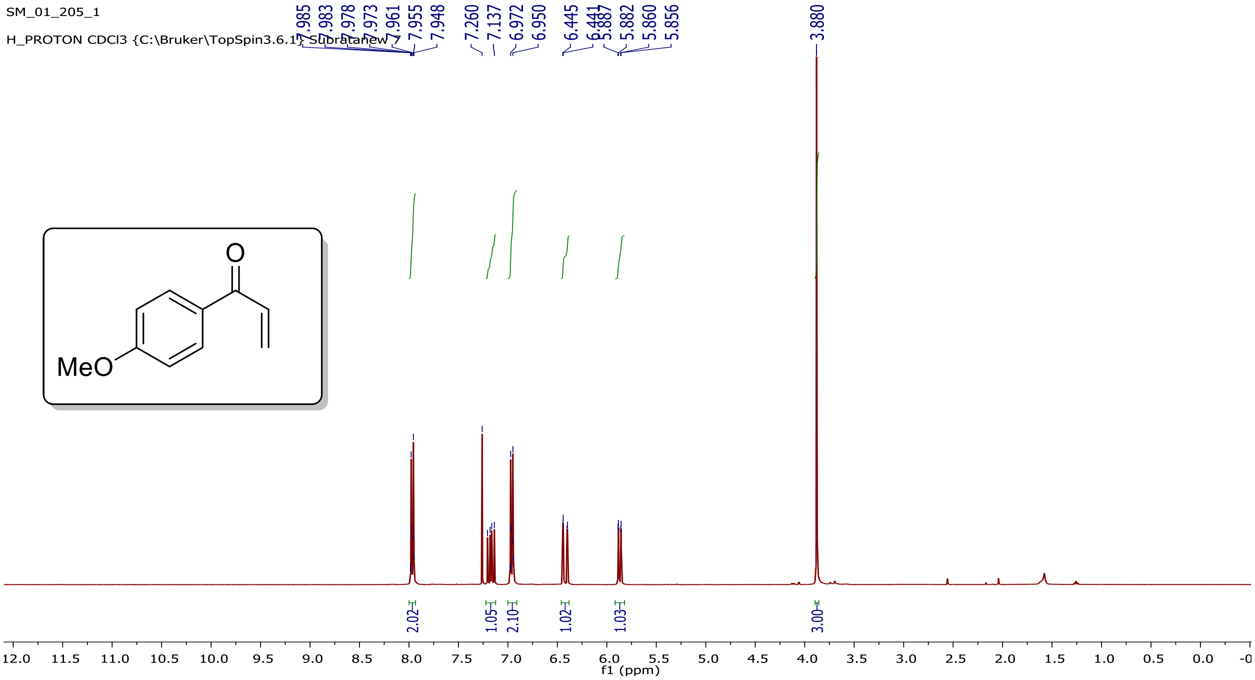

^{1}H NMR Spectrum of Compound **48k** (400 MHz, $CDCl_3$)

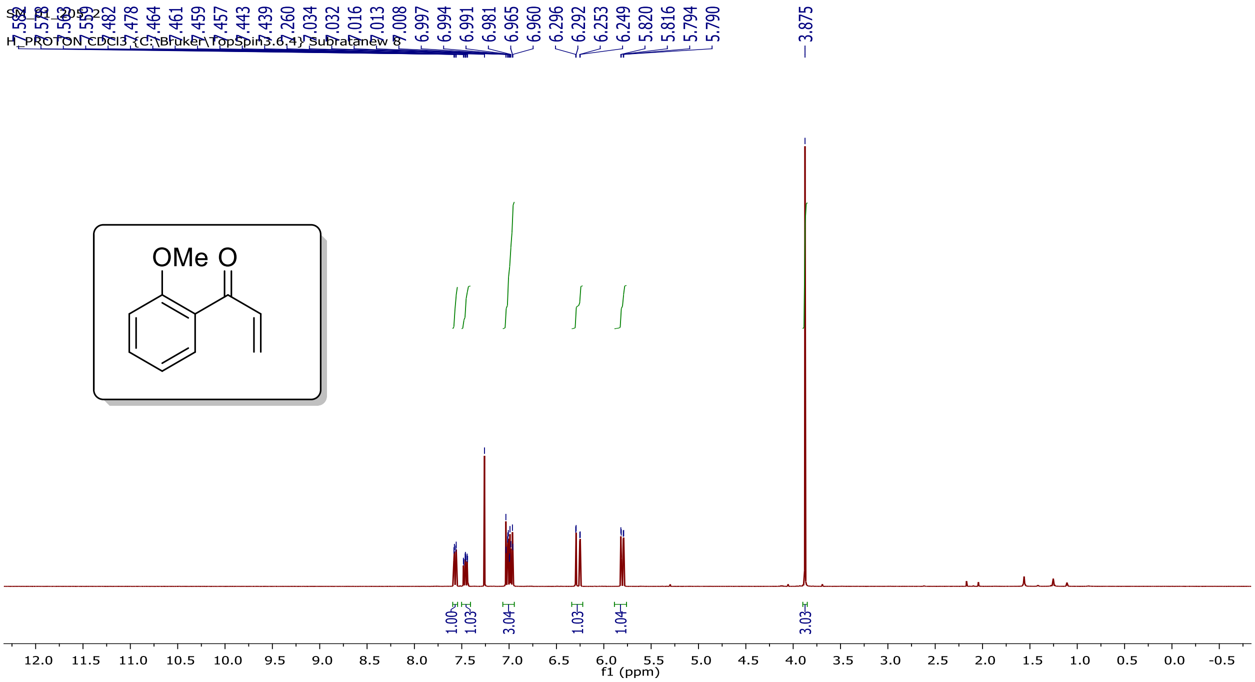

^{1}H NMR Spectrum of Compound **48l** (400 MHz, $CDCl_3$)

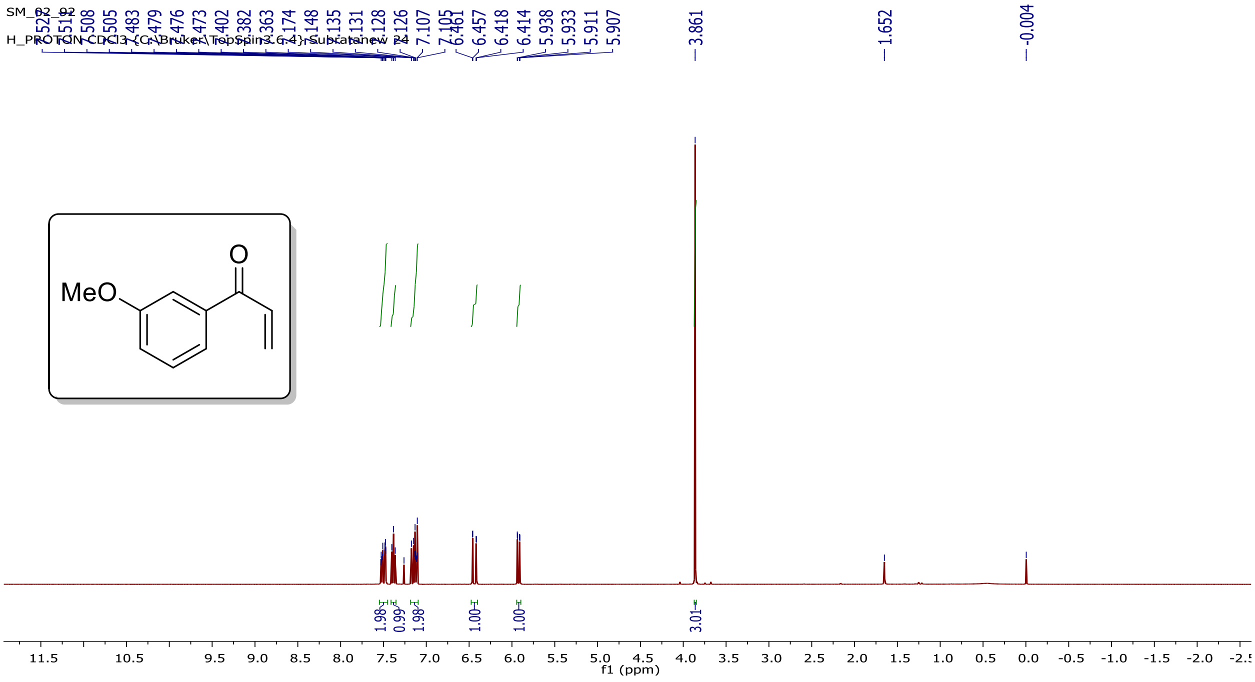

^{1}H NMR Spectrum of Compound **48m** (400 MHz, $CDCl_3$)

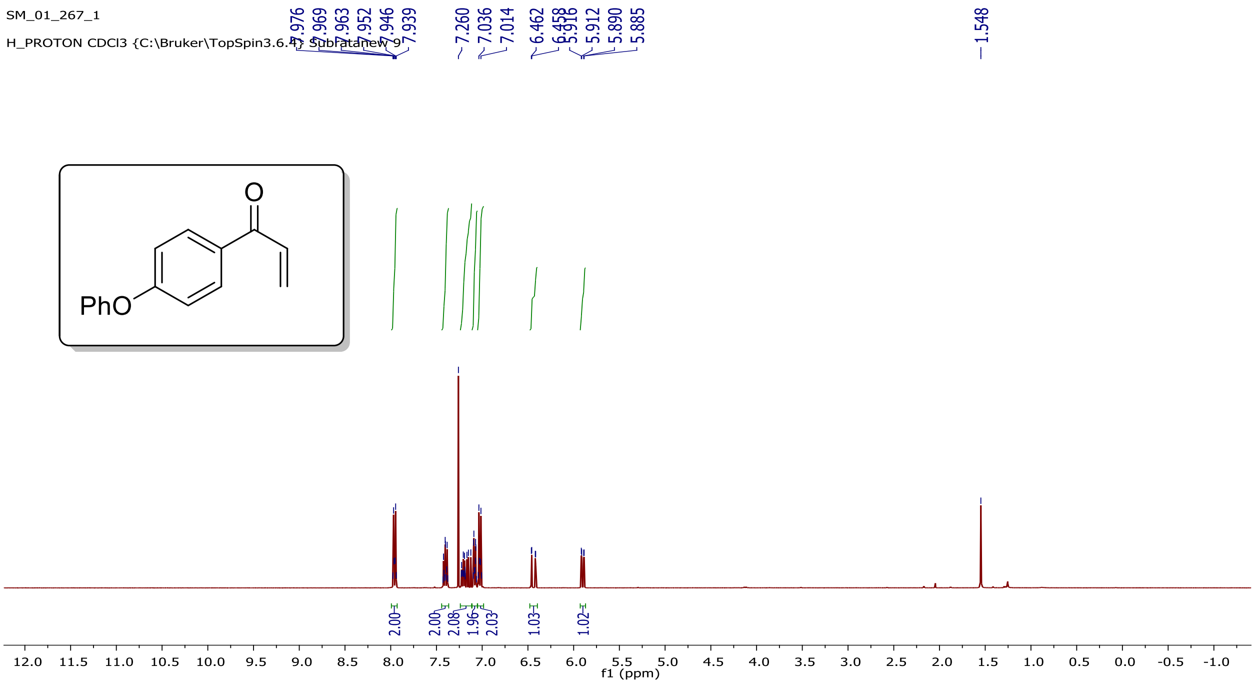

^{1}H NMR Spectrum of Compound **48n** (400 MHz, $CDCl_3$)

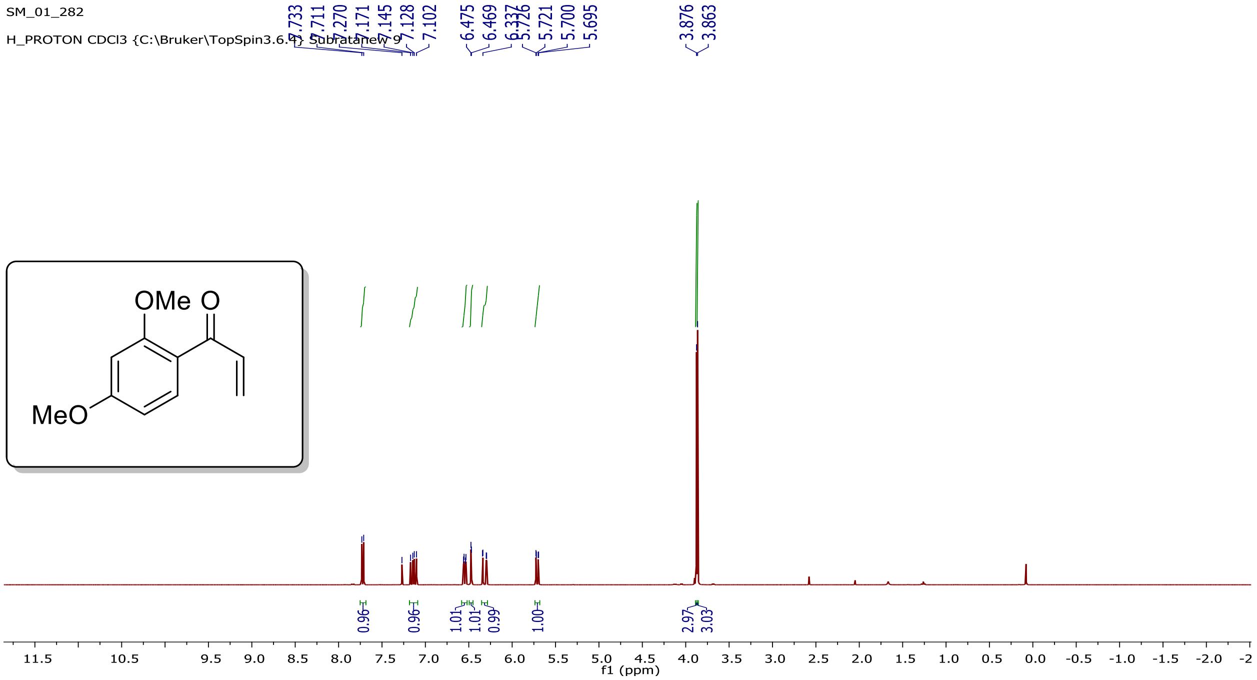

^{1}H NMR Spectrum of Compound **48o** (400 MHz, $CDCl_3$)

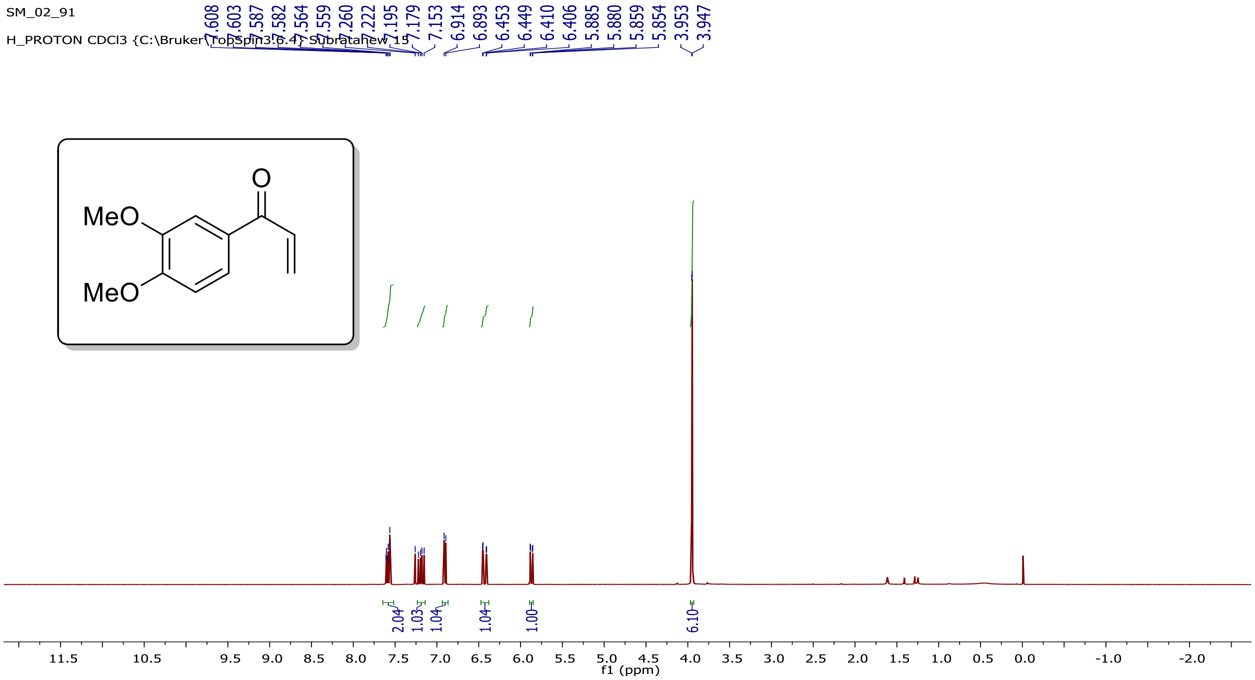

^{1}H NMR Spectrum of Compound **48p** (400 MHz, $CDCl_3$)

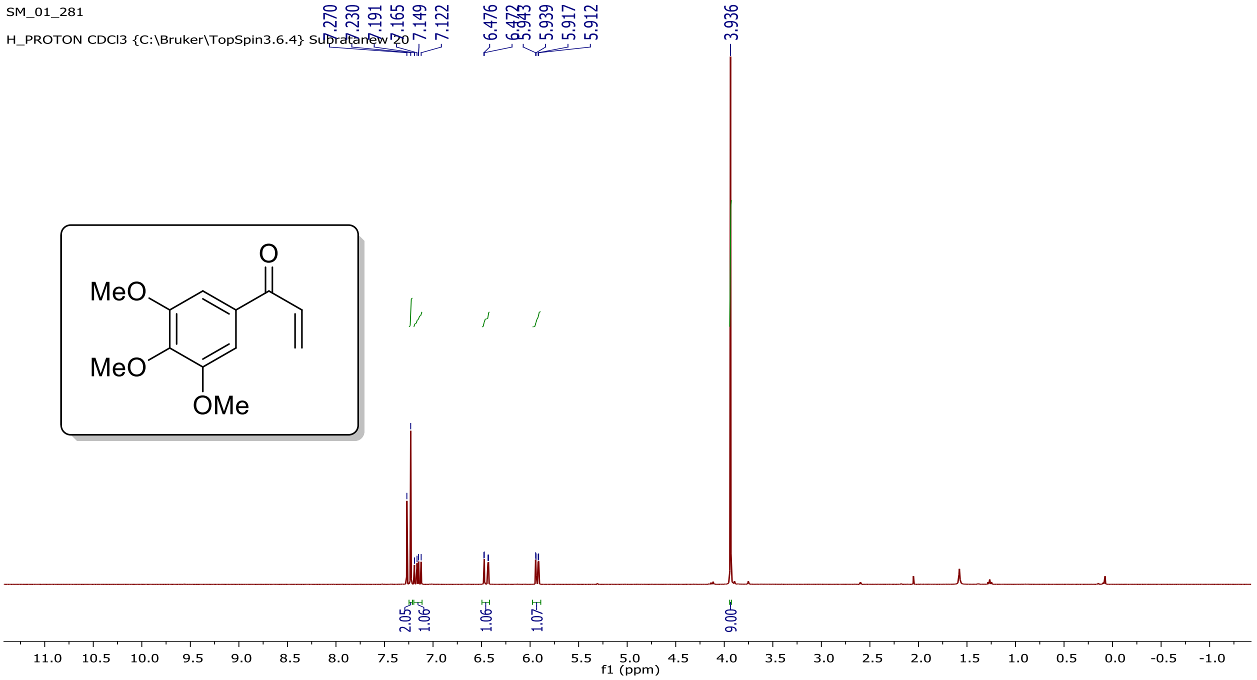

^{1}H NMR Spectrum of Compound **48q** (400 MHz, $CDCl_3$)

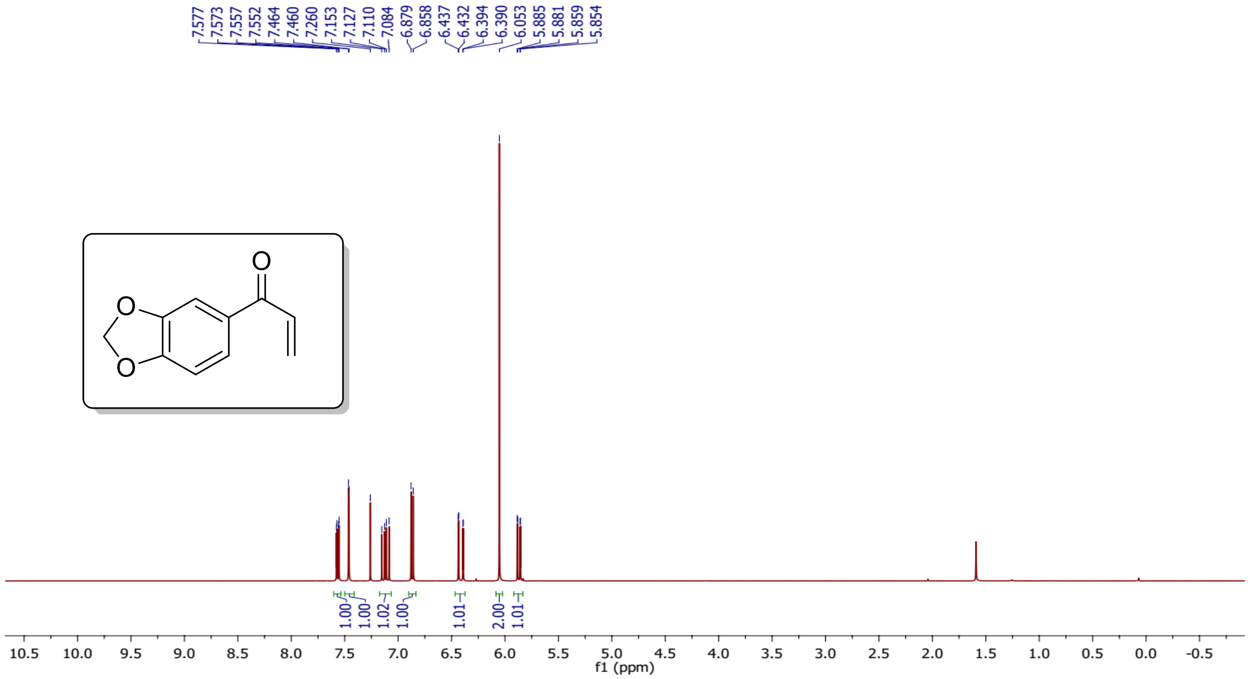

^{1}H NMR Spectrum of Compound **48r** (400 MHz, $CDCl_3$)

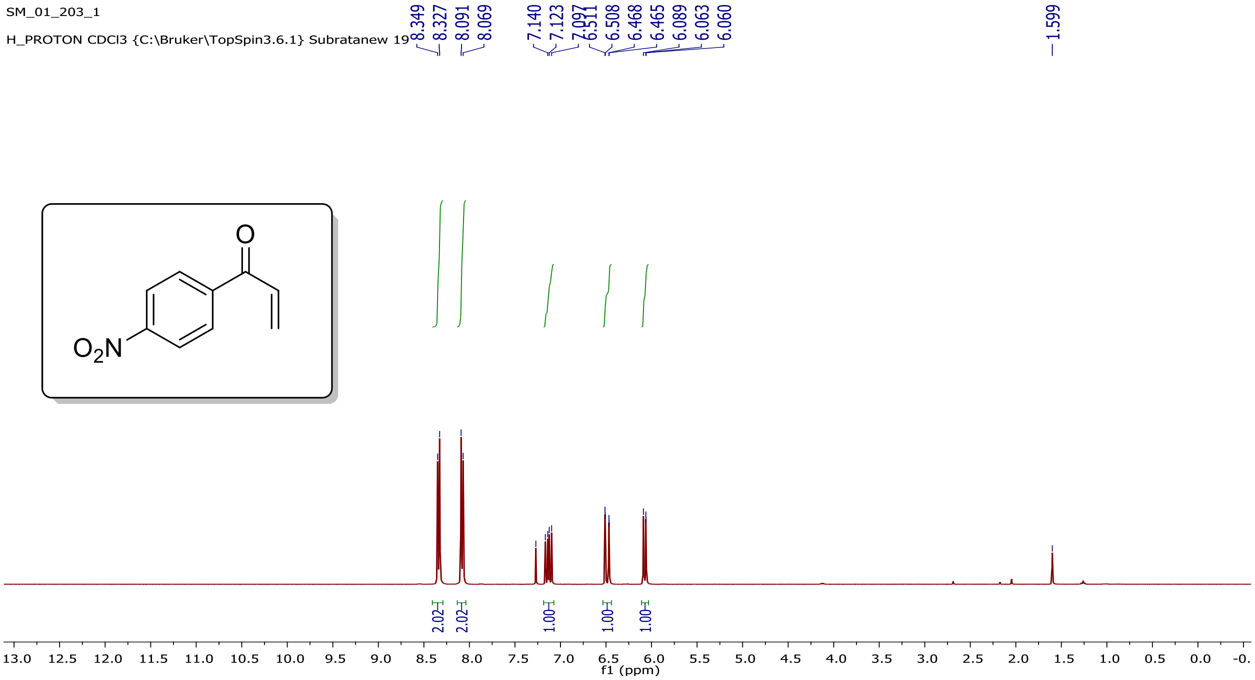

^{1}H NMR Spectrum of Compound **48s** (400 MHz, $CDCl_3$)

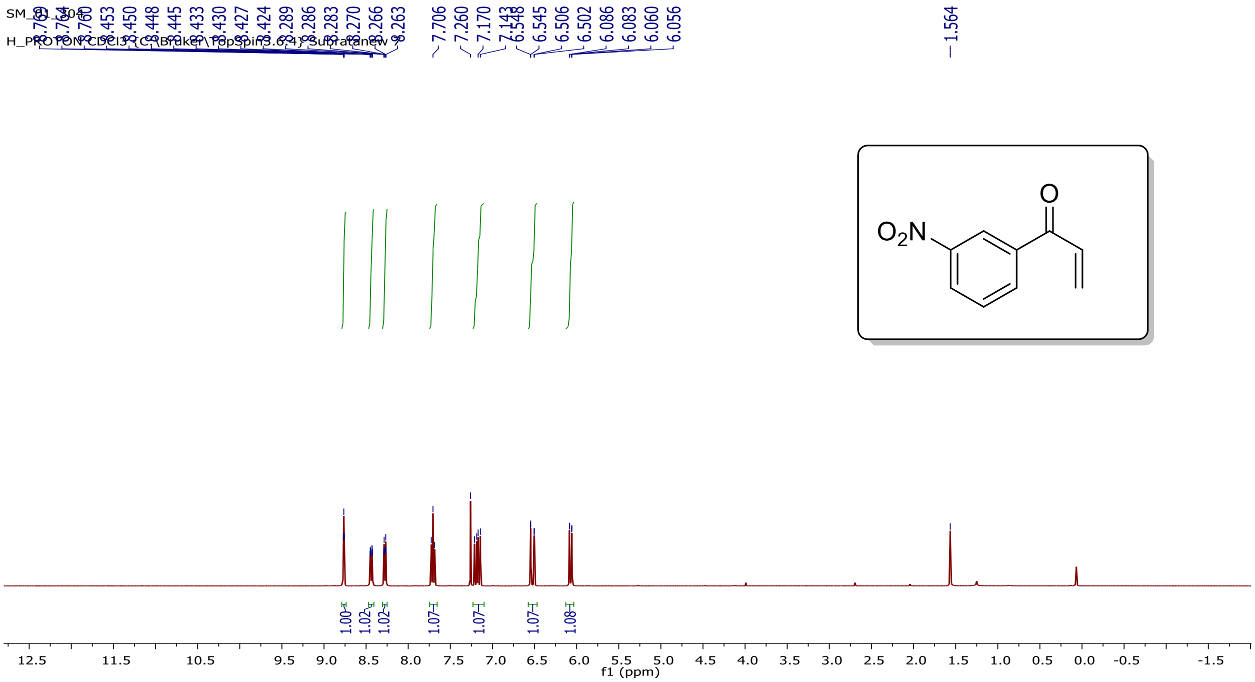

^{1}H NMR Spectrum of Compound **48t** (400 MHz, $CDCl_3$)

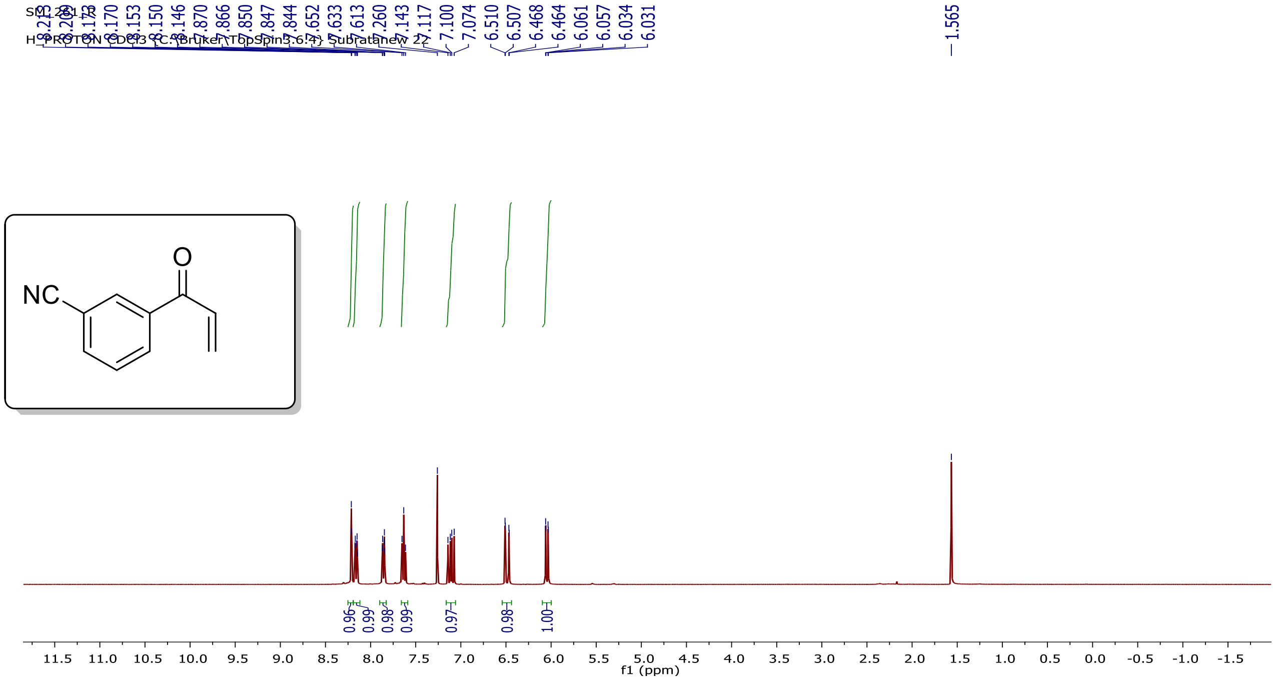

^{1}H NMR Spectrum of Compound **48u** (400 MHz, $CDCl_3$)

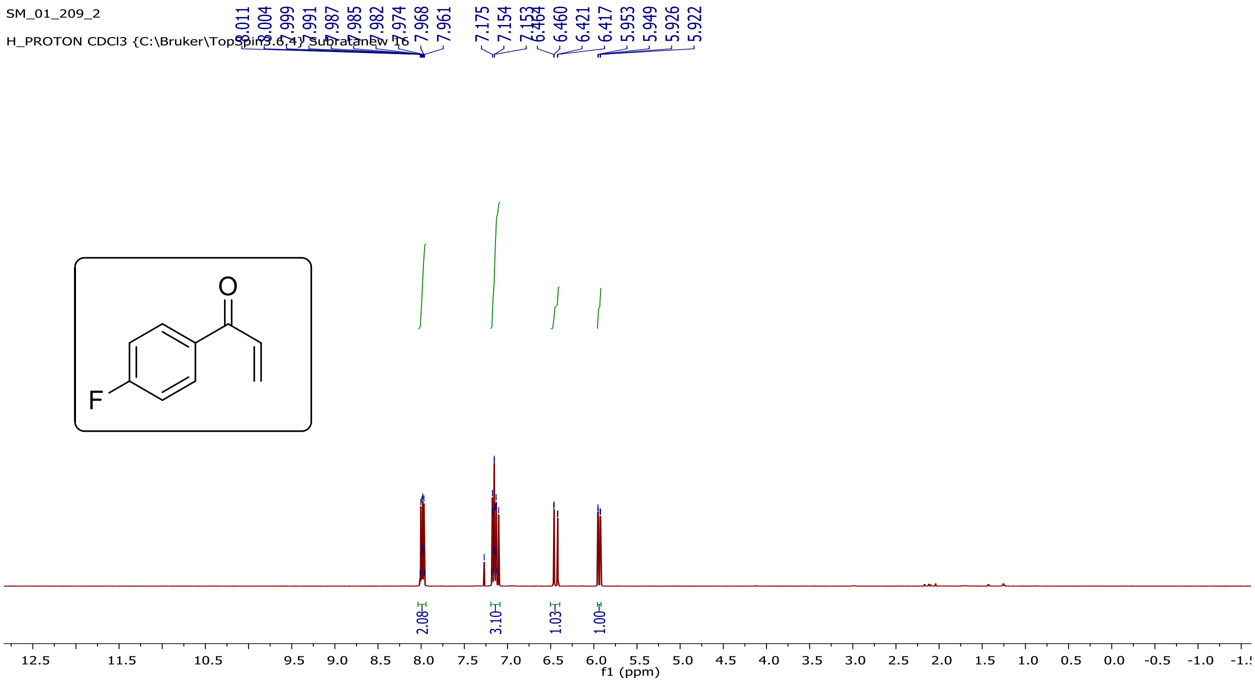

^{1}H NMR Spectrum of Compound **48v** (400 MHz, $CDCl_3$)

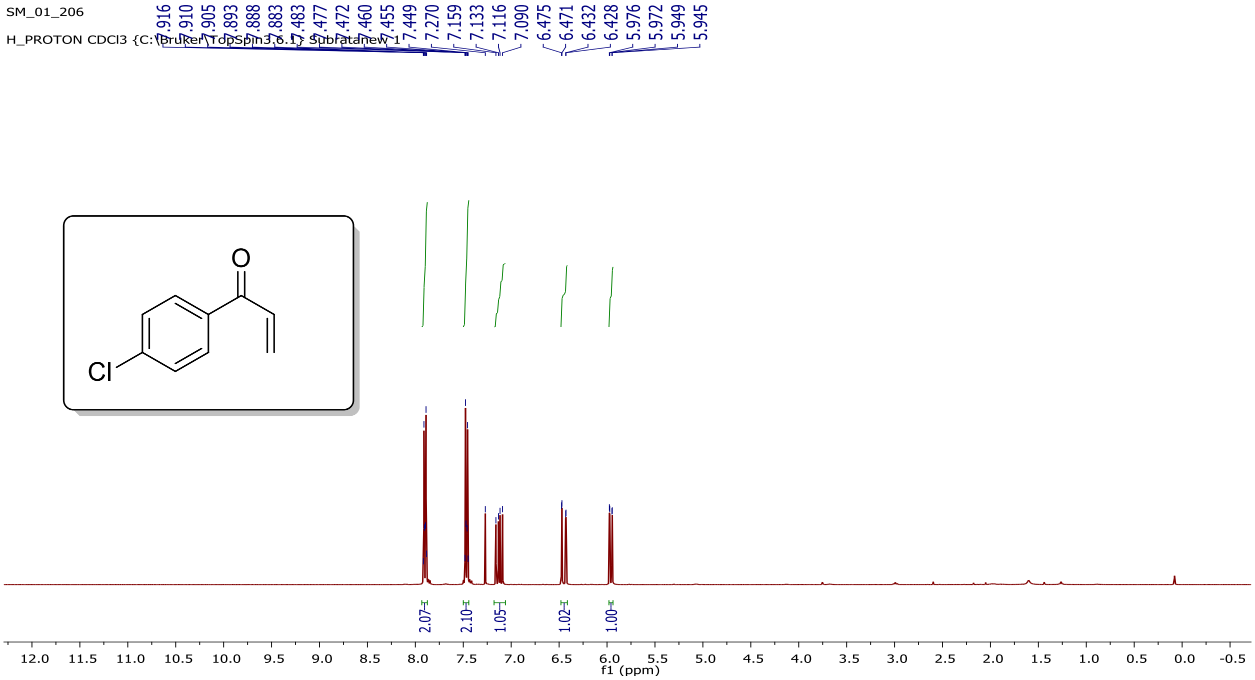

^{1}H NMR Spectrum of Compound **48w** (400 MHz, $CDCl_3$)

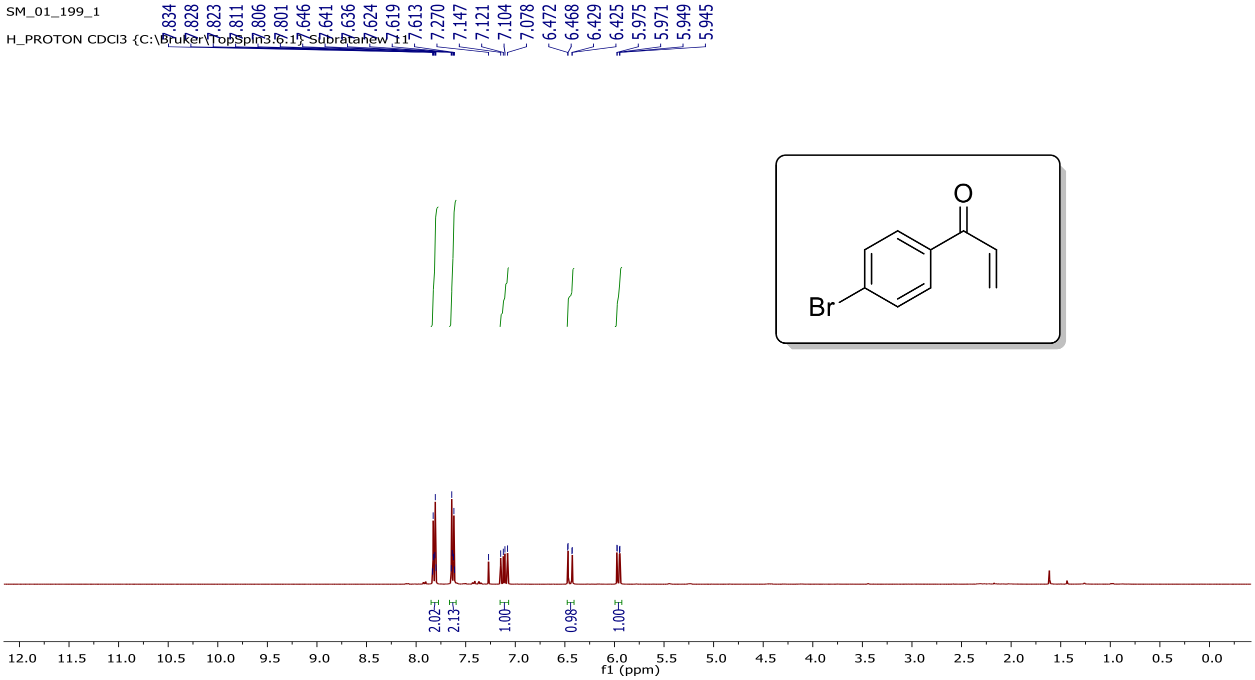

^{1}H NMR Spectrum of Compound **48x** (400 MHz, $CDCl_3$)

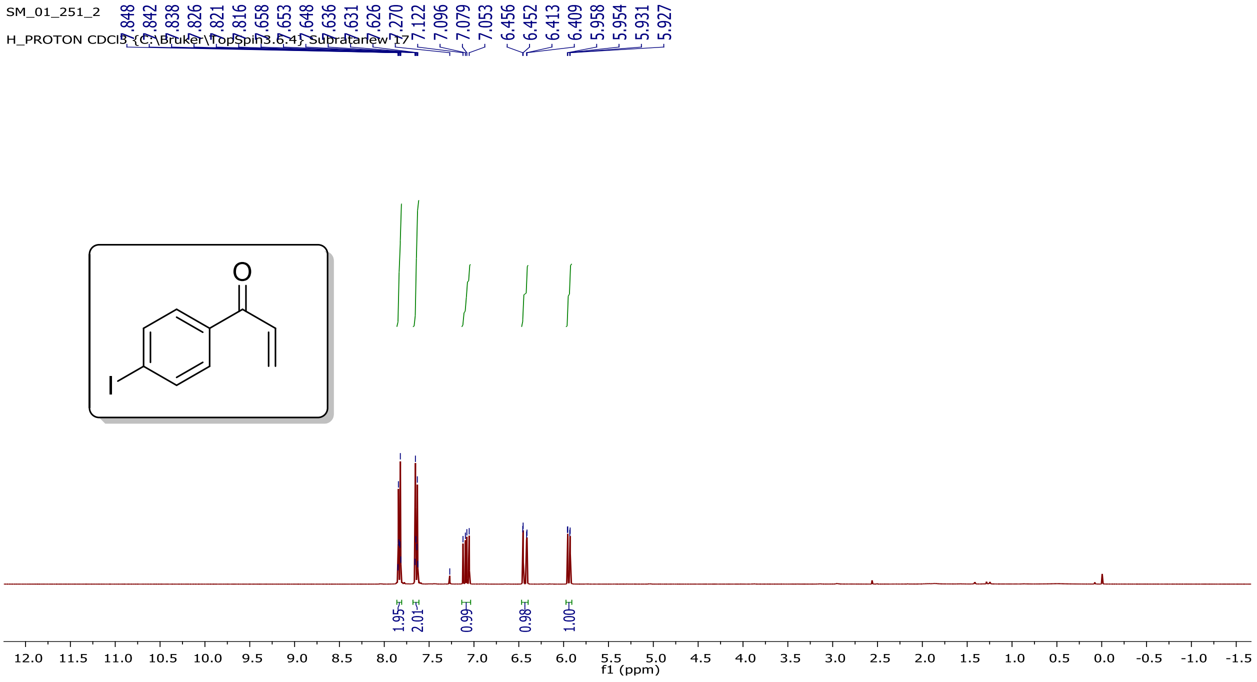

^{1}H NMR Spectrum of Compound **48y** (400 MHz, $CDCl_3$)

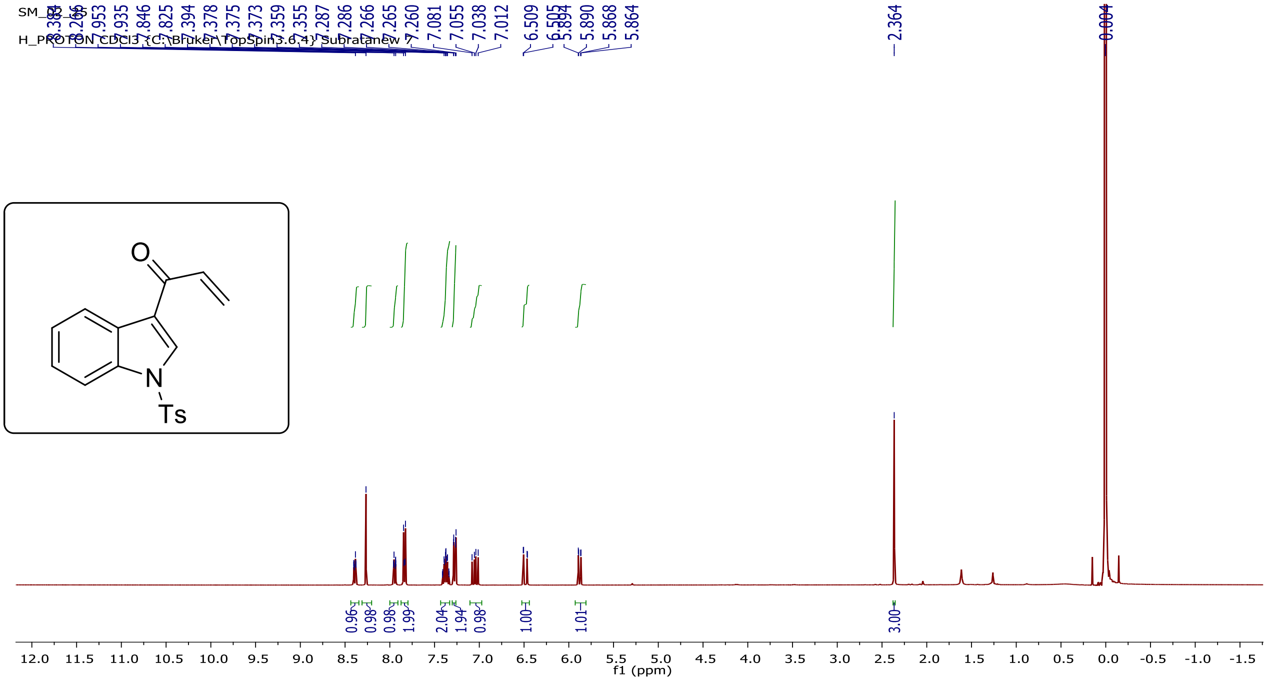

^{1}H NMR Spectrum of Compound **44** (400 MHz, $CDCl_3$)

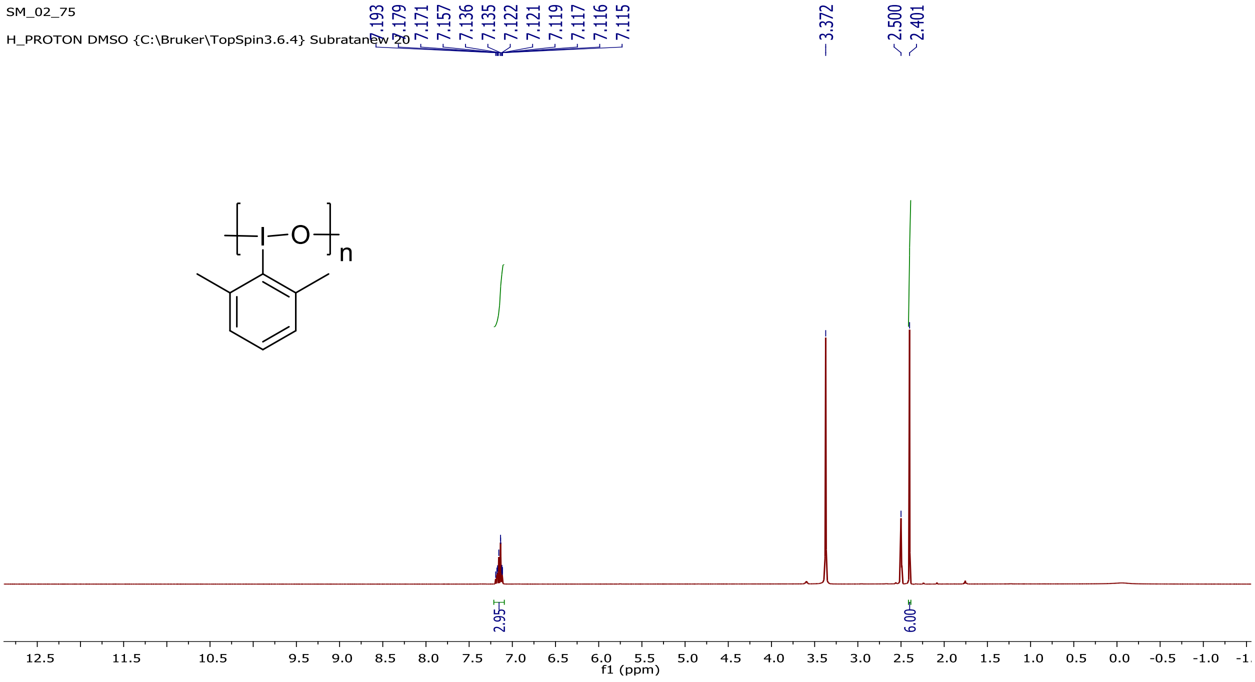

^{1}H NMR Spectrum of Compound **11** (400 MHz, DMSO-*d6*)

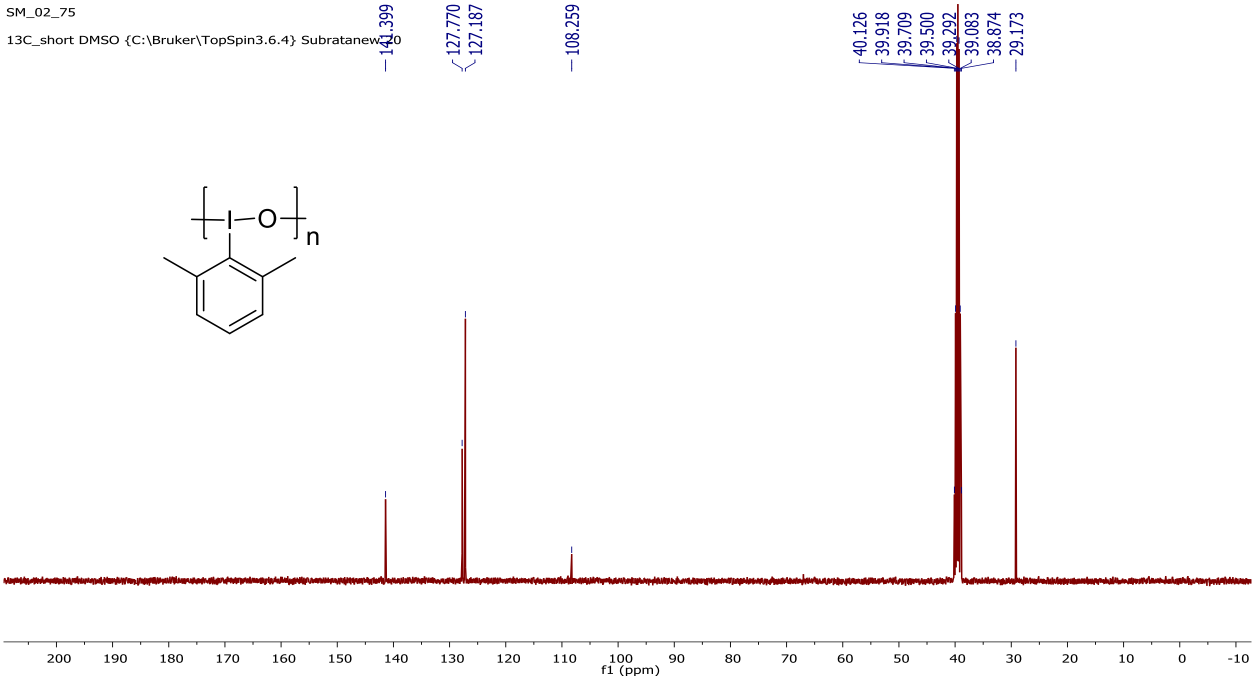

^{13}C NMR Spectrum of Compound **11** (101 MHz, DMSO-*d6*)

NMR Spectra of the Crude Reaction Mixture Before and After Addition of Triethylamine

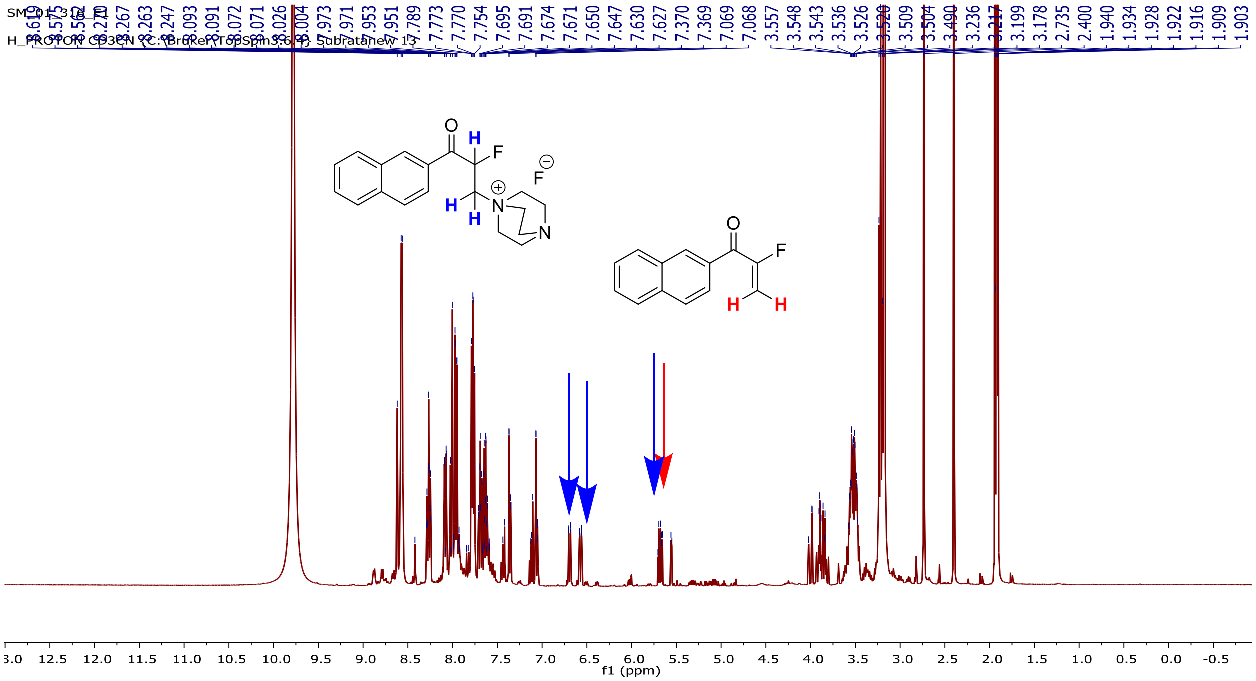

^{1}H NMR Spectrum of Crude Umpolung MBH Fluorination Reaction Mixture (400 MHz, CD_3CN) before addition of Et_3N.

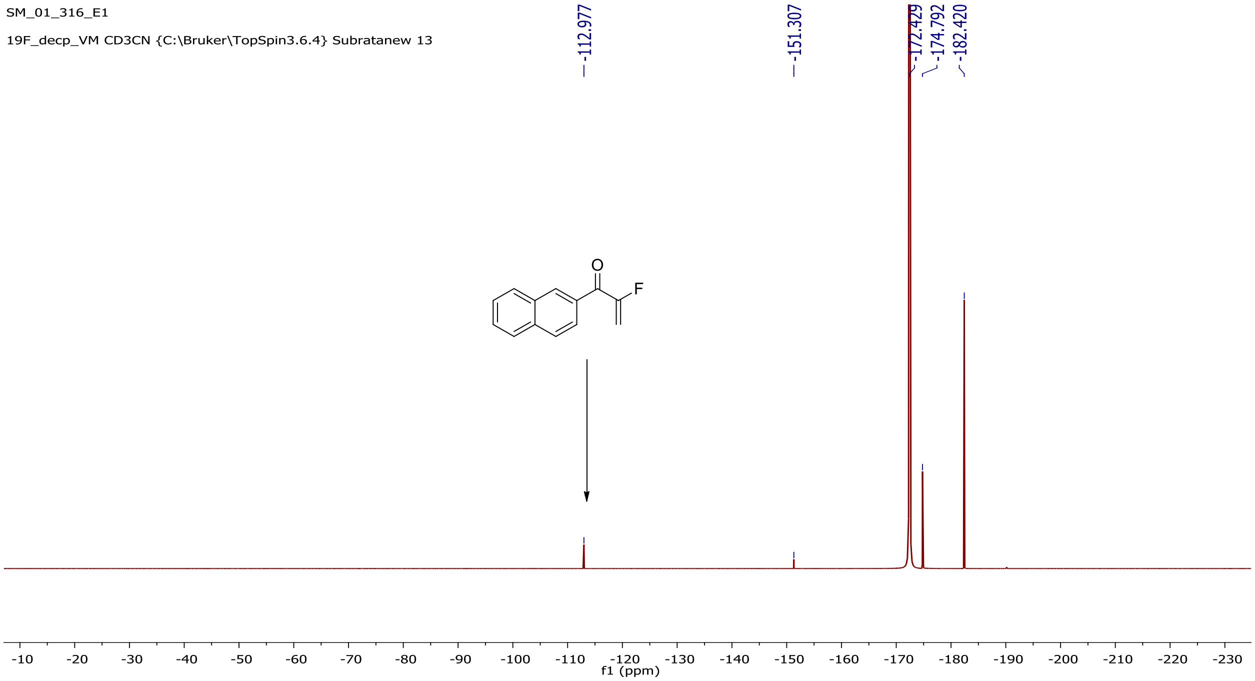

^{19}F NMR Spectrum of Crude Umpolung MBH Fluorination Reaction Mixture (376 MHz, CD_3CN) before addition of Et_3N.

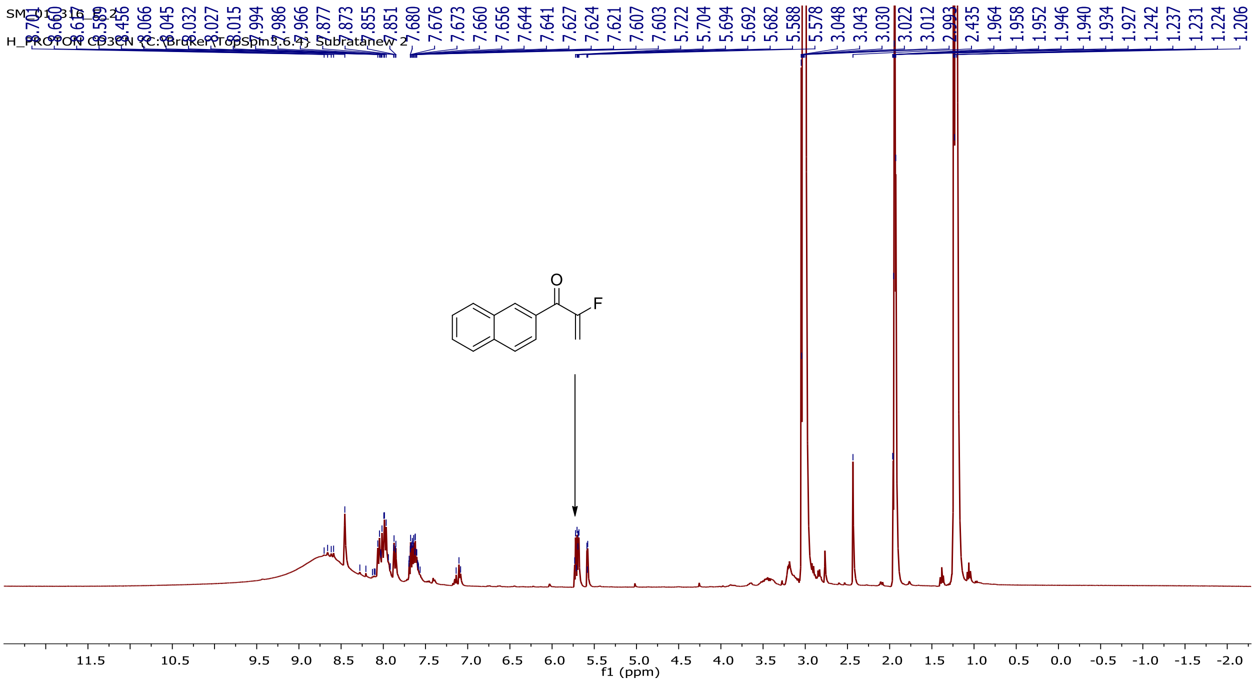

^{1}H NMR Spectrum of Crude Umpolung MBH Fluorination Reaction Mixture (400 MHz, CD_3CN) after addition of Et_3N.

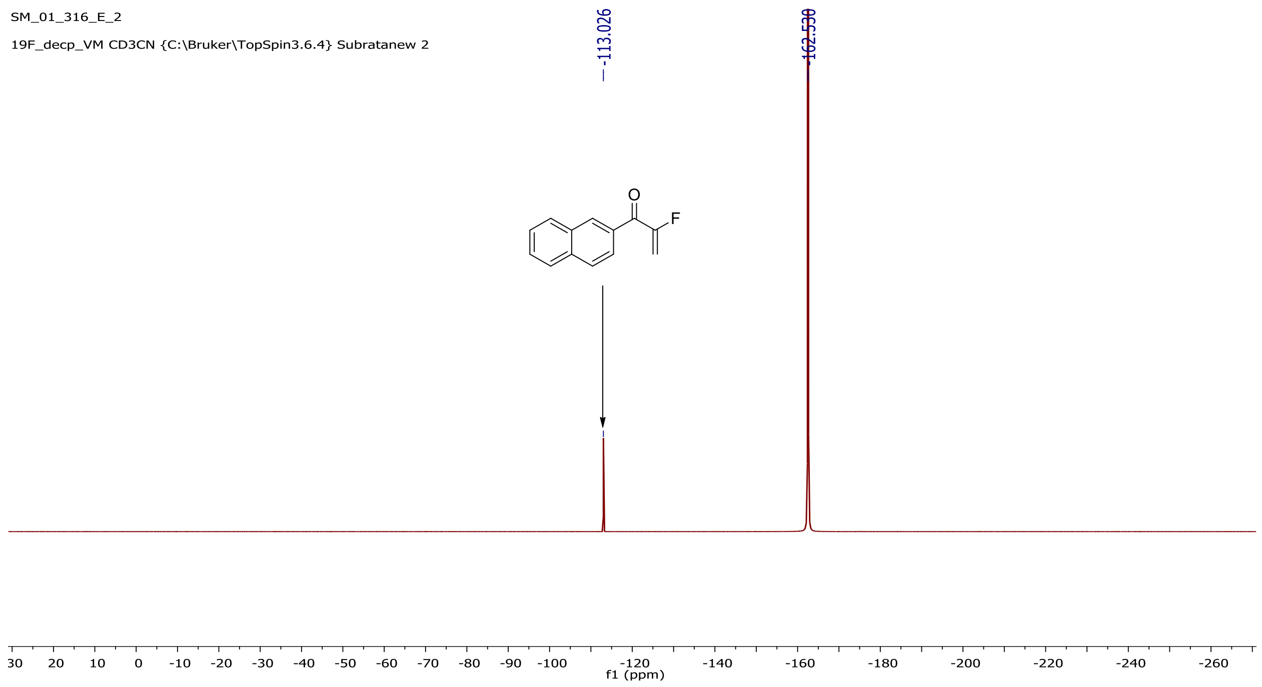

^{19}F NMR Spectrum of Crude Umpolung MBH Fluorination Reaction Mixture (376 MHz, CD_3CN) after addition of Et_3N.
